# Semi‐automated assessment for NatureServe subnational conservation status ranks for state floras

**DOI:** 10.1002/aps3.70046

**Published:** 2026-03-19

**Authors:** Julia H. Prins, Joey Shaw

**Affiliations:** ^1^ Department of Biology, Geology, and Environmental Science The University of Tennessee at Chattanooga 615 McCallie Avenue Chattanooga Tennessee USA

**Keywords:** area of occupancy, extinction risk, Feature Manipulation Engine (FME), GIS, modeling, range extent, RARECAT, S‐ranks

## Abstract

**Premise:**

Conservation status ranks measure the potential risk of extinction for species at global, national, and subnational levels, taking into account rarity, threats, and trends. These assessments are largely incomplete due to funding and resource limitations.

**Methods:**

This study addressed this problem by identifying five semi‐automated methods to assign provisionary NatureServe subnational conservation status ranks (S‐ranks) to state floras. These involved (1) estimation: (1A) calculating the mode S‐rank in surrounding states and (1B) assigning an S‐rank based on percentage of counties with occurrence records; and (2) calculating criteria and assigning an S‐rank based on the NatureServe rank calculator using (2A) a Feature Manipulation Engine (FME) workflow, (2B) NatureServe's Rapid Analysis of Rarity and Endangerment Conservation Assessment Tool (RARECAT), and (2C) ArcGIS. S‐ranks were assigned to Tennessee's native vascular flora using each method and evaluated for accuracy against published NatureServe S‐ranks, human rankings, and surrounding state S‐ranks.

**Results:**

ArcGIS was identified as a suitable method for future ranking efforts due to its batch processing capabilities, accessibility, and agreement with NatureServe S‐ranks. Therefore, a manual was created on how other states may implement this method to update and complete their S‐ranks.

**Discussion:**

By using these methods, states can more efficiently achieve comprehensive and current S‐ranks, enhancing plant conservation by providing a more complete understanding of subnational extinction risk.

Conservation status ranks measure potential extinction risk at global, national, or subnational levels, through consideration of rarity, threats, and species population trends. Maintaining current ranks is crucial for making informed conservation decisions as these ranks impact development initiatives, guide land management practices, provide evidence for policymakers, and help establish conservation priorities (Meynell, 2005; Rodrigues et al., 2006; Master et al., 2012; Havens et al., 2014). The need for conservation status ranks is particularly crucial for plant species, given their essential role in ecosystem health and the growing threat of extinction many face (Millennium Ecosystem Assessment, 2005; NatureServe, 2023; Bachman et al., 2024). However, limited protection, insufficient funding, and lack of attention has led to a significant gap in understanding their extinction risk (George et al., 1998; Wandersee and Schussler, 1999; Stein and Gravuer, 2008; Havens et al., 2014; U.S. Fish and Wildlife Service, 2020, 2024). Many ranking systems face challenges in updating and maintaining accurate conservation status ranks for plants, and as a result, numerous rankings remain outdated and incomplete. Under the NatureServe ranking system, global assessments (G‐ranks) are completed for 81% of North American plant species and national assessments (N‐ranks) are completed for 56% of the United States flora (NatureServe, 2024). On average, subnational assessments (S‐ranks) are completed for 57% of each state's flora, with six states (in descending order: West Virginia, Virginia, Vermont, Hawaii, Delaware, and North Carolina) having over 90% of their flora ranked (Figure 1) (NatureServe, 2024).

**Figure 1:**
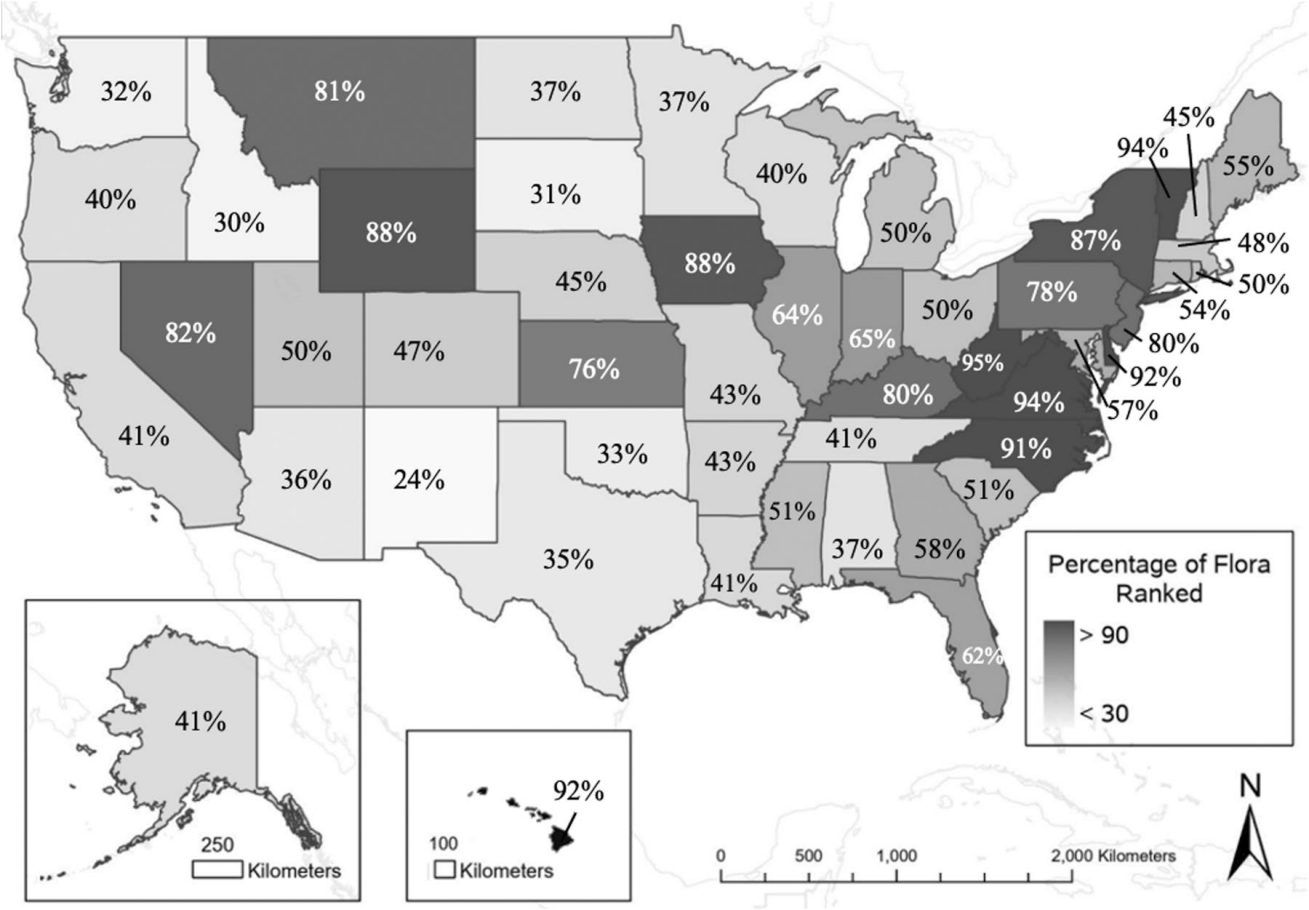
Percentage of species for each state with a subnational rank (S‐rank) under the NatureServe system. The number of species and assigned S‐ranks for each state were based on published data in NatureServe Explorer as of November 2024. The number of species per state ranged from 1480 to 6793 with an average of 2980.

Due to the absence of conservation status ranks for many species and the lack of resources available to update those that are established, innovative solutions must be explored. Traditional assessments require a species‐by‐species evaluation by a panel of experts who consult published literature, occurrence records, and distribution data. This approach requires significant time and relies on extensive data availability (IUCN, 2025). Automated assessments are gaining popularity as they accelerate species assessment while remaining reliable and informative. They help to combat the need for large amounts of human resources and time by batch calculating criteria and providing preliminary assessments (Dauby et al., 2017; Walker et al., 2023). Several studies have demonstrated strong agreement between automated and traditional assessments, supporting their effectiveness at assessing conservation status (Bland et al., 2014; Dauby et al., 2017; Pelletier et al., 2018; Stévart et al., 2019; Zizka et al., 2020b). Importantly, automated methods are useful for identifying unassessed species at high risk of extinction, aiding in conservation prioritization (Bland et al., 2014; Pelletier et al., 2018; Stévart et al., 2019). They also address taxonomic biases in current assessments, which tend to focus on woody perennials, economically important plants, and charismatic species (Nic Lughadha et al., 2020). Furthermore, the systematic and data‐driven nature of automated assessments enhances objectivity and consistency compared to expert‐based evaluations, which may be subject to bias (Zizka et al., 2024). Beyond conservation status assessments, these tools can support studies involving species range size, habitat change, and temporal trends in extinction risk (Lee et al., 2019).

There are several organizations worldwide that generate conservation status ranks at various levels for varying taxa. NatureServe is a nonprofit organization that assesses species and ecosystems at the global, national, and subnational levels, ranking them from Critically Imperiled to Secure. Subnational ranks are completed by state‐level partner organizations such as natural heritage programs, which employ NatureServe‐developed methodologies to assess species at the state, province, or territory level. A NatureServe rank consists of two parts: (1) a letter denoting the geographic scale (“G” for global, “N” for national, and “S” for subnational) and (2) a number denoting status (“1” for Critically Imperiled, “2” for Imperiled, “3” for Vulnerable, “4” for Apparently Secure, and “5” for Secure; Master et al., 2012). In the early 2000s, NatureServe developed a rank calculator to standardize and automate the ranking process (NatureServe, 2020b). There are 10 criteria used in the calculator, which fall under three categories: rarity, trends, and threats. Point weightings are assigned to criteria based on designated ranges determined by NatureServe, making the process more transparent and repeatable. These ranges and point values are standardized and do not change with state size or geographic level of ranking (global, national, or subnational).

This study identified five semi‐automated methods to assign subnational conservation status ranks (S‐ranks) to species using the NatureServe system. Each method was used to assign S‐ranks to the native vascular flora of Tennessee; these ranks were then evaluated for accuracy against published NatureServe S‐ranks, human rankings, and surrounding state S‐ranks. Additionally, a manual was created on how other states may implement these methods to update and complete their S‐ranks. Ultimately, this will result in a greater understanding of species extinction risks, thereby guiding further improvements in conservation efforts.

## METHODS

### Species list

A species list was generated using the manuscript of the second edition of the *Guide to the Vascular Plants of Tennessee* (Shaw et al., in press), excluding all infraspecific taxa. This consisted of 3033 species in 923 genera and 192 families. Based on the designations in the *Guide to the Vascular Plants of Tennessee*, 572 were non‐native species and were automatically assigned a rank of SNA (Not Applicable). Conservation status ranks do not apply to non‐native species because they are “not a suitable target for conservation activities” (Master et al., 2012). NatureServe guidelines state that a subnational rank cannot be more secure than the global or national rank (Master et al., 2012); therefore, 26 species were automatically assigned a rank of S1 for having a global rank of G1 and two species were assigned a rank of S1 for having a national rank of N1 (NatureServe, 2024). One species was assigned a rank of SH (Possibly Extirpated) due to a global status of GH (Possibly Extinct), 23 species were assigned a rank of SH for being possibly extirpated in Tennessee (TN), and five species were presumed extirpated in TN and assigned a rank of SX (NatureServe, 2024). After these automatic rank assignments, S‐ranks were generated for the remaining 2404 native species using two estimation methods independent of the NatureServe ranking system: (1) assigning an S‐rank based on mode S‐rank in surrounding states and (2) determining an S‐rank based on the percentage of counties in TN where the species was documented by a herbarium specimen. All 2404 species were ranked again using three systematic methods based on NatureServe's rank calculator: (1) a Feature Manipulation Engine (FME) workflow, (2) NatureServe's Rapid Analysis of Rarity and Endangerment Conservation Assessment Tool (RARECAT), and (3) a series of geoprocessing tools in ArcGIS Pro (Esri, Redlands, California, USA).

### Estimation ranking methods

#### Ranking based on surrounding state ranks

**Figure 2:**
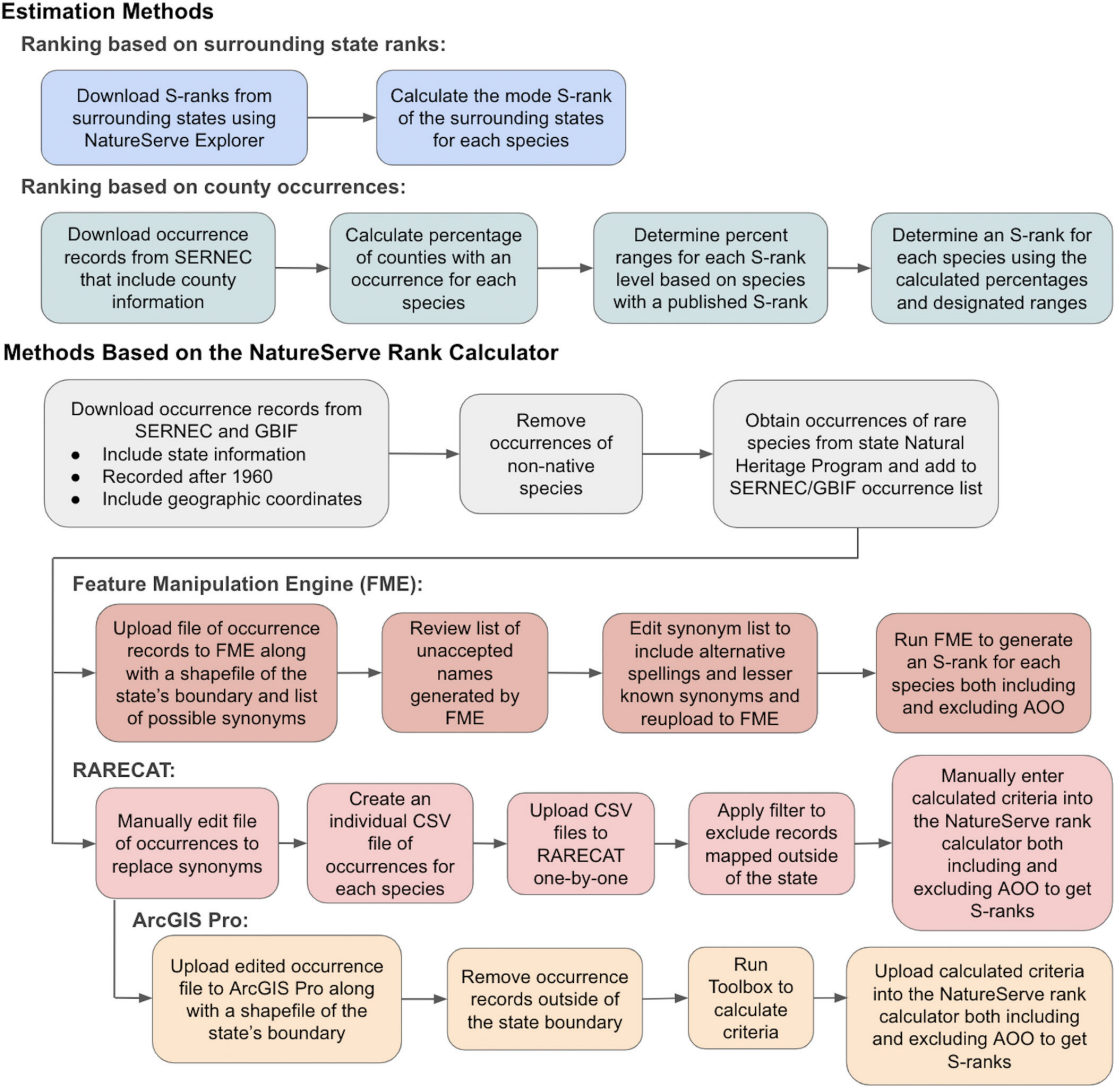
Workflow for generating ranks using the five semi‐automated methods. AOO, area of occupancy; GBIF, Global Biodiversity Information Facility; SERNEC, Southeast Regional Network of Expertise and Collections; RARECAT = Rapid Analysis of Rarity and Endangerment Conservation Assessment Tool.

S‐ranks were assigned to each species based on the S‐ranks in the states directly surrounding TN using NatureServe Explorer (Figure 2). The S‐rank for TN was estimated by looking at the S‐ranks assigned in Alabama, Arkansas, Georgia, Kentucky, Mississippi, Missouri, North Carolina, and Virginia. This method was selected because much of a state's flora can be found in neighboring states due to similar climates and geology. S‐rank data for these states were downloaded from NatureServe Explorer in January 2024, and the mode rank of the surrounding states for each species was applied as the rank estimate for TN. Mode was selected as it was less affected by outliers (for example, species at the edge of a range in one state versus occupying the entirety of others). This method generated ranks for 1319 of the 2404 species (55%). Not all species could be ranked using this method for one of three reasons: (1) not all TN species were found in surrounding states; (2) S‐ranks assigned in the surrounding states varied considerably, resulting in no mode; and (3) most states did not have ranks assigned to their entire flora, resulting in only a single state rank or no data available for some species.

#### Ranking based on county occurrences

Each species was ranked based on the percentage of the 95 counties in TN where the species had a known herbarium occurrence record (Figure 2). This percentage was obtained using data downloaded from the Southeast Regional Network of Expertise and Collections (SERNEC; SERNEC Data Portal, 2024). The download was made in February 2024 and only included records that had state locality listed as Tennessee and county locality listed as one of the 95 counties in TN. To determine what percentages were associated with each S‐rank, the percentage of counties was calculated for the 608 species with a published NatureServe S‐rank of S1–S5 assigned by the Tennessee Natural Heritage Program (TNHP). From these percentages, mean, median, standard deviation, and interquartile range were calculated for each S‐rank level to understand the distribution of percentages. Guided by the interquartile range for each level, the ranges of percentages and their associated S‐ranks were determined (S1 = 1–5% of counties; S2 = 6–9%, S3 = 10–20%, S4 = 21–42%, S5 > 42%). This method generated ranks for 2390 of the 2404 species (99%).

### Defining calculated criteria

The three systematic methods (FME, RARECAT, and ArcGIS Pro) were based on NatureServe's rank calculator and used occurrence records with geographic coordinates to calculate the three criteria of range extent, area of occupancy, and number of occurrences. When using the calculator to assign a rank, a minimum of two criteria are needed (Faber‐Langendoen et al., 2012). Range extent is defined as the smallest possible boundary that encompasses all known occurrence points of a taxon. Area of occupancy (AOO) is the area within its range extent that is occupied by the taxon. It is calculated by counting the number of 2 × 2 km grid cells that contain a unique occurrence record. This reflects that a taxon may not occupy the entirety of its range extent due to unsuitable or unoccupied habitat. Number of occurrences is the number of “on‐the‐ground” locations where a taxon is present (Master et al., 2012). To eliminate the effect of including multiple occurrence records of the same “on‐the‐ground” location, it is recommended that a minimum of 1 km separation distance be present between occurrences. If records are less than 1 km apart, they should be counted as a single occurrence (NatureServe, 2020a). An S‐rank was calculated for each species using the three systematic methods, based on the weightings of range extent, AOO, and number of occurrences used by NatureServe.

### Obtaining occurrence records

The process for calculating the three criteria varied slightly across the methods, but all used occurrence data downloaded in January 2024 from SERNEC and the Global Biodiversity Information Facility (GBIF), which included research‐grade iNaturalist observations. Records were downloaded using the following parameters: included geographic coordinates, state locality listed as Tennessee, and recorded after 1960 as anything prior was considered historic (GBIF.org, 2024; SERNEC Data Portal, 2024). Pre‐1960 records were excluded from the dataset for two reasons: (1) the geographic coordinates associated with the historical data were most likely inaccurate due to the inaccessibility of precise global positioning system (GPS) devices at the time, and (2) given that the occurrence points were over 60 years old, there was a higher chance that the populations no longer existed at that location. The age of a record to be considered historic can vary, but for this study 1960 was chosen because it was the cutoff date Janel Johnson used in her FME workflow and consistency was needed across the three methods (personal communication, J. Johnson, Nevada Division of Natural Heritage, 2024). This cutoff date is consistent with the recommendation of Zizka et al. (2020a), who advise excluding records collected before the end of World War II due to their imprecise geographic coordinates. These early records often rely on georeferencing from locality descriptions, which can be unreliable given historical changes in place names, administrative boundaries, and mapping standards.

The downloaded list of occurrence records was manually edited to remove all records of non‐native species based on the designations found in the second edition of the *Guide to the Vascular Plants of Tennessee* (Shaw et al., in press). Species that did not have any records with geographic coordinates available on GBIF or SERNEC could not be ranked using FME, RARECAT, or ArcGIS Pro.

Data for the rare species of TN (Crabtree, 2021) were obtained from the internal database of the Tennessee Department of Environment and Conservation Division of Natural Areas (TDEC‐DNA) due to hidden locality strings on GBIF and SERNEC. Data were obtained in January 2024, and all records before 1960 were removed from the dataset (Tennessee Department of Environment and Conservation Division of Natural Areas, 2024).

### Methods based on the NatureServe rank calculator

#### Ranking using a Feature Manipulation Engine

The FME workflow used to assign S‐ranks in this study was developed by Janel Johnson from the Nevada Division of Natural Heritage. Occurrence records were uploaded to the workflow, which calculated an S‐rank for all species that had a minimum of one occurrence record in the uploaded file (Figure 2). Along with occurrence records, a shapefile of TN's political boundary was uploaded to FME, and each occurrence point was mapped to ensure it fell within the state border. If the occurrence point fell outside of TN, it was removed and not included in the ranking. A file containing a list of synonyms for each species was also uploaded to FME to account for taxonomic discrepancies. Because most of the occurrence records were obtained from SERNEC, a list of possible synonyms was downloaded from the Tennessee State Flora on SERNEC, which sourced synonyms from the United States Department of Agriculture Plant List of Attributes, Names, Taxonomy, and Symbols Database (USDA PLANTS; SERNEC Data Portal, 2024). In the FME workflow, each occurrence record was cross‐checked against the list of possible synonyms to ensure that all occurrences for a given species were included in its S‐rank calculation despite varying taxonomy. After the initial pass, a list of names found in the occurrence record file that did not match an accepted scientific name or a listed synonym was generated by the FME workflow. This list was manually examined to determine the reasoning for species rejection, which fell under three main categories: (1) the species was not known to the state of TN and most likely had false locality information, (2) the name was a lesser‐known synonym that was not included in the original synonym list, or (3) the scientific name was misspelled. Species not known to TN were removed from the dataset, and the synonym list was edited to include lesser‐known synonyms and common alternative spellings. The updated synonym list and occurrence records were reuploaded to the workflow, and occurrence data were then sent through a second time. The workflow grouped accepted occurrence records by species and calculated range extent, AOO, and number of occurrences for each species. The three criteria were then used to generate an S‐rank by FME based on the weightings used in NatureServe's rank calculator.

After examining the first output of S‐ranks calculated by the workflow, no S5 ranks were generated, even for common species present throughout TN. The data were analyzed further, and it was found that when only considering rarity in S‐rank calculations, as being done in this study, AOO may heavily skew data toward Imperiled. This is because AOO requires such a high threshold for a species to be considered common; instead, it is more helpful in showing that a species is rare (personal communication, Amanda Eberly, NatureServe, 2024). To combat this, data were ranked a second time, but this time only included range extent and number of occurrences in the calculation. Once completed, the FME workflow generated S‐ranks for 2034 of the 2404 species (85%). Not all species could be ranked using this method because access to the FME workflow was unavailable after January 2024, so that problematic species discovered after this date could not be rerun to get an accurate rank. Additionally, the rare species of TN could not be ranked using FME because data were obtained from TDEC‐DNA after this deadline.

#### Ranking using RARECAT

S‐ranks were generated using an RStudio (Posit Team, 2025) Shiny web application built by NatureServe, referred to as RARECAT (Figure 2). This app calculated range extent, AOO, and number of occurrences for a single species at a time using occurrence data uploaded as a comma‐separated values (CSV) file. The GBIF and SERNEC occurrence records used in the FME workflow were manually edited to replace synonyms and misspellings using the same list uploaded to FME. From this dataset, unique CSV files of occurrence records were created for each species and uploaded to RARECAT individually. In the app, a filter was applied to exclude all records outside of TN and the accepted occurrence points were used to calculate range extent, AOO, and number of occurrences. The values of these three criteria were manually entered into the NatureServe rank calculator, which generated an S‐rank for each species. All species were ranked a second time using the NatureServe rank calculator but only including range extent and number of occurrences in the calculation to eliminate the skew towards Imperiled caused by AOO. This method generated S‐ranks for 2254 of the 2404 species (94%).

#### Ranking using ArcGIS Pro

ArcGIS Pro Version 3.0.0 was used to generate S‐ranks using a variety of geoprocessing tools (Figure 2). The same GBIF/SERNEC dataset used in the other systematic methods was uploaded to ArcGIS Pro along with a shapefile of TN's political boundary, and any occurrence points that fell outside the boundary were removed. A model with a series of geoprocessing tools was created to sort through the uploaded data, create a unique layer for each species, and calculate range extent, AOO, and number of occurrences for each species. The calculated criteria were then batch‐uploaded to the NatureServe rank calculator to generate S‐ranks. All species were ranked a second time using the calculator but only including range extent and number of occurrences in the calculation to eliminate skew caused by AOO. This method generated S‐ranks for 2254 of the 2404 species (94%). A Toolbox containing this model was created in ArcGIS Pro that allows users to upload a layer of occurrence points; from this, range extent, AOO, and number of occurrences are automatically calculated for each species. This Toolbox was made available on GitHub (https://github.com/juliaprins/S-rank-ArcGIS-Manual), along with a user's manual that includes instructions on how to convert rarity criteria for use in NatureServe's rank calculator (Appendix S1).

### Analyzing accuracy of the semi‐automated methods

To compare S‐ranks generated using the semi‐automated methods to traditional assessments, the percentage of time the ranks from each of the five methods agreed with published NatureServe S‐ranks assigned by TNHP was calculated. Most of these published S‐ranks were assessed traditionally prior to the release of the NatureServe rank calculator and therefore were not influenced by this tool.

#### Botanist blind S‐rank assessment

To compare semi‐automated methods to human rankings, a subset of 100 randomly selected species was subject to human rank assessment by five TN botanists in October 2024. Each botanist was randomly assigned 40 species to blindly assign an S‐rank without looking at the S‐ranks assigned in this study or published NatureServe S‐ranks. Botanists were asked to assign a rank based on immediate reaction and to not spend time researching the species. At its completion, each of the 100 species had a human assessment completed by two botanists; these were then analyzed to assess agreement between botanists. If the rank assigned by two botanists differed for a species, the average was taken and rounded down toward Imperiled, as is commonly done when using range ranks. These ranks were analyzed to assess agreement with the five semi‐automated methods used in this study and the published NatureServe S‐ranks assigned by TNHP.

#### Analyzing semi‐automated S‐ranks frequency distribution against five comparison states

The frequency distributions of the S‐ranks from each semi‐automated method were graphed, and the descriptive statistics of mean and standard deviation and shape measurements of skewness and kurtosis were analyzed against five surrounding states. S‐ranks were downloaded from NatureServe Explorer in January 2025 for the five surrounding states with the highest percentage of their flora ranked: Georgia, Kentucky, North Carolina, Virginia, and West Virginia (Figure 1). The frequencies of S‐ranks S1, S2, S3, S4, and S5 were graphed, and descriptive statistics and shape measurements were calculated for each state. For this analysis, all range ranks were rounded down toward Imperiled, and all graphing and calculations were completed using IBM SPSS Statistics for Mac (Version 29).

## RESULTS

### Raw data

For the county occurrence ranking method, 458,760 records were downloaded from SERNEC with state locality listed as Tennessee and county locality listed as one of its 95 counties. For the surrounding state ranking method, NatureServe ranks were downloaded for 6245 species found in Alabama, Arkansas, Georgia, Kentucky, Mississippi, Missouri, North Carolina, Tennessee, and Virginia. For the ranking methods based on the NatureServe rank calculator, 227,988 records were downloaded from GBIF and 27,505 records from SERNEC. These records included geographic coordinates, had state locality listed as Tennessee, and were recorded after 1960. An additional 5998 occurrences (recorded after 1960) were obtained from TDEC‐DNA for the rare species of TN. During the synonym curation process, a list of 11,016 possible synonyms was downloaded from SERNEC for the TN state flora. After the initial pass through FME, an additional 1022 synonyms and alternative spellings were recognized and added to the synonym list.

### Semi‐automated ranking methods

Of TN's 3033 plant species, 572 were automatically ranked as SNA for being non‐native, 28 as S1 for being Critically Imperiled at the global (G1) or national (N1) level, 24 as SH for being Possibly Extirpated, and five as SX for being Presumed Extirpated. The remaining 2404, including 586 with an existing NatureServe S‐rank, were ranked using the semi‐automated methods. For estimation methods independent from the NatureServe rank calculator, the surrounding state mode method generated ranks for 1319 species and the county percentage method generated ranks for 2390 species (Table 1). For the systematic methods based on the NatureServe rank calculator, FME generated ranks for 2034 species, RARECAT generated ranks for 2254 species, and ArcGIS Pro generated ranks for 2254 species (Table 1). Individual ranks assigned to each species from each method are in Appendix S2.

**Table 1:** Number of species generated for each S‐rank for eight semi‐automated ranking methods.

	No. of species ranked					
Ranking method	S1	S2	S3	S4	S5	Total
Surrounding state	240	92	172	203	612	1319
County percentage	502	272	416	517	683	2390
FME including AOO	497	604	701	232	0	2034
FME excluding AOO	460	305	935	269	65	2034
RARECAT including AOO	600	666	755	233	0	2254
RARECAT excluding AOO	569	385	945	278	77	2254
ArcGIS including AOO	555	717	740	242	0	2254
ArcGIS excluding AOO	538	354	1019	273	70	2254

Abbreviations: AOO = area of occupancy, FME = Feature Manipulation Engine, RARECAT = Rapid Analysis of Rarity and Endangerment Conservation Assessment Tool.

### Analyzing accuracy

**Figure 3:**
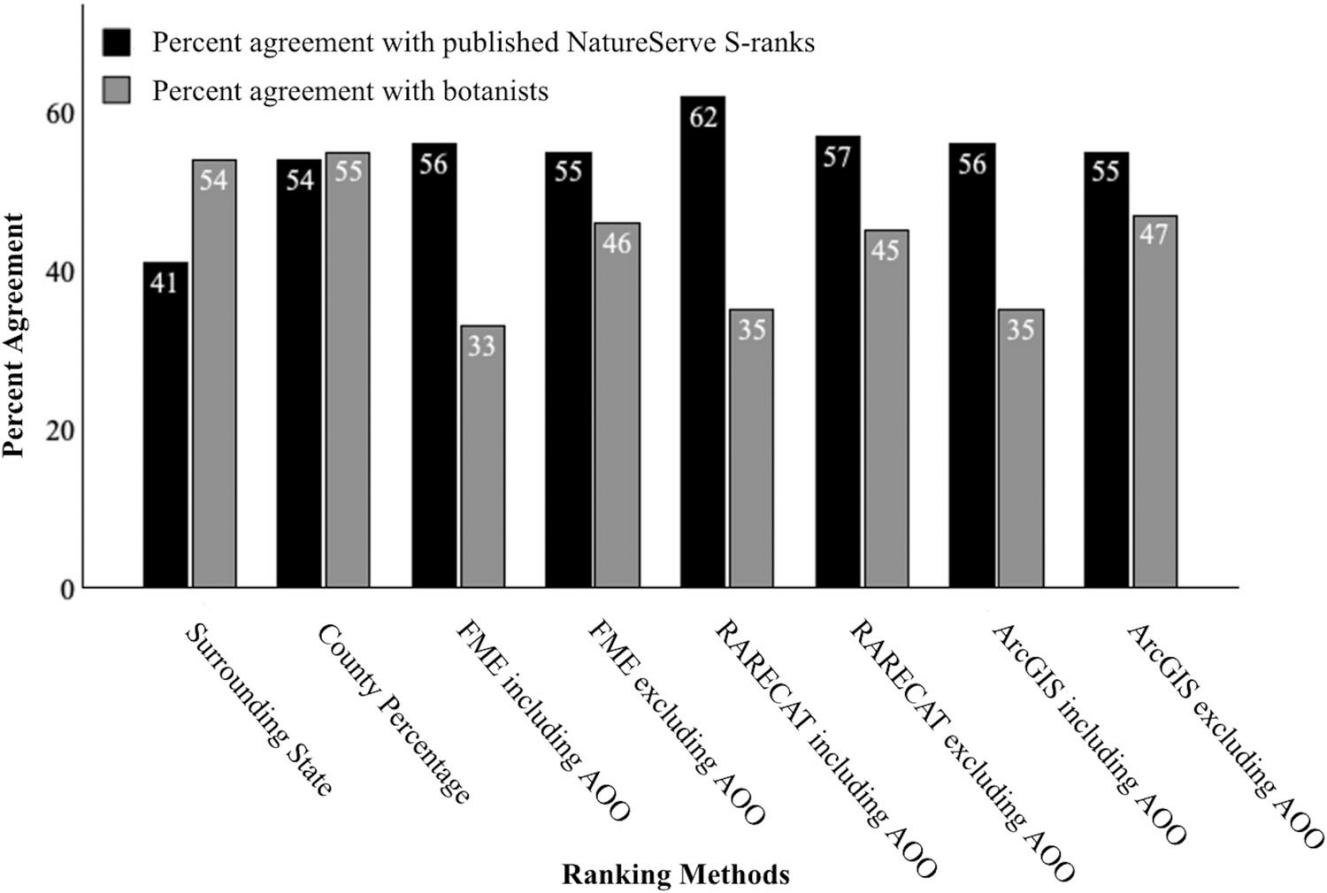
Agreement of eight semi‐automated ranking methods with published NatureServe S‐ranks and botanist‐assigned S‐ranks during blind S‐rank assessment.

The ranking methods in order of highest to lowest agreement with published NatureServe S‐ranks were: RARECAT including AOO, RARECAT excluding AOO, FME including AOO, ArcGIS Pro including AOO, FME excluding AOO, ArcGIS Pro excluding AOO, county percentage, and surrounding state (Figure 3).

#### Botanist blind S‐rank assessment

The same rank was assigned by two botanists 52% of the time. Of 48 species assigned different ranks, 65% disagreed by one rank, 23% disagreed by two ranks, and 12% only had one botanist assign a rank. Two species were not ranked by either botanist and were assigned a rank of SNR (Unranked). The ranking methods in order of highest to lowest agreement with human assessment ranks were: county percentage, surrounding state, ArcGIS excluding AOO, FME excluding AOO, RARECAT excluding AOO, ArcGIS including AOO, RARECAT including AOO, and FME including AOO (Figure 3). Twenty of the 100 species included in this assessment had a published NatureServe S‐rank assigned by TNHP, and botanists agreed with 65% of these S‐ranks. Those that disagreed did so by one rank, and a more secure rank was suggested by botanists for six out of the seven species.

#### Comparison states

The estimation methods independent of the NatureServe rank calculator (surrounding state mode and county percentage) had means ranging from 3.25 to 3.65, standard deviations from 1.5 to 1.546, skewness values from −0.699 to −0.303, and kurtosis values from −1.334 to −1.063 (Table 2). Systematic methods based on the NatureServe rank calculator (FME, RARECAT, and ArcGIS) had means ranging from 2.28 to 2.59, standard deviations from 0.957 to 1.099, skewness values from −0.084 to 0.113, and kurtosis values from −1.056 to −0.67 (Table 2). The surrounding states of Georgia, Kentucky, North Carolina, Virginia, and West Virginia had means ranging from 2.81 to 3.71, standard deviations from 1.388 to 1.5, skewness values from −0.785 to 0.134, and kurtosis values from −1.224 to −0.793 (Table 3).

**Table 2:** Descriptive statistics for the eight semi‐automated ranking methods.

Ranking method	No. of species	Mean	Standard deviation	Skewness	Kurtosis
Surrounding state	1319	3.65	1.546	−0.699	−1.063
County percentage	2390	3.25	1.500	−0.303	−1.334
FME include AOO	2034	2.33	0.968	0.059	−1.036
FME excluding AOO	2034	2.59	1.073	−0.084	−0.670
RARECAT including AOO	2254	2.28	0.969	0.109	−1.056
RARECAT excluding AOO	2254	2.52	1.099	0.054	−0.754
ArcGIS including AOO	2254	2.30	0.957	0.113	−0.994
ArcGIS excluding AOO	2254	2.55	1.074	−0.035	−0.696

Abbreviations: AOO = area of occupancy, FME = Feature Manipulation Engine, RARECAT = Rapid Analysis of Rarity and Endangerment Conservation Assessment Tool.

**Table 3:** Descriptive statistics for the five comparison states.

State	No. of species	Mean	Standard deviation	Skewness	Kurtosis
West Virginia	1642	3.46	1.500	−0.535	−1.194
Virginia	2132	3.63	1.498	−0.715	−0.951
North Carolina	2356	3.14	1.388	−0.112	−1.204
Kentucky	1415	3.71	1.437	−0.785	−0.793
Georgia	1445	2.81	1.415	0.134	−1.224

## DISCUSSION

### Botanist blind S‐rank assessment

The botanist blind S‐rank assessment allowed for comparison between human assessment rankings and ranks generated using the semi‐automated methods of this study. The results of the assessment indicated that the S‐ranks generated by the methods based on the NatureServe rank calculator (FME, RARECAT, and ArcGIS) often leaned towards Imperiled, with human assessment rankings often suggesting a more secure status. The estimation methods independent of the NatureServe rank calculator (surrounding state and county percentage) showed higher agreement with botanists than the methods based on the NatureServe rank calculator (FME, RARECAT, and ArcGIS). This again showed that botanists tended to assign more secure rankings, as the estimation methods independent of the NatureServe rank calculator generated more secure rankings than the methods based on the calculator. Similarly, botanists showed higher agreement with ranks when FME, RARECAT, and ArcGIS were run excluding AOO; these ranks were more secure than those including AOO.

The botanist blind assessment showed higher agreement with published NatureServe S‐ranks assigned by the TNHP than with S‐ranks generated by the semi‐automated methods. However, the botanists involved in this assessment frequently worked with published S‐ranks to inform conservation decisions, and several were employees of the TNHP who were involved in the original ranking of these published S‐ranks. This made it challenging to fully isolate their existing knowledge of these species’ ranks from the assessment process. Yet even with pre‐existing knowledge, during the blind S‐rank assessment, botanists disagreed with published NatureServe S‐ranks 35% of the time. Additionally, botanists disagreed with each other for 48% of species. These results demonstrate the high variability present when relying on human assessment rankings rather than a standardized method. Personal beliefs regarding the conservation status of a species were often inconsistent, making it difficult to commonly agree, which emphasized the importance of relying on standardized ranking methods free from human beliefs and biases.

### Agreement of ranking methods

The surrounding state mode ranks had the lowest agreement (41%) with published NatureServe S‐ranks (Figure 3), highlighting substantial variability in S‐ranks among states. This discrepancy may stem from several factors, including ecological differences in species’ regional abundance or distribution, methodological inconsistencies in how states assess conservation status, and administrative variation in assessment frequency, funding, or prioritization. Such interstate variability emphasizes the limitation of relying on adjacent state data for local rank estimation and reinforces the importance of conducting state‐specific assessments. Moreover, it raises important implications for interstate coordination, suggesting a need for more standardized assessment protocols or collaborative frameworks to improve consistency and comparability across state lines.

The RARECAT ranking methods including and excluding AOO had the highest agreement with published NatureServe S‐ranks (62% and 57%, respectively), with the other systematic methods just a few percentage points behind (Figure 3). The systematic methods run including AOO (FME, RARECAT, and ArcGIS Pro) showed a slightly higher agreement with published NatureServe S‐ranks than when these methods were run excluding AOO (Figure 3). It was originally thought that including AOO skewed results towards Imperiled, but these results showed that including AOO more accurately matched published NatureServe S‐ranks. However, it is possible that these data could be skewed due to most published S‐ranks being completed for rare species, with few assessments done for common species (95% of published S‐ranks for TN were S1–S3; NatureServe, 2024). Consequently, the role of AOO in rank assessments remains complex; its inclusion may improve alignment with known rarity patterns, but further research is needed to determine whether this represents a true increase in accuracy or a result of biased reference data.

### Tennessee S‐ranks in relation to surrounding states

Descriptive statistics and shape measurements of the semi‐automated methods and five comparison states indicated that the methods based on the NatureServe rank calculator leaned towards Imperiled. FME, RARECAT, and ArcGIS (both including and excluding AOO) all had a mean S‐rank of less than 2.6, whereas the mean S‐ranks for Kentucky, North Carolina, Virginia, and West Virginia were all greater than 3.1 (Tables 2 and 3). The surrounding state mode and county percentage ranks were closer to surrounding states, with a mean of 3.65 and 3.25, respectively. Similarly, FME, RARECAT, and ArcGIS (both including and excluding AOO) had lower standard deviations than surrounding states, while the standard deviations for the surrounding state mode and county percentage methods were higher and more comparable to those of surrounding states (Tables 2 and 3). This indicated that the ranks generated from the methods based on the NatureServe rank calculator were less evenly spread out and were instead concentrated near the mean, signifying the presence of more S2 and S3 ranks.

The skewness values resulting from the methods based on the NatureServe rank calculator were slightly positive or negative but close to zero, indicating that most S‐ranks were clustered near Imperiled (lower S‐ranks), whereas the skewness values of Kentucky, North Carolina, Virginia, and West Virginia as well as the skewness values of the surrounding state mode and county percentage methods were more negative, indicating that most S‐ranks were clustered near Secure (higher S‐ranks).

### Skewness of the NatureServe rank calculator

Through analyzing the semi‐automated methods against human rankings and surrounding state ranks, it was found that the S‐ranks generated using methods based on the NatureServe rank calculator lean towards Imperiled. FME, RARECAT, and ArcGIS including AOO had zero species ranked S5; when AOO was excluded, the number increased to only ~3% of species ranked S5 (Table 1).

The NatureServe rank calculator appears flawed because it does not account for state size. When calculating an S‐rank for a species, the same point values are assigned to criteria based on designated ranges that are standardized. Therefore, if a state has a smaller area, it will automatically receive lower point values, and S‐ranks will skew towards Imperiled. For example, a range extent throughout the entire state of Rhode Island (~4000 km^2^) would receive 2.36 points, whereas a range extent throughout the entire state of California (~400,000 km^2^) would receive 4.71 points. Tennessee's area falls between these with an area of ~100,000 km^2^ and would receive 3.93 points for a range extent throughout the entire state (Faber‐Langendoen et al., 2012). Despite the species being present across the entirety of all three states, the calculated S‐rank would be lower for a smaller state (Rhode Island), higher for a larger state (California), and moderate for an average‐sized state (Tennessee). This could potentially explain why the most frequent S‐rank calculated for TN in this study using the NatureServe rank calculator methods was S3. For this same reason, ecoregion‐specific species will automatically receive a low S‐rank. A species that is present throughout the entirety of the Blue Ridge Mountains ecoregion in TN (~6700 km^2^) would receive 3.14 points for range extent. This shows that despite occupying the entirety of that ecoregion in a state, the species would receive a low S‐rank. This potential flaw in the rank calculator is most evident when discussing range, but the same issue arises with number of occurrences and AOO. A state or ecoregion of smaller size will have fewer occurrence points and a smaller AOO, even if a species is spread throughout the state/ecoregion.

The NatureServe rank calculator tends to generate S‐ranks towards Imperiled, which may reflect its sensitivity to rarity or underdocumentation rather than indicate the actual extinction risk of the species. Similar patterns have been observed in other studies employing automated assessments, which often identify a high number of species potentially at risk of extinction (Bland et al., 2014; Stévart et al., 2019; Zizka et al., 2019). In contrast, alternative methods used in this study (particularly the county percentage method) produced a more balanced distribution of S‐ranks, highlighting the potential overestimation of rarity by strict calculator applications. While this may limit the accuracy of calculator methods in assigning definitive conservation status ranks, it highlights their utility in efficiently flagging potentially threatened species. In this way, automated approaches may serve as effective prioritization tools by narrowing large candidate pools to a subset warranting expert review. Given the constraints of limited funding and personnel, these tools can help streamline the process and improve the efficiency of species assessment workflows, allowing scientists to more easily recognize species that are truly at risk of extinction and in need of conservation prioritization.

### Evaluation of ranking methods

While assessing the accuracy of ranking methods was crucial, it was equally important to evaluate their practicality for future use. If a semi‐automated ranking method requires specialized expertise or is not easily accessible, it may not be feasible for use in ongoing ranking efforts. Therefore, it is essential to identify user‐friendly, available, and accurate ranking methods.

While the surrounding state mode ranking method was easy to understand and could be done using publicly accessible data, it could not be applied to all species due to a lack of data and its results showed poor agreement with published NatureServe S‐ranks. The county percentage ranking method could also be done using publicly available data and required minimal data analysis. Although this method was independent of the NatureServe rank calculator, it provided a reliable estimate of S‐rank with minimal knowledge and work. FME was not readily available due to high licensing costs and the requirement of advanced expertise to operate. This method produced results that were in moderate agreement with NatureServe S‐ranks, but its limited accessibility may prohibit the software from being used by a wide range of people, making it less suitable for mass‐ranking efforts. The RARECAT web app was relatively easy to use and produced results with the highest agreement to NatureServe S‐ranks. However, at the time of this study, it was limited to species‐by‐species analyses, making it favorable for single species assessments but time consuming for large‐scale assessments. In May 2025, after the completion of this study, NatureServe released a multi‐species version of RARECAT, potentially increasing its feasibility for large‐scale ranking efforts in the future. ArcGIS Pro produced results with the second highest agreement to NatureServe S‐ranks (along with FME), but offered several advantages over FME. It had lower licensing costs than FME, was more readily available through existing institutional licenses at state agencies and universities, and benefited from Esri's discounted pricing for nonprofit organizations. Although some technical expertise was required, extensive publicly available tutorials and resources improved user accessibility. Moreover, ArcGIS Pro supported batch processing for multiple species, enabling greater efficiency in large‐scale assessments, and allowed greater ability to inspect and customize data, broadening applicability to other taxonomic groups and geographic regions. Given these considerations, ArcGIS Pro is recommended for future ranking efforts primarily due to its scalability, accessibility, and integration into existing institutional workflows.

### Limitations

One limitation of this study was the use of occurrence records, which sometimes fail to represent the true status of a species due to data availability, taxonomic challenges, overcollection/undercollection, and data accuracy. Occurrence records were inherently biased as data were only available for areas that had been surveyed, most commonly public lands. Information for private lands and remote, inaccessible areas was often lacking and thus was not included in ranking calculations (Pearman et al., 2006). Additionally, there were limitations in using both citizen science observations and herbarium records. Species that were difficult for iNaturalist's image recognition technology to identify (e.g., grasses, sedges, asters) had fewer occurrence records than those that iNaturalist could easily assign a scientific name. Therefore, troublesome taxa had fewer occurrence records, not necessarily because these species were rare, but because their identification required expertise that was not as readily available. On the other hand, species that were easy to identify, as well as visually charismatic species, were sometimes overdocumented. A limitation for herbarium records was that only digitized, georeferenced specimens could be utilized. Some of the same biases mentioned above were also true for herbarium records, with some taxa being over‐ or underdocumented. Finally, cultivated populations may have been present in both citizen science and herbarium records, which could have skewed calculations as these records did not accurately reflect a species’ natural presence.

### Future ranking efforts

The status of S‐ranks throughout the United States is largely incomplete, with an average of 57% of each state's flora assessed (Figure 1), resulting in severe gaps in our understanding of the subnational extinction risk for plant species and yielding less‐informed conservation decisions. An instruction manual and Toolbox were created to streamline the use of ArcGIS Pro to facilitate assigning S‐ranks in other states or updating previously assigned ones. This Toolbox allows the user to upload a layer of occurrence points and automatically calculate range extent, AOO, and number of occurrences for each species. The manual provides instructions on how to use the Toolbox and how to convert rarity criteria for use in NatureServe's rank calculator (Appendix S1). A separate manual outlines how to calculate criteria without the Toolbox, instead using a series of geoprocessing tools, which makes the process longer but allows more customization (Appendix S3). Both manuals include the specific parameters required for the tools to function properly as well as pictures to aid in understanding to ensure that these methods can be used by those with little to no GIS experience. The Toolbox and manuals are publicly available on GitHub (https://github.com/juliaprins/S-rank-ArcGIS-Manual), ensuring broad accessibility. The ability to batch process in ArcGIS Pro allows users to rank multiple species simultaneously, streamlining the process and significantly reducing the time required compared to a species‐by‐species approach. This increased efficiency enables the ranking of more species in less time, providing an even greater benefit for conservation and overcoming many resource limitations.

The manuals and methods employed in this study were originally developed to rank vascular plant species but are broadly applicable to other taxonomic groups and geographic regions. Using the same approach, taxa such as mammals, insects, fungi, fish, and non‐vascular plants can also be assessed, provided sufficient occurrence data are available. Although this study focused on subnational rankings, the methodology is also applicable at the national or global level, as long as appropriate geographic boundaries are defined. The NatureServe rank calculator uses standardized point values across all geographic levels (subnational, national, and global), facilitating the transferability of methodology. While NatureServe is currently a North American ranking system, the same methods can be applied internationally to evaluate species rarity and inform conservation priorities in other countries. Scientists can use these methods to easily analyze how species populations have changed over time by ranking historical data and then current data, providing insight into species abundance and identifying trends in population dynamics.

The methods discussed in this study should be used for the purpose of providing provisionary S‐ranks that experts can use as a basis for ranking species. They are not meant to remove expert opinion and replace traditional assessments but rather to accelerate and enhance the ranking process, allowing for more species to be assessed with fewer resources and in less time. This standardized ranking process will increase consistency of ranks and eliminate the bias inherent in human beliefs. Future work is needed by experts in reviewing provisionary S‐ranks to ensure accuracy and account for factors that automated methods could not recognize. To minimize variability present when relying on personal beliefs, it is essential that expert review be conducted systematically. One approach is to translate qualitative expert opinions into a standardized quantitative framework (such as through structured surveys using Likert scales) and then integrate the anonymous input of a broad panel of professionals. This method can reduce bias, enhance consistency, and better reflect collective expert judgement. Once reviewed, these S‐ranks can be utilized to make better conservation decisions that more adequately protect state floras.

## AUTHOR CONTRIBUTIONS

J.H.P. designed the work, completed the dataset building, implemented the methodology, and performed data analysis. J.S. provided the conceptual framework, expertise on the flora, and technical review. J.H.P. wrote the original draft of the manuscript, and all authors approved the final version.

## Supporting information


**Appendix S1.** Manual for calculating rarity criteria and provisionary S‐ranks using ArcGIS Pro and a Toolbox.

**Appendix S2.** List of 2404 native Tennessee species ranked using the semi‐automated methods, S‐ranks from each method, and calculated criteria used to assign ranks.

**Appendix S3.** Manual for calculating rarity criteria and provisionary S‐ranks using a series of geoprocessing tools in ArcGIS Pro (without a Toolbox).

## Data Availability

The ArcGIS Pro Toolbox and manuals are available at https://github.com/juliaprins/S-rank-ArcGIS-Manual along with the CSV file of the GBIF and SERNEC occurrence records used for the ranking methods based on the NatureServe rank calculator. Data for sensitive species can be obtained by contacting the Tennessee Department of Environment and Conservation Division of Natural Areas (TDEC‐DNA).
