# Supplementary material for: Semi‐automated assessment for NatureServe subnational conservation status ranks for state floras
Source: Appl Plant Sci. 2026 Mar 19;14(2):e70046. doi: 10.1002/aps3.70046 (PMC13103536; doi:10.1002/aps3.70046)
Supplement: Supplementary file 1 — Appendix S1. Manual for calculating rarity criteria and provisionary S‐ranks using ArcGIS Pro and a Toolbox. [file APS3-14-e70046-s002.docx]

Appendix S1. Manual for calculating rarity criteria and provisionary S-ranks using ArcGIS Pro and a Toolbox.

Using ArcGIS Pro to Calculate Rarity Criteria for NatureServe Conservation Status Assessments with a Toolbox

By: Julia Prins

The University of Tennessee at Chattanooga

May 2025

This document describes the steps to be taken when using ArcGIS Pro to calculate rarity criteria for NatureServe conservation status assessments for species. More specifically, it describes how to gather species occurrence data, clip data to a boundary, and use a Toolbox to calculate range extent, number of occurrences, and area of occupancy (Toolbox available at https://github.com/juliaprins/S-rank-ArcGIS-Manual). This information can then be used directly to assign conservation status ranks using the NatureServe rank calculator (available at natureserve.org; NatureServe, 2020b[)](https://www.natureserve.org/conservation-tools/conservation-rank-calculator). These processes can be used to calculate criteria for an individual species or as a batch process to calculate criteria for multiple species simultaneously. Additionally, the processes can be applied at all geographic scales (global, national, and subnational) provided that the appropriate boundary is selected. Another version of this manual that does not use a Toolbox and calculates the rarity criteria individually through a series of steps can be found at https://github.com/juliaprins/S-rank-ArcGIS-Manual.

To follow these instructions, a basic understanding of ArcGIS software is required. Developed in 2025, these guidelines use Esri ArcGIS Pro Version 3.0.0. Please note that as applications are continuously evolving, the applicability of these instructions may change slightly over time. Questions and further information related to the instructions below can be directed to Julia Prins (juliaprins19@gmail.com).

I would like to thank Dillon Blankenship (Tennessee Natural Heritage Program) and Nyssa Hunt (The University of Tennessee at Chattanooga - Interdisciplinary Geospatial Technology Lab) for their GIS expertise and assistance with this manual.

**Gathering Species Occurrence Data**

Species occurrence records containing geographic coordinates are necessary to calculate the rarity criteria of range extent, number of occurrences, and area of occupancy. These records can be obtained from internal sources such as Biotics or external sources such as GBIF and the Symbiota portals.

Obtaining occurrences records from GBIF (GBIF.org, 2024):

1. Visit gbif.org and select occurrences.
2. Select the All filters tab.
3. Under the Taxon category, apply a filter to only include records of the taxon of interest.
4. Under the Location category apply a filter to only include records from your area of interest by selecting the desired Country or area and State province. Under location select including coordinates.
5. Under the Event category apply a filter to exclude historic records using the desired cutoff year.
6. In the display window select Download and choose either the Simple or Darwin Core download option.

Obtaining occurrences records from a Symbiota portal (SERNEC Data Portal, 2024):

Note: the instructions below are for the Symbiota portal of the Southeast Regional Network of Expertise and Collections (SERNEC) but similar steps can be taken to obtain occurrence records from the other Symbiota portals (available at symbiota.org).

1. Visit https://sernecportal.org/portal/
2. Under the Specimen Search tab select Search Collections.
3. Under the Taxon category, apply a filter to only include records of the taxon of interest.
4. Under the Locality category, enter the Country or State of interest.
5. Under the Sample Properties category, check the Limit to Specimens with Geocoordinates box.
6. Under the Collecting Event category, enter the desired historic cutoff date.
7. Under Results Display Format, select Table and hit Search.
8. Click the Download Specimen Data button at the top of the page.
9. Select the settings of your choice and hit Download Data.

Prepping Occurrence Data for Upload:

If gathering occurrence data from multiple sources, all occurrences need to be merged into a single spreadsheet and several steps need to be taken to ensure information matches.

1. Ensure the same scientific names are being used across datasets. If any synonyms are being used in a dataset replace the synonym names with the correct scientific name. This can be done by obtaining a synonym list and cross-checking it against the dataset.
2. Ensure the same coordinate system is being used across datasets. Coordinates should be in decimal degrees of latitude and longitude, with latitudes south of the equator represented by negative values and longitudes west of the Prime Meridian also indicated by negative values.
3. Next, the datasets can be combined into a single spreadsheet. The first column should contain scientific names and in the first cell should be the column title “Scientific_name” (ensure the title follows this exact spelling and capitalization to ensure the Toolbox functions properly). The second column should contain latitude points and be titled “Latitude”. The third column should contain longitude points and be titled “Longitude”.
4. If ranking at the species level all infraspecific designations should be removed and simplified to species level (e.g. *Asplenium scolopendrium var. americanum* simplified to *Asplenium scolopendrium*). This ensures that all occurrences for a species are being accounted for in its calculations.
5. Lastly, ensure no spaces exist in column names or scientific names. Any spaces should be replaced with an underscore (“_”) using the find and replace tool. The same should be done for any special characters (e.g. the genus *Isoëtes* should be replaced with *Isoetes* so the special character is replaced). This is because ArcGIS Pro can have difficulty reading file names that contain spaces or special characters.

**Clipping the Data to Area of Interest**

When using occurrence records from external sources, such as GBIF and Symbiota portals, it is crucial to verify that all records fall within the area of interest when mapped. Even if data is filtered during the initial collection, inaccuracies in locality information can lead to records being mapped outside of the intended area. Therefore, to ensure the accuracy of data, it is important to clip the occurrence records to the boundary of the area of interest as described below.

Step 1: Upload a boundary file.

If you already have a layer defining the boundary of your area of interest add this to your map by navigating to the ribbon at the top of the page and under the Map tab in the Layer group, select Add Data. Select the boundary layer and hit OK.


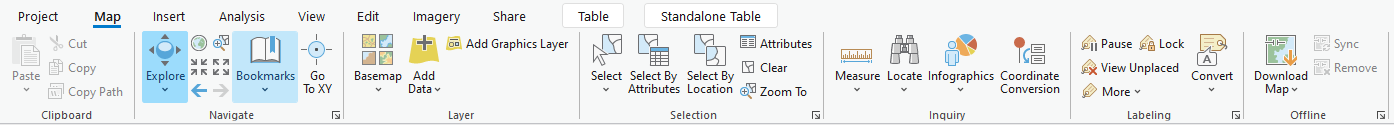


If you do not have a layer defining the boundary of your area of interest, you can use a pre-existing layer from the ArcGIS Online Living Atlas (option 1 below) or define your boundary (option 2 below).

*Option 1: Using a layer from the ArcGIS Online Living Atlas for U.S. States or Canadian Provinces/Territories*

1. In the Catalog pane select the Portal tab. Select Living Atlas.


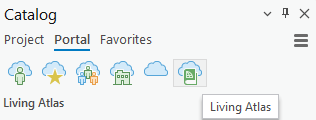


1. Search “USA State Boundaries” or “Provinces and Territories of Canada”. Multiple layers containing the boundaries of your search will appear. Right-click the feature layer of your choice and select Add to Current Map.
2. To only include the boundary of your state or province/territory of interest a definition query can be applied to the layer. In the Contents pane right-click the boundary layer and select Properties. In the Properties window select the Definition Query tab. Select New definition query and add a query where state or province/territory name is equal to state or province/territory of interest. Select Apply and hit OK.


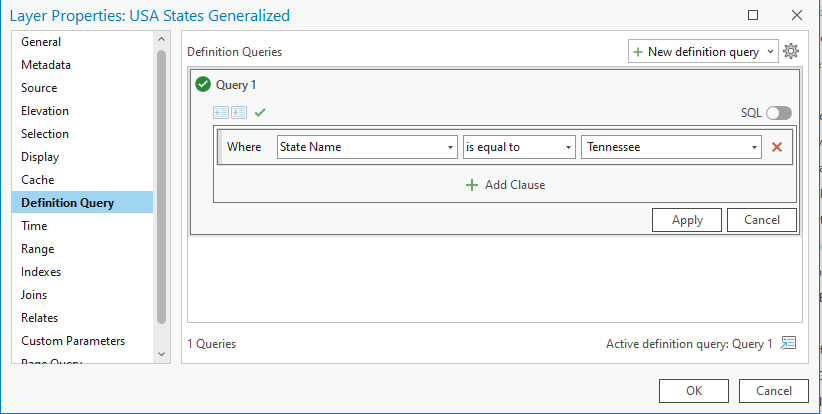


1. In the Contents pane right-click the boundary layer and select Data and Export Features. Name the Output Feature Class as the state or province/territory boundary and select OK. This will add the living atlas layer to the local geodatabase so it can be used for geoprocessing tools.


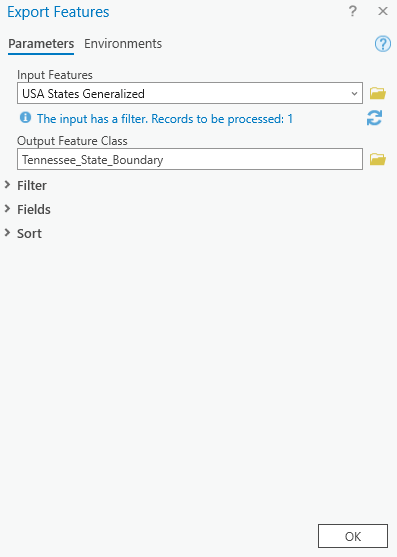


*Option 2: Manually Defining a Boundary*

1. In the Catalog pane, under the Project tab expand Databases. Right-click the current project geodatabase and select New and Feature Class.


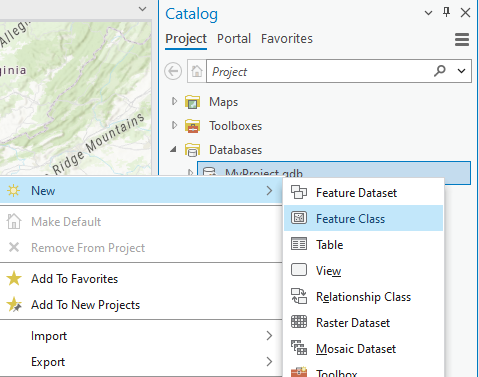


1. Name the Feature Class “AreaBoundary” and ensure Polygon is selected as the Feature Class Type. Select Finish.


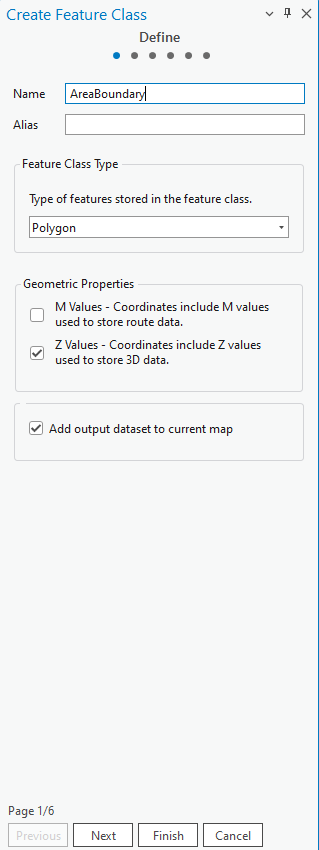


1. Navigate to the ribbon at the top of the page. Under the Edit tab in the Features group select Create.


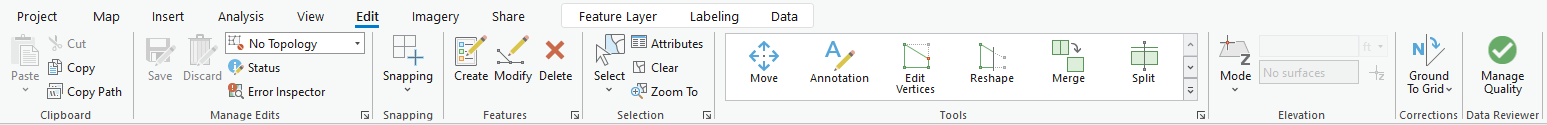


1. In the Create Features pane select the new AreaBoundary layer and use the Polygon tool to draw a boundary for your area of interest on the map. Double-click to finish the polygon.


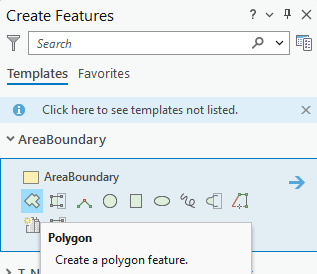


1. Navigate to the ribbon at the top of the page. Under the Edit tab in the Manage Edits group select Save.

Step 2: Upload your occurrence records.

1. Ensure your spreadsheet is downloaded as a CSV file.
2. Navigate to the ribbon at the top of the page. Under the Map tab in the Layer group select Add Data.


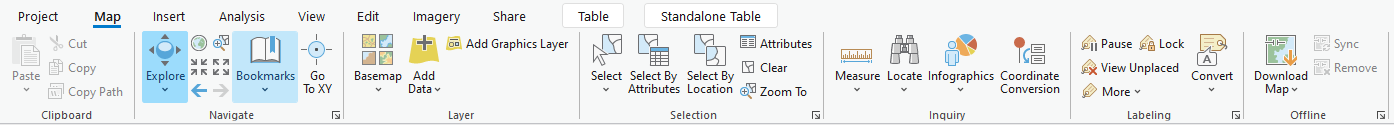


1. Select the file containing your occurrence records and hit OK.
2. In the Contents pane right-click the newly added attribute table containing your occurrence records and select Display XY Data (Note: newer versions of ArcGIS Pro may require you to select Create Points From Table and then XY Table to Point instead).
   1. Ensure your occurrence file is selected as the Input Table.
   2. Name your Output Feature Class as “Occurrence_Records_ALL”.
   3. For the X Field ensure the column containing longitude is selected.
   4. For the Y Field ensure the column containing latitude is selected.
   5. Hit OK.
   6. This will add the occurrence points to your map.


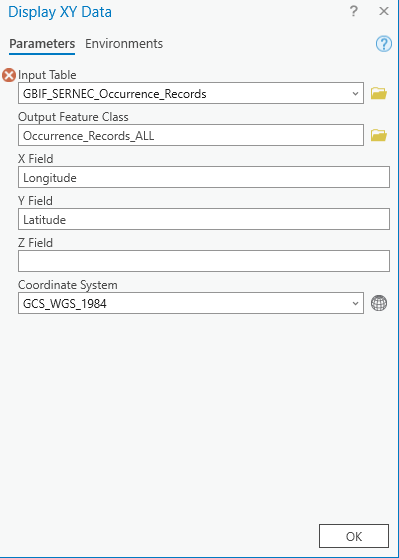


Step 3: Ensure the coordinate system of your dataset is UTM (Universal Transverse Mercator)

1. To check the coordinate system of your dataset, in the Contents pane right-click the layer containing all occurrence records and select Properties. In the Properties window select the Source tab. Expand Spatial Reference and the Geographic Coordinate System will be listed. If the coordinate system is not UTM the occurrence record layer will need to be Projected into a UTM coordinate system using the following steps (Note: the image below shows a coordinate system *not* in UTM).


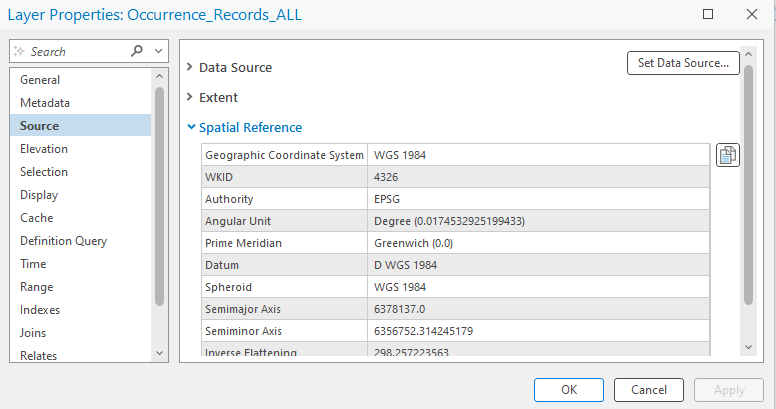


1. Open the geoprocessing pane by navigating to the ribbon at the top of the page. Under the Analysis tab in the Geoprocessing group select Tools.


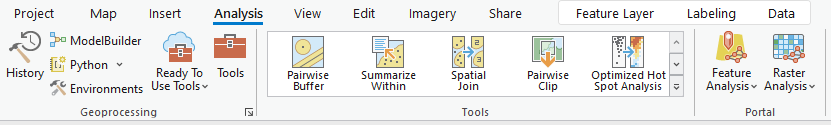


1. In the geoprocessing pane search for and open the *Project* tool.
2. For Input Dataset or Feature Class select the layer containing all occurrence records.
3. Name the Output Dataset or Feature Class “Occurrence_Records_UTM”.
4. Select a UTM Output Coordinate System.
   1. Click the globe icon to select a coordinate system.


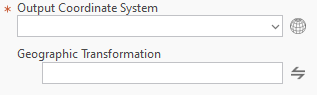


- 1. In the Output Coordinate System window under XY Coordinate Systems Available expand Projected Coordinate System.


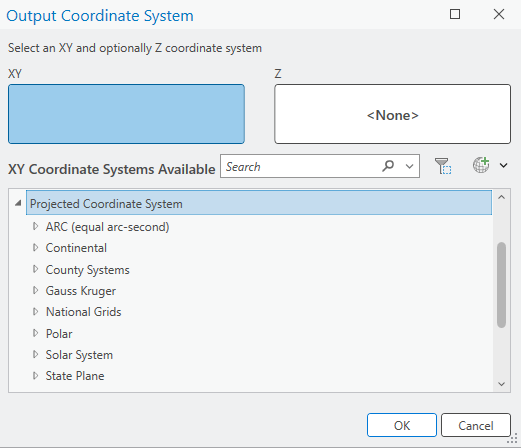


- 1. Scroll down and expand UTM.


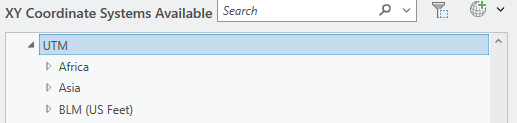


- 1. Scroll down and expand WGS 1984 and expand Northern Hemisphere.


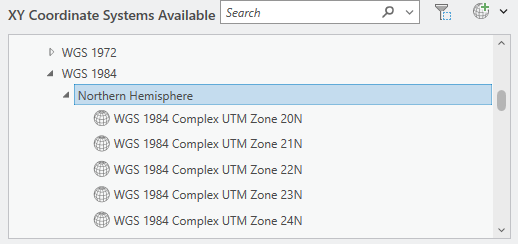


- 1. Find and select the UTM zone located at the center of your area of interest (this information is readily available on the internet).


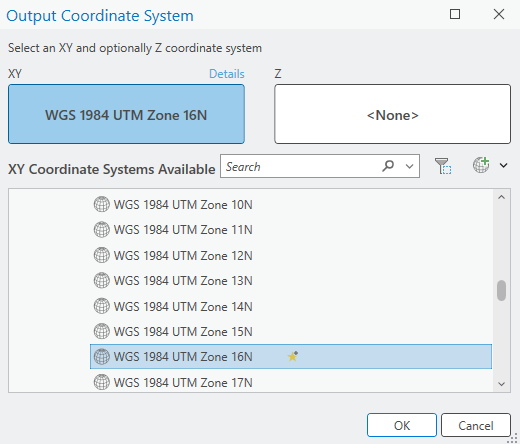


- 1. Click OK.


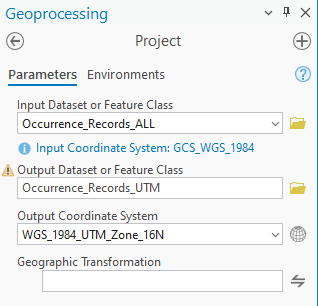


1. Click Run.

Step 4: Use the *Clip Layer* tool to remove all occurrence points outside of the area of interest.

1. In the geoprocessing pane search for and open the *Clip Layer* tool.
   1. For Input Layer select the layer containing all occurrence records in the UTM coordinate system created in step 3.
   2. For Clip Layer select the layer containing the boundary of your area of interest.
   3. Name the Output Feature Class “Occurrence_Records_Clipped”.
   4. Click Run.


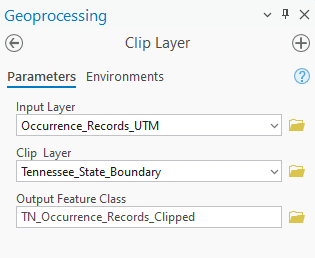


**Using the Toolbox to Calculate Range Extent, Number of Occurrences, and Area of Occupancy**

The Toolbox (available at https://github.com/juliaprins/S-rank-ArcGIS-Manual) streamlines the process of calculating range extent, area of occupancy, and number of occurrences. A file containing your occurrence points is entered and the Toolbox will use the uploaded records to create individual occurrence layers for each species and from those layers calculate range extent, area of occupancy, and number of occurrences. Range extent is calculated in square meters, area of occupancy is calculated based on the number of occupied 2 x 2 km grid cells, and number of occurrences is calculated using a 1 km separation distance (NatureServe, 2020a).

Step 1: Use the *Generate Tessellation* tool to create a layer of 2 x 2 km grid cells (this layer will be needed by the Toolbox to calculate area of occupancy).

1. Open the geoprocessing pane by navigating to the ribbon at the top of the page. Under the Analysis tab in the Geoprocessing group select Tools.


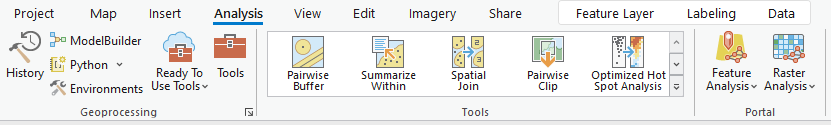


1. In the geoprocessing pane search for and open the *Generate Tessellation* tool.
2. Name the Output Feature Class “AOO_Grid_Cells”.
3. For Extent click the dropdown and select the layer containing the boundary for your area of interest (this was created during step 1 of the Clipping the Data to Area of Interest section of the manual). Extent will auto populate based on this layer.
4. For Shape Type select Square.
5. For Size enter 4 and select Square Kilometers as the unit.
6. Click Run.


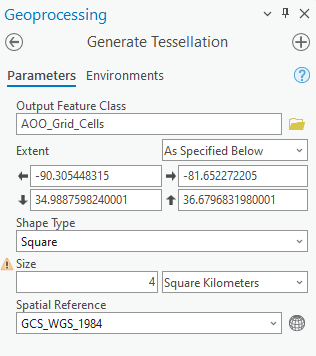


Step 2: Download the Toolbox from GitHub and add it to your map

1. Visit the “S-rank-ArcGIS-Manual” repository on GitHub (Available at: https://github.com/juliaprins/S-rank-ArcGIS-Manual). Click on “Rarity Criteria Toolbox.atbx”


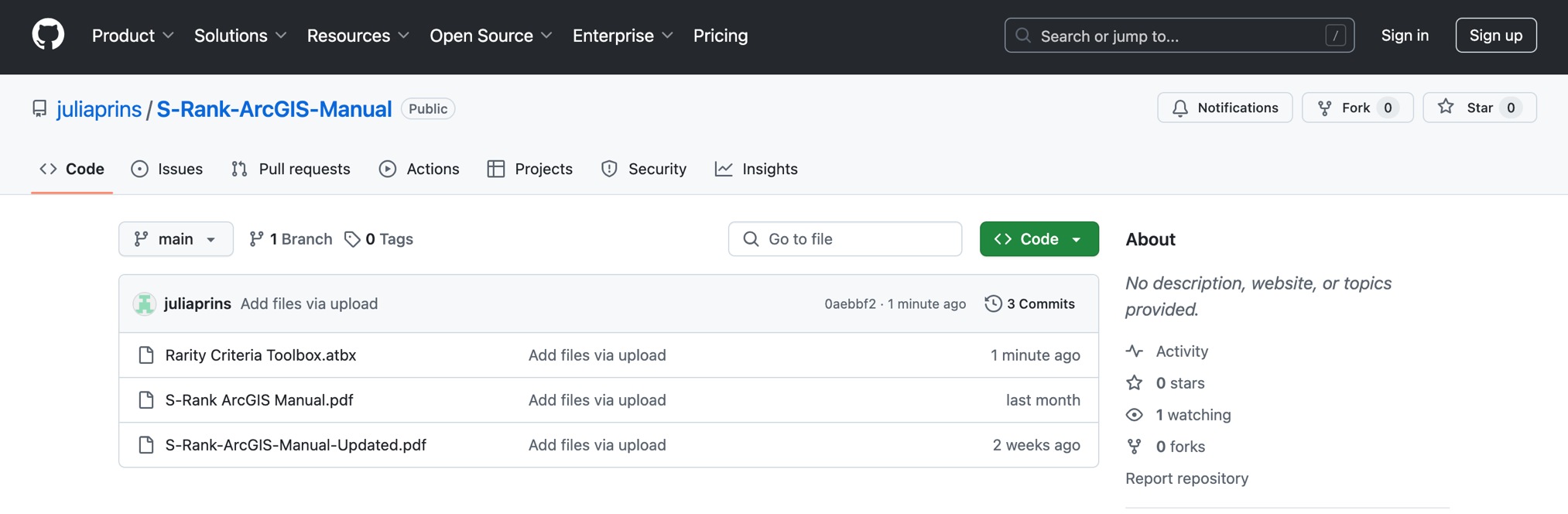


1. Download the Toolbox by clicking on the Download raw file button. Save the Toolbox to your desired location.


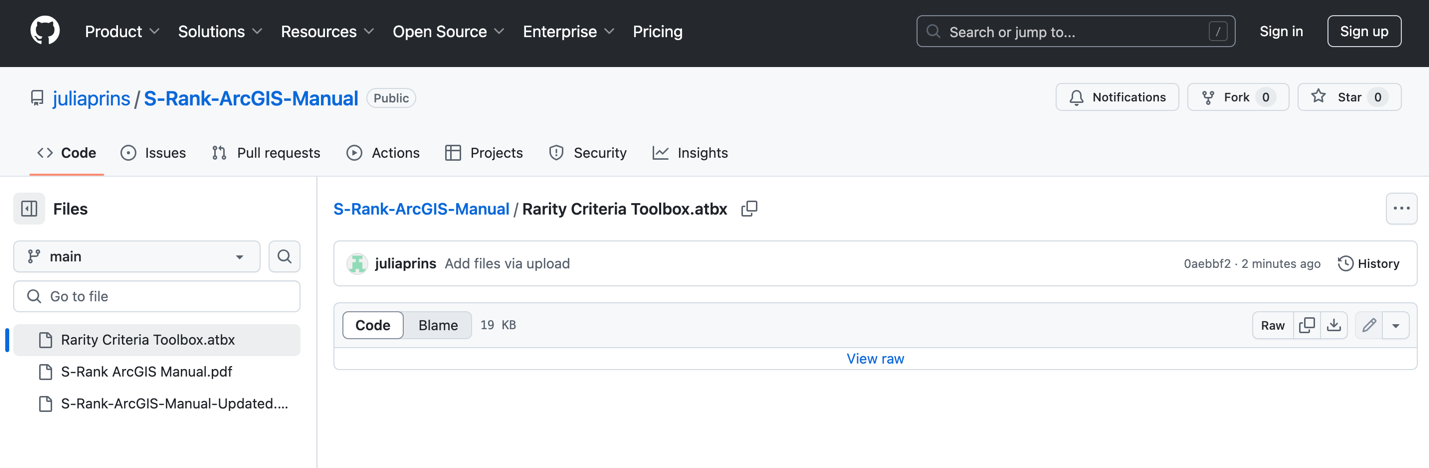


1. In ArcGIS Pro, navigate to the ribbon at the top of the page. Under the Insert tab in the Project group select Toolbox and Add Toolbox. Add the downloaded Toolbox from the location it was saved to in the previous step.


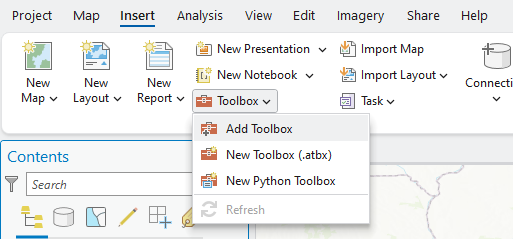


Step 3: Calculate range, area of occupancy, and number of occurrence using the Toolbox

1. In the Catalog pane, under the Project tab expand Toolboxes and expand the Rarity Criteria Toolbox. Double-click the “Occurrence Layer to Range, AOO, and EO” model to open it.


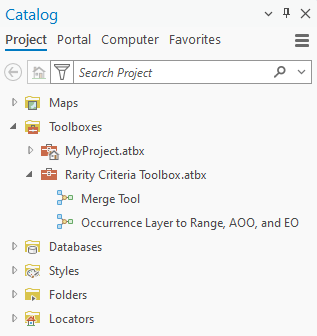


1. For Input_Occurrence_Data select the clipped occurrence layer created in the Clipping the Data to Area of Interest section of the manual.
2. For AOO_Grid_Cells select the 4 km^2^ grid cells created in Step 1 of this section.
3. For Output_Species_Occurrence_Layers open the folder and select the current project geodatabase as your save location and name the file “Occurrences_%Value%”. Ensure the exact spelling and capitalization is used in order for the model to run properly.
   1. The “%Value%” command tells the model to pull in the name from the “Scientific_name” column when creating each unique layer. This ensures that each layer will contain its scientific name (e.g. “Occurrences_Acer_rubrum”, “Occurrence_Abies_fraseri”, etc.).
4. For Output_Range_Layers open the folder and select the current project geodatabase as your save location and name the file “Range_%Value%”. Ensure the exact spelling and capitalization is used.
5. For Output_EO_Layers open the folder and select the current project geodatabase as your save location and name the file “EO_Count_%Value%”. Ensure the exact spelling and capitalization is used.
6. For Output_AOO_Layers open the folder and select the current project geodatabase as your save location and name the file “AOO_Count_%Value%”. Ensure the exact spelling and capitalization is used.


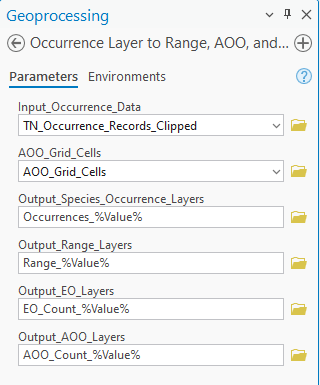


1. Click Run. Depending on how many occurrences records are in your file and the processing speed of your device this may take several hours

Step 4: Use the Merge Tool in the Toolbox to create a single layer for all range, AOO, and EO values

1. In the Catalog pane, under the Project tab expand Toolboxes and expand the Rarity Criteria Toolbox. Double-click the “Merge Tool” model to open it.


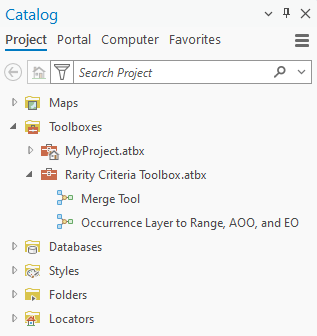


1. For All_Range_Extent_Layers open the folder and select all range layers that were created in step 3 and saved to your current maps geodatabase. Click OK.


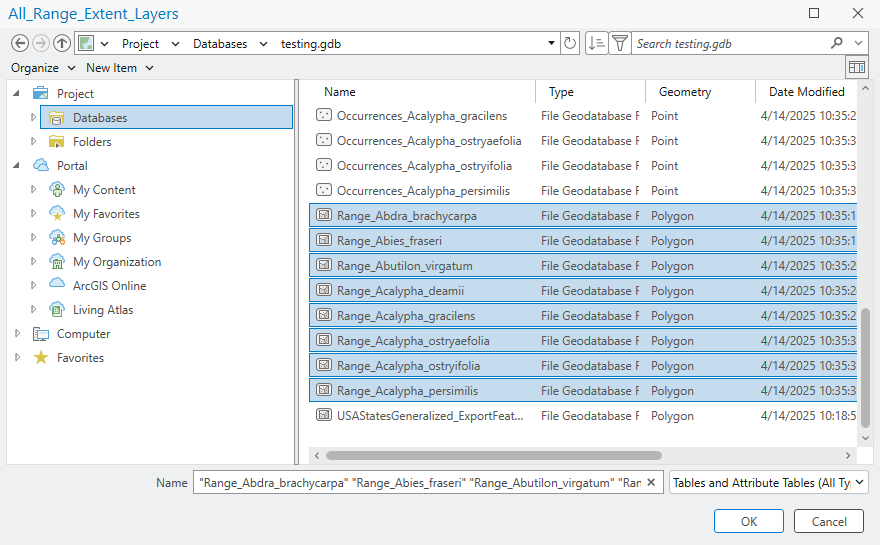


1. For All_AOO _Layers open the folder and select all AOO layers that were created in step 3 and saved to your current maps geodatabase.
2. For All_EO _Layers open the folder and select all EO layers that were created in step 3 and saved to your current maps geodatabase.
3. For Species_Range_Extent name the file. This will contain range values for all species.
4. For Species_AOO name the file. This will contain area of occupancy values for all species.
5. For Species_EO_Counts name the file. This will contain number of occurrences values for all species.
6. Click Run.


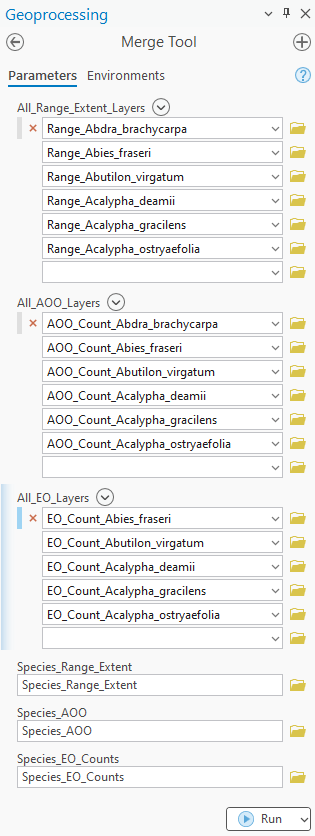


**Using the NatureServe Rank Calculator to Generate a Rank**

The calculated rarity criteria can be individually, or batch uploaded to the NatureServe rank calculator to generate a rank. This calculator includes 10 criteria which fall under three categories: rarity, trends, and threats. A minimum of two criteria are needed when assigning ranks using the calculator. Criteria are entered and an S-rank is automatically assigned. The user cannot adjust any weightings or point values (NatureServe, 2020b; Faber-Langendoen et al., 2012).

Step 1: Export range extent, number of occurrences, and area of occupancy merge tables from ArcGIS Pro to Microsoft Excel

1. In ArcGIS Pro open the geoprocessing pane by navigating to the ribbon at the top of the page. Under the Analysis tab in the Geoprocessing group select Tools.


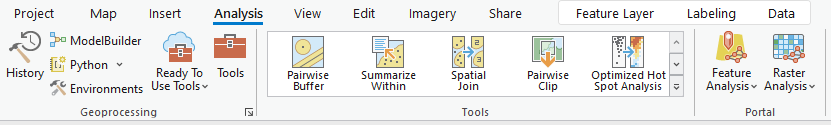


1. In the geoprocessing pane search for and open the *Table To Excel* tool.
2. Next to Input Table click the down arrow and select the three merge tables created for area of occupancy, number of occurrences, and range extent. Each table should contain the calculated criteria for all species. Click Add.
3. Name your Output Excel File and select where you want the file to be saved.
4. Click Run.


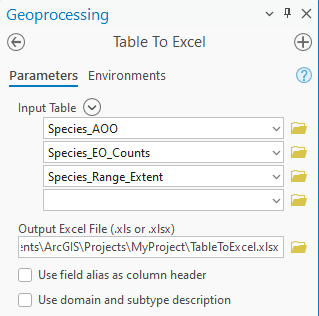


1. Open the newly created Excel file. The file should contain a separate sheet for each of the criteria (range, EO, and AOO).


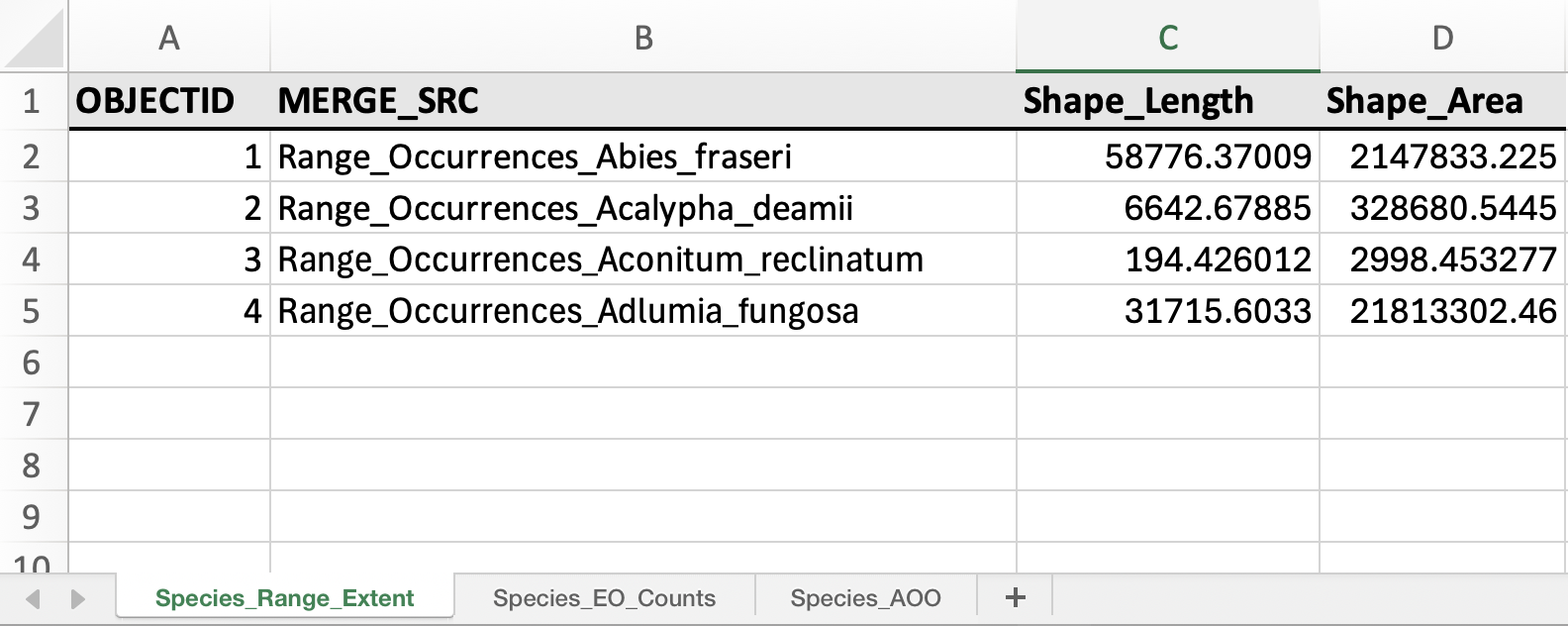


Step 2: Convert calculated values into rating codes

Values must be converted into the appropriate rating codes used by the NatureServe rank calculator based on where the values fall in designated ranges (Table 1). This can easily be done using Microsoft Excel (or equivalent).

1. Convert exported range extent values from ArcGIS to rating codes based on ranges found in Table 1.
   1. Range extent values first need to be converted from square meters to square kilometers by dividing values by 1,000,000. This can be done in Excel by creating a new column adjacent to the column containing range extent values and in the first cell entering the formula =D2/1000000 (assuming your range extent values are in column D and start in the second row). Pull the formula down by selecting the cell that contains the formula and clicking on the small square that appears in the bottom-right corner of the selected cell (this is called the fill handle). Drag the fill handle down until the formula is copied to all rows. Excel will automatically adjust the cell number in the formula to match the adjacent cell number.


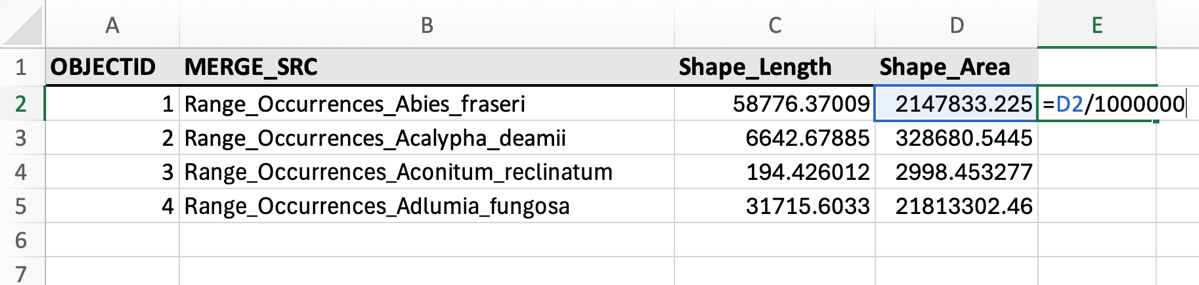


- 1. To convert the range extent values now in km^2^ to the appropriate rating code the IF formula can be used. This formula will return a value based on a specified condition. The ranges from Table 1 and their associated rating codes can be entered into the formula as the condition and the formula will return the appropriate rating code based on the range extent value in a specified cell. The following formula applies the ranges and their associated rating codes for range extent from Table 1 as the condition. This formula can be entered into the first cell adjacent to the column containing range extent in km^2^ and pulled down to all rows using the fill handle.

=IF(AND(E2>=0,E2<=99),"A",IF(AND(E2>=100,E2<=249),"B",IF(AND(E2>=250,E2<=999),"C",IF(AND(E2>=1000,E2<=4999),"D",IF(AND(E2>=5000,E2<=19999),"E",IF(AND(E2>=20000,E2<=199999),"F",IF(AND(E2>=200000,E2<=2499999),"G",IF(AND(E2>=2500000),"H","N/A"))))))))

This formula assumes that your range extent values in km^2^ are in column E and start in the second row.


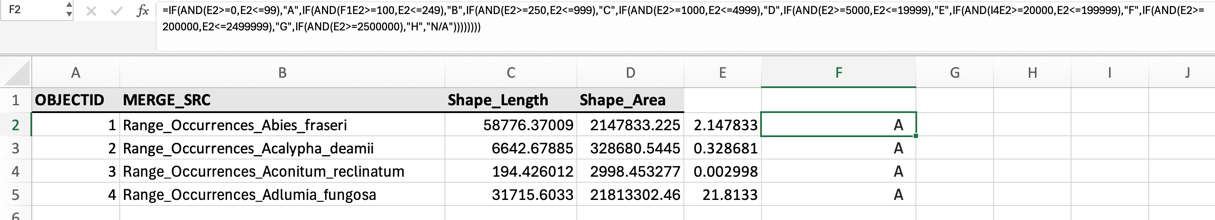


1. Convert exported number of occurrences values from ArcGIS to rating codes based on ranges found in Table 1.
   1. To convert the number of occurrences values to the appropriate rating code the IF formula can again be used. The following formula applies the ranges and their associated rating codes for number of occurrences from Table 1 as the condition. This formula can be entered into the first empty cell adjacent to the occupied columns and pulled down to all rows using the fill handle.

=IF(AND(B2>=1,B2<=5),"A",IF(AND(B2>=6,B2<=20),"B",IF(AND(B2>=21,B2<=80),"C",IF(AND(B2>=81,B2<=300),"D",IF(AND(B2>=301),"E","N/A")))))

This formula assumes that your number of occurrences values are in column B and start in the second row.


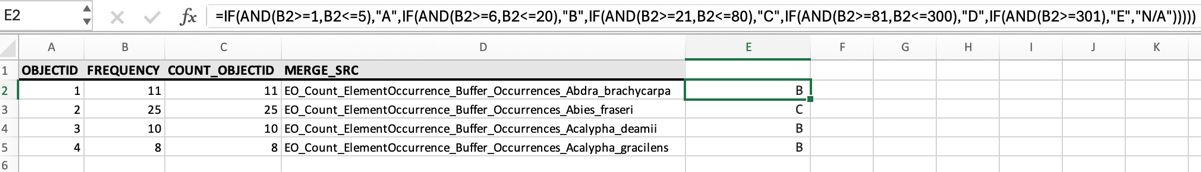


1. Convert exported area of occupancy values from ArcGIS to rating codes based on ranges found in Table 1.
   1. To convert the area of occupancy values to the appropriate rating code the IF formula can again be used. The following formula applies the ranges and their associated rating codes for area of occupancy from Table 1 as the condition. This formula can be entered into the first empty cell adjacent to the occupied columns and pulled down to all rows using the fill handle.

=IF(AND(B2=1),"A",IF(AND(B2=2),"B",IF(AND(B2>=3,B2<=5),"C",IF(AND(B2>=6,B2<=25),"D",IF(AND(B2>=26,B2<=125),"E",IF(AND(B2>=126,B2<=500),"F",IF(AND(B2>=501,B2<=2500),"G",IF(AND(B2>=2501,B2<=12500),"H",IF(AND(B2>12500),"I","N/A")))))))))

This formula assumes that your area of occupancy values are in column B and start in the second row.


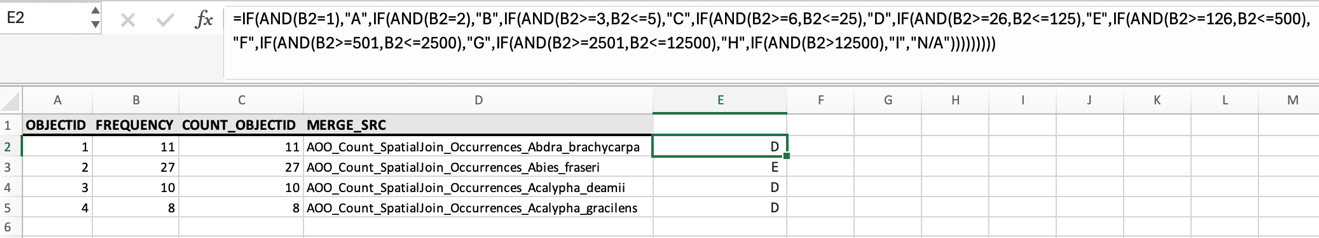


Table 1: Rating codes for rarity criteria (Master et al., 2012)

| Range Extent | Number of Occurrences | Area of Occupancy |
| --- | --- | --- |
| Z = 0 | Z = 0 | Z = 0 |
| A = <100 km^2^ | A = 1 – 5 | A = 1 |
| B = 100 – 250 km^2^ | B = 6 – 20 | B = 2 |
| C = 250 – 1,000 km^2^ | C = 21 – 80 | C = 3 – 5 |
| D = 1,000 – 5,000 km^2^ | D = 81 – 300 | D = 6 – 25 |
| E = 5,000 – 20,000 km^2^ | E = >300 | E = 26 – 125 |
| F = 20,000 – 200,000 km^2^ |  | F = 126 – 500 |
| G = 200,000 – 2,500,000 km^2^ |  | G = 501 – 2,500 |
| H > 2,500,000 km^2^ |  | H = 2,501 – 12,500 |
|  |  | I = >12,500 |

Step 3: Download the NatureServe rank calculator

1. Visit natureserve.org/products/conservation-rank-calculator and download the latest version of the rank calculator.
2. Enter your personal information and email. A download link will be sent to your email.
3. Click the download link and open the downloaded Microsoft Excel file (Note: you will need to enable macros for the file to work properly).
4. Select Enter

Step 4: Insert rating codes into NatureServe rank calculator (the following steps use Version 3.2)

1. Open the “Calculator Table” tab of the rank calculator Excel spreadsheet
2. Copy scientific names and paste them into Column C of the rank calculator titled “Species or Community Scientific Name”
3. Copy range extent rating codes and paste them into Column K of the rank calculator titled “Range Extent” (ensure the appropriate rating code is in the same row as its correct scientific name)
4. Copy area of occupancy rating codes and paste them into Column M of the rank calculator titled “Area of Occup 4-km^2^ grid cells” (ensure the appropriate rating code is in the same row as its correct scientific name)
5. Copy number of occurrences rating codes and paste them into Column O of the rank calculator titled “# Occur” (ensure the appropriate rating code is in the same row as its correct scientific name)
6. Ranks will auto-populate from the entered information in Column A titled “Calc Rank”


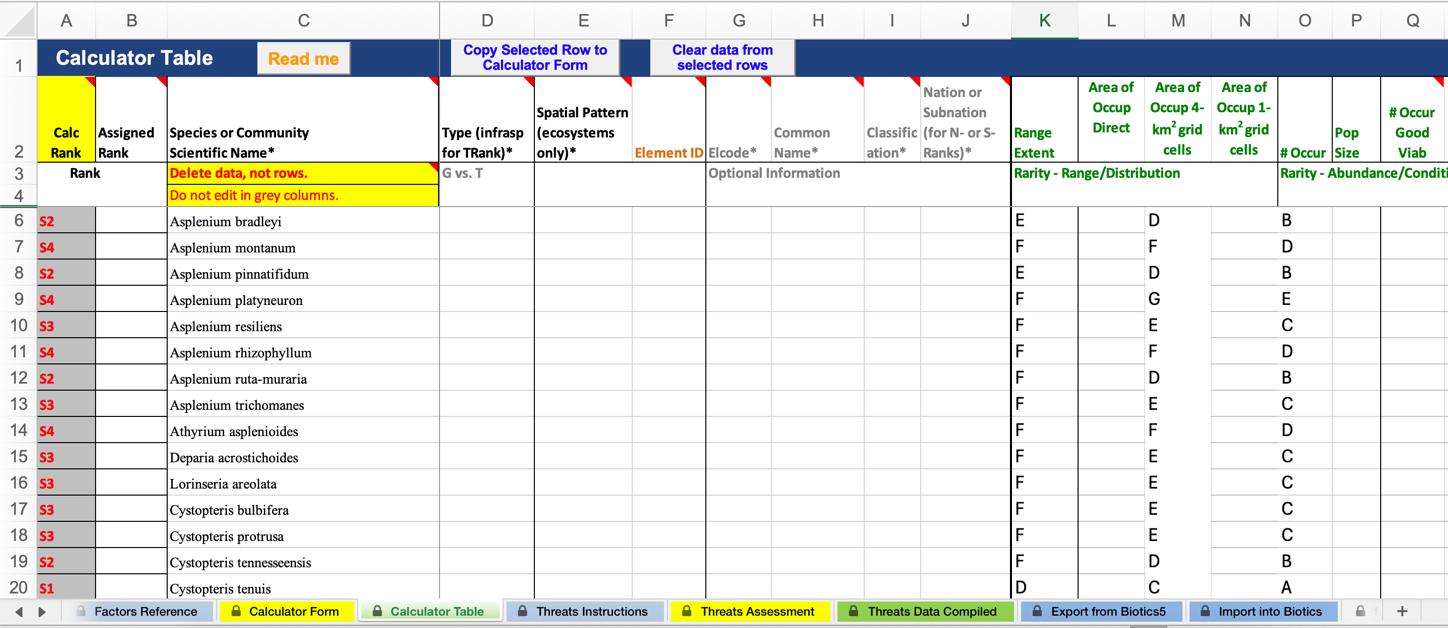


1. If available, information on threats, trends, or additional rarity criteria can be entered into the appropriate columns and the rank will automatically be adjusted. A breakdown of the rating codes for additional criteria can be found in the “Factors Reference” tab. The “Calculator Form” tab is useful for species-by-species assessments and allows for the selection of the appropriate rating code directly with the ranges for each code provided.

References

Faber-Langendoen, D., J. Nichols, L. Master, K. Snow, A. Tomaino, R. Bittman, G. Hammerson, et al. 2012. NatureServe Conservation Status Assessments: Methodology for Assigning Ranks. NatureServe, Arlington, Virginia, USA.

GBIF.org. 2024. Occurrence Search [online]. Website https://doi.org/10.15468/dl.7h2ehs [accessed 9 Jan 2024].

Master, L. L., D. Faber-Langendoen, R. Bittman, G. A. Hammerson, B. Heidal, L. Ramsay, K.

Snow, et al. 2012. NatureServe Conservation Status Assessments: Factors for Evaluating

Species and Ecosystem Risk. NatureServe, Arlington, Virginia, USA.

NatureServe. 2020a. Habitat-based Plant Element Occurrence Delimitation Guide. NatureServe, Arlington, Virginia, USA.

NatureServe. 2020b. NatureServe Conservation Status Assessments: Rank Calculator, Version

3.2. NatureServe, Arlington, Virginia, USA. Website

https://www.natureserve.org/conservation-tools/conservation-rank-calculator [accessed 9 Nov 2023].

SERNEC Data Portal. 2024. SERNEC Collections Search Result [online]. Website

https://sernecportal.org/portal/ [accessed 9 Jan 2024].
