# Supplementary material for: Semi‐automated assessment for NatureServe subnational conservation status ranks for state floras
Source: Appl Plant Sci. 2026 Mar 19;14(2):e70046. doi: 10.1002/aps3.70046 (PMC13103536; doi:10.1002/aps3.70046)
Supplement: Supplementary file 2 — Appendix S2. List of 2404 native Tennessee species ranked using the semi‐automated methods, S‐ranks from each method, and calculated criteria used to assign ranks. [file APS3-14-e70046-s003.docx]

Appendix S2. List of 2404 native Tennessee species ranked using the semi-automated methods, S-ranks from each method, and calculated criteria used to assign ranks.

| **Scientific name^a,b^** | **Surrounding state mode S-rank** | **County percentage S-rank** | **FME including AOO S-rank** | **FME excluding AOO S-rank** | **RARECAT including AOO S-rank** | **RARECAT excluding AOO S-rank** | **ArcGIS Pro including AOO S-rank** | **ArcGIS Pro excluding AOO S-rank** | **Area of occupancy (number of 2 x 2 km grid cells)^c^** | **Range extent (km^2^)** | **Number of occurrences (1 km separation distance)** |
| --- | --- | --- | --- | --- | --- | --- | --- | --- | --- | --- | --- |
| **ASPLENIACEAE** |  |  |  |  |  |  |  |  |  |  |  |
| *Asplenium ebenoides* | S1 | S1 | S1 | S1 | S1 | S1 | S1 | S1 | 1 | <1 | 1 |
| *Asplenium bradleyi* | S2 | S3 | S2 | S2 | S2 | S2 | S2 | S2 | 7 | 7,033 | 7 |
| *Asplenium montanum* |  | S4 | S3 | S4 | S3 | S4 | S4 | S4 | 121 | 49,590 | 107 |
| *Asplenium pinnatifidum* | S3 | S3 | S2 | S2 | S2 | S2 | S2 | S2 | 11 | 8,933 | 11 |
| *Asplenium platyneuron* | S5 | S5 | S4 | S5 | S4 | S5 | S4 | S5 | 512 | 125,570 | 450 |
| *Asplenium resiliens* | S4 | S5 | S3 | S3 | S3 | S3 | S3 | S3 | 57 | 65,466 | 52 |
| *Asplenium rhizophyllum* | S5 | S5 | S4 | S4 | S4 | S4 | S4 | S4 | 167 | 97,244 | 154 |
| *Asplenium ruta-muraria* | S1 | S4 | S2 | S3 | S3 | S3 | S2 | S3 | 20 | 55,189 | 20 |
| *Asplenium scolopendrium* |  | S1 |  |  |  |  |  |  |  |  |  |
| *Asplenium trichomanes* | S5 | S4 | S3 | S3 | S3 | S3 | S3 | S3 | 42 | 47,325 | 38 |
| *Asplenium trudellii* | S1 | S1 |  |  |  |  |  |  |  |  |  |
| **ATHYRIACEAE** |  |  |  |  |  |  |  |  |  |  |  |
| *Athyrium angustum* |  | S2 | S1 | S1 | S1 | S1 | S1 | S1 | 1 | <1 | 1 |
| *Athyrium asplenioides* | S5 | S5 | S4 | S4 | S4 | S4 | S4 | S4 | 191 | 117,230 | 182 |
| *Deparia acrostichoides* |  | S4 | S3 | S3 | S3 | S3 | S3 | S3 | 53 | 90,938 | 51 |
| **BLECHNACEAE** |  |  |  |  |  |  |  |  |  |  |  |
| *Anchistea virginica* | S5 | S2 | S2 | S2 | S2 | S2 | S2 | S2 | 10 | 5,877 | 10 |
| *Lorinseria areolata* | S5 | S3 | S3 | S4 | S3 | S4 | S3 | S4 | 85 | 93,635 | 84 |
| **CYSTOPTERIDACEAE** |  |  |  |  |  |  |  |  |  |  |  |
| *Cystopteris bulbifera* | S5 | S5 | S3 | S3 | S3 | S3 | S3 | S3 | 37 | 74,166 | 35 |
| *Cystopteris protrusa* | S5 | S5 | S3 | S3 | S3 | S3 | S3 | S3 | 78 | 114,659 | 71 |
| *Cystopteris tennesseensis* | S1 | S5 | S2 | S3 | S2 | S3 | S2 | S3 | 13 | 55,213 | 13 |
| *Cystopteris tenuis* | S1 | S3 | S1 | S1 | S1 | S1 | S1 | S1 | 3 | 3,765 | 3 |
| **DENNSTAEDTIACEAE** |  |  |  |  |  |  |  |  |  |  |  |
| *Pteridium latiusculum* | S5 | S4 | S1 | S1 | S1 | S1 | S1 | S1 | 2 | 38 | 2 |
| *Pteridium pseudocaudatum* | S5 | S1 | S4 | S4 | S4 | S4 | S4 | S4 | 146 | 89,243 | 146 |
| *Sitobolium punctilobulum* |  | S4 | S3 | S3 | S3 | S3 | S3 | S3 | 23 | 35,705 | 23 |
| **DIPLAZIOPSIDACEAE** |  |  |  |  |  |  |  |  |  |  |  |
| *Homalosorus pycnocarpos* | S4 | S3 | S3 | S4 | S3 | S4 | S3 | S4 | 83 | 115,062 | 85 |
| **DRYOPTERIDACEAE** |  |  |  |  |  |  |  |  |  |  |  |
| *Dryopteris campyloptera* | S3 | S3 | S2 | S2 | S2 | S2 | S2 | S2 | 14 | 11,846 | 14 |
| *Dryopteris carthusiana* |  | S3 | S2 | S2 | S2 | S2 | S2 | S2 | 6 | 9,084 | 6 |
| *Dryopteris celsa* | S2 | S4 | S2 | S3 | S2 | S3 | S2 | S3 | 10 | 52,574 | 10 |
| *Dryopteris cristata* |  | S1 |  |  | S2 | S1 | S2 | S2 | 16 | 839 | 15 |
| *Dryopteris goldieana* |  | S4 | S2 | S3 | S2 | S3 | S2 | S3 | 6 | 35,497 | 6 |
| *Dryopteris intermedia* | S5 | S4 | S4 | S4 | S4 | S4 | S4 | S4 | 131 | 87,875 | 106 |
| *Dryopteris marginalis* | S5 | S5 | S4 | S4 | S4 | S4 | S4 | S4 | 133 | 79,986 | 120 |
| *Polystichum acrostichoides* | S5 | S5 | S4 | S5 | S4 | S5 | S4 | S5 | 950 | 133,050 | 788 |
| **EQUISETACEAE** |  |  |  |  |  |  |  |  |  |  |  |
| *Equisetum arvense* | S5 | S5 | S3 | S3 | S3 | S3 | S3 | S3 | 22 | 79,514 | 22 |
| *Equisetum hyemale* | S5 | S5 | S2 | S3 | S3 | S3 | S3 | S3 | 18 | 116,246 | 18 |
| **HUPERZIACEAE** |  |  |  |  |  |  |  |  |  |  |  |
| *Huperzia appressa* |  | S1 | S1 | S1 | S1 | S1 | S1 | S1 | 2 | 13 | 2 |
| *Huperzia lucidula* | S5 | S4 | S4 | S4 | S4 | S4 | S4 | S4 | 176 | 48,441 | 147 |
| *Huperzia porophila* |  | S2 | S2 | S2 | S2 | S2 | S2 | S2 | 8 | 9,765 | 7 |
| **HYMENOPHYLLACEAE** |  |  |  |  |  |  |  |  |  |  |  |
| *Crepidomanes intricatum* | S3 | S1 | S1 | S1 | S1 | S1 | S1 | S1 | 2 | 107 | 2 |
| *Didymoglossum petersii* | S2 | S1 | S1 | S1 | S1 | S1 | S1 | S1 | 2 | 4 | 2 |
| *Hymenophyllum tayloriae* | S1 | S1 |  |  | S2 | S2 | S2 | S2 | 9 | 2,040 | 8 |
| *Vandenboschia boschiana* | S1 | S1 | S2 | S2 | S2 | S2 | S2 | S2 | 8 | 3,346 | 8 |
| **ISOETACEAE** |  |  |  |  |  |  |  |  |  |  |  |
| *Isoetes appalachiana* | S2 | S1 | S1 | S1 | S1 | S1 | S1 | S1 | 1 | <1 | 1 |
| *Isoetes butleri* | S1 | S3 | S2 | S1 | S2 | S1 | S2 | S1 | 8 | 757 | 8 |
| *Isoetes engelmannii* | S4 | S4 | S2 | S3 | S2 | S3 | S2 | S3 | 19 | 52,192 | 18 |
| *Isoetes melanopoda* | S2 | S2 | S2 | S2 | S2 | S2 | S2 | S3 | 12 | 17,882 | 12 |
| *Isoetes valida* |  | S3 | S2 | S3 | S2 | S3 | S2 | S3 | 6 | 31,172 | 6 |
| **LYCOPODIACEAE** |  |  |  |  |  |  |  |  |  |  |  |
| *Dendrolycopodium dendroideum* |  | S1 | S1 | S2 | S1 | S2 | S1 | S2 | 5 | 11,691 | 4 |
| *Dendrolycopodium hickeyi* |  | S3 | S2 | S2 | S2 | S2 | S2 | S2 | 6 | 1,055 | 6 |
| *Dendrolycopodium obscurum* | S4 | S4 | S3 | S3 | S3 | S3 | S3 | S3 | 81 | 69,389 | 74 |
| *Diphasiastrum digitatum* | S5 | S5 | S4 | S5 | S4 | S5 | S4 | S5 | 435 | 114,479 | 383 |
| *Diphasiastrum tristachyum* | S1 | S3 | S2 | S3 | S2 | S2 | S2 | S3 | 10 | 24,502 | 10 |
| *Lycopodiella alopecuroides* | S5 | S2 |  |  | S2 | S1 | S2 | S1 | 13 | 349 | 13 |
| *Lycopodiella appressa* |  | S2 | S3 | S3 | S3 | S3 | S3 | S3 | 26 | 51,193 | 23 |
| *Lycopodiella inundata* | S1 | S1 | S1 | S1 | S1 | S1 | S1 | S1 | 1 | <1 | 1 |
| *Lycopodium clavatum* |  | S2 | S3 | S2 | S2 | S2 | S3 | S3 | 26 | 13,488 | 19 |
| **LYGODIACEAE** |  |  |  |  |  |  |  |  |  |  |  |
| *Lygodium palmatum* | S3 | S4 | S4 | S4 | S4 | S4 | S4 | S4 | 198 | 59,207 | 180 |
| **MARSILEACEAE** |  |  |  |  |  |  |  |  |  |  |  |
| *Pilularia americana* | S2 | S1 | S2 | S2 | S2 | S2 | S2 | S2 | 6 | 14,207 | 6 |
| **ONOCLEACEAE** |  |  |  |  |  |  |  |  |  |  |  |
| *Onoclea sensibilis* | S5 | S5 | S4 | S4 | S4 | S4 | S4 | S4 | 238 | 131,430 | 227 |
| **OPHIOGLOSSACEAE** |  |  |  |  |  |  |  |  |  |  |  |
| *Botrychium matricariifolium* | S1 | S2 | S1 | S1 | S1 | S1 | S1 | S1 | 4 | 1,377 | 4 |
| *Botrychium simplex* |  | S1 | S1 | S1 | S1 | S1 | S1 | S1 | 1 | <1 | 1 |
| *Botrypus virginianus* | S5 | S5 | S4 | S5 | S4 | S5 | S4 | S5 | 405 | 129,936 | 358 |
| *Ophioglossum engelmannii* | S3 | S3 | S3 | S3 | S3 | S3 | S3 | S3 | 39 | 31,677 | 37 |
| *Ophioglossum pycnostichum* |  | S4 | S2 | S3 | S3 | S3 | S2 | S3 | 13 | 57,420 | 14 |
| *Sceptridium biternatum* | S5 | S4 | S3 | S3 | S3 | S3 | S3 | S3 | 38 | 93,482 | 37 |
| *Sceptridium dissectum* | S4 | S4 | S3 | S4 | S3 | S4 | S3 | S4 | 92 | 114,028 | 88 |
| *Sceptridium jenmanii* |  | S1 | S2 | S2 | S2 | S2 | S2 | S2 | 6 | 2,130 | 6 |
| *Sceptridium oneidense* |  | S1 | S1 | S1 | S1 | S1 | S1 | S1 | 2 | 5 | 2 |
| **OSMUNDACEAE** |  |  |  |  |  |  |  |  |  |  |  |
| *Claytosmunda claytoniana* |  | S1 | S3 | S3 | S3 | S3 | S3 | S3 | 31 | 70,173 | 31 |
| *Osmunda spectabilis* | S5 | S4 | S4 | S4 | S4 | S4 | S4 | S4 | 140 | 97,614 | 134 |
| *Osmundastrum cinnamomeum* | S5 | S4 | S4 | S4 | S4 | S4 | S4 | S4 | 199 | 98,162 | 188 |
| **POLYPODIACEAE** |  |  |  |  |  |  |  |  |  |  |  |
| *Pleopeltis michauxiana* | S5 | S4 | S4 | S4 | S4 | S4 | S4 | S4 | 224 | 122,366 | 205 |
| *Polypodium appalachianum* | S4 | S4 | S3 | S3 | S3 | S3 | S3 | S3 | 42 | 36,072 | 41 |
| *Polypodium virginianum* | S5 | S4 | S3 | S3 | S3 | S3 | S3 | S3 | 39 | 53,143 | 38 |
| **PTERIDACEAE** |  |  |  |  |  |  |  |  |  |  |  |
| *Adiantum capillus-veneris* | S2 | S4 | S3 | S3 | S3 | S3 | S3 | S3 | 25 | 77,769 | 24 |
| *Adiantum pedatum* | S5 | S5 | S4 | S4 | S4 | S5 | S4 | S4 | 314 | 125,744 | 281 |
| *Myriopteris alabamensis* | S1 | S4 | S3 | S3 | S3 | S3 | S3 | S3 | 34 | 59,410 | 31 |
| *Myriopteris lanosa* | S5 | S4 | S3 | S3 | S3 | S3 | S3 | S3 | 77 | 83,716 | 69 |
| *Myriopteris tomentosa* | S3 | S3 | S2 | S3 | S2 | S3 | S2 | S3 | 14 | 39,649 | 14 |
| *Pellaea atropurpurea* | S4 | S5 | S3 | S4 | S3 | S4 | S4 | S4 | 125 | 102,503 | 121 |
| *Pellaea glabella* |  | S3 | S2 | S3 | S2 | S3 | S2 | S3 | 13 | 56,465 | 13 |
| *Vittaria appalachiana* | S3 | S3 | S3 | S3 | S3 | S3 | S3 | S3 | 22 | 37,032 | 22 |
| **SALVINIACEAE** |  |  |  |  |  |  |  |  |  |  |  |
| *Azolla caroliniana* |  | S4 | S1 | S1 | S1 | S1 | S1 | S1 | 4 | 1,108 | 4 |
| **SELAGINELLACEAE** |  |  |  |  |  |  |  |  |  |  |  |
| *Bryodesma rupestre* | S5 | S1 | S1 | S1 | S1 | S1 | S1 | S1 | 2 | <1 | 1 |
| *Lycopodioides apodum* | S5 | S3 | S3 | S3 | S3 | S3 | S3 | S3 | 28 | 82,046 | 27 |
| *Lycopodioides eclipes* |  | S1 | S1 | S1 | S1 | S1 | S1 | S1 | 1 | 1 | 2 |
| **THELYPTERIDACEAE** |  |  |  |  |  |  |  |  |  |  |  |
| *Amauropelta noveboracensis* | S5 | S3 | S4 | S4 | S4 | S4 | S4 | S4 | 164 | 89,420 | 143 |
| *Phegopteris connectilis* |  | S1 |  |  | S1 | S1 | S2 | S1 | 4 | 540 | 3 |
| *Phegopteris hexagonoptera* | S5 | S5 | S4 | S4 | S4 | S4 | S4 | S4 | 178 | 115,117 | 167 |
| *Thelypteris palustris* | S5 | S4 | S2 | S3 | S2 | S3 | S2 | S3 | 8 | 50,780 | 8 |
| **WOODSIACEAE** |  |  |  |  |  |  |  |  |  |  |  |
| *Woodsia appalachiana* | S1 | S1 | S1 | S1 | S1 | S1 | S1 | S1 | 1 | <1 | 1 |
| *Woodsia obtusa* |  | S5 | S3 | S3 | S3 | S3 | S3 | S3 | 27 | 62,637 | 26 |
| **CUPRESSACEAE** |  |  |  |  |  |  |  |  |  |  |  |
| *Juniperus virginiana* | S5 | S5 | S4 | S5 | S4 | S5 | S4 | S5 | 690 | 129,014 | 630 |
| *Taxodium distichum* | S5 | S5 | S4 | S4 | S4 | S4 | S4 | S4 | 202 | 127,665 | 180 |
| *Thuja occidentalis* |  | S4 | S2 | S3 | S2 | S3 | S2 | S3 | 19 | 50,278 | 19 |
| **PINACEAE** |  |  |  |  |  |  |  |  |  |  |  |
| *Abies fraseri* |  | S3 | S2 | S2 | S2 | S2 | S2 | S2 | 28 | 3,051 | 20 |
| *Picea rubens* | S3 | S3 | S3 | S3 | S3 | S3 | S3 | S3 | 33 | 2,733 | 26 |
| *Pinus echinata* | S5 | S5 | S3 | S3 | S3 | S3 | S3 | S3 | 85 | 111,536 | 76 |
| *Pinus pungens* |  | S4 | S3 | S3 | S3 | S3 | S3 | S3 | 42 | 9,164 | 41 |
| *Pinus rigida* | S4 | S4 | S3 | S3 | S3 | S3 | S3 | S3 | 28 | 42,525 | 29 |
| *Pinus strobus* | S5 | S5 | S4 | S5 | S4 | S5 | S4 | S5 | 358 | 105,205 | 329 |
| *Pinus taeda* | S5 | S5 | S3 | S4 | S3 | S4 | S3 | S4 | 103 | 102,396 | 99 |
| *Pinus virginiana* | S5 | S5 | S4 | S4 | S4 | S4 | S4 | S4 | 217 | 100,395 | 207 |
| *Tsuga canadensis* | S4 | S5 | S4 | S5 | S4 | S5 | S4 | S5 | 432 | 84,685 | 354 |
| *Tsuga caroliniana* | S2 | S3 |  |  | S3 | S3 | S3 | S3 | 37 | 1,443 | 50 |
| **TAXACEAE** |  |  |  |  |  |  |  |  |  |  |  |
| *Taxus canadensis* |  | S1 |  |  | S1 | S1 | S1 | S1 | 1 | <1 | 1 |
| **CABOMBACEAE** |  |  |  |  |  |  |  |  |  |  |  |
| *Brasenia schreberi* | S3 | S4 | S3 | S3 | S3 | S3 | S3 | S3 | 32 | 97,571 | 31 |
| *Cabomba caroliniana* |  | S1 | S1 | S1 | S1 | S1 | S1 | S1 | 2 | 5 | 2 |
| **ILLICIACEAE** |  |  |  |  |  |  |  |  |  |  |  |
| *Schisandra glabra* | S2 | S1 |  |  | S2 | S2 | S2 | S2 | 13 | 8,029 | 11 |
| **NYMPHAEACEAE** |  |  |  |  |  |  |  |  |  |  |  |
| *Nuphar advena* |  | S3 | S2 | S3 | S2 | S3 | S2 | S3 | 19 | 72,499 | 18 |
| *Nymphaea odorata* |  | S3 | S3 | S3 | S3 | S3 | S3 | S3 | 22 | 96,420 | 22 |
| **ANNONACEAE** |  |  |  |  |  |  |  |  |  |  |  |
| *Asimina triloba* | S5 | S5 | S4 | S5 | S4 | S5 | S4 | S5 | 458 | 128,369 | 411 |
| **ARISTOLOCHIACEAE** |  |  |  |  |  |  |  |  |  |  |  |
| *Asarum acuminatum* |  | S3 | S1 | S1 | S1 | S1 | S1 | S1 | 2 | <1 | 2 |
| *Asarum canadense* | S5 | S5 | S4 | S4 | S4 | S4 | S4 | S4 | 226 | 113,649 | 208 |
| *Asarum reflexum* |  | S3 | S1 | S1 | S1 | S1 | S1 | S1 | 1 | <1 | 1 |
| *Endodeca serpentaria* | S4 | S3 | S3 | S3 | S3 | S3 | S3 | S3 | 36 | 94,339 | 36 |
| *Hexastylis arifolia* | S5 | S4 | S4 | S5 | S4 | S5 | S4 | S5 | 409 | 88,417 | 365 |
| *Hexastylis chueyi* |  |  |  |  |  |  |  |  |  |  |  |
| *Hexastylis contracta* | S1 | S2 | S2 | S1 | S2 | S1 | S2 | S2 | 10 | 934 | 9 |
| *Hexastylis harperi* | S2 |  |  |  |  |  |  |  |  |  |  |
| *Hexastylis heterophylla* |  | S3 | S2 | S2 | S2 | S2 | S2 | S2 | 16 | 3,621 | 16 |
| *Hexastylis ruthii* | S3 | S3 | S1 | S1 | S1 | S1 | S1 | S1 | 1 | <1 | 1 |
| *Hexastylis shuttleworthii* | S4 | S3 | S2 | S3 | S2 | S2 | S2 | S3 | 16 | 24,517 | 13 |
| *Hexastylis virginica* |  | S2 |  |  | S2 | S2 | S2 | S2 | 6 | 6,725 | 6 |
| *Isotrema macrophyllum* | S4 | S3 | S3 | S3 | S3 | S3 | S3 | S3 | 26 | 37,173 | 25 |
| *Isotrema tomentosum* |  | S2 | S3 | S3 | S3 | S3 | S3 | S3 | 33 | 51,881 | 31 |
| **CALYCANTHACEAE** |  |  |  |  |  |  |  |  |  |  |  |
| *Calycanthus floridus* |  | S5 | S4 | S4 | S4 | S5 | S4 | S4 | 327 | 113,317 | 271 |
| **LAURACEAE** |  |  |  |  |  |  |  |  |  |  |  |
| *Lindera benzoin* | S5 | S5 | S4 | S4 | S4 | S5 | S4 | S4 | 310 | 127,046 | 281 |
| *Sassafras albidum* | S5 | S5 | S4 | S5 | S4 | S5 | S4 | S5 | 683 | 134,621 | 616 |
| **MAGNOLIACEAE** |  |  |  |  |  |  |  |  |  |  |  |
| *Liriodendron tulipifera* | S5 | S5 | S4 | S5 | S4 | S5 | S4 | S5 | 988 | 133,416 | 846 |
| *Magnolia acuminata* | S5 | S5 | S3 | S4 | S3 | S4 | S3 | S4 | 110 | 88,186 | 105 |
| *Magnolia fraseri* | S5 | S3 | S4 | S4 | S4 | S4 | S4 | S4 | 158 | 34,586 | 133 |
| *Magnolia macrophylla* | S1 | S4 | S4 | S4 | S4 | S4 | S4 | S4 | 151 | 100,398 | 134 |
| *Magnolia tripetala* | S5 | S5 | S4 | S4 | S4 | S4 | S4 | S4 | 216 | 71,892 | 180 |
| *Magnolia virginiana* | S5 | S3 | S3 | S3 | S3 | S3 | S3 | S3 | 52 | 105,895 | 51 |
| **SAURURACEAE** |  |  |  |  |  |  |  |  |  |  |  |
| *Saururus cernuus* | S5 | S5 | S4 | S4 | S4 | S4 | S4 | S4 | 142 | 113,455 | 133 |
| **CERATOPHYLLACEAE** |  |  |  |  |  |  |  |  |  |  |  |
| *Ceratophyllum demersum* | S5 | S4 | S2 | S3 | S2 | S3 | S2 | S3 | 13 | 91,002 | 13 |
| *Ceratophyllum echinatum* |  | S1 | S2 | S3 | S2 | S3 | S2 | S3 | 7 | 24,996 | 7 |
| **AGAVACEAE** |  |  |  |  |  |  |  |  |  |  |  |
| *Agave virginica* |  | S4 | S3 | S3 | S3 | S3 | S3 | S3 | 55 | 85,609 | 53 |
| *Camassia scilloides* |  | S4 | S3 | S3 | S3 | S3 | S3 | S3 | 62 | 64,236 | 54 |
| *Schoenolirion croceum* |  | S2 |  |  | S3 | S3 | S3 | S3 | 47 | 3,190 | 44 |
| *Yucca filamentosa* |  | S4 | S3 | S3 | S3 | S3 | S3 | S3 | 64 | 93,299 | 63 |
| *Yucca flaccida* | S1 | S3 | S2 | S3 | S3 | S3 | S2 | S3 | 21 | 57,016 | 20 |
| **ALISMATACEAE** |  |  |  |  |  |  |  |  |  |  |  |
| *Alisma subcordatum* | S4 | S5 | S3 | S3 | S3 | S3 | S3 | S3 | 24 | 92,403 | 24 |
| *Echinodorus berteroi* | S2 | S1 |  |  |  |  |  |  |  |  |  |
| *Echinodorus cordifolius* | S3 | S4 |  |  | S3 | S3 | S3 | S3 | 27 | 58,184 | 26 |
| *Sagittaria australis* | S4 | S5 | S3 | S3 | S3 | S3 | S3 | S3 | 34 | 84,436 | 33 |
| *Sagittaria brevirostra* |  | S3 |  |  | S1 | S1 | S2 | S1 | 5 | 1,971 | 5 |
| *Sagittaria calycina* |  | S3 | S2 | S3 | S2 | S3 | S2 | S3 | 13 | 79,355 | 13 |
| *Sagittaria graminea* | S2 | S1 |  |  | S1 | S2 | S2 | S2 | 5 | 10,648 | 5 |
| *Sagittaria latifolia* | S5 | S5 | S3 | S3 | S3 | S3 | S3 | S3 | 30 | 114,560 | 29 |
| *Sagittaria platyphylla* | S1 | S1 | S2 | S3 | S2 | S3 | S2 | S3 | 8 | 32,142 | 8 |
| *Sagittaria rigida* | S1 | S1 | S1 | S1 | S1 | S1 | S1 | S1 | 1 | <1 | 1 |
| **ALLIACEAE** |  |  |  |  |  |  |  |  |  |  |  |
| *Allium allegheniense* | S1 | S1 |  |  |  |  |  |  |  |  |  |
| *Allium burdickii* |  | S3 |  |  | S2 | S3 | S2 | S3 | 16 | 39,338 | 15 |
| *Allium canadense* | S5 | S5 | S3 | S3 | S3 | S3 | S3 | S3 | 71 | 106,577 | 71 |
| *Allium cernuum* | S3 | S5 | S3 | S3 | S3 | S3 | S3 | S3 | 59 | 68,477 | 56 |
| *Allium lavendulare* |  | S1 |  |  |  |  |  |  |  |  |  |
| *Allium mobilense* |  | S1 |  |  |  |  |  |  |  |  |  |
| *Allium stellatum* | S1 | S2 |  |  |  |  |  |  |  |  |  |
| *Allium tricoccum* |  | S4 | S2 | S3 | S3 | S3 | S3 | S3 | 37 | 47,557 | 36 |
| *Nothoscordum bivalve* | S4 | S5 | S4 | S4 | S4 | S4 | S4 | S4 | 173 | 93,732 | 156 |
| **AMARYLLIDACEAE** |  |  |  |  |  |  |  |  |  |  |  |
| *Hymenocallis occidentalis* |  | S4 | S3 | S3 | S3 | S4 | S3 | S3 | 86 | 87,521 | 79 |
| **ARACEAE** |  |  |  |  |  |  |  |  |  |  |  |
| *Arisaema dracontium* | S4 | S5 | S4 | S4 | S4 | S4 | S4 | S4 | 156 | 121,961 | 143 |
| *Arisaema pusillum* |  | S3 | S2 | S3 | S2 | S2 | S2 | S3 | 6 | 41,038 | 6 |
| *Arisaema quinatum* |  | S2 | S3 | S3 | S3 | S3 | S3 | S3 | 34 | 45,880 | 31 |
| *Arisaema triphyllum* | S5 | S5 | S4 | S5 | S4 | S5 | S4 | S5 | 461 | 123,896 | 402 |
| *Lemna aequinoctialis* |  | S2 |  |  |  |  |  |  |  |  |  |
| *Lemna minor* |  | S4 | S2 | S3 | S2 | S3 | S2 | S3 | 12 | 41,099 | 12 |
| *Lemna obscura* |  | S1 |  |  |  |  |  |  |  |  |  |
| *Lemna perpusilla* | S4 | S2 | S1 | S1 | S1 | S1 | S1 | S1 | 1 | <1 | 1 |
| *Lemna trisulca* |  | S1 | S1 | S1 | S1 | S1 | S1 | S1 | 1 | <1 | 1 |
| *Lemna valdiviana* |  | S2 |  |  |  |  |  |  |  |  |  |
| *Orontium aquaticum* |  | S3 | S2 | S3 | S3 | S3 | S2 | S3 | 22 | 64,424 | 20 |
| *Peltandra virginica* | S5 | S4 | S3 | S3 | S3 | S3 | S3 | S3 | 33 | 103,257 | 33 |
| *Spirodela polyrhiza* | S4 | S4 | S2 | S3 | S2 | S3 | S2 | S3 | 12 | 39,518 | 12 |
| *Symplocarpus foetidus* |  | S1 |  |  | S2 | S1 | S2 | S2 | 8 | 791 | 8 |
| *Wolffia brasiliensis* | S4 | S3 | S1 | S1 | S1 | S1 | S1 | S1 | 1 | <1 | 1 |
| *Wolffia columbiana* |  | S1 |  |  | S1 | S1 | S1 | S1 | 2 | <1 | 1 |
| *Wolffiella gladiata* | S3 | S1 |  |  |  |  |  |  |  |  |  |
| **COLCHICACEAE** |  |  |  |  |  |  |  |  |  |  |  |
| *Uvularia grandiflora* | S4 | S5 | S4 | S4 | S4 | S4 | S4 | S4 | 143 | 107,729 | 124 |
| *Uvularia perfoliata* | S5 | S5 | S4 | S4 | S4 | S4 | S4 | S4 | 174 | 81,155 | 161 |
| *Uvularia puberula* | S5 | S3 | S3 | S3 | S3 | S3 | S3 | S3 | 75 | 39,248 | 69 |
| *Uvularia sessilifolia* |  | S5 | S3 | S3 | S3 | S3 | S3 | S3 | 24 | 68,583 | 25 |
| **COMMELINACEAE** |  |  |  |  |  |  |  |  |  |  |  |
| *Commelina erecta* | S5 | S4 | S3 | S4 | S3 | S4 | S3 | S4 | 96 | 73,952 | 91 |
| *Commelina virginica* | S5 | S5 | S3 | S3 | S3 | S3 | S3 | S3 | 71 | 101,844 | 66 |
| *Tradescantia ernestiana* | S1 | S1 | S1 | S1 | S1 | S1 | S1 | S1 | 2 | 14 | 2 |
| *Tradescantia hirsuticaulis* |  | S2 | S1 | S1 | S1 | S1 | S1 | S1 | 4 | 245 | 4 |
| *Tradescantia ohiensis* | S4 | S4 | S2 | S3 | S2 | S3 | S2 | S3 | 15 | 72,967 | 14 |
| *Tradescantia subaspera* | S5 | S5 | S3 | S3 | S3 | S3 | S3 | S3 | 72 | 76,348 | 69 |
| *Tradescantia virginiana* | S5 | S5 | S3 | S3 | S3 | S3 | S3 | S3 | 56 | 100,627 | 52 |
| **CYPERACEAE** |  |  |  |  |  |  |  |  |  |  |  |
| *Bolboschoenus fluviatilis* | S1 | S1 |  |  | S1 | S2 | S1 | S2 | 4 | 6,701 | 4 |
| *Bulbostylis capillaris* | S4 | S4 | S1 | S1 | S1 | S1 | S1 | S1 | 4 | 225 | 4 |
| *Bulbostylis coarctata* | S3 | S1 |  |  | S1 | S1 | S1 | S1 | 1 | <1 | 2 |
| *Carex abscondita* |  | S4 | S1 | S1 | S1 | S1 | S1 | S1 | 3 | 22 | 3 |
| *Carex aestivaliformis* | S1 | S1 |  |  |  |  |  |  |  |  |  |
| *Carex aestivalis* | S3 | S3 | S2 | S2 | S2 | S2 | S2 | S2 | 13 | 3,942 | 13 |
| *Carex aggregata* | S4 | S3 | S2 | S3 | S2 | S3 | S2 | S3 | 9 | 29,112 | 9 |
| *Carex alata* | S1 | S2 | S1 | S1 | S1 | S1 | S1 | S1 | 5 | 3,135 | 5 |
| *Carex albicans* | S5 | S5 | S3 | S3 | S3 | S3 | S3 | S3 | 46 | 75,785 | 44 |
| *Carex albolutescens* | S4 | S4 | S2 | S2 | S2 | S2 | S2 | S2 | 7 | 12,281 | 7 |
| *Carex albursina* |  | S5 | S3 | S3 | S3 | S3 | S3 | S3 | 29 | 51,567 | 28 |
| *Carex allegheniensis* |  | S2 | S1 | S1 | S1 | S1 | S1 | S1 | 4 | 577 | 4 |
| *Carex amphibola* | S4 | S5 | S3 | S3 | S3 | S3 | S3 | S3 | 34 | 71,980 | 31 |
| *Carex annectens* | S5 | S5 | S3 | S3 | S3 | S3 | S3 | S3 | 31 | 68,549 | 29 |
| *Carex appalachica* | S1 | S3 | S2 | S2 | S2 | S2 | S2 | S2 | 18 | 3,210 | 18 |
| *Carex argyrantha* |  | S1 |  |  | S1 | S1 | S1 | S1 | 1 | <1 | 1 |
| *Carex atlantica* | S5 | S4 | S2 | S3 | S2 | S3 | S2 | S3 | 17 | 48,649 | 17 |
| *Carex aureolensis* |  | S4 | S2 | S3 | S2 | S3 | S2 | S3 | 8 | 49,058 | 8 |
| *Carex austrina* |  | S2 | S1 | S1 | S1 | S1 | S1 | S1 | 3 | 35 | 2 |
| *Carex austrocaroliniana* | S2 | S4 | S3 | S3 | S3 | S3 | S3 | S3 | 26 | 26,969 | 24 |
| *Carex austrolucorum* | S3 | S3 | S2 | S2 | S2 | S2 | S2 | S2 | 7 | 13,478 | 7 |
| *Carex baileyi* | S4 | S4 | S1 | S1 | S1 | S1 | S1 | S1 | 2 | 27 | 2 |
| *Carex barrattii* | S1 | S1 | S2 | S1 | S2 | S1 | S2 | S1 | 7 | 500 | 7 |
| *Carex basiantha* | S5 | S4 | S2 | S2 | S2 | S2 | S2 | S2 | 6 | 13,483 | 6 |
| *Carex blanda* | S5 | S5 | S3 | S3 | S3 | S3 | S3 | S3 | 59 | 72,599 | 56 |
| *Carex brevior* | S4 | S3 | S2 | S2 | S2 | S2 | S2 | S2 | 4 | 38,192 | 4 |
| *Carex bromoides* |  | S4 | S2 | S3 | S2 | S3 | S2 | S3 | 12 | 38,937 | 12 |
| *Carex brunnescens* |  | S2 | S2 | S2 | S1 | S1 | S1 | S1 | 5 | 2,793 | 6 |
| *Carex bulbostylis* | S4 | S1 |  |  |  |  |  |  |  |  |  |
| *Carex bullata* | S1 | S2 | S2 | S1 | S2 | S1 | S2 | S1 | 6 | 722 | 6 |
| *Carex bushii* | S4 | S3 | S2 | S3 | S2 | S3 | S2 | S3 | 15 | 32,502 | 15 |
| *Carex buxbaumii* | S2 | S1 |  |  | S2 | S1 | S2 | S2 | 5 | 8,515 | 8 |
| *Carex canescens* |  | S1 |  |  |  |  |  |  |  |  |  |
| *Carex careyana* |  | S4 | S2 | S3 | S2 | S3 | S2 | S3 | 18 | 65,622 | 18 |
| *Carex caroliniana* | S5 | S5 | S2 | S3 | S3 | S3 | S2 | S3 | 18 | 64,447 | 20 |
| *Carex cephalophora* | S5 | S5 | S3 | S3 | S3 | S3 | S3 | S3 | 34 | 96,930 | 34 |
| *Carex cherokeensis* |  | S4 | S3 | S3 | S3 | S3 | S3 | S3 | 83 | 73,025 | 72 |
| *Carex communis* | S5 | S5 | S3 | S3 | S3 | S3 | S3 | S3 | 29 | 51,137 | 27 |
| *Carex comosa* | S1 | S3 |  |  | S2 | S3 | S2 | S3 | 8 | 28,476 | 8 |
| *Carex complanata* | S5 | S5 | S2 | S3 | S2 | S3 | S2 | S3 | 15 | 39,952 | 15 |
| *Carex conjuncta* |  | S4 | S2 | S3 | S2 | S3 | S2 | S3 | 9 | 32,697 | 7 |
| *Carex corrugata* |  | S4 | S1 | S1 | S1 | S1 | S1 | S2 | 3 | 4,738 | 3 |
| *Carex crawei* | S3 | S2 | S2 | S2 | S2 | S2 | S2 | S2 | 12 | 6,511 | 13 |
| *Carex crebriflora* | S2 | S4 | S1 | S1 | S1 | S1 | S1 | S1 | 3 | 12 | 3 |
| *Carex crinita* | S5 | S5 | S3 | S3 | S3 | S3 | S3 | S3 | 46 | 61,496 | 45 |
| *Carex cristatella* | S1 | S1 | S1 | S1 | S1 | S1 | S1 | S1 | 1 | <1 | 1 |
| *Carex crus-corvi* | S5 | S4 | S2 | S3 | S2 | S3 | S2 | S3 | 10 | 32,147 | 9 |
| *Carex cumberlandensis* |  | S5 | S3 | S3 | S3 | S3 | S3 | S3 | 36 | 65,557 | 35 |
| *Carex davisii* |  | S2 |  |  | S2 | S2 | S2 | S2 | 6 | 1,698 | 6 |
| *Carex debilis* | S5 | S5 | S3 | S3 | S3 | S3 | S3 | S3 | 46 | 80,553 | 45 |
| *Carex decomposita* | S2 | S3 | S1 | S1 | S1 | S1 | S1 | S1 | 2 | 7 | 2 |
| *Carex digitalis* |  | S5 | S3 | S3 | S3 | S3 | S3 | S3 | 58 | 92,938 | 56 |
| *Carex eburnea* |  | S4 | S2 | S2 | S2 | S2 | S2 | S2 | 6 | 3,963 | 6 |
| *Carex echinata* |  | S1 | S1 | S1 | S1 | S1 | S1 | S1 | 1 | <1 | 1 |
| *Carex emoryi* |  | S1 |  |  |  |  |  |  |  |  |  |
| *Carex festucacea* | S5 | S5 | S2 | S3 | S2 | S3 | S2 | S3 | 14 | 42,995 | 12 |
| *Carex flaccosperma* | S5 | S4 | S2 | S1 | S1 | S1 | S1 | S1 | 6 | 4,903 | 5 |
| *Carex flexuosa* | S3 | S2 | S2 | S1 | S2 | S1 | S2 | S1 | 11 | 244 | 8 |
| *Carex folliculata* |  | S1 | S1 | S1 | S1 | S1 | S1 | S1 | 1 | <1 | 1 |
| *Carex frankii* | S5 | S5 | S3 | S3 | S3 | S3 | S3 | S3 | 43 | 95,108 | 42 |
| *Carex fraseriana* |  | S2 | S3 | S3 | S3 | S4 | S3 | S3 | 77 | 26,800 | 67 |
| *Carex fumosimontana* |  | S1 | S1 | S1 | S1 | S1 | S1 | S1 | 4 | 31 | 3 |
| *Carex gigantea* |  | S3 | S2 | S2 | S2 | S2 | S2 | S3 | 20 | 19,783 | 19 |
| *Carex glaucescens* | S4 | S2 | S2 | S1 | S2 | S1 | S2 | S1 | 8 | 792 | 8 |
| *Carex glaucodea* | S4 | S4 | S2 | S3 | S2 | S3 | S2 | S3 | 13 | 49,015 | 12 |
| *Carex gracilescens* |  | S4 | S1 | S1 | S1 | S1 | S1 | S1 | 3 | 690 | 3 |
| *Carex gracillima* | S1 | S4 | S1 | S2 | S1 | S2 | S1 | S2 | 4 | 13,482 | 4 |
| *Carex granularis* | S5 | S5 | S3 | S3 | S3 | S3 | S3 | S3 | 39 | 55,647 | 37 |
| *Carex gravida* |  | S4 | S2 | S3 | S2 | S3 | S2 | S3 | 15 | 35,699 | 14 |
| *Carex grayi* |  | S5 | S3 | S3 | S3 | S3 | S3 | S3 | 72 | 88,202 | 67 |
| *Carex grisea* |  | S4 | S2 | S3 | S2 | S3 | S2 | S3 | 9 | 36,497 | 8 |
| *Carex gynandra* | S5 | S3 | S2 | S2 | S2 | S2 | S2 | S2 | 15 | 10,328 | 12 |
| *Carex hirsutella* | S5 | S5 | S3 | S3 | S3 | S3 | S3 | S3 | 35 | 86,139 | 34 |
| *Carex hirtifolia* | S4 | S2 |  |  | S2 | S2 | S2 | S3 | 8 | 19,173 | 8 |
| *Carex hitchcockiana* | S1 | S2 |  |  | S2 | S3 | S2 | S3 | 10 | 27,352 | 10 |
| *Carex howei* | S3 | S3 | S1 | S1 | S1 | S1 | S1 | S1 | 2 | 52 | 2 |
| *Carex hyalina* |  | S1 | S1 | S1 | S1 | S1 | S1 | S1 | 1 | <1 | 1 |
| *Carex hyalinolepis* | S5 | S2 | S1 | S1 | S1 | S1 | S1 | S1 | 3 | 28 | 3 |
| *Carex hystericina* |  | S3 | S2 | S2 | S2 | S2 | S2 | S2 | 5 | 27,951 | 5 |
| *Carex interior* | S1 | S2 | S1 | S1 | S1 | S1 | S1 | S1 | 2 | 1 | 2 |
| *Carex intumescens* | S5 | S5 | S3 | S3 | S3 | S3 | S3 | S3 | 66 | 112,040 | 64 |
| *Carex jamesii* | S5 | S5 | S3 | S3 | S3 | S3 | S3 | S3 | 28 | 49,053 | 28 |
| *Carex joorii* | S5 | S4 | S2 | S3 | S2 | S3 | S2 | S3 | 9 | 26,974 | 9 |
| *Carex kraliana* | S4 | S5 | S3 | S3 | S3 | S3 | S3 | S3 | 27 | 71,842 | 27 |
| *Carex laevivaginata* |  | S4 | S2 | S3 | S2 | S3 | S2 | S3 | 19 | 55,180 | 19 |
| *Carex laxiculmis* | S3 | S4 | S2 | S3 | S2 | S3 | S2 | S3 | 13 | 28,820 | 13 |
| *Carex laxiflora* | S5 | S5 | S3 | S4 | S3 | S4 | S3 | S4 | 89 | 90,730 | 86 |
| *Carex leavenworthii* | S5 | S5 | S2 | S3 | S2 | S3 | S2 | S3 | 21 | 84,029 | 19 |
| *Carex leptalea* | S5 | S4 | S2 | S3 | S2 | S3 | S2 | S2 | 8 | 27,820 | 8 |
| *Carex leptonervia* | S3 | S3 | S2 | S2 | S2 | S2 | S2 | S2 | 9 | 2,308 | 9 |
| *Carex lonchocarpa* | S5 | S1 | S1 | S1 | S1 | S1 | S1 | S1 | 1 | <1 | 1 |
| *Carex longii* | S5 | S4 | S2 | S2 | S1 | S2 | S2 | S2 | 4 | 24,488 | 4 |
| *Carex louisianica* | S4 | S4 | S2 | S3 | S2 | S3 | S2 | S3 | 16 | 48,445 | 16 |
| *Carex lupuliformis* | S1 | S3 | S1 | S1 | S1 | S1 | S1 | S1 | 1 | <1 | 1 |
| *Carex lupulina* | S5 | S5 | S3 | S3 | S3 | S3 | S3 | S3 | 33 | 84,005 | 34 |
| *Carex lurida* | S5 | S5 | S3 | S4 | S3 | S4 | S3 | S4 | 104 | 109,574 | 98 |
| *Carex manhartii* |  | S1 |  |  | S2 | S2 | S2 | S2 | 13 | 1,585 | 10 |
| *Carex meadii* |  | S3 | S1 | S2 | S1 | S2 | S1 | S2 | 5 | 12,330 | 5 |
| *Carex mesochorea* | S1 | S3 | S1 | S2 | S1 | S2 | S2 | S2 | 5 | 17,232 | 5 |
| *Carex misera* |  | S1 | S1 | S1 | S1 | S1 | S1 | S1 | 1 | <1 | 1 |
| *Carex mitchelliana* | S2 | S3 | S1 | S2 | S1 | S2 | S1 | S2 | 3 | 11,788 | 3 |
| *Carex molesta* | S4 | S3 | S2 | S2 | S2 | S2 | S2 | S3 | 8 | 19,302 | 8 |
| *Carex molestiformis* | S1 | S2 | S1 | S1 | S1 | S1 | S1 | S1 | 4 | 1,299 | 4 |
| *Carex muehlenbergii* | S4 | S4 | S2 | S3 | S2 | S3 | S2 | S3 | 16 | 51,361 | 16 |
| *Carex muskingumensis* |  | S1 |  |  | S1 | S1 | S1 | S1 | 1 | <1 | 1 |
| *Carex nigromarginata* | S5 | S5 | S3 | S3 | S3 | S3 | S3 | S3 | 36 | 80,604 | 36 |
| *Carex normalis* | S1 | S4 | S2 | S3 | S2 | S2 | S2 | S3 | 10 | 22,081 | 10 |
| *Carex oblita* |  | S1 |  |  |  |  |  |  |  |  |  |
| *Carex oklahomensis* | S3 | S2 | S2 | S3 | S2 | S3 | S2 | S3 | 6 | 40,074 | 6 |
| *Carex oligocarpa* |  | S5 | S2 | S3 | S2 | S3 | S2 | S3 | 19 | 38,147 | 19 |
| *Carex ouachitana* |  | S1 | S1 | S1 | S1 | S1 | S1 | S1 | 2 | 19 | 2 |
| *Carex ovalis* |  | S1 |  |  |  |  |  |  |  |  |  |
| *Carex oxylepis* | S4 | S5 | S2 | S3 | S2 | S3 | S2 | S3 | 15 | 57,152 | 14 |
| *Carex pallescens* |  | S1 |  |  | S1 | S1 | S1 | S1 | 1 | <1 | 1 |
| *Carex pedunculata* | S1 | S3 | S2 | S3 | S2 | S2 | S2 | S3 | 12 | 29,758 | 10 |
| *Carex pellita* | S1 | S1 |  |  | S1 | S1 | S1 | S1 | 2 | <1 | 2 |
| *Carex pensylvanica* | S5 | S5 | S3 | S3 | S3 | S3 | S3 | S3 | 30 | 48,631 | 25 |
| *Carex picta* | S3 | S3 | S2 | S2 | S2 | S2 | S2 | S2 | 10 | 2,718 | 9 |
| *Carex pigra* |  | S3 | S1 | S1 | S1 | S1 | S1 | S1 | 2 | 15 | 2 |
| *Carex planispicata* | S4 | S4 | S2 | S3 | S2 | S3 | S2 | S3 | 8 | 33,520 | 8 |
| *Carex plantaginea* | S3 | S4 | S3 | S3 | S3 | S4 | S3 | S3 | 86 | 63,844 | 71 |
| *Carex platyphylla* | S5 | S4 | S2 | S2 | S1 | S2 | S1 | S2 | 6 | 7,498 | 5 |
| *Carex prasina* |  | S4 | S3 | S3 | S3 | S3 | S3 | S3 | 34 | 59,691 | 31 |
| *Carex projecta* |  | S1 |  |  |  |  |  |  |  |  |  |
| *Carex purpurifera* | S3 | S4 | S3 | S3 | S3 | S3 | S3 | S3 | 35 | 47,329 | 33 |
| *Carex radiata* |  | S4 | S2 | S3 | S2 | S3 | S2 | S3 | 10 | 79,755 | 10 |
| *Carex reniformis* | S1 | S1 | S1 | S1 | S1 | S1 | S1 | S1 | 1 | <1 | 1 |
| *Carex retroflexa* | S5 | S5 | S2 | S3 | S2 | S3 | S2 | S3 | 13 | 69,208 | 13 |
| *Carex reznicekii* | S2 | S4 | S2 | S3 | S2 | S3 | S2 | S3 | 9 | 38,438 | 9 |
| *Carex roanensis* |  | S1 |  |  | S3 | S3 | S3 | S3 | 38 | 5,069 | 48 |
| *Carex rosea* | S5 | S5 | S3 | S3 | S3 | S3 | S3 | S3 | 35 | 58,673 | 33 |
| *Carex rugosperma* |  | S1 | S1 | S1 | S1 | S1 | S1 | S1 | 1 | <1 | 1 |
| *Carex ruthii* | S3 | S2 |  |  | S3 | S3 | S3 | S3 | 22 | 5,128 | 28 |
| *Carex sangamonensis* |  | S3 | S1 | S1 | S1 | S1 | S1 | S1 | 2 | 3 | 2 |
| *Carex scabrata* |  | S3 | S2 | S2 | S2 | S3 | S2 | S2 | 18 | 3,306 | 14 |
| *Carex scoparia* | S4 | S4 | S2 | S3 | S2 | S3 | S2 | S3 | 14 | 42,436 | 14 |
| *Carex seorsa* | S1 | S3 | S1 | S1 | S1 | S1 | S1 | S1 | 2 | 24 | 2 |
| *Carex shortiana* |  | S3 | S1 | S1 | S1 | S1 | S1 | S1 | 4 | 27 | 3 |
| *Carex socialis* | S1 | S4 | S2 | S2 | S2 | S2 | S2 | S2 | 6 | 5,993 | 6 |
| *Carex sparganioides* |  | S4 | S2 | S3 | S2 | S3 | S2 | S3 | 7 | 34,646 | 7 |
| *Carex squarrosa* | S5 | S5 | S2 | S3 | S3 | S3 | S2 | S3 | 23 | 78,075 | 20 |
| *Carex stipata* | S3 | S4 | S2 | S3 | S2 | S3 | S2 | S3 | 16 | 71,978 | 16 |
| *Carex striatula* | S4 | S5 | S2 | S3 | S2 | S3 | S2 | S3 | 13 | 42,067 | 12 |
| *Carex stricta* | S3 | S3 | S1 | S2 | S1 | S1 | S1 | S2 | 3 | 5,795 | 3 |
| *Carex styloflexa* | S4 | S5 | S3 | S3 | S3 | S3 | S3 | S3 | 31 | 47,128 | 29 |
| *Carex superata* | S1 | S3 | S2 | S2 | S2 | S2 | S2 | S2 | 4 | 31,570 | 4 |
| *Carex swanii* | S3 | S5 | S3 | S3 | S3 | S3 | S3 | S3 | 35 | 33,300 | 27 |
| *Carex tenera* |  | S1 | S1 | S1 | S1 | S1 | S1 | S1 | 2 | 5 | 2 |
| *Carex tetanica* | S3 | S1 |  |  |  |  |  |  |  |  |  |
| *Carex texensis* |  | S5 | S2 | S3 | S2 | S3 | S2 | S3 | 19 | 46,895 | 18 |
| *Carex timida* | S2 | S1 | S2 | S3 | S2 | S3 | S2 | S3 | 9 | 27,053 | 9 |
| *Carex tonsa* |  | S3 | S1 | S1 | S1 | S1 | S1 | S1 | 4 | 1,997 | 4 |
| *Carex torta* |  | S4 | S2 | S3 | S2 | S3 | S2 | S3 | 17 | 46,398 | 17 |
| *Carex triangularis* |  | S2 | S1 | S1 | S1 | S1 | S1 | S1 | 1 | <1 | 1 |
| *Carex tribuloides* | S5 | S5 | S3 | S3 | S3 | S3 | S2 | S3 | 21 | 84,662 | 21 |
| *Carex trichocarpa* | S1 | S1 | S1 | S1 | S1 | S1 | S1 | S1 | 4 | 2,634 | 4 |
| *Carex trisperma* |  | S1 | S1 | S1 | S1 | S1 | S1 | S1 | 1 | <1 | 1 |
| *Carex typhina* | S5 | S4 | S2 | S3 | S2 | S3 | S2 | S3 | 6 | 38,921 | 6 |
| *Carex umbellata* | S5 | S4 | S3 | S3 | S3 | S3 | S3 | S3 | 22 | 64,541 | 21 |
| *Carex utriculata* | S1 | S1 |  |  |  |  |  |  |  |  |  |
| *Carex virescens* |  | S5 | S3 | S3 | S3 | S3 | S3 | S3 | 36 | 36,569 | 34 |
| *Carex vulpinoidea* | S5 | S5 | S3 | S3 | S3 | S3 | S3 | S3 | 64 | 90,943 | 61 |
| *Carex willdenowii* | S1 | S4 | S2 | S3 | S2 | S3 | S2 | S3 | 9 | 34,962 | 9 |
| *Carex woodii* |  | S1 | S1 | S1 | S1 | S1 | S1 | S1 | 1 | <1 | 1 |
| *Cyperus acuminatus* | S5 | S4 | S2 | S2 | S2 | S2 | S2 | S2 | 5 | 48,002 | 5 |
| *Cyperus bipartitus* |  | S3 | S1 | S1 | S1 | S1 | S1 | S1 | 4 | 2,787 | 4 |
| *Cyperus brevifolius* |  | S2 | S1 | S2 | S1 | S2 | S1 | S2 | 4 | 12,003 | 4 |
| *Cyperus croceus* | S5 | S3 | S1 | S1 | S1 | S1 | S1 | S1 | 2 | 31 | 2 |
| *Cyperus dentatus* |  | S1 | S1 | S1 | S1 | S1 | S1 | S1 | 1 | <1 | 1 |
| *Cyperus diandrus* | S1 | S2 | S1 | S1 | S1 | S1 | S1 | S1 | 1 | <1 | 1 |
| *Cyperus echinatus* | S5 | S4 | S3 | S3 | S3 | S3 | S3 | S3 | 46 | 111,379 | 46 |
| *Cyperus erythrorhizos* |  | S4 | S2 | S2 | S2 | S2 | S2 | S2 | 5 | 32,328 | 5 |
| *Cyperus esculentus* |  | S5 |  |  | S2 | S3 | S2 | S3 | 16 | 71,523 | 16 |
| *Cyperus flavescens* | S5 | S5 | S3 | S3 | S3 | S3 | S3 | S3 | 21 | 64,252 | 21 |
| *Cyperus flavicomus* |  | S3 | S1 | S1 | S1 | S1 | S1 | S1 | 1 | <1 | 1 |
| *Cyperus granitophilus* | S2 | S1 | S1 | S1 | S1 | S1 | S1 | S1 | 1 | <1 | 1 |
| *Cyperus hortensis* | S5 | S3 | S2 | S3 | S2 | S3 | S2 | S3 | 6 | 49,235 | 6 |
| *Cyperus hystricinus* | S2 | S1 |  |  |  |  |  |  |  |  |  |
| *Cyperus lancastriensis* | S3 | S4 | S2 | S3 | S2 | S3 | S2 | S3 | 12 | 40,412 | 12 |
| *Cyperus lupulinus* |  | S2 | S1 | S1 | S1 | S1 | S1 | S1 | 2 | <1 | 1 |
| *Cyperus odoratus* |  | S5 | S2 | S3 | S2 | S3 | S2 | S3 | 10 | 46,411 | 10 |
| *Cyperus plukenetii* |  | S1 | S1 | S1 | S1 | S1 | S1 | S1 | 1 | <1 | 1 |
| *Cyperus polystachyos* |  | S3 | S1 | S1 | S1 | S1 | S1 | S1 | 2 | 52 | 2 |
| *Cyperus pseudovegetus* | S5 | S5 | S2 | S3 | S2 | S3 | S2 | S3 | 12 | 61,453 | 12 |
| *Cyperus refractus* | S3 | S4 | S1 | S2 | S1 | S2 | S1 | S2 | 3 | 8,356 | 3 |
| *Cyperus retrofractus* | S3 | S4 | S2 | S2 | S2 | S2 | S2 | S3 | 7 | 17,435 | 7 |
| *Cyperus retrorsus* | S5 | S3 | S1 | S1 | S1 | S1 | S1 | S1 | 2 | 8 | 2 |
| *Cyperus sesquiflorus* |  | S1 |  |  |  |  |  |  |  |  |  |
| *Cyperus squarrosus* | S4 | S4 | S2 | S3 | S2 | S3 | S2 | S3 | 7 | 35,863 | 7 |
| *Cyperus strigosus* | S5 | S5 | S3 | S3 | S3 | S3 | S3 | S3 | 52 | 105,964 | 52 |
| *Cyperus subsquarrosus* |  | S3 | S1 | S1 | S1 | S1 | S1 | S1 | 1 | <1 | 1 |
| *Cyperus surinamensis* |  | S1 |  |  |  |  |  |  |  |  |  |
| *Cyperus virens* |  | S1 |  |  |  |  |  |  |  |  |  |
| *Dulichium arundinaceum* | S5 | S4 | S3 | S3 | S3 | S3 | S3 | S3 | 21 | 51,936 | 21 |
| *Eleocharis acicularis* |  | S4 | S2 | S3 | S2 | S3 | S2 | S3 | 8 | 34,038 | 7 |
| *Eleocharis bifida* | S1 | S3 | S2 | S2 | S2 | S2 | S2 | S2 | 13 | 2,917 | 13 |
| *Eleocharis brittonii* |  | S3 |  |  |  |  |  |  |  |  |  |
| *Eleocharis compressa* |  | S3 | S2 | S3 | S2 | S3 | S2 | S3 | 14 | 30,307 | 13 |
| *Eleocharis elliptica* | S1 | S1 |  |  |  |  |  |  |  |  |  |
| *Eleocharis engelmannii* | S4 | S4 | S2 | S2 | S1 | S2 | S2 | S2 | 4 | 20,556 | 4 |
| *Eleocharis equisetoides* | S3 | S1 |  |  | S1 | S2 | S2 | S2 | 4 | 7,201 | 4 |
| *Eleocharis erythropoda* | S1 | S4 | S1 | S1 | S1 | S1 | S1 | S1 | 3 | 122 | 3 |
| *Eleocharis intermedia* | S1 | S2 |  |  | S1 | S1 | S2 | S2 | 3 | 4,449 | 3 |
| *Eleocharis lanceolata* |  | S1 |  |  | S1 | S1 | S1 | S1 | 2 | 43 | 2 |
| *Eleocharis macrostachya* |  | S3 |  |  |  |  |  |  |  |  |  |
| *Eleocharis microcarpa* | S4 | S3 | S1 | S2 | S1 | S2 | S1 | S2 | 4 | 6,174 | 4 |
| *Eleocharis obtusa* | S5 | S5 | S3 | S3 | S3 | S3 | S3 | S3 | 39 | 99,378 | 39 |
| *Eleocharis olivacea* | S1 |  |  |  |  |  |  |  |  |  |  |
| *Eleocharis ovata* |  | S4 | S1 | S2 | S1 | S2 | S1 | S2 | 3 | 12,252 | 3 |
| *Eleocharis palustris* | S1 | S3 | S1 | S1 | S1 | S1 | S1 | S1 | 1 | <1 | 1 |
| *Eleocharis parvula* | S1 | S1 |  |  |  |  |  |  |  |  |  |
| *Eleocharis quadrangulata* | S4 | S4 | S2 | S3 | S2 | S3 | S2 | S3 | 13 | 44,727 | 13 |
| *Eleocharis tenuis* | S5 | S4 | S2 | S3 | S2 | S3 | S2 | S3 | 14 | 50,077 | 14 |
| *Eleocharis tortilis* | S3 | S1 |  |  | S1 | S1 | S1 | S1 | 3 | 520 | 3 |
| *Eleocharis tuberculosa* | S5 | S3 | S1 | S2 | S1 | S1 | S1 | S2 | 5 | 5,544 | 5 |
| *Eleocharis verrucosa* |  | S3 | S1 | S1 | S1 | S1 | S1 | S1 | 3 | 3,308 | 3 |
| *Eleocharis wolfii* | S1 | S2 |  |  | S1 | S1 | S2 | S1 | 4 | 1,581 | 4 |
| *Eriophorum virginicum* |  | S2 |  |  | S2 | S3 | S2 | S2 | 6 | 21,365 | 6 |
| *Fimbristylis annua* |  | S3 | S1 | S1 | S1 | S1 | S1 | S1 | 1 | <1 | 1 |
| *Fimbristylis autumnalis* | S5 | S5 | S2 | S3 | S2 | S3 | S2 | S3 | 13 | 35,333 | 13 |
| *Fimbristylis perpusilla* | S1 | S1 |  |  | S1 | S1 | S1 | S1 | 1 | <1 | 1 |
| *Fimbristylis puberula* |  | S2 |  |  | S2 | S2 | S2 | S2 | 7 | 5,828 | 7 |
| *Fimbristylis vahlii* |  | S3 |  |  | S1 | S1 | S1 | S1 | 3 | 76 | 3 |
| *Fuirena squarrosa* | S4 | S2 |  |  | S2 | S3 | S2 | S3 | 10 | 33,154 | 10 |
| *Isolepis carinata* | S4 | S4 | S2 | S2 | S2 | S2 | S2 | S2 | 6 | 3,646 | 6 |
| *Rhynchospora caduca* | S3 | S1 | S1 | S1 | S1 | S1 | S1 | S1 | 4 | 143 | 4 |
| *Rhynchospora capillacea* | S1 | S1 | S1 | S1 | S1 | S1 | S1 | S1 | 1 | <1 | 1 |
| *Rhynchospora capitellata* | S5 | S5 | S3 | S3 | S3 | S3 | S3 | S3 | 23 | 37,446 | 23 |
| *Rhynchospora chalarocephala* | S4 | S1 |  |  | S1 | S1 | S1 | S1 | 4 | 666 | 3 |
| *Rhynchospora corniculata* | S4 | S5 | S2 | S3 | S2 | S3 | S2 | S3 | 16 | 48,602 | 16 |
| *Rhynchospora debilis* | S3 | S1 |  |  | S1 | S1 | S1 | S1 | 1 | <1 | 1 |
| *Rhynchospora globularis* | S5 | S3 | S1 | S2 | S1 | S2 | S1 | S2 | 4 | 14,478 | 4 |
| *Rhynchospora glomerata* | S5 | S4 | S2 | S3 | S2 | S3 | S2 | S3 | 16 | 26,766 | 16 |
| *Rhynchospora gracilenta* |  | S3 | S1 | S2 | S1 | S1 | S1 | S2 | 5 | 8,405 | 5 |
| *Rhynchospora harveyi* |  | S1 |  |  | S2 | S2 | S2 | S2 | 3 | 20,217 | 3 |
| *Rhynchospora inexpansa* | S5 | S1 |  |  |  |  |  |  |  |  |  |
| *Rhynchospora macrostachya* | S3 | S1 | S2 | S2 | S2 | S2 | S2 | S2 | 14 | 11,435 | 14 |
| *Rhynchospora perplexa* | S1 | S2 |  |  | S2 | S1 | S2 | S2 | 8 | 734 | 8 |
| *Rhynchospora rariflora* | S3 | S1 | S1 | S1 | S1 | S1 | S1 | S1 | 2 | 6 | 2 |
| *Rhynchospora recognita* |  | S3 | S2 | S2 | S2 | S2 | S2 | S2 | 7 | 13,247 | 7 |
| *Rhynchospora stiletto* | S1 | S1 | S1 | S1 | S1 | S1 | S2 | S1 | 6 | 984 | 5 |
| *Schoenoplectiella purshiana* | S4 | S3 | S2 | S2 | S2 | S2 | S2 | S3 | 6 | 17,110 | 6 |
| *Schoenoplectus pungens* |  | S3 | S1 | S1 | S1 | S1 | S1 | S1 | 2 | 33 | 2 |
| *Schoenoplectus subterminalis* | S1 | S1 |  |  | S1 | S1 | S1 | S1 | 1 | <1 | 1 |
| *Schoenoplectus tabernaemontani* | S4 | S4 | S2 | S3 | S2 | S3 | S2 | S3 | 8 | 63,586 | 8 |
| *Scirpus atrovirens* |  | S5 | S3 | S3 | S3 | S3 | S3 | S3 | 23 | 73,652 | 23 |
| *Scirpus cyperinus* | S5 | S5 | S3 | S3 | S3 | S3 | S3 | S3 | 47 | 104,968 | 44 |
| *Scirpus expansus* |  | S2 | S1 | S1 | S1 | S1 | S1 | S1 | 5 | 857 | 5 |
| *Scirpus georgianus* | S4 | S5 | S2 | S3 | S2 | S3 | S2 | S3 | 11 | 60,393 | 11 |
| *Scirpus pendulus* |  | S5 | S2 | S3 | S2 | S3 | S2 | S3 | 16 | 26,732 | 16 |
| *Scirpus polyphyllus* |  | S5 | S2 | S3 | S2 | S3 | S2 | S3 | 16 | 51,826 | 15 |
| *Scleria ciliata* | S4 | S2 | S1 | S2 | S1 | S2 | S1 | S2 | 5 | 15,055 | 5 |
| *Scleria minor* |  | S1 |  |  |  |  |  |  |  |  |  |
| *Scleria muehlenbergii* |  | S2 | S1 | S1 | S1 | S1 | S1 | S1 | 1 | <1 | 1 |
| *Scleria nitida* |  | S1 |  |  |  |  |  |  |  |  |  |
| *Scleria oligantha* | S4 | S5 | S3 | S3 | S3 | S3 | S3 | S3 | 23 | 54,292 | 23 |
| *Scleria pauciflora* |  | S5 | S2 | S3 | S2 | S3 | S2 | S3 | 9 | 28,331 | 9 |
| *Scleria triglomerata* | S4 | S4 | S2 | S2 | S2 | S2 | S2 | S2 | 13 | 15,084 | 13 |
| *Scleria verticillata* |  | S1 |  |  | S2 | S2 | S2 | S2 | 7 | 5,619 | 7 |
| *Trichophorum cespitosum* |  | S1 |  |  | S1 | S1 | S1 | S1 | 3 | 48 | 2 |
| **DIOSCOREACEAE** |  |  |  |  |  |  |  |  |  |  |  |
| *Dioscorea villosa* | S5 | S5 | S4 | S4 | S4 | S5 | S4 | S5 | 326 | 123,057 | 288 |
| **ERIOCAULACEAE** |  |  |  |  |  |  |  |  |  |  |  |
| *Eriocaulon decangulare* |  | S1 | S1 | S1 | S1 | S1 | S1 | S1 | 4 | 47 | 4 |
| *Lachnocaulon anceps* |  | S1 |  |  | S1 | S1 | S1 | S1 | 2 | 30 | 2 |
| **HAEMODORACEAE** |  |  |  |  |  |  |  |  |  |  |  |
| *Lachnanthes caroliana* |  | S1 | S1 | S1 | S1 | S1 | S1 | S1 | 5 | 251 | 5 |
| **HYDROCHARITACEAE** |  |  |  |  |  |  |  |  |  |  |  |
| *Elodea canadensis* | S1 | S4 | S1 | S2 | S1 | S1 | S1 | S2 | 5 | 6,222 | 5 |
| *Elodea nuttallii* | S2 | S3 |  |  | S2 | S2 | S2 | S2 | 8 | 9,278 | 8 |
| *Limnobium spongia* | S2 | S2 | S1 | S1 | S1 | S1 | S1 | S1 | 3 | 174 | 3 |
| *Najas gracillima* | S2 | S2 | S1 | S1 | S1 | S1 | S1 | S1 | 4 | 3,815 | 4 |
| *Najas guadalupensis* |  | S4 | S1 | S1 | S1 | S1 | S1 | S1 | 1 | <1 | 1 |
| *Vallisneria americana* |  | S3 | S1 | S1 | S1 | S1 | S1 | S1 | 2 | 32 | 2 |
| **HYPOXIDACEAE** |  |  |  |  |  |  |  |  |  |  |  |
| *Hypoxis hirsuta* | S5 | S5 | S4 | S4 | S4 | S4 | S4 | S4 | 233 | 101,167 | 210 |
| **IRIDACEAE** |  |  |  |  |  |  |  |  |  |  |  |
| *Iris brevicaulis* | S1 | S2 |  |  | S2 | S2 | S2 | S2 | 7 | 5,368 | 7 |
| *Iris cristata* | S5 | S5 | S4 | S5 | S4 | S5 | S4 | S5 | 476 | 122,640 | 401 |
| *Iris fulva* |  | S1 | S2 | S3 | S2 | S3 | S2 | S3 | 11 | 75,806 | 10 |
| *Iris prismatica* | S1 | S1 | S2 | S2 | S2 | S2 | S2 | S2 | 8 | 7,719 | 8 |
| *Iris shrevei* |  | S2 |  |  | S1 | S1 | S1 | S1 | 4 | 3,007 | 4 |
| *Iris verna* | S5 | S4 | S3 | S3 | S3 | S3 | S3 | S3 | 46 | 27,059 | 41 |
| *Iris virginica* | S5 | S4 | S3 | S3 | S3 | S3 | S3 | S3 | 34 | 101,330 | 33 |
| *Sisyrinchium albidum* |  | S5 | S3 | S3 | S3 | S3 | S3 | S3 | 43 | 70,401 | 37 |
| *Sisyrinchium angustifolium* | S5 | S5 | S3 | S4 | S3 | S4 | S3 | S4 | 105 | 121,170 | 104 |
| *Sisyrinchium atlanticum* | S5 | S4 | S2 | S3 | S2 | S3 | S2 | S3 | 14 | 41,755 | 14 |
| *Sisyrinchium langloisii* |  | S1 |  |  |  |  |  |  |  |  |  |
| *Sisyrinchium mucronatum* |  | S4 | S2 | S2 | S2 | S2 | S2 | S2 | 5 | 33,331 | 5 |
| *Sisyrinchium nashii* |  | S3 | S2 | S2 | S2 | S2 | S2 | S2 | 6 | 2,642 | 6 |
| *Sisyrinchium pruinosum* |  | S1 |  |  |  |  |  |  |  |  |  |
| **JUNCACEAE** |  |  |  |  |  |  |  |  |  |  |  |
| *Juncus acuminatus* | S5 | S5 | S3 | S3 | S3 | S3 | S3 | S3 | 22 | 50,033 | 22 |
| *Juncus anthelatus* |  | S5 | S2 | S3 | S2 | S3 | S2 | S3 | 10 | 43,181 | 10 |
| *Juncus articulatus* |  | S1 |  |  |  |  |  |  |  |  |  |
| *Juncus biflorus* | S5 | S5 | S2 | S3 | S2 | S3 | S2 | S3 | 9 | 37,195 | 9 |
| *Juncus brachycarpus* | S4 | S5 | S2 | S3 | S2 | S3 | S2 | S3 | 11 | 33,825 | 11 |
| *Juncus brachyphyllus* |  | S1 |  |  | S1 | S1 | S2 | S1 | 3 | 2,843 | 3 |
| *Juncus brevicaudatus* |  | S1 |  |  |  |  |  |  |  |  |  |
| *Juncus bufonius* |  | S2 | S1 | S1 | S1 | S1 | S1 | S1 | 1 | <1 | 1 |
| *Juncus canadensis* | S3 | S4 | S2 | S3 | S2 | S2 | S2 | S3 | 14 | 21,876 | 14 |
| *Juncus coriaceus* | S5 | S5 | S3 | S3 | S3 | S3 | S3 | S3 | 23 | 79,131 | 23 |
| *Juncus debilis* | S5 | S5 | S2 | S3 | S2 | S2 | S2 | S3 | 12 | 26,240 | 12 |
| *Juncus dichotomus* | S5 | S3 | S1 | S1 | S1 | S1 | S1 | S1 | 2 | 37 | 2 |
| *Juncus diffusissimus* | S5 | S4 | S2 | S2 | S2 | S2 | S2 | S3 | 6 | 19,299 | 6 |
| *Juncus dudleyi* | S1 | S4 | S2 | S3 | S2 | S2 | S2 | S3 | 7 | 20,740 | 7 |
| *Juncus effusus* | S5 | S5 | S3 | S3 | S3 | S3 | S3 | S3 | 57 | 102,651 | 54 |
| *Juncus elliottii* |  | S2 |  |  | S1 | S1 | S1 | S1 | 3 | 90 | 3 |
| *Juncus filipendulus* |  | S4 | S2 | S2 | S2 | S2 | S2 | S2 | 17 | 11,177 | 16 |
| *Juncus gymnocarpus* |  | S2 | S1 | S1 | S1 | S1 | S1 | S1 | 4 | 2,336 | 4 |
| *Juncus interior* | S1 | S5 | S1 | S2 | S1 | S1 | S1 | S2 | 4 | 6,074 | 3 |
| *Juncus longii* | S3 | S3 | S1 | S1 | S1 | S1 | S1 | S1 | 4 | 1,730 | 4 |
| *Juncus marginatus* | S5 | S5 | S2 | S3 | S2 | S3 | S2 | S3 | 12 | 39,405 | 12 |
| *Juncus nodatus* |  | S3 | S1 | S2 | S1 | S2 | S1 | S2 | 4 | 9,678 | 4 |
| *Juncus repens* |  | S3 | S1 | S1 | S1 | S1 | S1 | S1 | 5 | 1,020 | 5 |
| *Juncus scirpoides* | S5 | S4 | S1 | S1 | S1 | S1 | S1 | S1 | 2 | 47 | 2 |
| *Juncus secundus* |  | S4 | S1 | S1 | S1 | S1 | S1 | S1 | 1 | <1 | 1 |
| *Juncus subcaudatus* | S1 | S3 | S1 | S1 | S1 | S1 | S1 | S1 | 2 | 37 | 2 |
| *Juncus tenuis* | S5 | S5 | S3 | S3 | S3 | S3 | S3 | S3 | 35 | 88,949 | 36 |
| *Juncus torreyi* |  | S3 | S1 | S1 | S1 | S1 | S1 | S1 | 2 | 99 | 2 |
| *Juncus tweedyi* |  | S3 | S1 | S2 | S1 | S2 | S1 | S2 | 4 | 13,402 | 4 |
| *Juncus validus* | S1 | S3 | S1 | S1 | S1 | S1 | S1 | S1 | 2 | 71 | 2 |
| *Luzula acuminata* | S5 | S4 | S3 | S3 | S3 | S3 | S3 | S3 | 33 | 38,600 | 29 |
| *Luzula bulbosa* |  | S5 | S2 | S3 | S2 | S3 | S2 | S3 | 6 | 56,156 | 6 |
| *Luzula echinata* | S5 | S5 | S3 | S3 | S3 | S3 | S3 | S3 | 44 | 94,169 | 44 |
| *Luzula multiflora* |  | S5 | S2 | S3 | S2 | S3 | S2 | S3 | 16 | 41,867 | 16 |
| **LILIACEAE** |  |  |  |  |  |  |  |  |  |  |  |
| *Clintonia borealis* | S4 | S2 | S3 | S3 | S3 | S3 | S3 | S3 | 38 | 6,075 | 33 |
| *Clintonia umbellulata* | S5 | S4 | S3 | S4 | S3 | S4 | S3 | S4 | 96 | 31,014 | 83 |
| *Erythronium albidum* | S2 | S4 | S3 | S3 | S3 | S3 | S3 | S3 | 27 | 57,943 | 26 |
| *Erythronium americanum* |  | S5 | S4 | S4 | S4 | S4 | S4 | S4 | 270 | 112,484 | 246 |
| *Erythronium rostratum* |  | S3 |  |  | S3 | S3 | S3 | S3 | 23 | 14,860 | 23 |
| *Erythronium umbilicatum* | S5 | S4 | S3 | S3 | S3 | S3 | S3 | S3 | 37 | 44,969 | 35 |
| *Lilium canadense* |  | S4 | S2 | S3 | S2 | S2 | S2 | S3 | 13 | 21,665 | 13 |
| *Lilium michauxii* | S4 | S3 | S3 | S3 | S3 | S3 | S3 | S3 | 29 | 8,972 | 27 |
| *Lilium michiganense* | S1 | S4 | S2 | S3 | S2 | S2 | S2 | S3 | 11 | 20,008 | 11 |
| *Lilium philadelphicum* | S1 | S2 |  |  | S2 | S2 | S3 | S2 | 9 | 4,291 | 9 |
| *Lilium superbum* |  | S3 | S3 | S3 | S3 | S3 | S3 | S3 | 52 | 31,090 | 52 |
| *Medeola virginiana* |  | S5 | S4 | S4 | S4 | S4 | S4 | S4 | 257 | 103,350 | 213 |
| *Prosartes lanuginosa* | S5 | S4 | S4 | S4 | S4 | S4 | S4 | S4 | 133 | 64,045 | 119 |
| *Prosartes maculata* | S3 | S3 | S3 | S3 | S3 | S3 | S3 | S3 | 49 | 41,566 | 45 |
| *Streptopus amplexifolius* | S1 | S1 |  |  | S1 | S1 | S2 | S2 | 4 | 3,401 | 4 |
| *Streptopus lanceolatus* |  | S1 | S2 | S2 | S2 | S2 | S2 | S2 | 23 | 1,637 | 18 |
| **MELANTHIACEAE** |  |  |  |  |  |  |  |  |  |  |  |
| *Amianthium muscitoxicum* | S4 | S3 | S2 | S3 | S3 | S3 | S2 | S3 | 21 | 43,070 | 20 |
| *Anticlea glauca* |  | S1 | S1 | S1 | S1 | S1 | S1 | S1 | 2 | 1 | 2 |
| *Chamaelirium luteum* |  | S5 | S3 | S4 | S4 | S4 | S4 | S4 | 124 | 104,480 | 119 |
| *Melanthium hybridum* | S1 | S1 |  |  | S3 | S3 | S3 | S3 | 23 | 44,193 | 24 |
| *Melanthium parviflorum* | S4 | S3 | S2 | S2 | S2 | S2 | S2 | S2 | 6 | 3,586 | 6 |
| *Melanthium virginicum* |  | S1 |  |  | S1 | S1 | S1 | S1 | 1 | <1 | 1 |
| *Melanthium woodii* |  | S2 |  |  | S2 | S3 | S2 | S3 | 14 | 33,524 | 13 |
| *Stenanthium gramineum* | S3 | S4 | S3 | S3 | S3 | S3 | S3 | S3 | 36 | 77,567 | 36 |
| *Stenanthium tennesseense* |  | S1 |  |  | S2 | S2 | S2 | S2 | 8 | 911 | 7 |
| *Trillidium undulatum* |  | S1 | S3 | S4 | S3 | S4 | S3 | S4 | 96 | 11,159 | 94 |
| *Trillium catesbaei* |  | S3 | S4 | S4 | S3 | S4 | S4 | S4 | 129 | 16,301 | 114 |
| *Trillium cuneatum* |  | S5 | S4 | S4 | S4 | S5 | S4 | S4 | 321 | 91,621 | 282 |
| *Trillium decumbens* | S3 | S1 | S1 | S1 | S1 | S1 | S1 | S1 | 2 | 5 | 3 |
| *Trillium erectum* | S5 | S4 | S4 | S4 | S4 | S4 | S4 | S4 | 212 | 51,313 | 175 |
| *Trillium flexipes* | S1 | S5 | S3 | S3 | S3 | S3 | S3 | S3 | 56 | 78,109 | 52 |
| *Trillium freemanii* |  |  |  |  |  |  |  |  |  |  |  |
| *Trillium grandiflorum* | S5 | S5 | S4 | S4 | S4 | S4 | S4 | S4 | 197 | 64,178 | 161 |
| *Trillium lancifolium* |  | S1 | S3 | S3 | S3 | S4 | S3 | S3 | 100 | 6,937 | 70 |
| *Trillium luteum* |  | S5 | S4 | S4 | S4 | S5 | S4 | S4 | 336 | 57,505 | 277 |
| *Trillium pusillum* | S2 | S1 |  |  | S2 | S2 | S2 | S2 | 13 | 12,235 | 13 |
| *Trillium radiatum* |  |  |  |  |  |  |  |  |  |  |  |
| *Trillium recurvatum* |  | S5 | S4 | S4 | S4 | S4 | S4 | S4 | 151 | 92,197 | 128 |
| *Trillium rugelii* | S3 | S3 | S3 | S3 | S3 | S3 | S3 | S3 | 21 | 7,908 | 21 |
| *Trillium sessile* |  | S5 | S3 | S3 | S3 | S3 | S3 | S3 | 42 | 26,884 | 34 |
| *Trillium simile* | S2 | S3 | S3 | S3 | S3 | S3 | S3 | S3 | 34 | 15,090 | 30 |
| *Trillium stamineum* |  | S3 | S3 | S3 | S3 | S3 | S3 | S3 | 52 | 46,056 | 47 |
| *Trillium sulcatum* |  | S4 | S3 | S3 | S3 | S3 | S3 | S3 | 58 | 42,457 | 54 |
| *Trillium vaseyi* |  | S3 | S3 | S3 | S3 | S3 | S3 | S3 | 67 | 13,216 | 57 |
| *Veratrum viride* |  | S2 | S3 | S3 | S3 | S3 | S3 | S3 | 26 | 7,293 | 27 |
| *Xerophyllum asphodeloides* | S1 | S2 |  |  | S3 | S3 | S3 | S2 | 26 | 3,894 | 26 |
| **NARTHECIACEAE** |  |  |  |  |  |  |  |  |  |  |  |
| *Aletris farinosa* | S5 | S4 | S3 | S3 | S3 | S3 | S3 | S3 | 40 | 33,465 | 40 |
| **ORCHIDACEAE** |  |  |  |  |  |  |  |  |  |  |  |
| *Aplectrum hyemale* |  | S4 | S4 | S4 | S4 | S4 | S4 | S4 | 216 | 79,287 | 200 |
| *Calopogon tuberosus* |  | S3 | S2 | S3 | S2 | S2 | S2 | S3 | 10 | 21,615 | 10 |
| *Cleistesiopsis bifaria* | S1 | S3 | S3 | S3 | S3 | S3 | S3 | S3 | 40 | 36,543 | 39 |
| *Corallorhiza maculata* |  | S2 |  |  | S1 | S2 | S2 | S2 | 3 | 9 | 3 |
| *Corallorhiza odontorhiza* | S3 | S4 | S3 | S3 | S3 | S3 | S3 | S3 | 46 | 53,891 | 43 |
| *Corallorhiza wisteriana* |  | S4 | S3 | S3 | S3 | S3 | S3 | S3 | 42 | 91,135 | 41 |
| *Cypripedium acaule* |  | S5 | S4 | S5 | S4 | S5 | S4 | S5 | 434 | 63,918 | 402 |
| *Cypripedium kentuckiense* | S1 | S1 | S2 | S3 | S2 | S3 | S2 | S3 | 15 | 34,052 | 15 |
| *Cypripedium parviflorum* |  | S3 | S3 | S4 | S3 | S4 | S3 | S4 | 108 | 66,113 | 102 |
| *Cypripedium pubescens* | S3 | S3 | S1 | S1 | S1 | S1 | S1 | S1 | 1 | <1 | 1 |
| *Cypripedium reginae* | S1 | S1 | S2 | S2 | S2 | S2 | S2 | S2 | 6 | 9,433 | 6 |
| *Dactylorhiza viridis* |  | S1 | S1 | S1 | S1 | S1 | S1 | S1 | 5 | 376 | 5 |
| *Galearis spectabilis* |  | S4 | S4 | S4 | S4 | S4 | S4 | S4 | 212 | 82,387 | 178 |
| *Goodyera pubescens* | S5 | S1 | S4 | S5 | S4 | S5 | S4 | S5 | 513 | 96,814 | 433 |
| *Goodyera repens* | S1 | S2 | S2 | S3 | S2 | S3 | S3 | S3 | 23 | 4,260 | 23 |
| *Hexalectris spicata* |  | S4 |  |  |  |  |  |  |  |  |  |
| *Isotria medeoloides* | S2 | S1 | S1 | S1 | S1 | S1 | S1 | S1 | 3 | 207 | 3 |
| *Isotria verticillata* |  | S4 | S3 | S3 | S3 | S3 | S3 | S3 | 27 | 49,449 | 27 |
| *Liparis liliifolia* | S3 | S4 | S3 | S3 | S3 | S3 | S3 | S3 | 61 | 75,526 | 58 |
| *Liparis loeselii* | S1 | S2 |  |  | S2 | S3 | S2 | S3 | 6 | 13,382 | 6 |
| *Malaxis unifolia* | S4 | S4 | S2 | S3 | S2 | S3 | S2 | S3 | 20 | 68,204 | 20 |
| *Neottia bifolia* | S3 | S1 | S2 | S3 | S2 | S2 | S2 | S3 | 19 | 21,624 | 19 |
| *Neottia smallii* |  | S1 | S2 | S2 | S2 | S2 | S2 | S2 | 7 | 3,181 | 7 |
| *Platanthera ciliaris* |  | S4 | S4 | S4 | S4 | S4 | S4 | S4 | 199 | 64,111 | 194 |
| *Platanthera clavellata* |  | S4 | S3 | S3 | S3 | S3 | S3 | S3 | 71 | 96,179 | 68 |
| *Platanthera cristata* | S3 | S3 |  |  | S3 | S3 | S3 | S3 | 41 | 34,597 | 42 |
| *Platanthera flava* | S3 | S3 | S2 | S3 | S3 | S3 | S2 | S3 | 20 | 60,041 | 20 |
| *Platanthera grandiflora* | S2 | S1 | S2 | S1 | S2 | S1 | S2 | S1 | 7 | 185 | 7 |
| *Platanthera herbiola* | S1 | S2 |  |  | S2 | S3 | S2 | S3 | 14 | 22,720 | 14 |
| *Platanthera integra* | S1 | S1 | S1 | S1 | S1 | S1 | S1 | S1 | 1 | <1 | 1 |
| *Platanthera integrilabia* | S1 | S3 | S3 | S3 | S3 | S3 | S3 | S3 | 36 | 13,849 | 36 |
| *Platanthera lacera* |  | S3 | S3 | S3 | S3 | S3 | S3 | S3 | 41 | 84,654 | 40 |
| *Platanthera nivea* | S2 | S1 | S2 | S2 | S2 | S2 | S2 | S2 | 21 | 484 | 21 |
| *Platanthera orbiculata* |  | S1 | S2 | S1 | S2 | S1 | S2 | S1 | 7 | 453 | 6 |
| *Platanthera peramoena* |  | S4 | S3 | S3 | S3 | S3 | S3 | S3 | 32 | 91,072 | 32 |
| *Platanthera psycodes* | S1 | S1 | S3 | S3 | S2 | S2 | S3 | S3 | 21 | 6,413 | 21 |
| *Pogonia ophioglossoides* | S3 | S2 |  |  | S2 | S2 | S2 | S2 | 8 | 3,019 | 8 |
| *Ponthieva racemosa* | S2 | S1 |  |  | S1 | S1 | S1 | S1 | 2 | 5 | 2 |
| *Spiranthes cernua* | S5 | S5 | S3 | S3 | S3 | S3 | S3 | S3 | 27 | 60,043 | 27 |
| *Spiranthes lacera* |  | S3 | S3 | S4 | S3 | S4 | S3 | S4 | 118 | 98,285 | 112 |
| *Spiranthes lucida* | S1 | S2 | S2 | S3 | S2 | S3 | S2 | S3 | 8 | 27,697 | 8 |
| *Spiranthes magnicamporum* | S1 | S1 | S3 | S3 | S3 | S3 | S3 | S3 | 29 | 18,191 | 28 |
| *Spiranthes ochroleuca* |  | S2 | S1 | S1 | S1 | S1 | S1 | S1 | 2 | 60 | 2 |
| *Spiranthes odorata* |  | S1 | S2 | S2 | S2 | S2 | S2 | S2 | 13 | 14,518 | 13 |
| *Spiranthes ovalis* | S2 | S4 | S3 | S3 | S3 | S3 | S3 | S3 | 41 | 84,344 | 41 |
| *Spiranthes tuberosa* | S4 | S4 | S3 | S3 | S3 | S3 | S3 | S3 | 22 | 66,985 | 22 |
| *Spiranthes vernalis* | S5 | S4 | S3 | S3 | S3 | S3 | S3 | S3 | 76 | 108,891 | 75 |
| *Tipularia discolor* | S5 | S5 | S4 | S5 | S4 | S5 | S4 | S5 | 493 | 123,701 | 436 |
| *Triphora trianthophoros* | S2 | S3 | S3 | S3 | S3 | S3 | S3 | S3 | 28 | 61,514 | 26 |
| **POACEAE** |  |  |  |  |  |  |  |  |  |  |  |
| *Agrostis elliottiana* | S3 | S4 | S1 | S1 | S1 | S1 | S1 | S1 | 2 | 6 | 2 |
| *Agrostis hyemalis* | S5 | S5 | S2 | S3 | S2 | S3 | S2 | S3 | 10 | 29,322 | 10 |
| *Agrostis mertensii* |  | S1 | S1 | S1 | S1 | S1 | S1 | S1 | 1 | <1 | 1 |
| *Agrostis perennans* |  | S5 | S3 | S3 | S3 | S3 | S2 | S3 | 24 | 51,625 | 22 |
| *Agrostis scabra* | S3 | S4 | S2 | S2 | S2 | S2 | S2 | S2 | 6 | 2,215 | 6 |
| *Alopecurus aequalis* |  |  |  |  |  |  |  |  |  |  |  |
| *Alopecurus carolinianus* |  | S5 | S1 | S1 | S1 | S1 | S2 | S2 | 4 | 2,498 | 4 |
| *Andropogon gerardi* | S5 | S5 | S3 | S4 | S3 | S4 | S3 | S4 | 97 | 105,616 | 91 |
| *Andropogon glomeratus* | S5 | S4 | S2 | S3 | S2 | S3 | S2 | S3 | 20 | 44,697 | 18 |
| *Andropogon gyrans* | S5 | S5 | S2 | S3 | S2 | S3 | S2 | S3 | 19 | 36,099 | 18 |
| *Andropogon ternarius* | S5 | S5 | S3 | S3 | S3 | S3 | S3 | S3 | 22 | 91,960 | 22 |
| *Andropogon virginicus* | S5 | S5 | S3 | S3 | S3 | S3 | S3 | S3 | 80 | 111,809 | 76 |
| *Anthoxanthum hirtum* |  | S1 | S1 | S1 | S1 | S1 | S1 | S1 | 1 | <1 | 1 |
| *Aristida curtissii* | S3 | S1 | S1 | S1 | S1 | S1 | S1 | S1 | 1 | <1 | 1 |
| *Aristida dichotoma* | S4 | S5 | S2 | S2 | S2 | S2 | S2 | S2 | 7 | 18,205 | 7 |
| *Aristida geniculata* |  | S1 |  |  |  |  |  |  |  |  |  |
| *Aristida lanosa* | S3 | S1 |  |  |  |  |  |  |  |  |  |
| *Aristida longespica* | S5 | S4 | S1 | S1 | S1 | S2 | S1 | S1 | 2 | 78 | 2 |
| *Aristida oligantha* |  | S5 | S1 | S1 | S1 | S1 | S1 | S1 | 2 | 10 | 2 |
| *Aristida purpurascens* |  | S4 | S2 | S2 | S2 | S2 | S1 | S2 | 6 | 10,588 | 5 |
| *Aristida ramosissima* |  | S1 |  |  |  |  |  |  |  |  |  |
| *Aristida virgata* | S3 | S2 | S1 | S1 | S1 | S1 | S1 | S1 | 1 | <1 | 1 |
| *Arundinaria appalachiana* |  | S2 | S3 | S3 | S3 | S3 | S3 | S3 | 26 | 17,095 | 23 |
| *Arundinaria gigantea* |  | S5 | S4 | S4 | S4 | S4 | S4 | S4 | 166 | 119,899 | 155 |
| *Arundinaria tecta* | S5 | S3 | S1 | S1 | S1 | S1 | S1 | S1 | 1 | <1 | 1 |
| *Avenella flexuosa* |  | S2 | S1 | S2 | S1 | S2 | S1 | S2 | 5 | 9,702 | 5 |
| *Bouteloua curtipendula* |  | S3 | S2 | S3 | S2 | S3 | S2 | S3 | 15 | 32,818 | 13 |
| *Brachyelytrum aristosum* |  | S1 |  |  | S2 | S1 | S2 | S1 | 7 | 739 | 8 |
| *Brachyelytrum erectum* | S5 | S5 | S3 | S3 | S3 | S3 | S3 | S3 | 25 | 66,062 | 24 |
| *Bromus latiglumis* |  | S2 | S1 | S1 | S1 | S1 | S1 | S1 | 1 | <1 | 1 |
| *Bromus nottowayanus* | S3 | S4 | S1 | S1 | S1 | S1 | S1 | S1 | 2 | 1 | 2 |
| *Bromus pubescens* | S5 | S5 | S3 | S3 | S3 | S3 | S3 | S3 | 34 | 80,093 | 31 |
| *Calamagrostis canadensis* |  | S1 | S1 | S1 | S1 | S1 | S1 | S1 | 4 | 1,258 | 4 |
| *Calamagrostis insperata* |  | S1 | S1 | S1 | S1 | S1 | S1 | S1 | 1 | <1 | 1 |
| *Calamovilfa arcuata* | S1 | S1 |  |  |  |  |  |  |  |  |  |
| *Cenchrus longispinus* |  | S2 | S2 | S2 | S2 | S2 | S2 | S2 | 4 | 28,661 | 4 |
| *Cenchrus spinifex* |  | S1 |  |  |  |  |  |  |  |  |  |
| *Chasmanthium latifolium* | S5 | S5 | S4 | S4 | S4 | S4 | S4 | S4 | 178 | 113,432 | 161 |
| *Chasmanthium laxum* | S5 | S5 | S3 | S3 | S3 | S3 | S3 | S3 | 33 | 59,591 | 32 |
| *Chasmanthium sessiliflorum* |  | S4 | S1 | S1 | S1 | S1 | S1 | S1 | 5 | 2,708 | 5 |
| *Cinna arundinacea* | S5 | S5 | S3 | S3 | S3 | S3 | S3 | S3 | 25 | 82,977 | 24 |
| *Cinna latifolia* |  | S2 | S2 | S1 | S2 | S1 | S2 | S1 | 6 | 365 | 6 |
| *Coelorachis rugosa* |  | S1 |  |  |  |  |  |  |  |  |  |
| *Coleataenia anceps* | S5 | S4 | S3 | S3 | S3 | S3 | S3 | S3 | 46 | 100,788 | 43 |
| *Coleataenia longifolia* |  | S3 | S1 | S1 | S1 | S1 | S1 | S1 | 1 | <1 | 1 |
| *Coleataenia pulchra* | S5 | S3 | S1 | S1 | S1 | S1 | S1 | S1 | 2 | 20 | 2 |
| *Coleataenia rigidula* |  | S4 | S2 | S3 | S2 | S3 | S2 | S3 | 10 | 55,283 | 10 |
| *Danthonia compressa* | S3 | S4 | S2 | S2 | S2 | S2 | S2 | S2 | 10 | 15,392 | 9 |
| *Danthonia epilis* | S3 | S2 | S1 | S1 | S1 | S1 | S1 | S1 | 2 | 1 | 2 |
| *Danthonia sericea* | S5 | S4 | S3 | S3 | S3 | S3 | S3 | S3 | 26 | 56,450 | 26 |
| *Danthonia spicata* | S5 | S5 | S3 | S3 | S3 | S3 | S3 | S3 | 47 | 79,097 | 46 |
| *Diarrhena americana* | S1 | S4 | S2 | S2 | S2 | S2 | S2 | S2 | 11 | 10,833 | 11 |
| *Diarrhena obovata* |  | S2 |  |  | S2 | S2 | S2 | S2 | 8 | 4,855 | 8 |
| *Dichanthelium aciculare* |  | S3 | S1 | S2 | S1 | S2 | S1 | S2 | 5 | 15,529 | 5 |
| *Dichanthelium acuminatum* |  | S5 | S3 | S3 | S3 | S3 | S2 | S3 | 40 | 103,134 | 39 |
| *Dichanthelium angustifolium* |  | S4 | S2 | S3 | S2 | S3 | S2 | S3 | 6 | 24,497 | 6 |
| *Dichanthelium annulum* | S1 | S1 | S1 | S1 | S1 | S1 | S1 | S1 | 1 | <1 | 1 |
| *Dichanthelium boscii* | S5 | S5 | S3 | S3 | S3 | S3 | S3 | S3 | 60 | 86,615 | 58 |
| *Dichanthelium clandestinum* |  | S5 | S3 | S3 | S3 | S3 | S3 | S3 | 45 | 76,376 | 43 |
| *Dichanthelium columbianum* |  | S1 | S1 | S1 | S1 | S1 | S1 | S1 | 1 | <1 | 1 |
| *Dichanthelium commutatum* | S5 | S5 | S3 | S3 | S3 | S3 | S3 | S3 | 54 | 70,213 | 48 |
| *Dichanthelium consanguineum* |  | S1 | S1 | S1 | S1 | S1 | S1 | S1 | 3 | 134 | 3 |
| *Dichanthelium curtifolium* | S1 | S1 | S1 | S1 | S1 | S1 | S1 | S1 | 1 | <1 | 1 |
| *Dichanthelium depauperatum* |  | S5 | S2 | S3 | S2 | S3 | S2 | S3 | 18 | 30,295 | 18 |
| *Dichanthelium dichotomum* |  | S5 | S3 | S3 | S3 | S3 | S3 | S3 | 46 | 97,650 | 41 |
| *Dichanthelium ensifolium* |  | S3 | S1 | S1 | S1 | S1 | S1 | S1 | 2 | 81 | 2 |
| *Dichanthelium latifolium* |  | S3 | S3 | S3 | S3 | S3 | S3 | S3 | 21 | 53,073 | 23 |
| *Dichanthelium laxiflorum* | S5 | S5 | S3 | S3 | S3 | S3 | S3 | S3 | 50 | 98,773 | 45 |
| *Dichanthelium leucothrix* |  | S3 | S1 | S1 | S1 | S1 | S1 | S1 | 1 | <1 | 1 |
| *Dichanthelium linearifolium* |  | S4 | S2 | S2 | S2 | S2 | S2 | S2 | 6 | 5,836 | 6 |
| *Dichanthelium longiligulatum* | S4 | S4 | S1 | S1 | S1 | S1 | S1 | S1 | 4 | 3,498 | 4 |
| *Dichanthelium lucidum* |  | S3 | S1 | S1 | S1 | S1 | S1 | S1 | 4 | 4,017 | 4 |
| *Dichanthelium malacophyllum* |  | S4 | S2 | S2 | S2 | S2 | S2 | S3 | 9 | 18,432 | 9 |
| *Dichanthelium meridionale* |  | S4 | S1 | S2 | S1 | S2 | S1 | S2 | 3 | 9,445 | 3 |
| *Dichanthelium microcarpon* |  | S4 | S3 | S3 | S3 | S3 | S3 | S3 | 26 | 82,468 | 26 |
| *Dichanthelium oligosanthes* |  | S3 | S2 | S2 | S2 | S2 | S2 | S2 | 8 | 13,832 | 8 |
| *Dichanthelium ovale* |  | S2 | S1 | S1 | S1 | S1 | S1 | S1 | 2 | <1 | 2 |
| *Dichanthelium polyanthes* |  | S5 | S3 | S3 | S3 | S3 | S3 | S3 | 40 | 75,078 | 36 |
| *Dichanthelium ravenelii* | S3 | S3 | S2 | S2 | S2 | S2 | S2 | S2 | 6 | 12,216 | 6 |
| *Dichanthelium roanokense* |  | S1 | S1 | S1 | S1 | S1 | S1 | S1 | 1 | <1 | 1 |
| *Dichanthelium scabriusculum* |  | S3 |  |  |  |  |  |  |  |  |  |
| *Dichanthelium scoparium* | S5 | S5 | S3 | S3 | S3 | S3 | S3 | S3 | 39 | 88,032 | 38 |
| *Dichanthelium scribnerianum* |  | S1 | S1 | S1 | S1 | S1 | S1 | S1 | 3 | 315 | 3 |
| *Dichanthelium sphaerocarpon* |  | S5 | S2 | S3 | S2 | S3 | S2 | S3 | 19 | 43,219 | 19 |
| *Dichanthelium spretum* | S1 | S1 |  |  | S1 | S1 | S1 | S1 | 2 | <1 | 2 |
| *Dichanthelium strigosum* |  | S2 | S1 | S1 | S1 | S1 | S1 | S1 | 2 | 7 | 2 |
| *Dichanthelium tenue* | S3 | S3 | S1 | S1 | S1 | S1 | S1 | S1 | 3 | 1,778 | 3 |
| *Dichanthelium villosissimum* | S4 | S5 | S2 | S3 | S2 | S3 | S2 | S3 | 14 | 60,589 | 13 |
| *Dichanthelium wrightianum* |  | S1 |  |  | S1 | S1 | S1 | S1 | 2 | 2 | 2 |
| *Dichanthelium yadkinense* |  | S3 | S2 | S2 | S2 | S2 | S2 | S3 | 7 | 16,309 | 7 |
| *Digitaria ciliaris* |  | S4 | S2 | S3 | S2 | S2 | S2 | S3 | 6 | 20,947 | 6 |
| *Digitaria filiformis* | S4 | S4 | S1 | S1 | S1 | S1 | S1 | S1 | 1 | <1 | 1 |
| *Dinebra panicea* |  | S3 | S1 | S2 | S2 | S3 | S2 | S2 | 1 | <1 | 1 |
| *Dinebra panicoides* | S3 | S2 | S1 | S1 | S1 | S1 | S1 | S1 | 1 | <1 | 1 |
| *Diplachne fascicularis* |  | S1 | S1 | S1 | S1 | S1 | S1 | S1 | 1 | <1 | 1 |
| *Diplachne uninervia* |  | S1 |  |  |  |  |  |  |  |  |  |
| *Echinochloa muricata* |  | S5 | S2 | S3 | S2 | S3 | S2 | S3 | 11 | 51,806 | 10 |
| *Echinochloa walteri* | S1 | S1 | S1 | S1 | S1 | S1 | S1 | S1 | 2 | 27 | 2 |
| *Elymus canadensis* |  | S2 | S1 | S1 | S1 | S1 | S1 | S1 | 2 | 8 | 2 |
| *Elymus curvatus* |  | S1 | S1 | S1 | S1 | S1 | S1 | S1 | 2 | 8 | 2 |
| *Elymus glabriflorus* |  | S4 | S1 | S1 | S1 | S1 | S1 | S1 | 2 | 16 | 2 |
| *Elymus hirsutiglumis* |  | S1 |  |  |  |  |  |  |  |  |  |
| *Elymus hystrix* |  | S5 | S3 | S4 | S3 | S4 | S3 | S4 | 100 | 84,746 | 90 |
| *Elymus macgregorii* |  | S4 | S2 | S3 | S2 | S3 | S2 | S3 | 17 | 54,045 | 16 |
| *Elymus riparius* |  | S4 | S2 | S3 | S2 | S2 | S2 | S3 | 6 | 24,090 | 6 |
| *Elymus svensonii* |  | S2 | S2 | S2 | S2 | S2 | S2 | S2 | 8 | 9,159 | 7 |
| *Elymus villosus* | S2 | S5 | S2 | S2 | S2 | S2 | S2 | S2 | 12 | 17,572 | 12 |
| *Elymus virginicus* | S5 | S5 | S3 | S3 | S3 | S3 | S3 | S3 | 56 | 101,689 | 54 |
| *Eragrostis capillaris* | S4 | S4 | S1 | S1 | S1 | S1 | S1 | S1 | 3 | 764 | 3 |
| *Eragrostis frankii* |  | S4 | S1 | S1 | S1 | S1 | S1 | S1 | 3 | 2,765 | 3 |
| *Eragrostis hirsuta* | S5 | S4 | S2 | S2 | S2 | S2 | S2 | S2 | 4 | 41,482 | 4 |
| *Eragrostis hypnoides* | S5 | S5 | S2 | S2 | S1 | S2 | S2 | S2 | 3 | 20,380 | 3 |
| *Eragrostis intermedia* |  | S1 | S1 | S1 | S1 | S1 | S1 | S1 | 1 | <1 | 1 |
| *Eragrostis lugens* |  | S1 |  |  |  |  |  |  |  |  |  |
| *Eragrostis pectinacea* |  | S5 | S2 | S2 | S2 | S2 | S2 | S2 | 4 | 27,369 | 4 |
| *Eragrostis reptans* |  | S1 |  |  |  |  |  |  |  |  |  |
| *Eragrostis spectabilis* | S5 | S5 | S2 | S3 | S2 | S3 | S2 | S3 | 16 | 70,493 | 15 |
| *Eragrostis trichodes* |  | S1 |  |  |  |  |  |  |  |  |  |
| *Erianthus alopecuroides* |  | S5 | S2 | S3 | S2 | S3 | S2 | S3 | 17 | 77,944 | 17 |
| *Erianthus contortus* |  | S2 | S1 | S1 | S1 | S1 | S1 | S1 | 1 | <1 | 1 |
| *Erianthus giganteus* | S4 | S4 | S2 | S3 | S2 | S3 | S2 | S3 | 18 | 74,823 | 17 |
| *Erianthus strictus* | S3 | S3 | S1 | S1 | S1 | S1 | S1 | S1 | 3 | 5 | 3 |
| *Eriochloa acuminata* |  | S1 | S1 | S1 | S1 | S1 | S1 | S1 | 3 | 2 | 3 |
| *Eriochloa contracta* |  | S1 | S1 | S1 | S1 | S1 | S1 | S1 | 1 | <1 | 1 |
| *Festuca paradoxa* |  | S1 | S1 | S2 | S1 | S2 | S1 | S2 | 4 | 6,627 | 4 |
| *Festuca subverticillata* | S5 | S5 | S3 | S3 | S3 | S3 | S3 | S3 | 22 | 75,730 | 21 |
| *Glyceria acutiflora* | S1 | S3 |  |  | S2 | S2 | S3 | S2 | 15 | 8,422 | 16 |
| *Glyceria arkansana* | S2 | S1 |  |  |  |  |  |  |  |  |  |
| *Glyceria canadensis* |  | S1 | S1 | S1 | S1 | S1 | S1 | S1 | 1 | <1 | 1 |
| *Glyceria laxa* |  | S1 |  |  | S1 | S1 | S1 | S1 | 1 | <1 | 2 |
| *Glyceria melicaria* | S3 | S3 | S2 | S2 | S2 | S2 | S2 | S2 | 7 | 3,023 | 7 |
| *Glyceria nubigena* |  | S1 | S1 | S1 | S1 | S1 | S1 | S1 | 1 | <1 | 1 |
| *Glyceria septentrionalis* | S3 | S3 | S1 | S1 | S1 | S1 | S1 | S1 | 3 | 3,303 | 3 |
| *Glyceria striata* | S5 | S5 | S3 | S3 | S3 | S3 | S3 | S3 | 29 | 77,914 | 28 |
| *Greeneochloa coarctata* |  | S2 | S2 | S2 | S2 | S2 | S2 | S2 | 14 | 12,799 | 14 |
| *Gymnopogon ambiguus* | S4 | S3 | S2 | S2 | S2 | S2 | S2 | S2 | 7 | 4,480 | 7 |
| *Gymnopogon brevifolius* | S2 | S1 |  |  | S2 | S1 | S2 | S1 | 8 | 126 | 9 |
| *Hordeum pusillum* | S5 | S5 | S3 | S3 | S3 | S3 | S3 | S3 | 25 | 48,794 | 24 |
| *Hymenachne hemitoma* | S2 | S1 | S1 | S1 | S1 | S1 | S1 | S1 | 3 | 23 | 3 |
| *Kellochloa verrucosa* | S4 | S3 | S2 | S3 | S2 | S3 | S2 | S3 | 9 | 28,323 | 9 |
| *Leersia hexandra* |  | S2 | S1 | S1 | S1 | S1 | S1 | S1 | 3 | 879 | 3 |
| *Leersia lenticularis* | S4 | S3 | S1 | S2 | S1 | S2 | S2 | S2 | 5 | 18,327 | 5 |
| *Leersia oryzoides* | S5 | S5 | S2 | S3 | S2 | S3 | S2 | S3 | 14 | 62,115 | 14 |
| *Leersia virginica* | S5 | S5 | S2 | S3 | S3 | S3 | S2 | S3 | 21 | 43,045 | 19 |
| *Leptoloma cognatum* |  | S1 |  |  |  |  |  |  |  |  |  |
| *Melica mutica* | S5 | S5 | S3 | S3 | S3 | S3 | S3 | S3 | 53 | 76,414 | 50 |
| *Melica nitens* | S1 | S1 | S1 | S1 | S1 | S1 | S1 | S1 | 2 | 1 | 2 |
| *Milium effusum* |  | S1 | S1 | S1 | S1 | S1 | S1 | S1 | 2 | <1 | 1 |
| *Muhlenbergia bushii* | S1 | S1 |  |  |  |  |  |  |  |  |  |
| *Muhlenbergia capillaris* |  | S3 | S2 | S2 | S2 | S2 | S2 | S2 | 7 | 14,804 | 7 |
| *Muhlenbergia cuspidata* | S2 | S1 | S1 | S1 | S1 | S1 | S1 | S1 | 1 | <1 | 1 |
| *Muhlenbergia frondosa* |  | S4 | S2 | S3 | S2 | S3 | S2 | S3 | 7 | 24,977 | 7 |
| *Muhlenbergia glabrifloris* | S1 | S1 |  |  | S1 | S1 | S1 | S1 | 1 | <1 | 1 |
| *Muhlenbergia schreberi* | S5 | S5 | S2 | S3 | S2 | S3 | S2 | S3 | 15 | 54,656 | 15 |
| *Muhlenbergia sobolifera* | S1 | S4 | S2 | S3 | S2 | S3 | S2 | S3 | 6 | 30,939 | 6 |
| *Muhlenbergia sylvatica* | S1 | S4 | S2 | S3 | S2 | S2 | S2 | S3 | 6 | 20,488 | 6 |
| *Muhlenbergia tenuiflora* |  | S4 | S2 | S3 | S3 | S3 | S3 | S3 | 22 | 49,874 | 20 |
| *Muhlenbergia torreyana* |  | S1 | S1 | S1 | S1 | S1 | S1 | S1 | 1 | <1 | 1 |
| *Panicum capillare* | S5 | S5 | S2 | S3 | S3 | S3 | S2 | S3 | 20 | 44,868 | 20 |
| *Panicum dichotomiflorum* | S5 | S5 | S2 | S3 | S2 | S3 | S2 | S3 | 20 | 86,295 | 19 |
| *Panicum flexile* |  | S5 | S3 | S3 | S3 | S3 | S3 | S3 | 22 | 40,286 | 22 |
| *Panicum philadelphicum* |  | S4 | S2 | S3 | S2 | S3 | S2 | S3 | 10 | 45,398 | 10 |
| *Panicum virgatum* | S5 | S4 | S3 | S3 | S3 | S3 | S3 | S3 | 30 | 74,409 | 28 |
| *Paspalum bifidum* |  | S1 |  |  |  |  |  |  |  |  |  |
| *Paspalum boscianum* |  | S3 |  |  |  |  |  |  |  |  |  |
| *Paspalum dissectum* |  | S2 | S1 | S1 | S1 | S1 | S1 | S1 | 2 | <1 | 2 |
| *Paspalum distichum* |  | S2 | S1 | S1 | S1 | S1 | S1 | S1 | 1 | <1 | 1 |
| *Paspalum floridanum* | S5 | S4 | S2 | S3 | S2 | S3 | S2 | S3 | 11 | 35,388 | 11 |
| *Paspalum fluitans* | S4 | S3 | S1 | S1 | S1 | S1 | S1 | S1 | 2 | <1 | 2 |
| *Paspalum laeve* | S4 | S5 | S2 | S3 | S2 | S3 | S2 | S3 | 15 | 48,502 | 15 |
| *Paspalum pubiflorum* |  | S4 | S2 | S3 | S2 | S3 | S2 | S3 | 7 | 31,597 | 7 |
| *Paspalum setaceum* | S5 | S4 | S2 | S3 | S2 | S3 | S2 | S3 | 11 | 32,553 | 11 |
| *Patis racemosa* |  | S1 |  |  |  |  |  |  |  |  |  |
| *Phalaris arundinacea* | S5 | S4 | S2 | S2 | S2 | S2 | S2 | S3 | 13 | 19,715 | 13 |
| *Phalaris caroliniana* | S2 | S1 | S1 | S1 | S1 | S1 | S1 | S1 | 1 | <1 | 1 |
| *Phanopyrum gymnocarpon* |  | S1 | S1 | S1 | S1 | S1 | S1 | S1 | 2 | 5 | 2 |
| *Piptochaetium avenaceum* |  | S4 | S3 | S3 | S3 | S3 | S3 | S3 | 25 | 53,397 | 24 |
| *Poa alsodes* | S4 | S4 | S2 | S2 | S2 | S3 | S3 | S3 | 22 | 4,375 | 19 |
| *Poa autumnalis* | S5 | S5 | S2 | S2 | S2 | S2 | S2 | S2 | 8 | 7,440 | 8 |
| *Poa chapmaniana* |  | S4 | S1 | S1 | S1 | S1 | S1 | S1 | 1 | <1 | 1 |
| *Poa cuspidata* | S5 | S5 | S2 | S2 | S2 | S2 | S2 | S2 | 17 | 8,066 | 13 |
| *Poa palustris* | S1 | S1 |  |  | S1 | S1 | S2 | S1 | 4 | 2,377 | 4 |
| *Poa saltuensis* |  | S1 |  |  | S1 | S1 | S2 | S2 | 4 | 4,283 | 4 |
| *Poa sylvestris* | S5 | S5 | S2 | S3 | S2 | S3 | S2 | S3 | 11 | 35,419 | 11 |
| *Poa wolfii* | S1 | S1 | S1 | S1 | S1 | S1 | S1 | S1 | 1 | <1 | 1 |
| *Sacciolepis striata* |  | S2 |  |  | S1 | S1 | S2 | S2 | 5 | 3,966 | 5 |
| *Schizachyrium scoparium* |  | S5 | S3 | S3 | S3 | S3 | S3 | S3 | 76 | 103,605 | 74 |
| *Setaria parviflora* | S5 | S5 | S3 | S3 | S3 | S3 | S3 | S3 | 33 | 79,240 | 31 |
| *Sorghastrum elliottii* | S4 | S3 | S2 | S3 | S2 | S2 | S2 | S3 | 6 | 24,605 | 6 |
| *Sorghastrum nutans* | S5 | S5 | S3 | S4 | S3 | S4 | S3 | S4 | 94 | 113,314 | 90 |
| *Spartina pectinata* |  | S3 | S1 | S1 | S1 | S1 | S1 | S1 | 3 | 6 | 3 |
| *Sphenopholis × pallens* |  | S1 |  |  |  |  |  |  |  |  |  |
| *Sphenopholis filiformis* | S2 | S1 |  |  |  |  |  |  |  |  |  |
| *Sphenopholis intermedia* |  | S4 | S2 | S3 | S2 | S3 | S2 | S3 | 7 | 28,127 | 7 |
| *Sphenopholis nitida* | S4 | S5 | S2 | S3 | S2 | S3 | S2 | S3 | 11 | 44,688 | 11 |
| *Sphenopholis obtusata* | S5 | S5 | S2 | S3 | S2 | S3 | S2 | S3 | 10 | 37,324 | 10 |
| *Sphenopholis pensylvanica* | S1 | S4 | S2 | S1 | S2 | S1 | S2 | S1 | 7 | 700 | 6 |
| *Sporobolus clandestinus* | S4 | S4 | S2 | S2 | S2 | S2 | S2 | S2 | 4 | 25,165 | 4 |
| *Sporobolus compositus* |  | S3 | S1 | S1 | S1 | S2 | S1 | S1 | 3 | 1,011 | 3 |
| *Sporobolus cryptandrus* |  | S1 |  |  |  |  |  |  |  |  |  |
| *Sporobolus heterolepis* | S1 | S1 | S2 | S2 | S2 | S1 | S2 | S2 | 6 | 1,040 | 6 |
| *Sporobolus junceus* | S1 | S1 |  |  | S1 | S1 | S1 | S1 | 1 | <1 | 1 |
| *Sporobolus neglectus* |  | S4 | S1 | S2 | S1 | S2 | S1 | S2 | 5 | 14,069 | 5 |
| *Sporobolus ozarkanus* |  | S4 | S1 | S2 | S1 | S2 | S1 | S2 | 3 | 7,310 | 3 |
| *Sporobolus vaginiflorus* |  | S5 | S2 | S3 | S2 | S3 | S2 | S3 | 10 | 38,045 | 10 |
| *Steinchisma hians* |  | S2 | S1 | S1 | S1 | S1 | S1 | S1 | 1 | <1 | 1 |
| *Torreyochloa pallida* | S1 | S1 |  |  | S1 | S1 | S1 | S1 | 2 | 222 | 2 |
| *Tridens × oklahomensis* |  | S1 | S1 | S1 | S1 | S1 | S1 | S1 | 1 | <1 | 1 |
| *Tridens chapmanii* | S1 | S1 | S1 | S1 | S1 | S1 | S1 | S1 | 2 | 20 | 2 |
| *Tridens flavus* | S5 | S5 | S3 | S3 | S3 | S3 | S3 | S3 | 80 | 103,258 | 78 |
| *Tridens strictus* |  | S3 | S2 | S2 | S2 | S2 | S2 | S3 | 7 | 19,528 | 6 |
| *Triplasis purpurea* |  | S1 |  |  |  |  |  |  |  |  |  |
| *Tripsacum dactyloides* |  | S5 | S3 | S3 | S3 | S3 | S3 | S3 | 31 | 75,974 | 30 |
| *Urochloa platyphylla* |  | S3 |  |  | S1 | S2 | S2 | S2 | 4 | 16,719 | 4 |
| *Urochloa texana* |  | S1 |  |  |  |  |  |  |  |  |  |
| *Vulpia octoflora* |  | S3 | S1 | S1 | S1 | S2 | S1 | S1 | 3 | 326 | 3 |
| *Zizaniopsis miliacea* |  | S3 | S2 | S2 | S2 | S2 | S2 | S2 | 10 | 4,491 | 9 |
| **PONTEDERIACEAE** |  |  |  |  |  |  |  |  |  |  |  |
| *Heteranthera dubia* |  | S4 | S2 | S3 | S2 | S3 | S2 | S3 | 13 | 68,140 | 13 |
| *Heteranthera limosa* |  | S3 |  |  | S2 | S3 | S2 | S3 | 6 | 2,849 | 5 |
| *Heteranthera missouriensis* |  | S1 | S1 | S1 | S1 | S1 | S1 | S1 | 1 | <1 | 1 |
| *Heteranthera reniformis* | S4 | S4 | S2 | S3 | S2 | S3 | S2 | S3 | 11 | 39,439 | 11 |
| *Pontederia cordata* |  | S3 | S3 | S3 | S3 | S3 | S3 | S3 | 53 | 108,034 | 49 |
| **POTAMOGETONACEAE** |  |  |  |  |  |  |  |  |  |  |  |
| *Potamogeton amplifolius* | S1 | S2 |  |  | S2 | S2 | S2 | S2 | 8 | 2,105 | 8 |
| *Potamogeton berchtoldii* |  | S1 |  |  |  |  |  |  |  |  |  |
| *Potamogeton diversifolius* | S5 | S5 | S2 | S3 | S2 | S3 | S2 | S3 | 16 | 45,263 | 16 |
| *Potamogeton epihydrus* |  | S3 |  |  | S2 | S2 | S3 | S3 | 10 | 17,548 | 9 |
| *Potamogeton foliosus* |  | S4 | S2 | S2 | S2 | S2 | S2 | S2 | 5 | 28,834 | 5 |
| *Potamogeton illinoensis* |  | S1 |  |  |  |  |  |  |  |  |  |
| *Potamogeton nodosus* |  | S4 | S3 | S3 | S3 | S3 | S3 | S3 | 22 | 77,252 | 22 |
| *Potamogeton pulcher* |  | S3 | S1 | S1 | S1 | S1 | S1 | S1 | 1 | <1 | 1 |
| *Potamogeton pusillus* | S4 | S3 | S1 | S1 | S1 | S1 | S1 | S1 | 2 | 88 | 2 |
| *Potamogeton tennesseensis* | S1 | S2 |  |  | S2 | S2 | S3 | S2 | 14 | 7,908 | 14 |
| *Stuckenia pectinata* |  | S2 | S1 | S1 | S1 | S2 | S1 | S1 | 3 | 173 | 3 |
| *Zannichellia palustris* | S2 | S3 | S1 | S1 | S1 | S1 | S1 | S1 | 2 | 5 | 2 |
| **RUSCACEAE** |  |  |  |  |  |  |  |  |  |  |  |
| *Convallaria pseudomajalis* |  | S2 | S2 | S2 | S2 | S2 | S2 | S3 | 11 | 16,713 | 11 |
| *Maianthemum canadense* | S5 | S3 | S3 | S3 | S3 | S3 | S3 | S3 | 37 | 14,410 | 34 |
| *Maianthemum racemosum* | S5 | S5 | S4 | S5 | S4 | S5 | S4 | S5 | 471 | 121,787 | 402 |
| *Maianthemum stellatum* | S1 | S1 |  |  | S1 | S1 | S1 | S1 | 2 | 14 | 2 |
| *Polygonatum biflorum* | S5 | S5 | S4 | S4 | S4 | S5 | S4 | S5 | 327 | 127,963 | 289 |
| *Polygonatum pubescens* | S5 | S4 | S3 | S3 | S3 | S3 | S3 | S3 | 58 | 38,003 | 53 |
| **SMILACACEAE** |  |  |  |  |  |  |  |  |  |  |  |
| *Smilax bona-nox* | S5 | S5 | S4 | S4 | S4 | S4 | S4 | S4 | 302 | 113,215 | 284 |
| *Smilax ecirrata* |  | S3 | S2 | S2 | S2 | S2 | S2 | S2 | 9 | 11,753 | 8 |
| *Smilax glauca* | S5 | S5 | S4 | S4 | S4 | S4 | S4 | S4 | 192 | 120,570 | 175 |
| *Smilax herbacea* | S5 | S4 | S3 | S3 | S3 | S3 | S3 | S3 | 28 | 55,971 | 28 |
| *Smilax hispida* | S5 | S5 | S3 | S3 | S3 | S3 | S3 | S3 | 79 | 115,341 | 75 |
| *Smilax hugeri* |  | S4 | S2 | S3 | S2 | S2 | S2 | S3 | 11 | 23,917 | 11 |
| *Smilax lasioneura* |  | S2 | S1 | S1 | S1 | S1 | S1 | S1 | 2 | 64 | 2 |
| *Smilax laurifolia* | S5 | S1 | S1 | S1 | S1 | S1 | S1 | S1 | 1 | <1 | 1 |
| *Smilax pulverulenta* |  | S3 | S1 | S2 | S2 | S2 | S2 | S2 | 5 | 13,953 | 5 |
| *Smilax rotundifolia* | S5 | S5 | S4 | S4 | S4 | S4 | S4 | S4 | 215 | 116,187 | 198 |
| *Smilax walteri* | S5 | S1 | S1 | S1 | S1 | S1 | S1 | S1 | 4 | 1,715 | 4 |
| **SPARGANIACEAE** |  |  |  |  |  |  |  |  |  |  |  |
| *Sparganium americanum* | S5 | S5 | S3 | S3 | S3 | S3 | S3 | S3 | 25 | 83,081 | 25 |
| *Sparganium androcladum* |  | S1 |  |  | S1 | S1 | S2 | S1 | 4 | 1,104 | 4 |
| *Sparganium eurycarpum* |  | S1 |  |  |  |  |  |  |  |  |  |
| **TOFIELDIACEAE** |  |  |  |  |  |  |  |  |  |  |  |
| *Triantha glutinosa* | S1 | S1 | S1 | S1 | S1 | S1 | S1 | S1 | 2 | 49 | 2 |
| *Triantha racemosa* | S4 | S1 | S1 | S1 | S1 | S1 | S1 | S1 | 1 | <1 | 1 |
| **TYPHACEAE** |  |  |  |  |  |  |  |  |  |  |  |
| *Typha angustifolia* |  | S4 | S2 | S3 | S2 | S3 | S2 | S3 | 11 | 36,959 | 11 |
| *Typha latifolia* | S5 | S5 | S3 | S3 | S3 | S3 | S3 | S3 | 48 | 111,885 | 48 |
| **XYRIDACEAE** |  |  |  |  |  |  |  |  |  |  |  |
| *Xyris ambigua* | S4 | S1 |  |  | S1 | S1 | S1 | S1 | 2 | 5 | 2 |
| *Xyris caroliniana* |  | S1 |  |  |  |  |  |  |  |  |  |
| *Xyris curtissii* | S1 | S1 |  |  |  |  |  |  |  |  |  |
| *Xyris difformis* |  | S2 | S1 | S1 | S1 | S1 | S1 | S1 | 5 | 1,567 | 5 |
| *Xyris fimbriata* |  | S1 |  |  | S1 | S1 | S1 | S1 | 1 | <1 | 1 |
| *Xyris iridifolia* |  | S1 | S1 | S1 | S1 | S1 | S1 | S1 | 1 | <1 | 1 |
| *Xyris jupicai* |  | S2 | S1 | S1 | S1 | S1 | S1 | S1 | 2 | 13 | 2 |
| *Xyris tennesseensis* | S1 | S1 | S2 | S1 | S2 | S1 | S2 | S2 | 15 | 845 | 14 |
| *Xyris torta* |  | S4 | S3 | S3 | S3 | S3 | S3 | S3 | 27 | 39,235 | 26 |
| **ACANTHACEAE** |  |  |  |  |  |  |  |  |  |  |  |
| *Dicliptera brachiata* |  | S4 | S3 | S3 | S3 | S3 | S3 | S3 | 23 | 52,013 | 22 |
| *Justicia americana* | S5 | S5 | S4 | S4 | S4 | S4 | S4 | S4 | 145 | 98,820 | 142 |
| *Justicia lanceolata* |  | S1 | S1 | S1 | S1 | S1 | S1 | S1 | 2 | <1 | 2 |
| *Ruellia caroliniensis* | S5 | S5 | S4 | S4 | S4 | S4 | S4 | S4 | 267 | 122,738 | 240 |
| *Ruellia humilis* | S3 | S4 | S3 | S3 | S3 | S3 | S3 | S3 | 69 | 71,065 | 65 |
| *Ruellia purshiana* | S2 | S4 | S2 | S2 | S2 | S2 | S2 | S2 | 12 | 5,348 | 11 |
| *Ruellia strepens* |  | S5 | S3 | S4 | S3 | S4 | S3 | S4 | 98 | 111,524 | 94 |
| **ALTINGIACEAE** |  |  |  |  |  |  |  |  |  |  |  |
| *Liquidambar styraciflua* | S5 | S5 | S4 | S5 | S4 | S5 | S4 | S5 | 766 | 131,028 | 677 |
| **AMARANTHACEAE** |  |  |  |  |  |  |  |  |  |  |  |
| *Amaranthus albus* |  | S3 | S2 | S3 | S2 | S2 | S2 | S3 | 7 | 22,526 | 6 |
| *Amaranthus arenicola* |  | S1 |  |  |  |  |  |  |  |  |  |
| *Amaranthus tuberculatus* |  | S3 | S1 | S2 | S1 | S2 | S2 | S2 | 5 | 7,309 | 5 |
| *Chenopodiastrum simplex* |  | S1 |  |  |  |  |  |  |  |  |  |
| *Chenopodium berlandieri* |  | S1 |  |  |  |  |  |  |  |  |  |
| *Chenopodium standleyanum* |  | S2 | S1 | S1 | S1 | S1 | S1 | S1 | 1 | <1 | 1 |
| *Cycloloma atriplicifolium* |  | S1 |  |  |  |  |  |  |  |  |  |
| *Froelichia floridana* |  | S1 | S1 | S1 | S1 | S1 | S1 | S1 | 1 | <1 | 1 |
| *Froelichia gracilis* |  | S3 | S1 | S1 | S1 | S1 | S1 | S1 | 2 | 5 | 2 |
| *Iresine rhizomatosa* | S3 | S3 | S1 | S2 | S1 | S2 | S2 | S2 | 3 | 19,031 | 3 |
| **ANACARDIACEAE** |  |  |  |  |  |  |  |  |  |  |  |
| *Cotinus obovatus* |  | S3 | S2 | S3 | S2 | S3 | S2 | S3 | 21 | 52,573 | 19 |
| *Rhus aromatica* | S3 | S5 | S3 | S3 | S3 | S4 | S3 | S4 | 86 | 54,984 | 80 |
| *Rhus copallinum* | S5 | S5 | S4 | S5 | S4 | S5 | S4 | S5 | 409 | 123,145 | 387 |
| *Rhus glabra* | S5 | S5 | S4 | S4 | S4 | S4 | S4 | S4 | 223 | 125,911 | 218 |
| *Rhus typhina* |  | S4 | S3 | S3 | S3 | S3 | S3 | S3 | 54 | 78,849 | 51 |
| *Toxicodendron pubescens* | S5 | S3 | S2 | S3 | S2 | S3 | S2 | S3 | 7 | 36,857 | 7 |
| *Toxicodendron radicans* | S5 | S5 | S4 | S5 | S4 | S5 | S4 | S5 | 895 | 132,715 | 773 |
| *Toxicodendron vernix* |  | S2 | S2 | S3 | S2 | S3 | S2 | S3 | 16 | 54,293 | 15 |
| **APIACEAE** |  |  |  |  |  |  |  |  |  |  |  |
| *Ammoselinum popei* |  | S1 |  |  | S2 | S2 | S2 | S2 | 14 | 1,510 | 15 |
| *Angelica atropurpurea* |  | S1 |  |  |  |  |  |  |  |  |  |
| *Angelica triquinata* | S4 | S3 | S3 | S3 | S3 | S3 | S3 | S3 | 28 | 20,663 | 24 |
| *Angelica venenosa* | S5 | S3 | S3 | S3 | S3 | S3 | S3 | S3 | 31 | 52,185 | 29 |
| *Chaerophyllum procumbens* |  | S4 | S2 | S3 | S2 | S3 | S2 | S3 | 17 | 72,678 | 17 |
| *Chaerophyllum tainturieri* | S5 | S5 | S3 | S3 | S3 | S3 | S3 | S3 | 68 | 113,268 | 66 |
| *Cicuta maculata* |  | S5 | S3 | S3 | S3 | S3 | S3 | S3 | 28 | 88,510 | 27 |
| *Cryptotaenia canadensis* | S5 | S5 | S3 | S4 | S3 | S4 | S3 | S4 | 111 | 118,342 | 108 |
| *Cynosciadium digitatum* |  | S1 | S1 | S1 | S1 | S1 | S1 | S1 | 1 | <1 | 1 |
| *Daucus pusillus* |  | S2 | S1 | S1 | S1 | S1 | S1 | S1 | 2 | 29 | 2 |
| *Erigenia bulbosa* | S1 | S5 | S3 | S3 | S3 | S3 | S3 | S3 | 74 | 91,210 | 62 |
| *Eryngium integrifolium* | S1 | S2 | S2 | S2 | S2 | S2 | S2 | S2 | 8 | 11,835 | 8 |
| *Eryngium prostratum* |  | S4 | S2 | S3 | S2 | S3 | S2 | S3 | 16 | 42,644 | 14 |
| *Eryngium yuccifolium* | S5 | S4 | S3 | S3 | S3 | S3 | S3 | S3 | 71 | 98,646 | 69 |
| *Heracleum maximum* | S1 | S2 |  |  | S2 | S3 | S3 | S3 | 8 | 45,165 | 8 |
| *Ligusticum canadense* | S4 | S4 | S3 | S3 | S3 | S3 | S3 | S3 | 32 | 82,820 | 29 |
| *Osmorhiza claytonii* | S5 | S4 | S3 | S3 | S3 | S3 | S3 | S3 | 32 | 49,955 | 26 |
| *Osmorhiza longistylis* | S5 | S5 | S3 | S3 | S3 | S3 | S3 | S3 | 67 | 116,268 | 65 |
| *Oxypolis rigidior* | S4 | S5 | S3 | S3 | S3 | S3 | S3 | S3 | 60 | 72,859 | 58 |
| *Perideridia americana* | S2 | S1 |  |  | S2 | S2 | S2 | S2 | 9 | 4,437 | 9 |
| *Polytaenia nuttallii* |  | S1 |  |  | S1 | S1 | S1 | S1 | 3 | 249 | 3 |
| *Ptilimnium capillaceum* | S5 | S4 | S2 | S2 | S2 | S2 | S2 | S2 | 6 | 9,336 | 6 |
| *Ptilimnium costatum* | S1 | S3 | S1 | S1 | S1 | S1 | S1 | S1 | 5 | 763 | 5 |
| *Ptilimnium nuttallii* |  | S3 | S1 | S1 | S1 | S1 | S1 | S1 | 1 | <1 | 1 |
| *Sanicula canadensis* | S5 | S5 | S3 | S3 | S3 | S3 | S3 | S3 | 73 | 94,548 | 69 |
| *Sanicula marilandica* |  | S3 | S1 | S1 | S1 | S1 | S1 | S2 | 3 | 4,223 | 3 |
| *Sanicula odorata* | S4 | S5 | S3 | S3 | S3 | S3 | S3 | S3 | 39 | 97,796 | 34 |
| *Sanicula smallii* | S5 | S5 | S3 | S3 | S3 | S3 | S3 | S3 | 27 | 55,741 | 25 |
| *Sanicula trifoliata* | S4 | S4 | S3 | S3 | S3 | S3 | S3 | S3 | 24 | 20,187 | 23 |
| *Sium suave* | S3 | S3 | S1 | S2 | S1 | S2 | S2 | S2 | 4 | 16,500 | 4 |
| *Spermolepis echinata* |  | S1 | S1 | S1 | S1 | S1 | S1 | S1 | 1 | <1 | 1 |
| *Spermolepis inermis* |  | S1 | S1 | S1 | S1 | S1 | S1 | S1 | 1 | <1 | 1 |
| *Taenidia integerrima* | S5 | S5 | S3 | S3 | S3 | S3 | S3 | S3 | 56 | 76,874 | 53 |
| *Thaspium barbinode* | S5 | S5 | S3 | S3 | S3 | S3 | S3 | S3 | 61 | 58,636 | 56 |
| *Thaspium chapmanii* |  | S4 | S1 | S2 | S1 | S2 | S1 | S2 | 4 | 9,695 | 4 |
| *Thaspium pinnatifidum* | S1 | S2 | S1 | S1 | S1 | S1 | S1 | S1 | 1 | <1 | 1 |
| *Thaspium trifoliatum* | S5 | S5 | S3 | S3 | S3 | S3 | S3 | S3 | 51 | 70,608 | 50 |
| *Trepocarpus aethusae* | S1 | S3 | S2 | S3 | S2 | S3 | S2 | S3 | 12 | 36,974 | 12 |
| *Zizia aptera* | S5 | S5 | S3 | S3 | S3 | S3 | S3 | S3 | 32 | 70,845 | 31 |
| *Zizia aurea* | S4 | S5 | S3 | S3 | S3 | S3 | S3 | S3 | 61 | 108,321 | 59 |
| *Zizia trifoliata* | S5 | S4 | S2 | S1 | S2 | S1 | S2 | S1 | 14 | 974 | 13 |
| **APOCYNACEAE** |  |  |  |  |  |  |  |  |  |  |  |
| *Amsonia tabernaemontana* | S5 | S5 | S3 | S4 | S3 | S4 | S3 | S4 | 94 | 111,152 | 88 |
| *Apocynum androsaemifolium* |  | S3 | S1 | S1 | S1 | S1 | S1 | S1 | 3 | 1,216 | 3 |
| *Apocynum cannabinum* | S5 | S5 | S4 | S4 | S4 | S4 | S4 | S4 | 154 | 124,606 | 150 |
| *Apocynum xfloribundum* |  | S1 | S1 | S1 | S1 | S1 | S1 | S1 | 1 | <1 | 1 |
| *Asclepias amplexicaulis* | S5 | S4 | S3 | S3 | S3 | S3 | S3 | S3 | 60 | 76,910 | 57 |
| *Asclepias exaltata* |  | S4 | S3 | S3 | S3 | S3 | S3 | S3 | 50 | 30,777 | 50 |
| *Asclepias hirtella* | S2 | S3 | S2 | S2 | S2 | S2 | S2 | S2 | 15 | 7,376 | 15 |
| *Asclepias incarnata* | S5 | S5 | S3 | S4 | S3 | S4 | S3 | S4 | 117 | 120,209 | 113 |
| *Asclepias perennis* |  | S4 | S2 | S3 | S3 | S3 | S2 | S3 | 19 | 22,729 | 20 |
| *Asclepias purpurascens* |  | S3 | S2 | S3 | S2 | S3 | S2 | S3 | 14 | 39,307 | 14 |
| *Asclepias quadrifolia* | S4 | S5 | S3 | S4 | S3 | S4 | S3 | S4 | 116 | 73,216 | 111 |
| *Asclepias syriaca* | S5 | S5 | S4 | S5 | S4 | S5 | S4 | S5 | 381 | 125,645 | 348 |
| *Asclepias tuberosa* | S5 | S5 | S4 | S5 | S4 | S5 | S4 | S5 | 517 | 129,336 | 485 |
| *Asclepias variegata* | S5 | S5 | S4 | S4 | S4 | S4 | S4 | S4 | 191 | 110,926 | 183 |
| *Asclepias verticillata* | S4 | S5 | S3 | S3 | S3 | S3 | S3 | S3 | 68 | 53,643 | 66 |
| *Asclepias viridiflora* | S4 | S4 | S3 | S3 | S3 | S3 | S3 | S3 | 51 | 77,689 | 48 |
| *Asclepias viridis* |  | S2 | S3 | S3 | S3 | S3 | S3 | S3 | 38 | 19,124 | 38 |
| *Cynanchum laeve* |  | S5 | S3 | S4 | S3 | S4 | S3 | S4 | 115 | 109,403 | 109 |
| *Gonolobus suberosus* | S5 | S5 | S3 | S4 | S3 | S4 | S3 | S4 | 108 | 105,537 | 100 |
| *Matelea carolinensis* | S4 | S4 | S3 | S3 | S3 | S3 | S3 | S3 | 51 | 43,415 | 40 |
| *Matelea obliqua* |  | S4 | S2 | S3 | S2 | S3 | S2 | S3 | 17 | 33,816 | 15 |
| *Thyrsanthella difformis* |  | S3 | S2 | S3 | S2 | S3 | S2 | S3 | 10 | 32,592 | 10 |
| **AQUIFOLIACEAE** |  |  |  |  |  |  |  |  |  |  |  |
| *Ilex ambigua* | S4 | S4 | S2 | S2 | S2 | S2 | S2 | S3 | 11 | 17,923 | 11 |
| *Ilex collina* | S1 | S1 |  |  |  |  |  |  |  |  |  |
| *Ilex decidua* | S5 | S4 | S3 | S3 | S3 | S3 | S3 | S3 | 22 | 81,677 | 22 |
| *Ilex longipes* | S3 | S2 | S2 | S2 | S2 | S2 | S2 | S2 | 8 | 1,865 | 8 |
| *Ilex montana* | S5 | S4 | S3 | S3 | S3 | S3 | S3 | S3 | 35 | 45,136 | 35 |
| *Ilex opaca* | S5 | S5 | S4 | S5 | S4 | S5 | S4 | S5 | 408 | 130,248 | 348 |
| *Ilex verticillata* | S5 | S5 | S3 | S3 | S3 | S3 | S3 | S3 | 32 | 81,374 | 32 |
| **ARALIACEAE** |  |  |  |  |  |  |  |  |  |  |  |
| *Aralia nudicaulis* | S2 | S3 | S2 | S2 | S2 | S1 | S2 | S2 | 12 | 1,486 | 11 |
| *Aralia racemosa* | S4 | S4 | S3 | S3 | S3 | S3 | S3 | S3 | 42 | 72,080 | 40 |
| *Aralia spinosa* | S5 | S5 | S4 | S4 | S4 | S4 | S4 | S4 | 175 | 119,271 | 166 |
| *Hydrocotyle americana* | S1 | S1 |  |  | S1 | S2 | S2 | S2 | 4 | 9,004 | 4 |
| *Hydrocotyle ranunculoides* | S3 | S3 | S3 | S3 | S3 | S3 | S3 | S3 | 25 | 114,477 | 23 |
| *Hydrocotyle umbellata* | S5 | S1 | S1 | S2 | S1 | S1 | S1 | S2 | 4 | 5,614 | 4 |
| *Hydrocotyle verticillata* |  | S3 | S1 | S2 | S1 | S2 | S1 | S2 | 4 | 14,907 | 4 |
| *Panax quinquefolius* | S3 | S5 |  |  | S4 | S5 | S4 | S4 | 357 | 107,636 | 360 |
| *Panax trifoliatus* | S3 | S3 | S3 | S4 | S3 | S4 | S3 | S4 | 91 | 23,645 | 82 |
| **ASTERACEAE** |  |  |  |  |  |  |  |  |  |  |  |
| *Achillea millefolium* | S5 | S5 |  |  | S4 | S4 | S4 | S4 | 170 | 99,463 | 169 |
| *Acmella repens* |  | S2 | S2 | S2 | S2 | S2 | S2 | S2 | 11 | 3,581 | 10 |
| *Ageratina altissima* | S5 | S5 | S4 | S4 | S4 | S4 | S4 | S4 | 249 | 120,792 | 227 |
| *Ageratina aromatica* | S5 | S5 | S2 | S3 | S2 | S3 | S2 | S3 | 17 | 56,555 | 15 |
| *Ageratina luciae-brauniae* |  | S1 | S2 | S1 | S2 | S1 | S2 | S1 | 8 | 136 | 8 |
| *Ageratina roanensis* | S3 | S1 | S2 | S2 | S2 | S2 | S2 | S3 | 9 | 19,284 | 8 |
| *Ambrosia artemisiifolia* | S5 | S5 | S4 | S5 | S4 | S5 | S4 | S5 | 389 | 123,193 | 368 |
| *Ambrosia bidentata* |  | S4 | S2 | S3 | S2 | S3 | S2 | S3 | 9 | 52,893 | 9 |
| *Ambrosia trifida* | S5 | S5 | S4 | S5 | S4 | S5 | S4 | S5 | 318 | 127,093 | 306 |
| *Amphiachyris dracunculoides* |  | S3 | S2 | S2 | S2 | S2 | S2 | S2 | 15 | 4,285 | 14 |
| *Antennaria howellii* |  | S1 | S1 | S1 | S1 | S1 | S1 | S1 | 2 | 32 | 2 |
| *Antennaria neglecta* |  | S2 |  |  |  |  |  |  |  |  |  |
| *Antennaria parlinii* | S4 | S3 | S1 | S2 | S1 | S2 | S1 | S2 | 3 | 6,435 | 3 |
| *Antennaria plantaginifolia* | S5 | S5 | S3 | S3 | S3 | S3 | S3 | S3 | 68 | 87,272 | 68 |
| *Antennaria solitaria* | S4 | S5 | S3 | S3 | S3 | S3 | S3 | S3 | 71 | 89,314 | 63 |
| *Arnoglossum atriplicifolium* | S5 | S4 | S3 | S3 | S3 | S3 | S3 | S3 | 67 | 67,256 | 66 |
| *Arnoglossum plantagineum* |  | S1 | S2 | S2 | S2 | S2 | S2 | S2 | 13 | 3,976 | 13 |
| *Arnoglossum reniforme* |  | S3 | S3 | S3 | S3 | S3 | S3 | S3 | 29 | 88,037 | 29 |
| *Astranthium integrifolium* |  | S4 | S2 | S2 | S2 | S2 | S2 | S2 | 17 | 10,708 | 15 |
| *Bidens aristosa* |  | S5 | S3 | S3 | S3 | S3 | S3 | S3 | 49 | 99,716 | 47 |
| *Bidens bipinnata* | S5 | S5 | S3 | S4 | S3 | S4 | S3 | S4 | 118 | 109,861 | 112 |
| *Bidens cernua* |  | S4 | S2 | S3 | S2 | S3 | S2 | S3 | 10 | 55,570 | 10 |
| *Bidens connata* |  | S3 | S2 | S2 | S2 | S2 | S2 | S2 | 9 | 7,717 | 9 |
| *Bidens discoidea* | S4 | S4 | S2 | S2 | S1 | S2 | S2 | S2 | 5 | 30,964 | 5 |
| *Bidens frondosa* | S5 | S5 | S3 | S3 | S3 | S3 | S3 | S3 | 45 | 96,475 | 45 |
| *Bidens laevis* | S5 | S2 | S1 | S1 | S1 | S1 | S1 | S1 | 2 | 103 | 2 |
| *Bidens polylepis* |  | S5 | S3 | S3 | S3 | S3 | S3 | S3 | 27 | 78,582 | 25 |
| *Bidens tripartita* | S5 | S4 | S1 | S2 | S1 | S2 | S1 | S2 | 4 | 7,710 | 4 |
| *Bidens vulgata* | S5 | S3 | S1 | S1 | S1 | S1 | S1 | S1 | 2 | 47 | 2 |
| *Boltonia asteroides* | S3 | S3 | S2 | S3 | S2 | S3 | S2 | S3 | 7 | 46,842 | 7 |
| *Boltonia diffusa* |  | S3 | S1 | S2 | S1 | S2 | S1 | S2 | 5 | 7,720 | 5 |
| *Bradburia pilosa* |  | S1 |  |  |  |  |  |  |  |  |  |
| *Brickellia eupatorioides* | S5 | S4 | S3 | S3 | S3 | S3 | S3 | S3 | 30 | 69,379 | 29 |
| *Chrysogonum repens* |  | S1 |  |  |  |  |  |  |  |  |  |
| *Chrysopsis mariana* | S5 | S5 | S3 | S3 | S3 | S3 | S3 | S3 | 63 | 83,904 | 63 |
| *Cirsium altissimum* | S5 | S4 | S2 | S3 | S2 | S3 | S2 | S3 | 19 | 33,892 | 18 |
| *Cirsium carolinianum* |  | S4 | S2 | S2 | S2 | S2 | S2 | S2 | 8 | 8,246 | 8 |
| *Cirsium discolor* | S3 | S5 | S3 | S4 | S3 | S4 | S3 | S4 | 116 | 119,146 | 109 |
| *Cirsium horridulum* | S5 | S3 | S3 | S3 | S3 | S3 | S3 | S3 | 25 | 29,803 | 24 |
| *Cirsium muticum* |  | S3 | S2 | S3 | S2 | S3 | S2 | S3 | 13 | 37,006 | 13 |
| *Conoclinium coelestinum* | S5 | S5 | S4 | S5 | S4 | S5 | S4 | S5 | 630 | 127,669 | 561 |
| *Coreopsis auriculata* |  | S4 | S2 | S3 | S2 | S3 | S2 | S3 | 11 | 31,990 | 11 |
| *Coreopsis delphiniifolia* | S1 | S1 | S1 | S1 | S1 | S1 | S1 | S1 | 4 | 20 | 3 |
| *Coreopsis grandiflora* |  | S2 | S1 | S1 | S1 | S1 | S1 | S1 | 2 | <1 | 2 |
| *Coreopsis lanceolata* | S5 | S5 | S3 | S3 | S3 | S3 | S3 | S3 | 30 | 69,763 | 28 |
| *Coreopsis latifolia* |  | S1 |  |  | S2 | S2 | S2 | S1 | 6 | 2,909 | 7 |
| *Coreopsis major* | S5 | S5 | S4 | S4 | S4 | S4 | S4 | S4 | 150 | 98,044 | 141 |
| *Coreopsis pubescens* |  | S4 | S2 | S3 | S2 | S3 | S2 | S3 | 11 | 30,551 | 11 |
| *Coreopsis tinctoria* |  | S5 | S3 | S4 | S3 | S4 | S3 | S4 | 101 | 112,214 | 96 |
| *Coreopsis tripteris* | S5 | S5 | S3 | S3 | S3 | S3 | S3 | S3 | 63 | 73,439 | 59 |
| *Doellingeria infirma* | S5 | S4 | S2 | S3 | S2 | S3 | S2 | S3 | 8 | 36,673 | 8 |
| *Doellingeria umbellata* |  | S4 | S3 | S3 | S3 | S3 | S3 | S3 | 35 | 42,040 | 34 |
| *Echinacea pallida* |  | S1 | S1 | S1 | S1 | S1 | S1 | S1 | 1 | <1 | 1 |
| *Echinacea purpurea* | S3 | S4 | S3 | S3 | S3 | S3 | S3 | S3 | 77 | 107,704 | 76 |
| *Echinacea simulata* |  | S1 | S1 | S1 | S1 | S1 | S1 | S1 | 5 | 29 | 3 |
| *Echinacea tennesseensis* |  | S1 | S3 | S3 | S3 | S3 | S3 | S3 | 74 | 12,724 | 64 |
| *Eclipta prostrata* | S5 | S4 | S3 | S3 | S3 | S3 | S3 | S3 | 49 | 113,878 | 49 |
| *Elephantopus carolinianus* | S5 | S5 | S3 | S4 | S3 | S4 | S3 | S4 | 115 | 109,875 | 106 |
| *Elephantopus tomentosus* | S5 | S5 | S3 | S3 | S3 | S3 | S3 | S3 | 63 | 89,139 | 61 |
| *Erechtites hieraciifolius* | S5 | S5 | S4 | S4 | S4 | S4 | S4 | S4 | 141 | 112,071 | 135 |
| *Erigeron allisonii* | S1 | S1 | S2 | S1 | S2 | S1 | S2 | S1 | 10 | 680 | 9 |
| *Erigeron annuus* | S5 | S5 | S3 | S3 | S3 | S3 | S3 | S3 | 63 | 106,476 | 52 |
| *Erigeron canadensis* | S5 | S4 | S3 | S3 | S3 | S3 | S3 | S3 | 72 | 99,439 | 66 |
| *Erigeron divaricatus* |  | S3 | S1 | S1 | S1 | S1 | S1 | S1 | 1 | <1 | 1 |
| *Erigeron philadelphicus* |  | S5 | S4 | S4 | S4 | S4 | S4 | S4 | 238 | 126,776 | 221 |
| *Erigeron pulchellus* | S5 | S5 | S3 | S3 | S3 | S3 | S3 | S3 | 80 | 86,691 | 76 |
| *Erigeron strigosus* | S5 | S5 | S3 | S4 | S3 | S4 | S3 | S4 | 104 | 103,241 | 103 |
| *Eupatorium × pinnatifidum* |  | S1 | S1 | S1 | S1 | S1 | S1 | S1 | 1 | <1 | 1 |
| *Eupatorium album* | S5 | S5 | S3 | S3 | S3 | S3 | S3 | S3 | 29 | 64,695 | 28 |
| *Eupatorium altissimum* |  | S4 | S3 | S3 | S3 | S3 | S3 | S3 | 32 | 73,024 | 29 |
| *Eupatorium capillifolium* | S5 | S4 | S4 | S4 | S4 | S4 | S4 | S4 | 167 | 103,570 | 160 |
| *Eupatorium cordigerum* |  | S1 | S1 | S1 | S1 | S1 | S1 | S1 | 1 | <1 | 1 |
| *Eupatorium godfreyanum* |  | S1 | S1 | S1 | S1 | S1 | S1 | S1 | 2 | 16 | 2 |
| *Eupatorium hyssopifolium* | S5 | S5 | S3 | S3 | S3 | S3 | S3 | S3 | 28 | 81,503 | 27 |
| *Eupatorium leucolepis* |  | S1 | S1 | S1 | S1 | S1 | S1 | S1 | 2 | <1 | 2 |
| *Eupatorium perfoliatum* | S5 | S5 | S3 | S4 | S3 | S4 | S3 | S4 | 118 | 112,749 | 112 |
| *Eupatorium pilosum* | S5 | S4 | S3 | S3 | S3 | S3 | S3 | S3 | 32 | 23,653 | 31 |
| *Eupatorium pubescens* | S5 | S3 | S2 | S2 | S2 | S2 | S2 | S2 | 4 | 26,945 | 4 |
| *Eupatorium rotundifolium* | S5 | S5 | S3 | S4 | S3 | S4 | S3 | S3 | 82 | 84,230 | 82 |
| *Eupatorium scabridum* |  | S1 | S1 | S2 | S1 | S2 | S1 | S2 | 3 | 10,654 | 3 |
| *Eupatorium semiserratum* | S4 | S3 | S2 | S2 | S2 | S2 | S2 | S2 | 6 | 3,807 | 6 |
| *Eupatorium serotinum* | S5 | S5 | S4 | S4 | S4 | S4 | S4 | S4 | 294 | 124,093 | 283 |
| *Eupatorium sessilifolium* |  | S5 | S3 | S3 | S3 | S3 | S3 | S3 | 39 | 67,600 | 37 |
| *Eupatorium torreyanum* | S5 | S3 | S3 | S3 | S3 | S3 | S3 | S3 | 79 | 72,203 | 73 |
| *Eupatorium vaseyi* | S3 | S1 | S1 | S1 | S1 | S1 | S1 | S1 | 1 | <1 | 1 |
| *Eurybia chlorolepis* |  | S3 | S2 | S2 | S2 | S2 | S2 | S2 | 6 | 2,865 | 6 |
| *Eurybia divaricata* | S5 | S5 | S3 | S3 | S3 | S3 | S3 | S3 | 78 | 46,066 | 79 |
| *Eurybia hemispherica* |  | S5 | S3 | S3 | S3 | S3 | S3 | S3 | 61 | 59,964 | 56 |
| *Eurybia macrophylla* | S5 | S3 | S2 | S2 | S2 | S2 | S2 | S2 | 10 | 15,149 | 10 |
| *Eurybia saxicastellii* |  | S1 | S1 | S1 | S1 | S1 | S1 | S1 | 2 | 3 | 2 |
| *Eurybia schreberi* |  | S1 |  |  | S2 | S2 | S3 | S2 | 13 | 9,688 | 13 |
| *Eurybia surculosa* |  | S4 | S2 | S3 | S2 | S3 | S2 | S3 | 17 | 30,068 | 17 |
| *Euthamia gymnospermoides* |  | S1 | S1 | S1 | S1 | S1 | S1 | S1 | 1 | <1 | 1 |
| *Euthamia lanceolata* |  | S1 | S1 | S1 | S1 | S1 | S1 | S1 | 1 | <1 | 1 |
| *Euthamia leptocephala* |  | S2 | S1 | S1 | S1 | S1 | S1 | S1 | 2 | 11 | 2 |
| *Eutrochium fistulosum* | S5 | S4 | S3 | S4 | S3 | S4 | S3 | S4 | 98 | 87,025 | 95 |
| *Eutrochium maculatum* | S2 | S1 | S1 | S1 | S1 | S1 | S1 | S1 | 2 | 42 | 2 |
| *Eutrochium purpureum* | S5 | S4 | S3 | S3 | S3 | S3 | S3 | S3 | 46 | 92,370 | 45 |
| *Eutrochium steelei* | S3 | S2 | S2 | S2 | S2 | S2 | S2 | S2 | 9 | 5,192 | 9 |
| *Fleischmannia incarnata* | S2 | S5 | S3 | S3 | S3 | S3 | S3 | S3 | 45 | 103,939 | 43 |
| *Gamochaeta argyrinea* |  | S4 | S2 | S3 | S2 | S3 | S2 | S3 | 8 | 30,660 | 8 |
| *Gamochaeta pensylvanica* |  | S1 |  |  | S1 | S1 | S1 | S1 | 2 | <1 | 2 |
| *Gamochaeta purpurea* |  | S5 | S2 | S3 | S2 | S3 | S2 | S3 | 19 | 78,391 | 17 |
| *Grindelia lanceolata* |  | S3 |  |  | S3 | S3 | S3 | S3 | 30 | 9,536 | 29 |
| *Hasteola suaveolens* | S2 | S3 | S2 | S2 | S2 | S2 | S2 | S2 | 12 | 6,145 | 12 |
| *Helenium amarum* | S5 | S5 |  |  | S4 | S4 | S4 | S4 | 141 | 79,178 | 148 |
| *Helenium autumnale* | S5 | S5 | S3 | S4 | S3 | S4 | S3 | S4 | 106 | 107,501 | 99 |
| *Helenium brevifolium* | S1 | S1 |  |  | S1 | S1 | S1 | S1 | 4 | 126 | 4 |
| *Helenium flexuosum* | S5 | S5 | S3 | S4 | S3 | S4 | S3 | S4 | 111 | 108,232 | 107 |
| *Helianthus angustifolius* | S4 | S5 | S3 | S3 | S3 | S3 | S3 | S3 | 66 | 102,139 | 63 |
| *Helianthus atrorubens* |  | S5 | S3 | S3 | S3 | S3 | S3 | S3 | 37 | 63,119 | 38 |
| *Helianthus decapetalus* |  | S4 | S2 | S3 | S3 | S3 | S2 | S3 | 22 | 63,689 | 20 |
| *Helianthus divaricatus* | S5 | S5 | S3 | S3 | S3 | S3 | S3 | S3 | 35 | 69,662 | 33 |
| *Helianthus eggertii* |  | S3 |  |  | S3 | S4 | S3 | S4 | 34 | 30,462 | 34 |
| *Helianthus giganteus* | S5 | S3 | S2 | S2 | S2 | S2 | S2 | S2 | 6 | 4,995 | 6 |
| *Helianthus glaucophyllus* |  | S1 |  |  | S2 | S2 | S2 | S2 | 20 | 1,059 | 18 |
| *Helianthus grosseserratus* |  | S3 | S1 | S1 | S1 | S1 | S1 | S1 | 3 | <1 | 2 |
| *Helianthus hirsutus* | S3 | S5 | S3 | S3 | S3 | S3 | S3 | S3 | 66 | 92,363 | 63 |
| *Helianthus microcephalus* | S5 | S5 | S3 | S3 | S3 | S3 | S3 | S3 | 82 | 110,741 | 77 |
| *Helianthus mollis* |  | S4 | S3 | S3 | S3 | S3 | S3 | S3 | 31 | 55,908 | 30 |
| *Helianthus occidentalis* | S1 | S3 | S2 | S3 | S2 | S3 | S2 | S3 | 20 | 49,917 | 17 |
| *Helianthus petiolaris* |  | S1 |  |  |  |  |  |  |  |  |  |
| *Helianthus silphioides* |  | S3 | S2 | S3 | S2 | S3 | S2 | S3 | 7 | 42,825 | 6 |
| *Helianthus smithii* |  | S2 |  |  |  |  |  |  |  |  |  |
| *Helianthus strumosus* | S4 | S5 | S2 | S3 | S2 | S3 | S2 | S3 | 13 | 52,190 | 13 |
| *Helianthus tuberosus* | S5 | S5 | S3 | S3 | S3 | S3 | S3 | S3 | 41 | 97,957 | 41 |
| *Heliopsis helianthoides* |  | S4 | S3 | S3 | S3 | S3 | S3 | S3 | 28 | 66,222 | 26 |
| *Heterotheca camporum* |  | S5 |  |  | S3 | S3 | S3 | S3 | 60 | 67,414 | 59 |
| *Heterotheca subaxillaris* |  | S3 | S2 | S3 | S2 | S3 | S2 | S3 | 14 | 95,622 | 14 |
| *Hieracium gronovii* | S5 | S5 | S3 | S3 | S3 | S3 | S3 | S3 | 35 | 93,532 | 35 |
| *Hieracium longipilum* |  | S2 | S1 | S1 | S1 | S1 | S1 | S1 | 2 | 96 | 2 |
| *Hieracium paniculatum* | S5 | S4 | S2 | S3 | S2 | S3 | S2 | S3 | 18 | 31,273 | 16 |
| *Hieracium scabrum* | S2 | S3 |  |  |  |  |  |  |  |  |  |
| *Hieracium venosum* | S5 | S5 | S3 | S3 | S3 | S4 | S3 | S3 | 85 | 58,077 | 75 |
| *Ionactis linariifolia* | S5 | S4 | S3 | S3 | S3 | S3 | S3 | S3 | 29 | 56,720 | 28 |
| *Iva annua* |  | S5 | S3 | S3 | S3 | S3 | S3 | S3 | 28 | 95,814 | 28 |
| *Krigia biflora* |  | S5 | S4 | S4 | S4 | S4 | S4 | S4 | 159 | 73,613 | 151 |
| *Krigia cespitosa* | S3 | S5 | S3 | S3 | S3 | S3 | S3 | S3 | 35 | 85,302 | 33 |
| *Krigia dandelion* | S5 | S5 | S3 | S3 | S3 | S3 | S3 | S3 | 43 | 75,757 | 43 |
| *Krigia montana* | S3 | S1 | S1 | S1 | S1 | S1 | S1 | S1 | 4 | 1,246 | 4 |
| *Krigia virginica* | S5 | S5 | S2 | S3 | S2 | S3 | S2 | S3 | 16 | 51,730 | 15 |
| *Lactuca biennis* |  | S3 | S2 | S3 | S2 | S3 | S2 | S3 | 15 | 36,472 | 14 |
| *Lactuca canadensis* |  | S5 | S3 | S3 | S3 | S3 | S3 | S3 | 77 | 107,055 | 75 |
| *Lactuca floridana* |  | S5 | S4 | S4 | S4 | S4 | S4 | S4 | 144 | 121,562 | 135 |
| *Lactuca graminifolia* |  | S1 | S1 | S1 | S1 | S1 | S1 | S1 | 1 | <1 | 1 |
| *Lactuca hirsuta* | S3 | S2 | S2 | S2 | S2 | S2 | S2 | S2 | 9 | 5,405 | 8 |
| *Liatris aspera* | S3 | S4 | S2 | S3 | S3 | S3 | S2 | S3 | 24 | 70,202 | 20 |
| *Liatris cylindracea* | S2 | S2 | S2 | S2 | S2 | S2 | S2 | S2 | 9 | 11,897 | 8 |
| *Liatris hirsuta* |  | S1 | S1 | S1 | S1 | S1 | S1 | S1 | 1 | <1 | 1 |
| *Liatris microcephala* | S3 | S4 | S3 | S3 | S3 | S3 | S3 | S3 | 64 | 20,919 | 59 |
| *Liatris pilosa* |  | S1 |  |  | S1 | S1 | S1 | S1 | 1 | <1 | 1 |
| *Liatris scariosa* |  | S3 | S1 | S1 | S1 | S1 | S1 | S1 | 2 | 50 | 2 |
| *Liatris spicata* |  | S4 | S3 | S3 | S3 | S3 | S3 | S3 | 57 | 99,431 | 53 |
| *Liatris squarrosa* | S5 | S5 | S3 | S3 | S3 | S3 | S3 | S3 | 38 | 67,609 | 34 |
| *Liatris squarrulosa* |  | S5 | S3 | S3 | S3 | S3 | S3 | S3 | 54 | 94,118 | 52 |
| *Liatris virgata* |  | S1 |  |  | S1 | S1 | S1 | S1 | 1 | <1 | 1 |
| *Marshallia obovata* |  | S1 |  |  | S1 | S1 | S1 | S1 | 1 | <1 | 1 |
| *Marshallia pulchra* |  | S1 | S2 | S1 | S2 | S1 | S2 | S1 | 8 | 686 | 8 |
| *Marshallia trinervia* | S3 | S2 |  |  | S2 | S3 | S3 | S2 | 8 | 2,115 | 9 |
| *Melanthera nivea* | S3 | S3 | S2 | S2 | S2 | S2 | S2 | S3 | 9 | 18,735 | 9 |
| *Mikania scandens* |  | S4 | S3 | S3 | S3 | S3 | S3 | S3 | 28 | 61,477 | 27 |
| *Nabalus albus* | S2 | S1 | S1 | S2 | S1 | S2 | S1 | S2 | 4 | 7,572 | 4 |
| *Nabalus altissimus* | S5 | S4 | S3 | S3 | S3 | S3 | S3 | S3 | 18 | 32,050 | 16 |
| *Nabalus asper* | S2 | S1 | S1 | S2 | S1 | S2 | S1 | S2 | 5 | 12,592 | 5 |
| *Nabalus barbatus* | S1 | S3 | S3 | S3 | S3 | S3 | S3 | S3 | 27 | 25,150 | 25 |
| *Nabalus crepidineus* | S1 | S3 | S2 | S2 | S2 | S2 | S2 | S2 | 18 | 8,997 | 17 |
| *Nabalus roanensis* | S3 | S1 | S1 | S1 | S2 | S2 | S1 | S1 | 1 | <1 | 1 |
| *Nabalus serpentaria* | S5 | S3 | S3 | S3 | S3 | S3 | S3 | S3 | 21 | 25,854 | 21 |
| *Nabalus trifoliolatus* |  | S2 | S2 | S3 | S2 | S3 | S2 | S3 | 8 | 30,015 | 8 |
| *Oclemena acuminata* |  | S2 | S2 | S2 | S2 | S2 | S2 | S2 | 17 | 2,432 | 16 |
| *Packera anonyma* | S5 | S5 | S4 | S4 | S4 | S4 | S4 | S4 | 151 | 102,538 | 140 |
| *Packera aurea* | S5 | S4 | S3 | S4 | S3 | S4 | S3 | S4 | 94 | 68,133 | 92 |
| *Packera crawfordii* | S1 | S1 | S2 | S2 | S2 | S2 | S2 | S2 | 7 | 10,772 | 7 |
| *Packera glabella* | S5 | S5 | S4 | S5 | S4 | S5 | S4 | S5 | 401 | 105,580 | 367 |
| *Packera obovata* | S5 | S5 | S3 | S3 | S3 | S3 | S3 | S3 | 60 | 87,030 | 57 |
| *Packera paupercula* | S1 | S3 | S2 | S3 | S2 | S3 | S2 | S3 | 18 | 52,036 | 17 |
| *Packera schweinitziana* |  | S1 | S1 | S1 | S1 | S1 | S1 | S1 | 3 | 131 | 3 |
| *Parthenium integrifolium* |  | S5 | S3 | S4 | S3 | S4 | S3 | S4 | 97 | 69,352 | 92 |
| *Pityopsis graminifolia* |  | S4 | S3 | S3 | S3 | S3 | S3 | S3 | 40 | 92,996 | 40 |
| *Pluchea camphorata* | S5 | S5 | S3 | S3 | S3 | S3 | S3 | S3 | 71 | 103,128 | 66 |
| *Polymnia canadensis* | S5 | S5 | S4 | S4 | S4 | S4 | S4 | S4 | 152 | 73,516 | 141 |
| *Polymnia laevigata* | S1 | S3 | S2 | S2 | S2 | S2 | S2 | S2 | 17 | 1,467 | 13 |
| *Pseudognaphalium helleri* |  | S1 |  |  | S2 | S3 | S2 | S3 | 7 | 31,002 | 7 |
| *Pseudognaphalium micradenium* | S1 | S1 |  |  |  |  |  |  |  |  |  |
| *Pseudognaphalium obtusifolium* | S5 | S4 | S3 | S3 | S3 | S3 | S3 | S3 | 74 | 105,614 | 71 |
| *Pyrrhopappus carolinianus* | S5 | S5 | S3 | S4 | S3 | S4 | S3 | S4 | 85 | 99,436 | 81 |
| *Ratibida pinnata* |  | S4 | S3 | S3 | S3 | S3 | S3 | S3 | 62 | 79,748 | 57 |
| *Rudbeckia acuminata* |  | S1 |  |  |  |  |  |  |  |  |  |
| *Rudbeckia fulgida* | S4 | S5 | S3 | S3 | S3 | S3 | S3 | S3 | 32 | 65,428 | 32 |
| *Rudbeckia hirta* | S5 | S5 | S4 | S4 | S4 | S4 | S4 | S4 | 236 | 131,733 | 227 |
| *Rudbeckia laciniata* | S5 | S5 | S4 | S4 | S4 | S4 | S4 | S4 | 180 | 113,285 | 163 |
| *Rudbeckia palustris* |  | S3 | S2 | S2 | S2 | S2 | S2 | S2 | 12 | 15,306 | 12 |
| *Rudbeckia spathulata* |  | S1 |  |  | S1 | S1 | S1 | S1 | 2 | <1 | 2 |
| *Rudbeckia speciosa* |  | S1 |  |  |  |  |  |  |  |  |  |
| *Rudbeckia subtomentosa* | S1 | S1 | S2 | S2 | S1 | S2 | S2 | S2 | 4 | 23,324 | 4 |
| *Rudbeckia tenax* | S4 | S3 | S1 | S1 | S1 | S1 | S1 | S1 | 2 | 1 | 2 |
| *Rudbeckia terranigrae* |  | S1 | S1 | S1 | S1 | S1 | S1 | S1 | 1 | <1 | 1 |
| *Rudbeckia triloba* |  | S5 | S3 | S4 | S3 | S4 | S3 | S4 | 115 | 113,376 | 112 |
| *Rudbeckia truncata* |  | S1 |  |  |  |  |  |  |  |  |  |
| *Rudbeckia umbrosa* |  | S3 | S2 | S2 | S2 | S2 | S2 | S2 | 8 | 14,750 | 8 |
| *Rugelia nudicaulis* |  | S1 | S2 | S1 | S2 | S2 | S2 | S2 | 24 | 433 | 18 |
| *Sericocarpus caespitosus* |  | S2 | S1 | S1 | S1 | S1 | S1 | S1 | 1 | <1 | 1 |
| *Sericocarpus linifolius* | S5 | S5 | S3 | S3 | S3 | S3 | S3 | S3 | 38 | 75,338 | 37 |
| *Silphium asperrimum* |  | S2 | S2 | S2 | S2 | S2 | S2 | S3 | 16 | 18,550 | 16 |
| *Silphium asteriscus* |  | S4 | S3 | S3 | S3 | S3 | S3 | S3 | 63 | 72,507 | 59 |
| *Silphium brachiatum* |  | S1 |  |  | S2 | S2 | S2 | S1 | 10 | 331 | 9 |
| *Silphium compositum* | S5 | S3 | S2 | S2 | S2 | S2 | S2 | S2 | 8 | 6,441 | 8 |
| *Silphium glabrum* |  | S4 | S2 | S3 | S2 | S3 | S2 | S3 | 18 | 38,216 | 17 |
| *Silphium integrifolium* |  | S3 | S2 | S3 | S2 | S3 | S2 | S3 | 8 | 41,703 | 8 |
| *Silphium laciniatum* |  | S2 |  |  | S2 | S3 | S2 | S3 | 11 | 20,415 | 12 |
| *Silphium mohrii* | S1 | S3 | S2 | S2 | S2 | S2 | S2 | S3 | 14 | 18,655 | 13 |
| *Silphium perfoliatum* | S3 | S4 | S3 | S3 | S3 | S3 | S3 | S3 | 40 | 114,569 | 34 |
| *Silphium pinnatifidum* | S3 | S2 | S3 | S3 | S3 | S3 | S3 | S3 | 26 | 20,242 | 25 |
| *Silphium reniforme* |  | S1 |  |  |  |  |  |  |  |  |  |
| *Silphium terebinthinaceum* | S3 | S3 | S3 | S3 | S3 | S3 | S3 | S3 | 27 | 54,533 | 26 |
| *Silphium trifoliatum* | S4 | S4 | S3 | S3 | S3 | S3 | S3 | S3 | 22 | 32,543 | 21 |
| *Silphium wasiotense* |  | S1 | S1 | S1 | S1 | S1 | S1 | S1 | 5 | 490 | 4 |
| *Smallanthus uvedalia* | S4 | S4 | S4 | S4 | S4 | S4 | S4 | S4 | 265 | 123,161 | 230 |
| *Solidago altissima* | S5 | S4 | S3 | S4 | S3 | S4 | S3 | S4 | 87 | 124,777 | 82 |
| *Solidago arenicola* | S1 | S1 | S2 | S1 | S2 | S1 | S2 | S1 | 7 | 597 | 7 |
| *Solidago arguta* | S5 | S4 | S3 | S3 | S3 | S3 | S3 | S3 | 27 | 50,796 | 23 |
| *Solidago auriculata* |  | S1 | S1 | S1 | S1 | S1 | S1 | S1 | 5 | 207 | 4 |
| *Solidago austrina* |  | S1 | S1 | S1 | S1 | S1 | S1 | S1 | 3 | 31 | 3 |
| *Solidago bicolor* | S5 | S4 | S2 | S2 | S2 | S2 | S2 | S3 | 15 | 19,360 | 16 |
| *Solidago boottii* |  | S2 |  |  |  |  |  |  |  |  |  |
| *Solidago caesia* | S5 | S5 | S3 | S4 | S3 | S4 | S3 | S4 | 98 | 108,581 | 94 |
| *Solidago canadensis* |  | S5 | S2 | S3 | S2 | S3 | S2 | S3 | 9 | 62,283 | 8 |
| *Solidago curtisii* | S4 | S4 | S3 | S3 | S3 | S3 | S3 | S3 | 51 | 37,906 | 48 |
| *Solidago erecta* | S5 | S5 | S3 | S3 | S3 | S3 | S3 | S3 | 37 | 76,288 | 36 |
| *Solidago faucibus* | S3 | S1 |  |  | S1 | S1 | S1 | S1 | 1 | <1 | 1 |
| *Solidago flaccidifolia* | S4 | S4 | S2 | S2 | S2 | S2 | S2 | S2 | 9 | 10,036 | 9 |
| *Solidago flexicaulis* | S5 | S5 | S3 | S3 | S3 | S3 | S3 | S3 | 47 | 72,574 | 46 |
| *Solidago gattingeri* |  | S2 | S1 | S1 | S1 | S1 | S1 | S1 | 3 | 39 | 3 |
| *Solidago gigantea* | S5 | S5 | S3 | S3 | S3 | S3 | S3 | S3 | 29 | 93,086 | 29 |
| *Solidago glomerata* |  | S1 | S2 | S2 | S1 | S1 | S2 | S2 | 6 | 1,101 | 6 |
| *Solidago hispida* |  | S4 | S2 | S3 | S2 | S3 | S2 | S3 | 17 | 50,009 | 17 |
| *Solidago juncea* | S1 | S5 | S3 | S3 | S3 | S3 | S3 | S3 | 27 | 58,984 | 24 |
| *Solidago lancifolia* | S1 | S1 | S1 | S1 | S1 | S1 | S1 | S1 | 1 | <1 | 1 |
| *Solidago missouriensis* |  | S3 |  |  | S1 | S1 | S1 | S1 | 3 | 639 | 3 |
| *Solidago nemoralis* | S5 | S5 | S3 | S3 | S3 | S3 | S3 | S3 | 70 | 101,253 | 68 |
| *Solidago odora* | S5 | S5 | S3 | S3 | S3 | S3 | S3 | S3 | 54 | 87,706 | 54 |
| *Solidago patula* | S3 | S4 | S3 | S3 | S3 | S3 | S3 | S3 | 26 | 70,335 | 26 |
| *Solidago ptarmicoides* | S1 | S1 | S2 | S2 | S2 | S2 | S2 | S2 | 8 | 5,157 | 7 |
| *Solidago puberula* |  | S3 | S2 | S2 | S2 | S2 | S2 | S2 | 13 | 3,096 | 11 |
| *Solidago racemosa* |  | S1 | S1 | S1 | S1 | S1 | S1 | S1 | 4 | 18 | 5 |
| *Solidago radula* | S1 | S1 |  |  |  |  |  |  |  |  |  |
| *Solidago rigida* |  | S3 | S2 | S3 | S2 | S3 | S2 | S3 | 14 | 46,102 | 13 |
| *Solidago rigidiuscula* | S2 | S1 | S1 | S1 | S1 | S1 | S1 | S1 | 3 | 3,567 | 3 |
| *Solidago roanensis* | S4 | S4 | S2 | S3 | S2 | S3 | S2 | S3 | 12 | 34,716 | 11 |
| *Solidago rugosa* | S5 | S5 | S3 | S3 | S3 | S3 | S3 | S3 | 57 | 83,661 | 51 |
| *Solidago rupestris* |  | S2 | S1 | S1 | S1 | S1 | S1 | S1 | 2 | 31 | 2 |
| *Solidago simulans* |  | S1 |  |  |  |  |  |  |  |  |  |
| *Solidago speciosa* | S5 | S4 | S2 | S2 | S2 | S2 | S2 | S2 | 13 | 15,864 | 13 |
| *Solidago sphacelata* |  | S5 | S2 | S3 | S2 | S3 | S2 | S3 | 19 | 39,418 | 16 |
| *Solidago spithamaea* |  | S1 |  |  | S1 | S1 | S1 | S1 | 1 | <1 | 1 |
| *Solidago tarda* |  | S1 | S1 | S1 | S1 | S1 | S1 | S1 | 1 | <1 | 1 |
| *Solidago ulmifolia* |  | S5 | S3 | S3 | S3 | S3 | S3 | S3 | 31 | 93,486 | 30 |
| *Solidago vaseyi* | S4 | S1 | S2 | S2 | S2 | S2 | S2 | S3 | 11 | 16,021 | 10 |
| *Symphyotrichum concinnum* |  | S1 | S1 | S1 | S1 | S1 | S1 | S1 | 2 | <1 | 2 |
| *Symphyotrichum concolor* |  | S4 | S2 | S3 | S2 | S3 | S2 | S3 | 13 | 40,659 | 13 |
| *Symphyotrichum cordifolium* |  | S5 | S3 | S3 | S3 | S3 | S3 | S3 | 68 | 96,411 | 66 |
| *Symphyotrichum divaricatum* |  | S3 | S2 | S2 | S2 | S2 | S2 | S2 | 4 | 32,348 | 4 |
| *Symphyotrichum drummondii* |  | S1 | S1 | S1 | S1 | S1 | S1 | S1 | 4 | 2,465 | 4 |
| *Symphyotrichum dumosum* |  | S5 | S3 | S3 | S3 | S3 | S3 | S3 | 49 | 79,237 | 48 |
| *Symphyotrichum ericoides* |  | S1 | S1 | S1 | S1 | S1 | S1 | S1 | 3 | 706 | 3 |
| *Symphyotrichum georgianum* | S3 | S1 |  |  |  |  |  |  |  |  |  |
| *Symphyotrichum kentuckiense* |  | S2 | S2 | S2 | S2 | S2 | S2 | S3 | 14 | 17,785 | 11 |
| *Symphyotrichum laeve* |  | S3 | S3 | S3 | S3 | S3 | S3 | S3 | 25 | 49,400 | 23 |
| *Symphyotrichum lanceolatum* |  | S5 | S3 | S3 | S3 | S3 | S3 | S3 | 22 | 95,799 | 21 |
| *Symphyotrichum lateriflorum* | S5 | S5 | S3 | S3 | S3 | S3 | S3 | S3 | 72 | 105,179 | 70 |
| *Symphyotrichum lowrieanum* |  | S3 | S1 | S1 | S1 | S1 | S1 | S1 | 2 | 1 | 2 |
| *Symphyotrichum novae-angliae* | S5 | S4 | S3 | S3 | S3 | S3 | S3 | S3 | 46 | 111,271 | 43 |
| *Symphyotrichum oblongifolium* |  | S4 | S3 | S3 | S3 | S3 | S3 | S3 | 25 | 84,300 | 24 |
| *Symphyotrichum ontarionis* |  | S4 | S2 | S3 | S2 | S3 | S2 | S3 | 15 | 55,207 | 15 |
| *Symphyotrichum oolentangiense* | S1 | S1 |  |  |  |  |  |  |  |  |  |
| *Symphyotrichum patens* | S5 | S5 | S3 | S4 | S3 | S4 | S3 | S4 | 110 | 113,920 | 105 |
| *Symphyotrichum phlogifolium* |  | S4 | S2 | S3 | S2 | S2 | S2 | S3 | 6 | 23,174 | 6 |
| *Symphyotrichum pilosum* | S5 | S5 | S3 | S3 | S3 | S3 | S3 | S3 | 71 | 105,328 | 68 |
| *Symphyotrichum praealtum* | S1 | S1 |  |  | S2 | S2 | S2 | S2 | 4 | 29,015 | 5 |
| *Symphyotrichum pratense* | S1 | S2 |  |  | S2 | S2 | S2 | S2 | 8 | 185 | 8 |
| *Symphyotrichum puniceum* |  | S3 | S3 | S3 | S3 | S3 | S3 | S3 | 36 | 52,705 | 35 |
| *Symphyotrichum racemosum* |  | S4 | S1 | S1 | S1 | S1 | S1 | S1 | 2 | 93 | 2 |
| *Symphyotrichum retroflexum* |  | S3 | S3 | S3 | S3 | S3 | S3 | S3 | 27 | 5,022 | 27 |
| *Symphyotrichum shortii* | S1 | S4 | S3 | S3 | S3 | S3 | S3 | S3 | 52 | 61,611 | 50 |
| *Symphyotrichum undulatum* |  | S5 | S3 | S3 | S3 | S3 | S3 | S3 | 42 | 83,472 | 39 |
| *Symphyotrichum urophyllum* |  | S4 | S2 | S3 | S2 | S3 | S2 | S3 | 7 | 38,463 | 7 |
| *Verbesina alternifolia* | S5 | S5 | S4 | S5 | S4 | S5 | S4 | S5 | 390 | 121,090 | 341 |
| *Verbesina helianthoides* |  | S4 | S3 | S3 | S3 | S3 | S3 | S3 | 40 | 32,351 | 37 |
| *Verbesina occidentalis* | S5 | S5 | S4 | S4 | S4 | S4 | S4 | S4 | 229 | 70,864 | 220 |
| *Verbesina virginica* |  | S5 | S4 | S5 | S4 | S5 | S4 | S5 | 468 | 110,781 | 418 |
| *Vernonia flaccidifolia* |  | S2 | S2 | S1 | S2 | S1 | S2 | S1 | 6 | 665 | 6 |
| *Vernonia gigantea* |  | S5 | S4 | S4 | S4 | S4 | S4 | S4 | 191 | 124,563 | 180 |
| *Vernonia missurica* |  | S4 | S2 | S3 | S2 | S3 | S2 | S3 | 11 | 43,206 | 11 |
| *Vernonia noveboracensis* | S5 | S4 | S2 | S3 | S2 | S3 | S2 | S3 | 14 | 41,238 | 14 |
| **BALSAMINACEAE** |  |  |  |  |  |  |  |  |  |  |  |
| *Impatiens capensis* | S5 | S5 | S4 | S5 | S4 | S5 | S4 | S5 | 591 | 133,627 | 529 |
| *Impatiens pallida* | S5 | S5 | S4 | S4 | S4 | S4 | S4 | S4 | 207 | 89,497 | 184 |
| **BERBERIDACEAE** |  |  |  |  |  |  |  |  |  |  |  |
| *Berberis canadensis* | S1 | S4 |  |  | S3 | S3 | S3 | S3 | 27 | 19,235 | 26 |
| *Caulophyllum giganteum* |  | S1 | S2 | S3 | S2 | S3 | S2 | S3 | 17 | 47,055 | 16 |
| *Caulophyllum thalictroides* | S4 | S5 | S3 | S4 | S3 | S4 | S3 | S4 | 106 | 71,786 | 93 |
| *Diphylleia cymosa* | S3 | S2 | S3 | S3 | S3 | S3 | S3 | S3 | 49 | 6,077 | 42 |
| *Jeffersonia diphylla* |  | S5 | S3 | S4 | S3 | S4 | S3 | S4 | 97 | 71,363 | 83 |
| *Podophyllum peltatum* | S5 | S5 | S4 | S5 | S4 | S5 | S4 | S5 | 753 | 130,411 | 654 |
| **BETULACEAE** |  |  |  |  |  |  |  |  |  |  |  |
| *Alnus alnobetula* |  | S1 | S2 | S2 | S2 | S2 | S2 | S2 | 8 | 6,616 | 6 |
| *Alnus serrulata* | S5 | S5 | S3 | S3 | S3 | S3 | S3 | S3 | 74 | 110,906 | 72 |
| *Betula alleghaniensis* | S5 | S3 | S3 | S3 | S3 | S3 | S3 | S3 | 46 | 51,742 | 44 |
| *Betula cordifolia* |  | S1 |  |  | S1 | S1 | S1 | S1 | 1 | <1 | 1 |
| *Betula lenta* |  | S4 | S3 | S3 | S3 | S3 | S3 | S3 | 28 | 47,873 | 28 |
| *Betula nigra* | S5 | S5 | S3 | S3 | S3 | S3 | S3 | S3 | 68 | 117,942 | 67 |
| *Carpinus caroliniana* | S5 | S5 | S4 | S4 | S4 | S4 | S4 | S4 | 138 | 129,413 | 130 |
| *Corylus americana* | S5 | S5 | S3 | S3 | S3 | S3 | S3 | S3 | 50 | 107,782 | 49 |
| *Corylus cornuta* | S4 | S3 | S1 | S2 | S1 | S1 | S1 | S2 | 4 | 5,584 | 4 |
| *Ostrya virginiana* | S5 | S5 | S3 | S4 | S3 | S4 | S3 | S4 | 124 | 118,972 | 117 |
| **BIGNONIACEAE** |  |  |  |  |  |  |  |  |  |  |  |
| *Bignonia capreolata* | S5 | S5 | S4 | S5 | S4 | S5 | S4 | S5 | 566 | 125,998 | 509 |
| *Campsis radicans* | S5 | S5 | S4 | S5 | S4 | S5 | S4 | S5 | 583 | 126,325 | 514 |
| *Catalpa bignonioides* |  | S4 | S2 | S3 | S2 | S3 | S2 | S3 | 17 | 86,432 | 17 |
| *Catalpa speciosa* |  | S5 | S3 | S3 | S3 | S3 | S3 | S3 | 25 | 104,174 | 25 |
| **BORAGINACEAE** |  |  |  |  |  |  |  |  |  |  |  |
| *Andersonglossum virginianum* |  | S3 | S4 | S5 | S4 | S5 | S4 | S5 | 378 | 118,317 | 331 |
| *Euploca procumbens* |  | S1 | S1 | S1 | S1 | S1 | S1 | S1 | 1 | <1 | 1 |
| *Euploca tenella* |  | S1 | S3 | S3 | S3 | S3 | S3 | S3 | 37 | 9,023 | 30 |
| *Hackelia virginiana* |  | S4 | S2 | S2 | S2 | S2 | S2 | S2 | 8 | 5,342 | 7 |
| *Hydrophyllum appendiculatum* |  | S4 | S3 | S3 | S3 | S3 | S3 | S3 | 35 | 66,356 | 29 |
| *Hydrophyllum canadense* | S5 | S5 | S3 | S3 | S3 | S3 | S3 | S3 | 55 | 66,161 | 49 |
| *Hydrophyllum macrophyllum* |  | S4 | S3 | S3 | S3 | S3 | S3 | S3 | 51 | 102,637 | 47 |
| *Hydrophyllum virginianum* |  | S3 |  |  | S3 | S3 | S3 | S3 | 27 | 3,675 | 26 |
| *Lithospermum bejariense* | S1 | S1 |  |  | S2 | S2 | S2 | S2 | 10 | 12,982 | 8 |
| *Lithospermum canescens* | S4 | S5 | S3 | S3 | S3 | S3 | S3 | S3 | 66 | 64,578 | 56 |
| *Lithospermum latifolium* | S1 | S3 | S1 | S2 | S1 | S1 | S1 | S2 | 5 | 5,462 | 5 |
| *Lithospermum molle* |  | S3 | S2 | S2 | S2 | S3 | S2 | S2 | 23 | 4,688 | 19 |
| *Lithospermum parviflorum* | S3 | S2 | S2 | S2 | S2 | S2 | S2 | S3 | 8 | 17,372 | 9 |
| *Lithospermum subsetosum* |  | S1 | S1 | S1 | S1 | S1 | S1 | S1 | 2 | 1 | 2 |
| *Lithospermum tuberosum* | S3 | S4 | S3 | S3 | S3 | S3 | S3 | S3 | 41 | 50,003 | 37 |
| *Mertensia virginica* |  | S5 | S4 | S4 | S4 | S4 | S4 | S4 | 256 | 115,190 | 237 |
| *Myosotis laxa* |  | S3 | S1 | S1 | S1 | S1 | S1 | S1 | 5 | 749 | 5 |
| *Myosotis macrosperma* | S4 | S5 | S3 | S3 | S3 | S3 | S3 | S3 | 56 | 96,341 | 53 |
| *Myosotis verna* |  | S5 | S2 | S3 | S2 | S3 | S2 | S3 | 8 | 29,413 | 8 |
| *Nemophila aphylla* | S4 | S4 | S3 | S3 | S3 | S3 | S3 | S3 | 67 | 96,924 | 61 |
| *Phacelia bipinnatifida* | S4 | S5 | S4 | S4 | S4 | S4 | S4 | S4 | 229 | 108,876 | 200 |
| *Phacelia dubia* | S3 | S4 | S3 | S3 | S3 | S3 | S3 | S3 | 41 | 77,145 | 39 |
| *Phacelia fimbriata* |  | S3 | S3 | S3 | S3 | S3 | S3 | S3 | 38 | 19,020 | 31 |
| *Phacelia gilioides* |  | S1 | S1 | S1 | S1 | S1 | S1 | S1 | 2 | 22 | 2 |
| *Phacelia purshii* | S1 | S5 | S4 | S4 | S4 | S4 | S4 | S4 | 179 | 89,419 | 159 |
| *Phacelia ranunculacea* |  | S2 | S2 | S3 | S2 | S3 | S2 | S3 | 23 | 24,981 | 19 |
| *Phacelia sp. nov.* |  |  |  |  |  |  |  |  |  |  |  |
| **BRASSICACEAE** |  |  |  |  |  |  |  |  |  |  |  |
| *Abdra brachycarpa* | S3 | S3 | S2 | S3 | S2 | S3 | S2 | S3 | 11 | 59,098 | 11 |
| *Arabidopsis lyrata* |  | S2 | S1 | S1 | S1 | S1 | S1 | S1 | 5 | 3,255 | 4 |
| *Arabis adpressipilis* | S1 | S1 |  |  |  |  |  |  |  |  |  |
| *Arabis patens* | S1 | S2 | S1 | S1 | S1 | S1 | S1 | S1 | 3 | 122 | 3 |
| *Borodinia burkii* |  | S1 |  |  |  |  |  |  |  |  |  |
| *Borodinia canadensis* | S4 | S3 | S2 | S2 | S2 | S2 | S2 | S2 | 4 | 30,213 | 4 |
| *Borodinia dentata* |  | S1 | S1 | S1 | S1 | S1 | S1 | S1 | 2 | 16 | 2 |
| *Borodinia laevigata* |  | S4 | S3 | S4 | S3 | S4 | S3 | S4 | 82 | 88,349 | 74 |
| *Borodinia perstellata* |  | S1 | S1 | S1 | S1 | S1 | S1 | S1 | 3 | 607 | 3 |
| *Cardamine angustata* |  | S5 | S3 | S3 | S3 | S3 | S3 | S3 | 55 | 79,401 | 55 |
| *Cardamine bulbosa* |  | S5 | S3 | S4 | S3 | S4 | S3 | S4 | 91 | 118,172 | 81 |
| *Cardamine clematitis* | S1 | S2 |  |  | S2 | S2 | S3 | S2 | 18 | 7,119 | 19 |
| *Cardamine concatenata* | S5 | S5 | S4 | S4 | S4 | S4 | S4 | S4 | 268 | 115,339 | 220 |
| *Cardamine diphylla* | S5 | S5 | S4 | S4 | S4 | S4 | S4 | S4 | 154 | 92,143 | 137 |
| *Cardamine dissecta* | S1 | S4 | S3 | S3 | S3 | S3 | S3 | S3 | 51 | 43,458 | 48 |
| *Cardamine douglassii* | S2 | S4 | S3 | S3 | S3 | S3 | S3 | S3 | 31 | 35,322 | 29 |
| *Cardamine flagellifera* | S3 | S2 | S2 | S2 | S2 | S2 | S2 | S2 | 8 | 6,580 | 7 |
| *Cardamine parviflora* |  | S5 | S2 | S3 | S2 | S3 | S2 | S3 | 7 | 33,553 | 7 |
| *Cardamine pensylvanica* | S5 | S5 | S2 | S3 | S3 | S3 | S2 | S3 | 23 | 78,989 | 20 |
| *Cardamine rotundifolia* |  | S3 |  |  | S3 | S3 | S3 | S3 | 28 | 14,275 | 35 |
| *Descurainia pinnata* |  | S1 | S1 | S1 | S1 | S1 | S1 | S1 | 1 | <1 | 1 |
| *Draba ramosissima* |  | S3 |  |  | S2 | S3 | S3 | S3 | 6 | 18,004 | 5 |
| *Erysimum capitatum* | S2 | S2 | S2 | S3 | S3 | S3 | S2 | S3 | 22 | 44,841 | 20 |
| *Iodanthus pinnatifidus* |  | S4 | S3 | S4 | S3 | S4 | S3 | S4 | 95 | 57,741 | 84 |
| *Leavenworthia exigua* | S2 | S3 | S2 | S2 | S2 | S2 | S2 | S2 | 18 | 13,378 | 18 |
| *Leavenworthia stylosa* |  | S3 | S3 | S3 | S3 | S3 | S3 | S3 | 91 | 3,289 | 67 |
| *Leavenworthia torulosa* |  | S3 | S3 | S3 | S3 | S3 | S3 | S3 | 32 | 6,192 | 30 |
| *Leavenworthia uniflora* |  | S3 | S3 | S3 | S3 | S3 | S3 | S3 | 33 | 26,118 | 31 |
| *Lepidium oblongum* |  | S1 | S1 | S1 | S1 | S1 | S1 | S1 | 1 | <1 | 1 |
| *Lepidium virginicum* | S5 | S5 | S3 | S3 | S3 | S3 | S3 | S3 | 74 | 110,996 | 72 |
| *Paysonia densipila* |  | S2 |  |  | S3 | S3 | S3 | S3 | 44 | 8,490 | 44 |
| *Paysonia lescurii* |  | S3 | S2 | S3 | S2 | S3 | S2 | S3 | 22 | 3,146 | 21 |
| *Physaria globosa* |  | S1 |  |  | S2 | S3 | S2 | S2 | 15 | 749 | 14 |
| *Planodes virginicum* | S4 | S3 | S2 | S3 | S2 | S3 | S2 | S3 | 9 | 50,626 | 9 |
| *Rorippa aquatica* | S1 | S2 | S1 | S1 | S1 | S1 | S1 | S1 | 2 | 1 | 2 |
| *Rorippa palustris* |  | S5 | S2 | S3 | S2 | S3 | S2 | S3 | 6 | 24,184 | 6 |
| *Rorippa sessiliflora* |  | S4 |  |  |  |  |  |  |  |  |  |
| *Rorippa sinuata* |  | S1 |  |  |  |  |  |  |  |  |  |
| *Tomostima cuneifolia* | S1 | S1 | S1 | S1 | S1 | S1 | S1 | S1 | 4 | 45 | 4 |
| *Tomostima reptans* |  | S2 |  |  |  |  |  |  |  |  |  |
| *Turritis glabra* | S1 | S1 |  |  | S1 | S1 | S1 | S1 | 3 | 185 | 3 |
| **BUXACEAE** |  |  |  |  |  |  |  |  |  |  |  |
| *Pachysandra procumbens* | S1 | S5 | S4 | S4 | S4 | S4 | S4 | S4 | 134 | 89,463 | 116 |
| **CACTACEAE** |  |  |  |  |  |  |  |  |  |  |  |
| *Opuntia humifusa* | S5 | S4 | S3 | S4 | S3 | S4 | S3 | S4 | 116 | 89,570 | 103 |
| **CAMPANULACEAE** |  |  |  |  |  |  |  |  |  |  |  |
| *Campanula americana* | S4 | S5 | S4 | S4 | S4 | S4 | S4 | S4 | 234 | 127,477 | 219 |
| *Campanula aparinoides* |  | S2 | S1 | S2 | S1 | S2 | S1 | S2 | 3 | 6,427 | 3 |
| *Campanula divaricata* | S5 | S4 | S4 | S4 | S4 | S4 | S4 | S4 | 126 | 60,211 | 115 |
| *Campanula rotundifolia* | S1 | S1 | S1 | S1 | S1 | S1 | S1 | S1 | 1 | <1 | 1 |
| *Lobelia amoena* |  | S1 |  |  | S2 | S1 | S2 | S1 | 6 | 283 | 6 |
| *Lobelia canbyi* | S3 | S2 | S2 | S2 | S2 | S2 | S2 | S2 | 12 | 2,092 | 12 |
| *Lobelia cardinalis* | S5 | S5 | S4 | S5 | S4 | S5 | S4 | S5 | 530 | 133,162 | 478 |
| *Lobelia gattingeri* |  | S3 | S3 | S3 | S3 | S3 | S3 | S3 | 50 | 7,748 | 43 |
| *Lobelia inflata* | S5 | S5 | S4 | S4 | S4 | S4 | S4 | S4 | 176 | 111,883 | 166 |
| *Lobelia nuttallii* |  | S3 | S2 | S1 | S2 | S1 | S2 | S1 | 6 | 610 | 6 |
| *Lobelia puberula* | S5 | S5 | S4 | S4 | S4 | S4 | S4 | S4 | 179 | 113,155 | 162 |
| *Lobelia siphilitica* |  | S5 | S4 | S5 | S4 | S5 | S4 | S5 | 335 | 125,813 | 315 |
| *Lobelia spicata* | S5 | S5 | S3 | S3 | S3 | S3 | S3 | S3 | 42 | 80,632 | 40 |
| *Triodanis biflora* |  | S3 | S3 | S3 | S3 | S3 | S3 | S3 | 40 | 88,475 | 40 |
| *Triodanis holzingeri* |  | S1 |  |  |  |  |  |  |  |  |  |
| *Triodanis perfoliata* | S5 | S5 | S4 | S4 | S4 | S4 | S4 | S4 | 154 | 119,908 | 151 |
| **CANNABACEAE** |  |  |  |  |  |  |  |  |  |  |  |
| *Celtis laevigata* | S5 | S5 | S3 | S3 | S3 | S3 | S3 | S3 | 76 | 114,941 | 70 |
| *Celtis occidentalis* | S5 | S5 | S3 | S3 | S3 | S3 | S3 | S3 | 77 | 113,114 | 71 |
| *Celtis pumila* |  | S4 | S2 | S3 | S2 | S3 | S2 | S3 | 5 | 23,736 | 5 |
| *Celtis smallii* |  | S1 | S1 | S1 | S1 | S1 | S1 | S1 | 1 | <1 | 1 |
| **CAPRIFOLIACEAE** |  |  |  |  |  |  |  |  |  |  |  |
| *Lonicera canadensis* |  | S3 |  |  | S1 | S1 | S2 | S1 | 3 | 2,318 | 3 |
| *Lonicera dioica* |  | S3 |  |  | S3 | S3 | S3 | S3 | 27 | 38,907 | 29 |
| *Lonicera flava* | S3 | S2 |  |  | S1 | S2 | S2 | S2 | 2 | <1 | 2 |
| *Lonicera reticulata* |  | S1 |  |  | S1 | S1 | S1 | S1 | 1 | <1 | 1 |
| *Lonicera sempervirens* | S5 | S5 | S4 | S4 | S3 | S4 | S4 | S4 | 127 | 111,264 | 116 |
| *Symphoricarpos orbiculatus* |  | S5 | S4 | S4 | S4 | S4 | S4 | S4 | 202 | 96,079 | 187 |
| *Triosteum angustifolium* |  | S4 | S2 | S2 | S2 | S2 | S2 | S2 | 6 | 14,357 | 6 |
| *Triosteum aurantiacum* | S2 | S4 | S2 | S3 | S2 | S2 | S2 | S3 | 7 | 20,124 | 7 |
| *Triosteum perfoliatum* | S3 | S3 |  |  |  |  |  |  |  |  |  |
| **CARYOPHYLLACEAE** |  |  |  |  |  |  |  |  |  |  |  |
| *Arenaria lanuginosa* |  | S1 | S1 | S1 | S1 | S1 | S1 | S1 | 1 | <1 | 1 |
| *Cerastium brachypodum* |  | S2 | S1 | S1 | S1 | S1 | S1 | S1 | 1 | <1 | 1 |
| *Cerastium nutans* | S5 | S5 | S2 | S3 | S2 | S3 | S2 | S3 | 9 | 43,577 | 9 |
| *Cerastium velutinum* | S1 | S1 | S1 | S1 | S1 | S1 | S1 | S1 | 1 | <1 | 1 |
| *Geocarpon cumberlandense* |  | S1 | S2 | S2 | S2 | S2 | S2 | S2 | 27 | 687 | 27 |
| *Geocarpon glabrum* |  | S1 | S1 | S1 | S1 | S1 | S1 | S1 | 5 | 3,525 | 5 |
| *Geocarpon groenlandicum* |  | S1 |  |  | S1 | S1 | S1 | S1 | 2 | <1 | 2 |
| *Paronychia argyrocoma* | S1 | S2 |  |  | S2 | S2 | S2 | S3 | 7 | 13,640 | 7 |
| *Paronychia canadensis* | S4 | S4 | S2 | S3 | S2 | S3 | S2 | S3 | 7 | 34,692 | 7 |
| *Paronychia fastigiata* |  | S3 | S1 | S1 | S1 | S1 | S1 | S1 | 1 | <1 | 1 |
| *Paronychia montana* |  |  |  |  |  |  |  |  |  |  |  |
| *Paronychia virginica* | S1 | S1 | S1 | S1 | S1 | S1 | S1 | S1 | 1 | <1 | 1 |
| *Sabulina diffusa* |  |  |  |  |  |  |  |  |  |  |  |
| *Sabulina fontinalis* | S1 | S2 | S3 | S3 | S3 | S3 | S3 | S3 | 21 | 12,739 | 21 |
| *Sabulina muscorum* |  | S1 | S1 | S1 | S1 | S1 | S1 | S1 | 1 | <1 | 1 |
| *Sabulina patula* | S3 | S3 | S3 | S4 | S3 | S4 | S3 | S4 | 18 | 26,240 | 18 |
| *Sagina decumbens* |  | S4 | S2 | S3 | S2 | S3 | S2 | S3 | 6 | 25,027 | 6 |
| *Silene antirrhina* | S5 | S5 | S2 | S3 | S2 | S3 | S2 | S3 | 8 | 45,151 | 8 |
| *Silene caroliniana* | S2 | S1 | S1 | S1 | S1 | S1 | S1 | S1 | 3 | 170 | 3 |
| *Silene ovata* | S1 | S3 |  |  | S2 | S3 | S3 | S3 | 8 | 32,115 | 8 |
| *Silene rotundifolia* | S1 | S3 | S3 | S3 | S3 | S3 | S3 | S3 | 36 | 52,066 | 34 |
| *Silene stellata* |  | S5 | S3 | S4 | S3 | S3 | S3 | S4 | 84 | 114,495 | 81 |
| *Silene virginica* | S5 | S5 | S4 | S5 | S4 | S5 | S4 | S5 | 541 | 119,959 | 469 |
| *Stellaria alsine* |  | S1 |  |  | S1 | S1 | S1 | S1 | 1 | <1 | 1 |
| *Stellaria corei* |  | S4 | S3 | S3 | S3 | S3 | S3 | S3 | 43 | 62,502 | 36 |
| *Stellaria longifolia* |  | S1 |  |  | S1 | S1 | S1 | S1 | 2 | 8 | 3 |
| *Stellaria pubera* | S5 | S5 | S4 | S4 | S4 | S5 | S4 | S5 | 340 | 98,644 | 287 |
| **CELASTRACEAE** |  |  |  |  |  |  |  |  |  |  |  |
| *Celastrus scandens* | S2 | S4 | S2 | S3 | S2 | S3 | S2 | S3 | 10 | 52,986 | 10 |
| *Euonymus americanus* | S5 | S5 | S4 | S5 | S4 | S5 | S4 | S5 | 614 | 122,674 | 515 |
| *Euonymus atropurpureus* |  | S5 | S3 | S3 | S3 | S3 | S3 | S3 | 35 | 59,046 | 32 |
| *Euonymus obovatus* | S3 | S3 | S2 | S2 | S2 | S2 | S2 | S2 | 12 | 3,520 | 10 |
| *Paxistima canbyi* |  | S1 |  |  | S1 | S1 | S1 | S1 | 1 | <1 | 1 |
| **CISTACEAE** |  |  |  |  |  |  |  |  |  |  |  |
| *Crocanthemum canadense* | S1 | S1 |  |  | S1 | S1 | S1 | S1 | 2 | 62 | 2 |
| *Crocanthemum propinquum* | S1 | S1 |  |  | S2 | S1 | S2 | S2 | 7 | 739 | 7 |
| *Lechea minor* | S4 | S4 | S2 | S2 | S2 | S2 | S2 | S2 | 6 | 5,311 | 6 |
| *Lechea mucronata* | S4 | S4 | S2 | S3 | S2 | S3 | S2 | S3 | 14 | 29,801 | 14 |
| *Lechea pulchella* |  | S1 |  |  | S1 | S1 | S1 | S1 | 3 | 24 | 3 |
| *Lechea racemulosa* | S5 | S5 | S2 | S2 | S2 | S2 | S2 | S2 | 6 | 8,069 | 6 |
| *Lechea tenuifolia* | S4 | S4 | S2 | S2 | S2 | S2 | S2 | S2 | 6 | 10,076 | 6 |
| **CLETHRACEAE** |  |  |  |  |  |  |  |  |  |  |  |
| *Clethra acuminata* | S4 | S4 | S3 | S3 | S3 | S3 | S3 | S3 | 59 | 30,660 | 59 |
| *Clethra alnifolia* | S5 | S1 | S1 | S1 | S1 | S1 | S1 | S1 | 2 | 69 | 2 |
| **CONVOLVULACEAE** |  |  |  |  |  |  |  |  |  |  |  |
| *Convolvulus catesbeiana* | S1 | S1 | S2 | S3 | S2 | S3 | S2 | S3 | 11 | 37,437 | 11 |
| *Convolvulus fraterniflorus* |  | S3 | S1 | S1 | S1 | S1 | S1 | S1 | 2 | 91 | 2 |
| *Convolvulus sepium* |  | S4 | S1 | S1 | S1 | S1 | S1 | S1 | 1 | <1 | 1 |
| *Convolvulus spithamaeus* |  | S1 | S1 | S1 | S1 | S1 | S1 | S2 | 4 | 4,828 | 5 |
| *Cuscuta campestris* |  | S5 | S2 | S3 | S2 | S3 | S2 | S3 | 13 | 48,996 | 13 |
| *Cuscuta cephalanthi* | S1 | S1 |  |  |  |  |  |  |  |  |  |
| *Cuscuta compacta* | S5 | S4 | S1 | S2 | S1 | S2 | S1 | S2 | 4 | 15,360 | 4 |
| *Cuscuta coryli* |  | S1 | S1 | S1 | S1 | S1 | S1 | S1 | 1 | <1 | 1 |
| *Cuscuta cuspidata* |  | S3 | S1 | S1 | S1 | S1 | S1 | S1 | 1 | <1 | 1 |
| *Cuscuta glomerata* |  | S1 |  |  |  |  |  |  |  |  |  |
| *Cuscuta gronovii* | S5 | S5 | S2 | S3 | S2 | S3 | S2 | S3 | 20 | 67,106 | 20 |
| *Cuscuta indecora* |  | S1 | S1 | S1 | S1 | S1 | S1 | S1 | 1 | <1 | 1 |
| *Cuscuta pentagona* |  | S4 | S2 | S3 | S2 | S3 | S2 | S3 | 10 | 25,080 | 10 |
| *Cuscuta polygonorum* |  | S1 |  |  |  |  |  |  |  |  |  |
| *Cuscuta rostrata* | S3 | S2 | S2 | S2 | S2 | S2 | S2 | S2 | 12 | 3,797 | 11 |
| *Cuscuta saururi* |  |  |  |  |  |  |  |  |  |  |  |
| *Evolvulus nuttallianus* |  | S1 |  |  | S2 | S2 | S2 | S2 | 12 | 576 | 11 |
| *Ipomoea coccinea* |  | S5 | S3 | S4 | S3 | S4 | S3 | S4 | 97 | 106,567 | 94 |
| *Ipomoea lacunosa* | S5 | S5 | S3 | S3 | S3 | S3 | S3 | S3 | 68 | 120,171 | 68 |
| *Ipomoea pandurata* | S5 | S5 | S4 | S4 | S4 | S4 | S4 | S4 | 300 | 126,163 | 273 |
| *Stylisma humistrata* | S3 | S1 | S1 | S1 | S1 | S1 | S1 | S1 | 1 | <1 | 1 |
| **CORNACEAE** |  |  |  |  |  |  |  |  |  |  |  |
| *Cornus alternifolia* |  | S5 | S3 | S3 | S3 | S3 | S3 | S3 | 52 | 51,696 | 47 |
| *Cornus amomum* | S5 | S5 | S3 | S3 | S3 | S3 | S3 | S3 | 43 | 110,113 | 43 |
| *Cornus drummondii* |  | S4 | S3 | S3 | S3 | S3 | S3 | S3 | 39 | 77,139 | 39 |
| *Cornus florida* | S5 | S5 | S4 | S5 | S4 | S5 | S4 | S5 | 643 | 133,958 | 598 |
| *Cornus foemina* | S5 | S5 | S2 | S3 | S2 | S3 | S2 | S3 | 15 | 66,086 | 15 |
| *Cornus obliqua* |  | S2 | S2 | S3 | S2 | S3 | S2 | S3 | 13 | 29,115 | 13 |
| *Cornus racemosa* |  | S2 | S1 | S1 | S1 | S1 | S1 | S1 | 2 | 78 | 2 |
| *Cornus rugosa* |  | S1 |  |  |  |  |  |  |  |  |  |
| **CRASSULACEAE** |  |  |  |  |  |  |  |  |  |  |  |
| *Diamorpha smallii* | S3 | S2 |  |  | S2 | S2 | S3 | S2 | 9 | 3,480 | 10 |
| *Sedum nevii* |  | S2 |  |  | S2 | S1 | S2 | S1 | 6 | 257 | 9 |
| *Sedum pulchellum* | S1 | S5 | S4 | S4 | S4 | S4 | S4 | S4 | 245 | 86,098 | 224 |
| *Sedum ternatum* | S5 | S5 | S4 | S5 | S4 | S5 | S4 | S5 | 399 | 96,292 | 333 |
| **CUCURBITACEAE** |  |  |  |  |  |  |  |  |  |  |  |
| *Cayaponia quinqueloba* | S1 | S1 | S1 | S1 | S1 | S1 | S1 | S1 | 1 | <1 | 1 |
| *Melothria pendula* | S4 | S5 | S3 | S3 | S3 | S3 | S3 | S3 | 77 | 106,518 | 76 |
| *Sicyos angulatus* | S5 | S5 | S3 | S4 | S3 | S4 | S3 | S4 | 107 | 121,398 | 103 |
| **DIAPENSIACEAE** |  |  |  |  |  |  |  |  |  |  |  |
| *Galax urceolata* | S5 | S3 | S4 | S4 | S4 | S4 | S4 | S4 | 228 | 29,851 | 179 |
| **DIERVILLACEAE** |  |  |  |  |  |  |  |  |  |  |  |
| *Diervilla lonicera* |  | S3 |  |  | S2 | S3 | S3 | S3 | 16 | 23,467 | 16 |
| *Diervilla rivularis* |  | S2 | S1 | S2 | S1 | S1 | S1 | S2 | 5 | 5,053 | 5 |
| *Diervilla sessilifolia* |  | S3 | S2 | S2 | S2 | S2 | S2 | S2 | 18 | 4,039 | 17 |
| **DROSERACEAE** |  |  |  |  |  |  |  |  |  |  |  |
| *Drosera brevifolia* | S3 | S2 | S3 | S3 | S3 | S3 | S3 | S3 | 35 | 12,255 | 35 |
| *Drosera capillaris* |  | S1 | S1 | S1 | S1 | S1 | S1 | S1 | 4 | 474 | 4 |
| *Drosera intermedia* | S4 | S3 | S2 | S2 | S2 | S2 | S2 | S2 | 14 | 4,407 | 14 |
| *Drosera rotundifolia* |  | S1 | S1 | S1 | S1 | S1 | S1 | S1 | 3 | 34 | 3 |
| **EBENACEAE** |  |  |  |  |  |  |  |  |  |  |  |
| *Diospyros virginiana* | S5 | S3 | S4 | S5 | S4 | S5 | S4 | S5 | 487 | 126,777 | 455 |
| **ELATINACEAE** |  |  |  |  |  |  |  |  |  |  |  |
| *Elatine minima* |  | S1 | S1 | S1 | S1 | S1 | S1 | S1 | 1 | <1 | 1 |
| **ERICACEAE** |  |  |  |  |  |  |  |  |  |  |  |
| *Chimaphila maculata* | S5 | S5 | S4 | S5 | S4 | S5 | S4 | S5 | 799 | 107,720 | 671 |
| *Epigaea repens* | S5 | S5 | S4 | S4 | S4 | S4 | S4 | S4 | 172 | 50,254 | 140 |
| *Eubotrys racemosus* | S5 | S2 | S2 | S2 | S2 | S2 | S2 | S2 | 9 | 2,771 | 8 |
| *Eubotrys recurvus* |  | S3 | S2 | S2 | S2 | S2 | S2 | S2 | 13 | 1,865 | 13 |
| *Gaultheria procumbens* |  | S4 | S3 | S4 | S3 | S4 | S3 | S4 | 114 | 55,494 | 107 |
| *Gaylussacia baccata* | S5 | S4 | S3 | S3 | S3 | S3 | S3 | S3 | 22 | 36,602 | 22 |
| *Gaylussacia brachycera* | S1 | S2 | S2 | S1 | S2 | S1 | S2 | S1 | 14 | 707 | 14 |
| *Gaylussacia dumosa* |  | S2 |  |  | S3 | S3 | S3 | S3 | 30 | 1,885 | 36 |
| *Gaylussacia frondosa* | S5 | S2 | S1 | S1 | S1 | S1 | S1 | S1 | 2 | 38 | 2 |
| *Gaylussacia ursina* |  | S3 | S3 | S3 | S3 | S3 | S3 | S3 | 23 | 12,749 | 24 |
| *Hypopitys lanuginosa* |  | S5 | S2 | S3 | S4 | S4 | S2 | S3 | 8 | 34,094 | 8 |
| *Kalmia buxifolia* |  | S1 | S3 | S3 | S2 | S3 | S3 | S3 | 28 | 2,887 | 22 |
| *Kalmia latifolia* | S5 | S5 | S4 | S5 | S4 | S5 | S4 | S5 | 465 | 87,601 | 384 |
| *Leucothoe fontanesiana* |  | S4 | S4 | S4 | S4 | S4 | S4 | S4 | 146 | 29,304 | 109 |
| *Lyonia ligustrina* | S5 | S4 | S3 | S3 | S3 | S3 | S3 | S3 | 34 | 38,328 | 32 |
| *Monotropa uniflora* |  | S5 | S4 | S4 | S4 | S4 | S4 | S4 | 266 | 123,450 | 232 |
| *Monotropsis odorata* |  | S3 |  |  | S3 | S3 | S3 | S3 | 32 | 15,988 | 32 |
| *Oxydendrum arboreum* | S5 | S5 | S4 | S4 | S4 | S4 | S4 | S4 | 288 | 123,470 | 270 |
| *Pieris floribunda* |  | S1 |  |  | S2 | S2 | S2 | S2 | 6 | 133 | 6 |
| *Pyrola americana* |  | S1 |  |  | S2 | S1 | S2 | S1 | 16 | 266 | 14 |
| *Rhododendron alabamense* |  | S3 | S2 | S2 | S2 | S2 | S2 | S2 | 17 | 7,995 | 14 |
| *Rhododendron arborescens* |  | S4 | S2 | S2 | S2 | S2 | S2 | S3 | 10 | 16,783 | 10 |
| *Rhododendron calendulaceum* | S5 | S4 | S4 | S4 | S4 | S4 | S4 | S4 | 173 | 61,092 | 153 |
| *Rhododendron canescens* |  | S5 | S3 | S3 | S3 | S3 | S3 | S3 | 51 | 63,587 | 49 |
| *Rhododendron carolinianum* |  | S2 | S2 | S1 | S2 | S1 | S2 | S1 | 7 | 370 | 6 |
| *Rhododendron catawbiense* | S5 | S4 | S3 | S3 | S3 | S3 | S3 | S3 | 51 | 32,398 | 45 |
| *Rhododendron cumberlandense* | S3 | S4 | S2 | S2 | S2 | S2 | S2 | S2 | 9 | 8,388 | 9 |
| *Rhododendron maximum* | S5 | S5 | S4 | S4 | S4 | S5 | S4 | S5 | 301 | 52,398 | 233 |
| *Rhododendron minus* |  | S3 | S2 | S2 | S2 | S2 | S2 | S2 | 18 | 14,569 | 20 |
| *Rhododendron periclymenoides* | S5 | S4 | S3 | S3 | S3 | S3 | S3 | S3 | 28 | 56,823 | 27 |
| *Rhododendron pilosum* |  | S1 | S1 | S1 | S1 | S1 | S1 | S1 | 4 | 86 | 4 |
| *Rhododendron prinophyllum* |  | S2 | S1 | S1 | S1 | S1 | S1 | S1 | 1 | <1 | 1 |
| *Rhododendron smokianum* |  | S1 | S1 | S1 | S1 | S1 | S1 | S1 | 14 | 85 | 9 |
| *Rhododendron viscosum* |  | S3 | S1 | S1 | S1 | S1 | S1 | S1 | 2 | 41 | 2 |
| *Vaccinium altomontanum* |  | S1 | S1 | S1 | S1 | S1 | S1 | S1 | 2 | 1 | 2 |
| *Vaccinium angustifolium* |  | S1 | S1 | S2 | S1 | S2 | S1 | S2 | 4 | 12,380 | 4 |
| *Vaccinium arboreum* | S5 | S5 | S3 | S4 | S3 | S4 | S3 | S4 | 107 | 90,573 | 99 |
| *Vaccinium corymbosum* | S5 | S5 | S3 | S3 | S3 | S3 | S3 | S3 | 49 | 60,004 | 49 |
| *Vaccinium elliottii* |  | S1 |  |  | S1 | S1 | S1 | S1 | 1 | <1 | 1 |
| *Vaccinium erythrocarpum* |  | S3 | S3 | S3 | S3 | S3 | S3 | S3 | 26 | 17,311 | 25 |
| *Vaccinium fuscatum* | S5 | S4 | S1 | S1 | S1 | S1 | S1 | S1 | 1 | <1 | 1 |
| *Vaccinium hirsutum* |  | S2 | S3 | S3 | S3 | S3 | S3 | S3 | 26 | 6,650 | 22 |
| *Vaccinium macrocarpon* | S2 | S1 |  |  | S2 | S2 | S2 | S3 | 10 | 19,506 | 10 |
| *Vaccinium pallidum* | S5 | S5 | S3 | S3 | S3 | S3 | S3 | S3 | 56 | 79,423 | 55 |
| *Vaccinium simulatum* | S4 | S3 | S2 | S2 | S2 | S2 | S2 | S2 | 13 | 6,932 | 12 |
| *Vaccinium stamineum* | S5 | S5 | S4 | S4 | S4 | S4 | S4 | S4 | 132 | 107,287 | 126 |
| **EUPHORBIACEAE** |  |  |  |  |  |  |  |  |  |  |  |
| *Acalypha deamii* | S3 | S2 | S2 | S2 | S2 | S2 | S2 | S2 | 10 | 13,897 | 10 |
| *Acalypha gracilens* | S5 | S5 | S2 | S3 | S2 | S3 | S2 | S3 | 7 | 21,746 | 7 |
| *Acalypha ostryifolia* |  | S4 | S3 | S3 | S3 | S3 | S3 | S3 | 31 | 79,263 | 30 |
| *Acalypha rhomboidea* | S5 | S5 | S3 | S3 | S3 | S3 | S3 | S3 | 49 | 103,173 | 49 |
| *Acalypha virginica* |  | S5 | S2 | S3 | S2 | S3 | S2 | S3 | 14 | 61,440 | 14 |
| *Croton capitatus* |  | S4 | S3 | S3 | S3 | S3 | S3 | S3 | 29 | 41,510 | 27 |
| *Croton glandulosus* | S5 | S4 | S2 | S3 | S2 | S3 | S2 | S3 | 16 | 72,478 | 15 |
| *Croton lindheimeri* |  | S2 | S2 | S3 | S3 | S3 | S2 | S3 | 16 | 30,686 | 14 |
| *Croton michauxii* | S1 | S1 | S2 | S2 | S2 | S2 | S2 | S2 | 10 | 7,165 | 10 |
| *Croton monanthogynus* | S3 | S5 | S3 | S3 | S3 | S3 | S3 | S3 | 77 | 79,006 | 70 |
| *Croton willdenowii* | S3 | S3 | S1 | S1 | S1 | S1 | S1 | S1 | 1 | <1 | 1 |
| *Euphorbia commutata* | S4 | S5 | S3 | S3 | S3 | S3 | S3 | S3 | 33 | 68,331 | 31 |
| *Euphorbia corollata* | S5 | S5 | S4 | S4 | S4 | S4 | S4 | S4 | 279 | 112,355 | 259 |
| *Euphorbia cyathophora* |  | S2 | S1 | S2 | S1 | S2 | S1 | S2 | 4 | 14,220 | 4 |
| *Euphorbia dentata* |  | S5 | S3 | S3 | S3 | S3 | S3 | S3 | 50 | 66,733 | 47 |
| *Euphorbia glyptosperma* |  | S1 |  |  |  |  |  |  |  |  |  |
| *Euphorbia humistrata* |  | S4 | S2 | S3 | S2 | S3 | S2 | S3 | 12 | 40,220 | 12 |
| *Euphorbia maculata* | S5 | S5 | S3 | S4 | S3 | S4 | S3 | S4 | 121 | 117,842 | 115 |
| *Euphorbia marginata* |  | S3 | S1 | S1 | S1 | S1 | S1 | S1 | 2 | 28 | 2 |
| *Euphorbia mercurialina* | S3 | S5 | S3 | S4 | S3 | S4 | S4 | S4 | 121 | 50,275 | 114 |
| *Euphorbia nutans* | S5 | S5 | S3 | S4 | S4 | S4 | S3 | S4 | 123 | 114,331 | 118 |
| *Euphorbia ouachitana* |  | S1 | S1 | S1 | S1 | S1 | S1 | S1 | 5 | 602 | 4 |
| *Euphorbia pubentissima* |  | S4 | S2 | S3 | S2 | S3 | S2 | S3 | 16 | 40,588 | 16 |
| *Euphorbia serpens* |  | S1 | S1 | S1 | S1 | S1 | S1 | S1 | 2 | 37 | 2 |
| *Euphorbia sp. nov. aff. pubentissima* |  |  |  |  |  |  |  |  |  |  |  |
| *Euphorbia spathulata* |  | S3 | S2 | S2 | S2 | S2 | S2 | S2 | 9 | 2,145 | 9 |
| *Tragia betonicifolia* |  | S2 | S1 | S1 | S1 | S1 | S1 | S1 | 3 | 118 | 2 |
| *Tragia cordata* | S2 | S4 | S2 | S2 | S2 | S2 | S2 | S3 | 18 | 19,653 | 18 |
| *Tragia urticifolia* | S3 | S1 | S1 | S1 | S1 | S1 | S1 | S1 | 1 | <1 | 1 |
| **FABACEAE** |  |  |  |  |  |  |  |  |  |  |  |
| *Aeschynomene indica* |  | S1 | S1 | S1 | S1 | S1 | S1 | S1 | 3 | 2,777 | 3 |
| *Amorpha fruticosa* | S3 | S5 | S3 | S3 | S3 | S3 | S3 | S3 | 79 | 123,396 | 75 |
| *Amorpha glabra* | S3 | S3 | S1 | S1 | S1 | S1 | S1 | S1 | 5 | 176 | 5 |
| *Amorpha nitens* | S1 | S4 |  |  |  |  |  |  |  |  |  |
| *Amphicarpaea bracteata* | S5 | S5 | S4 | S4 | S4 | S4 | S4 | S4 | 167 | 107,112 | 155 |
| *Apios americana* | S5 | S5 | S3 | S4 | S3 | S4 | S3 | S4 | 99 | 123,248 | 98 |
| *Apios priceana* | S1 | S3 |  |  | S3 | S3 | S3 | S3 | 30 | 30,829 | 29 |
| *Astragalus canadensis* | S1 | S4 | S2 | S3 | S2 | S3 | S2 | S3 | 18 | 74,369 | 16 |
| *Astragalus tennesseensis* |  | S2 | S3 | S3 | S3 | S3 | S3 | S3 | 73 | 4,140 | 63 |
| *Baptisia aberrans* |  | S3 | S2 | S2 | S2 | S2 | S2 | S2 | 6 | 15,393 | 5 |
| *Baptisia albescens* |  | S1 | S2 | S3 | S2 | S3 | S2 | S3 | 13 | 63,981 | 13 |
| *Baptisia australis* |  | S3 | S3 | S3 | S3 | S3 | S3 | S3 | 38 | 97,723 | 37 |
| *Baptisia lactea* |  | S1 | S1 | S1 | S1 | S1 | S1 | S1 | 1 | <1 | 1 |
| *Baptisia leucophaea* |  | S1 |  |  | S1 | S1 | S1 | S1 | 1 | <1 | 1 |
| *Baptisia tinctoria* |  | S4 | S3 | S3 | S3 | S3 | S3 | S3 | 29 | 36,435 | 30 |
| *Centrosema virginianum* | S5 | S4 | S3 | S3 | S3 | S3 | S3 | S3 | 32 | 71,559 | 31 |
| *Cercis canadensis* | S5 | S5 | S4 | S5 | S4 | S5 | S4 | S5 | 787 | 131,430 | 692 |
| *Chamaecrista fasciculata* | S5 | S5 | S4 | S5 | S4 | S5 | S4 | S5 | 324 | 125,518 | 310 |
| *Chamaecrista nictitans* | S5 | S4 | S3 | S4 | S3 | S4 | S3 | S4 | 107 | 117,760 | 100 |
| *Cladrastis kentukea* | S3 | S4 | S3 | S3 | S3 | S3 | S3 | S3 | 59 | 94,126 | 56 |
| *Clitoria mariana* | S5 | S5 | S3 | S4 | S3 | S4 | S3 | S4 | 101 | 94,034 | 101 |
| *Crotalaria sagittalis* | S3 | S4 | S2 | S3 | S2 | S3 | S2 | S3 | 13 | 25,190 | 13 |
| *Dalea candida* |  | S2 | S2 | S2 | S2 | S2 | S2 | S2 | 14 | 6,335 | 12 |
| *Dalea foliosa* |  | S2 | S3 | S3 | S3 | S3 | S3 | S3 | 32 | 4,377 | 32 |
| *Dalea gattingeri* | S2 | S3 | S3 | S3 | S3 | S3 | S3 | S3 | 52 | 11,119 | 45 |
| *Dalea leporina* |  | S1 | S1 | S1 | S1 | S1 | S1 | S1 | 1 | <1 | 1 |
| *Dalea purpurea* |  | S1 | S1 | S1 | S1 | S1 | S1 | S1 | 2 | 8 | 2 |
| *Desmanthus illinoensis* |  | S4 | S3 | S3 | S3 | S3 | S3 | S3 | 73 | 98,865 | 66 |
| *Desmodium canescens* | S4 | S5 | S3 | S3 | S3 | S3 | S3 | S3 | 24 | 101,452 | 24 |
| *Desmodium ciliare* | S5 | S5 | S3 | S3 | S3 | S3 | S3 | S3 | 31 | 68,055 | 30 |
| *Desmodium cuspidatum* | S2 | S4 | S1 | S2 | S1 | S2 | S2 | S2 | 4 | 17,894 | 4 |
| *Desmodium glabellum* | S5 | S5 | S3 | S3 | S3 | S3 | S3 | S3 | 24 | 68,096 | 24 |
| *Desmodium laevigatum* | S5 | S4 | S2 | S3 | S2 | S3 | S2 | S3 | 16 | 71,737 | 16 |
| *Desmodium marilandicum* | S5 | S4 | S2 | S2 | S2 | S2 | S2 | S2 | 7 | 14,153 | 7 |
| *Desmodium nuttallii* | S3 | S4 | S2 | S3 | S2 | S3 | S2 | S3 | 8 | 25,548 | 8 |
| *Desmodium obtusum* |  | S4 | S2 | S2 | S2 | S2 | S2 | S2 | 7 | 13,234 | 7 |
| *Desmodium ochroleucum* | S1 | S2 |  |  | S1 | S2 | S2 | S2 | 3 | 9 | 3 |
| *Desmodium paniculatum* | S5 | S5 | S3 | S3 | S3 | S3 | S3 | S3 | 62 | 114,925 | 60 |
| *Desmodium perplexum* | S5 | S5 | S3 | S3 | S3 | S3 | S3 | S3 | 23 | 63,596 | 23 |
| *Desmodium rotundifolium* | S5 | S5 | S3 | S3 | S3 | S3 | S3 | S3 | 80 | 97,948 | 77 |
| *Desmodium sessilifolium* |  | S2 | S1 | S1 | S1 | S1 | S1 | S1 | 4 | 565 | 4 |
| *Desmodium viridiflorum* | S4 | S4 | S2 | S3 | S2 | S3 | S2 | S3 | 17 | 52,802 | 17 |
| *Galactia regularis* |  | S4 | S3 | S3 | S3 | S3 | S3 | S3 | 23 | 56,228 | 23 |
| *Galactia volubilis* | S5 | S5 | S2 | S3 | S2 | S3 | S2 | S3 | 8 | 25,548 | 7 |
| *Gleditsia aquatica* |  | S1 | S1 | S1 | S1 | S1 | S1 | S1 | 5 | 29 | 4 |
| *Gleditsia triacanthos* | S5 | S5 | S4 | S4 | S4 | S4 | S4 | S4 | 200 | 123,179 | 187 |
| *Gymnocladus dioicus* |  | S4 | S3 | S3 | S3 | S3 | S3 | S3 | 30 | 90,412 | 29 |
| *Hylodesmum glutinosum* |  | S3 | S3 | S3 | S3 | S3 | S3 | S3 | 58 | 94,322 | 54 |
| *Hylodesmum nudiflorum* | S5 | S4 | S4 | S4 | S4 | S4 | S4 | S4 | 215 | 112,709 | 190 |
| *Hylodesmum pauciflorum* |  | S4 | S3 | S3 | S3 | S3 | S3 | S3 | 29 | 54,891 | 28 |
| *Lackeya multiflora* |  | S2 | S1 | S2 | S1 | S2 | S1 | S2 | 3 | 8,556 | 3 |
| *Lathyrus palustris* | S1 | S2 | S1 | S2 | S1 | S2 | S1 | S2 | 4 | 12,488 | 4 |
| *Lathyrus venosus* | S3 | S3 | S1 | S1 | S1 | S1 | S1 | S1 | 2 | 49 | 2 |
| *Lespedeza angustifolia* | S4 | S2 | S2 | S2 | S2 | S2 | S2 | S2 | 6 | 2,384 | 6 |
| *Lespedeza capitata* |  | S4 | S3 | S3 | S3 | S3 | S3 | S3 | 28 | 46,560 | 26 |
| *Lespedeza frutescens* | S4 | S3 | S1 | S1 | S1 | S1 | S1 | S1 | 1 | <1 | 1 |
| *Lespedeza hirta* | S5 | S5 | S3 | S3 | S3 | S3 | S3 | S3 | 55 | 67,286 | 52 |
| *Lespedeza procumbens* | S5 | S5 | S3 | S3 | S3 | S3 | S3 | S3 | 67 | 81,018 | 65 |
| *Lespedeza repens* | S5 | S5 | S3 | S3 | S3 | S3 | S3 | S3 | 29 | 52,521 | 26 |
| *Lespedeza stuevei* | S4 | S4 | S1 | S1 | S1 | S1 | S1 | S1 | 3 | 1,817 | 3 |
| *Lespedeza violacea* | S4 | S5 | S3 | S3 | S3 | S3 | S3 | S3 | 35 | 71,525 | 33 |
| *Lespedeza virginica* | S5 | S5 | S3 | S3 | S3 | S3 | S3 | S3 | 71 | 81,315 | 66 |
| *Mimosa microphylla* | S4 | S3 | S1 | S1 | S2 | S3 | S1 | S1 | 3 | 1,326 | 3 |
| *Orbexilum onobrychis* |  | S2 | S1 | S1 | S1 | S1 | S1 | S1 | 1 | <1 | 1 |
| *Orbexilum pedunculatum* | S4 | S4 | S3 | S3 | S3 | S3 | S3 | S3 | 37 | 71,451 | 38 |
| *Pediomelum subacaule* | S2 | S2 | S3 | S3 | S3 | S4 | S3 | S4 | 85 | 11,849 | 69 |
| *Phaseolus polystachios* | S4 | S4 | S2 | S3 | S2 | S3 | S2 | S3 | 10 | 51,274 | 10 |
| *Rhynchosia difformis* | S3 | S1 |  |  |  |  |  |  |  |  |  |
| *Rhynchosia latifolia* |  | S1 | S1 | S1 | S1 | S1 | S1 | S1 | 2 | <1 | 2 |
| *Rhynchosia tomentosa* | S4 | S4 | S2 | S3 | S2 | S3 | S2 | S3 | 13 | 52,490 | 13 |
| *Robinia hispida* | S4 | S5 | S3 | S3 | S3 | S3 | S3 | S3 | 54 | 84,892 | 50 |
| *Robinia pseudoacacia* | S5 | S4 | S4 | S4 | S4 | S4 | S4 | S4 | 204 | 118,211 | 191 |
| *Senna hebecarpa* |  | S3 | S2 | S3 | S2 | S3 | S2 | S3 | 11 | 28,201 | 11 |
| *Senna marilandica* | S5 | S5 | S3 | S3 | S3 | S3 | S3 | S3 | 78 | 86,902 | 74 |
| *Sesbania herbacea* |  | S2 | S2 | S3 | S3 | S3 | S2 | S3 | 22 | 74,790 | 20 |
| *Strophostyles helvola* | S5 | S4 | S3 | S3 | S3 | S3 | S3 | S3 | 48 | 91,215 | 48 |
| *Strophostyles leiosperma* |  | S2 |  |  |  |  |  |  |  |  |  |
| *Strophostyles umbellata* | S5 | S5 | S3 | S3 | S3 | S3 | S3 | S3 | 60 | 75,207 | 56 |
| *Stylosanthes biflora* | S5 | S5 | S3 | S3 | S3 | S3 | S3 | S3 | 68 | 91,869 | 64 |
| *Tephrosia spicata* |  | S4 | S1 | S1 | S1 | S1 | S1 | S1 | 2 | 32 | 2 |
| *Tephrosia virginiana* | S5 | S5 | S3 | S4 | S3 | S4 | S3 | S4 | 119 | 101,955 | 114 |
| *Thermopsis fraxinifolia* | S2 | S1 |  |  | S2 | S3 | S3 | S3 | 21 | 2,830 | 26 |
| *Thermopsis mollis* | S1 | S2 | S2 | S2 | S2 | S2 | S2 | S2 | 9 | 1,180 | 8 |
| *Thermopsis villosa* | S1 | S2 | S1 | S1 | S1 | S1 | S1 | S1 | 4 | 3,711 | 4 |
| *Trifolium reflexum* | S1 | S3 |  |  | S1 | S2 | S1 | S2 | 5 | 376 | 5 |
| *Vicia caroliniana* | S5 | S5 | S4 | S4 | S4 | S4 | S4 | S4 | 133 | 77,805 | 116 |
| *Vicia minutiflora* |  | S4 | S2 | S2 | S2 | S2 | S2 | S2 | 10 | 5,648 | 10 |
| *Wisteria frutescens* |  | S5 | S3 | S3 | S3 | S3 | S3 | S3 | 23 | 100,633 | 23 |
| **FAGACEAE** |  |  |  |  |  |  |  |  |  |  |  |
| *Castanea dentata* |  | S5 | S4 | S4 | S4 | S4 | S4 | S4 | 180 | 107,357 | 168 |
| *Castanea pumila* |  | S4 | S3 | S3 | S3 | S3 | S3 | S3 | 39 | 34,400 | 37 |
| *Fagus grandifolia* | S5 | S5 | S4 | S5 | S4 | S5 | S4 | S5 | 517 | 127,589 | 454 |
| *Quercus alba* | S5 | S5 | S4 | S4 | S4 | S4 | S4 | S4 | 287 | 116,643 | 261 |
| *Quercus bicolor* |  | S4 | S2 | S3 | S2 | S3 | S2 | S3 | 22 | 53,928 | 18 |
| *Quercus coccinea* | S5 | S5 | S3 | S3 | S3 | S3 | S3 | S3 | 64 | 70,056 | 64 |
| *Quercus falcata* | S5 | S5 | S4 | S4 | S4 | S4 | S4 | S4 | 187 | 121,506 | 180 |
| *Quercus imbricaria* | S4 | S5 | S3 | S3 | S3 | S3 | S3 | S3 | 29 | 47,765 | 29 |
| *Quercus lyrata* | S5 | S5 | S3 | S3 | S3 | S3 | S3 | S3 | 28 | 82,221 | 28 |
| *Quercus macrocarpa* | S2 | S4 | S3 | S3 | S3 | S3 | S3 | S3 | 42 | 57,422 | 41 |
| *Quercus margaretiae* |  | S1 | S1 | S1 | S1 | S1 | S1 | S1 | 2 | 1 | 2 |
| *Quercus marilandica* | S5 | S5 | S3 | S4 | S3 | S4 | S3 | S4 | 112 | 98,751 | 108 |
| *Quercus michauxii* | S5 | S5 | S3 | S3 | S3 | S3 | S3 | S3 | 29 | 98,880 | 26 |
| *Quercus montana* | S5 | S5 | S4 | S4 | S4 | S4 | S4 | S4 | 158 | 96,923 | 146 |
| *Quercus muehlenbergii* | S5 | S5 | S3 | S4 | S3 | S4 | S3 | S4 | 103 | 107,750 | 95 |
| *Quercus nigra* |  | S5 | S3 | S4 | S3 | S4 | S3 | S4 | 114 | 111,588 | 104 |
| *Quercus pagoda* | S4 | S4 | S2 | S3 | S2 | S3 | S2 | S3 | 13 | 51,655 | 13 |
| *Quercus palustris* |  | S5 | S3 | S3 | S3 | S3 | S3 | S3 | 28 | 98,599 | 26 |
| *Quercus phellos* | S5 | S5 | S3 | S4 | S3 | S4 | S3 | S4 | 113 | 100,083 | 107 |
| *Quercus prinoides* | S1 | S3 | S2 | S2 | S2 | S2 | S2 | S2 | 3 | 40,765 | 3 |
| *Quercus rubra* | S5 | S5 | S4 | S4 | S3 | S4 | S4 | S4 | 131 | 107,867 | 120 |
| *Quercus shumardii* |  | S5 | S3 | S3 | S3 | S3 | S3 | S3 | 46 | 105,465 | 47 |
| *Quercus similis* |  | S1 |  |  |  |  |  |  |  |  |  |
| *Quercus stellata* | S5 | S5 | S4 | S4 | S4 | S4 | S4 | S4 | 240 | 111,311 | 228 |
| *Quercus texana* | S2 | S3 | S1 | S1 | S1 | S1 | S1 | S2 | 3 | 3,984 | 3 |
| *Quercus velutina* | S5 | S5 | S3 | S4 | S3 | S4 | S3 | S4 | 101 | 115,070 | 98 |
| **GELSEMIACEAE** |  |  |  |  |  |  |  |  |  |  |  |
| *Gelsemium sempervirens* | S5 | S3 | S2 | S1 | S2 | S1 | S2 | S1 | 13 | 796 | 11 |
| **GENTIANACEAE** |  |  |  |  |  |  |  |  |  |  |  |
| *Bartonia paniculata* | S3 | S4 | S1 | S1 | S1 | S1 | S1 | S1 | 3 | 165 | 3 |
| *Bartonia virginica* | S4 | S4 | S2 | S2 | S2 | S2 | S2 | S3 | 11 | 18,990 | 11 |
| *Frasera caroliniensis* |  | S4 | S3 | S4 | S3 | S4 | S3 | S4 | 94 | 68,004 | 81 |
| *Gentiana austromontana* |  | S2 | S1 | S1 | S1 | S1 | S1 | S1 | 5 | 2,910 | 5 |
| *Gentiana clausa* |  | S2 | S1 | S1 | S1 | S1 | S1 | S1 | 3 | 73 | 3 |
| *Gentiana decora* | S3 | S3 | S2 | S2 | S2 | S2 | S2 | S2 | 13 | 2,093 | 13 |
| *Gentiana linearis* |  | S1 | S2 | S1 | S2 | S1 | S2 | S1 | 8 | 173 | 7 |
| *Gentiana puberulenta* |  | S1 | S1 | S1 | S1 | S1 | S1 | S1 | 1 | <1 | 1 |
| *Gentiana saponaria* | S3 | S4 | S3 | S3 | S3 | S3 | S3 | S3 | 21 | 41,701 | 21 |
| *Gentiana villosa* | S4 | S5 | S3 | S3 | S3 | S3 | S3 | S3 | 28 | 73,065 | 29 |
| *Gentianella occidentalis* |  | S1 |  |  |  |  |  |  |  |  |  |
| *Gentianella quinquefolia* |  | S3 | S3 | S3 | S3 | S3 | S3 | S3 | 21 | 36,289 | 21 |
| *Gentianopsis crinita* | S1 |  |  |  |  |  |  |  |  |  |  |
| *Obolaria virginica* |  | S5 | S4 | S4 | S4 | S4 | S4 | S4 | 168 | 99,554 | 155 |
| *Sabatia angularis* | S5 | S5 | S4 | S4 | S4 | S4 | S4 | S4 | 260 | 106,520 | 253 |
| *Sabatia brachiata* | S3 | S4 | S2 | S3 | S2 | S2 | S2 | S3 | 13 | 22,844 | 13 |
| *Sabatia campanulata* | S1 | S3 | S3 | S3 | S3 | S3 | S3 | S3 | 31 | 8,590 | 31 |
| *Sabatia capitata* | S2 | S1 |  |  | S2 | S1 | S2 | S1 | 15 | 230 | 16 |
| **GERANIACEAE** |  |  |  |  |  |  |  |  |  |  |  |
| *Geranium carolinianum* | S5 | S5 | S4 | S4 | S4 | S4 | S4 | S4 | 169 | 123,390 | 160 |
| *Geranium maculatum* | S5 | S5 | S4 | S4 | S4 | S5 | S4 | S5 | 340 | 116,553 | 290 |
| *Geranium robertianum* |  | S2 | S2 | S2 | S2 | S2 | S2 | S2 | 6 | 3,576 | 6 |
| **GROSSULARIACEAE** |  |  |  |  |  |  |  |  |  |  |  |
| *Ribes aureum* |  | S1 | S1 | S1 | S1 | S1 | S1 | S1 | 2 | 6 | 2 |
| *Ribes curvatum* | S2 | S2 | S1 | S1 | S1 | S1 | S1 | S1 | 3 | 68 | 3 |
| *Ribes cynosbati* | S3 | S4 | S3 | S3 | S3 | S3 | S3 | S3 | 26 | 39,942 | 25 |
| *Ribes glandulosum* | S3 | S2 | S2 | S2 | S2 | S2 | S2 | S2 | 8 | 5,162 | 8 |
| *Ribes missouriense* |  | S2 |  |  | S2 | S2 | S2 | S3 | 6 | 19,980 | 7 |
| *Ribes rotundifolium* |  | S2 | S2 | S2 | S2 | S2 | S2 | S2 | 9 | 10,010 | 9 |
| **HALORAGACEAE** |  |  |  |  |  |  |  |  |  |  |  |
| *Myriophyllum heterophyllum* | S3 | S2 | S1 | S1 | S1 | S1 | S1 | S1 | 2 | 75 | 2 |
| *Myriophyllum pinnatum* |  | S1 |  |  | S1 | S2 | S2 | S2 | 4 | 7,112 | 4 |
| *Proserpinaca intermedia* |  | S1 |  |  |  |  |  |  |  |  |  |
| *Proserpinaca palustris* | S5 | S4 | S2 | S3 | S2 | S3 | S2 | S3 | 12 | 56,475 | 12 |
| *Proserpinaca pectinata* | S4 | S2 | S2 | S2 | S2 | S2 | S2 | S2 | 8 | 1,814 | 8 |
| **HAMAMELIDACEAE** |  |  |  |  |  |  |  |  |  |  |  |
| *Fothergilla major* | S1 | S2 |  |  | S2 | S2 | S3 | S3 | 12 | 19,567 | 12 |
| *Hamamelis virginiana* | S5 | S5 | S4 | S4 | S4 | S4 | S4 | S4 | 171 | 112,007 | 157 |
| **HYDRANGEACEAE** |  |  |  |  |  |  |  |  |  |  |  |
| *Hydrangea arborescens* | S5 | S5 | S4 | S4 | S4 | S4 | S4 | S4 | 231 | 130,638 | 201 |
| *Hydrangea barbara* | S4 | S2 | S3 | S3 | S3 | S3 | S3 | S3 | 29 | 66,335 | 27 |
| *Hydrangea cinerea* |  | S4 | S3 | S3 | S3 | S3 | S3 | S3 | 22 | 44,126 | 21 |
| *Hydrangea quercifolia* |  | S4 | S3 | S4 | S3 | S4 | S3 | S4 | 94 | 68,849 | 85 |
| *Hydrangea radiata* | S3 | S2 | S1 | S1 | S1 | S1 | S1 | S1 | 3 | 792 | 3 |
| *Philadelphus hirsutus* |  | S5 | S3 | S3 | S3 | S3 | S3 | S3 | 22 | 67,993 | 22 |
| *Philadelphus inodorus* | S3 | S4 | S2 | S3 | S2 | S3 | S2 | S3 | 10 | 59,273 | 9 |
| *Philadelphus pubescens* |  | S3 | S2 | S2 | S2 | S2 | S2 | S2 | 7 | 3,638 | 7 |
| **HYDROLEACEAE** |  |  |  |  |  |  |  |  |  |  |  |
| *Hydrolea ovata* | S4 | S1 |  |  | S1 | S1 | S1 | S1 | 1 | <1 | 1 |
| *Hydrolea quadrivalvis* | S3 | S2 |  |  | S1 | S1 | S2 | S2 | 4 | 4,088 | 4 |
| *Hydrolea uniflora* |  | S3 | S2 | S2 | S2 | S2 | S2 | S2 | 6 | 10,992 | 6 |
| **HYPERICACEAE** |  |  |  |  |  |  |  |  |  |  |  |
| *Hypericum adpressum* | S1 | S1 |  |  | S1 | S1 | S1 | S1 | 2 | 4 | 2 |
| *Hypericum canadense* | S3 | S3 | S2 | S1 | S2 | S1 | S2 | S2 | 6 | 4,512 | 5 |
| *Hypericum crux-andreae* | S5 | S4 | S2 | S2 | S3 | S3 | S2 | S2 | 21 | 18,237 | 20 |
| *Hypericum densiflorum* | S4 | S4 | S2 | S3 | S2 | S3 | S2 | S3 | 11 | 39,819 | 11 |
| *Hypericum denticulatum* | S1 | S4 | S2 | S2 | S2 | S2 | S2 | S2 | 10 | 12,353 | 10 |
| *Hypericum dolabriforme* |  | S3 | S1 | S1 | S1 | S1 | S1 | S1 | 5 | 671 | 5 |
| *Hypericum drummondii* |  | S5 | S2 | S3 | S2 | S3 | S2 | S3 | 11 | 41,767 | 11 |
| *Hypericum ellipticum* |  | S1 | S1 | S1 | S1 | S1 | S1 | S1 | 1 | <1 | 1 |
| *Hypericum frondosum* |  | S4 | S3 | S3 | S3 | S3 | S3 | S3 | 48 | 32,595 | 42 |
| *Hypericum gentianoides* | S5 | S5 | S3 | S3 | S3 | S3 | S3 | S3 | 59 | 77,084 | 55 |
| *Hypericum graveolens* |  | S2 | S1 | S1 | S1 | S1 | S1 | S1 | 2 | 33 | 2 |
| *Hypericum gymnanthum* |  | S2 | S2 | S2 | S2 | S2 | S2 | S2 | 9 | 6,043 | 9 |
| *Hypericum hypericoides* |  | S5 | S4 | S4 | S4 | S4 | S4 | S4 | 146 | 112,699 | 144 |
| *Hypericum interior* |  | S2 | S1 | S1 | S1 | S1 | S1 | S1 | 2 | 9 | 2 |
| *Hypericum lobocarpum* |  | S3 | S1 | S1 | S1 | S1 | S1 | S1 | 1 | <1 | 1 |
| *Hypericum mitchellianum* |  | S2 |  |  | S2 | S2 | S3 | S2 | 11 | 4,825 | 11 |
| *Hypericum mutilum* | S5 | S5 | S3 | S3 | S3 | S3 | S3 | S3 | 50 | 93,177 | 47 |
| *Hypericum nudiflorum* | S4 | S3 | S2 | S2 | S2 | S2 | S2 | S2 | 16 | 2,904 | 14 |
| *Hypericum prolificum* | S5 | S5 | S3 | S3 | S3 | S3 | S3 | S3 | 41 | 109,010 | 41 |
| *Hypericum punctatum* | S5 | S5 | S3 | S4 | S3 | S4 | S3 | S4 | 87 | 110,802 | 83 |
| *Hypericum sphaerocarpum* |  | S4 | S3 | S3 | S3 | S3 | S3 | S3 | 29 | 25,115 | 28 |
| *Hypericum stragulum* | S5 | S5 | S2 | S3 | S2 | S3 | S2 | S3 | 10 | 30,799 | 9 |
| *Hypericum virgatum* | S4 | S4 | S3 | S3 | S3 | S3 | S3 | S3 | 21 | 41,912 | 21 |
| *Triadenum fraseri* |  | S1 |  |  | S1 | S1 | S1 | S1 | 2 | 2 | 2 |
| *Triadenum tubulosum* | S2 | S3 | S1 | S1 | S1 | S1 | S1 | S1 | 2 | 67 | 2 |
| *Triadenum virginicum* |  | S3 | S1 | S1 | S1 | S1 | S1 | S1 | 1 | <1 | 1 |
| *Triadenum walteri* | S5 | S4 | S2 | S3 | S2 | S3 | S2 | S3 | 8 | 42,426 | 8 |
| **ITEACEAE** |  |  |  |  |  |  |  |  |  |  |  |
| *Itea virginica* | S5 | S5 | S3 | S4 | S3 | S4 | S3 | S4 | 82 | 101,766 | 82 |
| **JUGLANDACEAE** |  |  |  |  |  |  |  |  |  |  |  |
| *Carya aquatica* |  | S3 | S1 | S1 | S1 | S1 | S1 | S1 | 2 | 30 | 2 |
| *Carya carolinae-septentrionalis* | S3 | S4 | S3 | S3 | S3 | S3 | S3 | S3 | 21 | 33,204 | 21 |
| *Carya cordiformis* | S5 | S5 | S3 | S3 | S3 | S3 | S3 | S3 | 53 | 104,712 | 49 |
| *Carya glabra* | S5 | S5 | S3 | S3 | S3 | S3 | S3 | S3 | 57 | 82,798 | 55 |
| *Carya illinoinensis* |  | S4 | S3 | S3 | S3 | S3 | S3 | S3 | 44 | 104,997 | 43 |
| *Carya laciniosa* | S2 | S5 | S2 | S3 | S2 | S3 | S2 | S3 | 18 | 59,283 | 18 |
| *Carya ovalis* |  | S5 | S2 | S2 | S2 | S2 | S2 | S2 | 8 | 9,760 | 7 |
| *Carya ovata* | S5 | S5 | S3 | S4 | S3 | S4 | S3 | S4 | 113 | 107,721 | 104 |
| *Carya pallida* | S4 | S5 | S2 | S3 | S2 | S3 | S2 | S3 | 18 | 67,428 | 17 |
| *Carya texana* |  | S1 | S1 | S1 | S1 | S1 | S1 | S1 | 1 | <1 | 1 |
| *Carya tomentosa* | S5 | S5 | S2 | S3 | S3 | S3 | S2 | S3 | 20 | 54,869 | 20 |
| *Juglans cinerea* | S2 | S5 |  |  | S4 | S4 | S4 | S4 | 205 | 92,856 | 205 |
| *Juglans nigra* | S5 | S5 | S4 | S4 | S4 | S4 | S4 | S4 | 290 | 129,690 | 274 |
| **LAMIACEAE** |  |  |  |  |  |  |  |  |  |  |  |
| *Agastache nepetoides* | S1 | S4 | S2 | S3 | S2 | S3 | S2 | S3 | 10 | 40,949 | 9 |
| *Agastache scrophulariifolia* | S1 | S1 |  |  | S1 | S1 | S2 | S2 | 4 | 235 | 5 |
| *Blephilia ciliata* | S4 | S5 | S3 | S3 | S3 | S3 | S3 | S3 | 59 | 42,233 | 56 |
| *Blephilia hirsuta* |  | S4 | S2 | S3 | S2 | S3 | S2 | S3 | 17 | 46,734 | 17 |
| *Callicarpa americana* |  | S5 | S4 | S4 | S4 | S4 | S4 | S4 | 205 | 110,673 | 183 |
| *Clinopodium arkansanum* |  | S1 | S3 | S3 | S3 | S3 | S3 | S3 | 27 | 3,394 | 23 |
| *Clinopodium glabellum* | S1 | S2 | S2 | S2 | S2 | S2 | S2 | S2 | 18 | 2,382 | 17 |
| *Clinopodium vulgare* |  | S3 | S3 | S3 | S3 | S3 | S3 | S3 | 52 | 41,685 | 45 |
| *Collinsonia canadensis* | S5 | S5 | S3 | S4 | S3 | S4 | S3 | S4 | 121 | 111,060 | 114 |
| *Collinsonia tuberosa* | S3 | S2 |  |  |  |  |  |  |  |  |  |
| *Collinsonia verticillata* | S1 | S3 | S3 | S3 | S3 | S3 | S3 | S3 | 26 | 28,661 | 25 |
| *Conradina verticillata* |  | S2 | S3 | S3 | S3 | S3 | S3 | S3 | 41 | 5,627 | 40 |
| *Cunila origanoides* | S5 | S4 | S3 | S3 | S3 | S3 | S3 | S3 | 46 | 77,752 | 46 |
| *Hedeoma hispida* |  | S3 | S1 | S1 | S1 | S1 | S2 | S1 | 5 | 1,919 | 5 |
| *Hedeoma pulegioides* |  | S5 | S2 | S3 | S2 | S3 | S2 | S3 | 12 | 43,499 | 11 |
| *Lycopus americanus* | S5 | S5 | S2 | S3 | S2 | S3 | S2 | S3 | 9 | 46,467 | 9 |
| *Lycopus rubellus* | S4 | S4 | S2 | S3 | S2 | S3 | S2 | S3 | 8 | 46,991 | 8 |
| *Lycopus uniflorus* |  | S2 | S2 | S3 | S1 | S1 | S2 | S3 | 6 | 34,308 | 6 |
| *Lycopus virginicus* | S5 | S5 | S3 | S3 | S3 | S3 | S3 | S3 | 25 | 65,862 | 24 |
| *Meehania cordata* |  | S2 |  |  | S2 | S2 | S2 | S2 | 10 | 5,667 | 10 |
| *Mentha canadensis* |  | S1 | S1 | S2 | S1 | S2 | S1 | S2 | 4 | 14,008 | 4 |
| *Monarda austroappalachiana* | S1 | S1 | S2 | S2 | S2 | S2 | S2 | S2 | 8 | 9,564 | 7 |
| *Monarda bradburiana* |  | S3 | S3 | S3 | S3 | S3 | S3 | S3 | 36 | 12,099 | 29 |
| *Monarda citriodora* |  | S3 | S3 | S3 | S3 | S3 | S3 | S3 | 37 | 72,990 | 34 |
| *Monarda clinopodia* | S5 | S4 | S3 | S3 | S3 | S3 | S3 | S3 | 63 | 44,200 | 59 |
| *Monarda didyma* | S4 | S3 | S4 | S4 | S3 | S4 | S4 | S4 | 127 | 78,967 | 106 |
| *Monarda fistulosa* | S4 | S5 | S4 | S4 | S4 | S4 | S4 | S4 | 208 | 121,158 | 200 |
| *Monarda media* |  | S1 | S1 | S1 | S1 | S1 | S1 | S1 | 1 | <1 | 2 |
| *Monarda punctata* |  | S2 | S2 | S3 | S2 | S3 | S2 | S3 | 17 | 82,143 | 17 |
| *Physostegia virginiana* |  | S5 | S3 | S3 | S3 | S3 | S3 | S3 | 56 | 80,190 | 51 |
| *Prunella vulgaris* | S5 | S5 | S4 | S5 | S4 | S5 | S4 | S5 | 578 | 133,804 | 515 |
| *Pycnanthemum albescens* |  | S1 |  |  |  |  |  |  |  |  |  |
| *Pycnanthemum beadlei* |  | S1 |  |  | S1 | S1 | S1 | S1 | 1 | <1 | 1 |
| *Pycnanthemum curvipes* | S1 | S2 | S2 | S2 | S2 | S2 | S2 | S2 | 6 | 5,571 | 6 |
| *Pycnanthemum incanum* |  | S5 | S2 | S2 | S2 | S2 | S2 | S3 | 11 | 19,385 | 11 |
| *Pycnanthemum loomisii* |  | S5 | S3 | S3 | S3 | S3 | S3 | S3 | 60 | 82,980 | 57 |
| *Pycnanthemum montanum* | S3 | S3 | S2 | S2 | S2 | S2 | S2 | S2 | 15 | 2,761 | 14 |
| *Pycnanthemum muticum* |  | S4 | S3 | S3 | S3 | S3 | S3 | S3 | 40 | 70,012 | 38 |
| *Pycnanthemum pilosum* |  | S3 | S1 | S1 | S1 | S1 | S1 | S1 | 1 | <1 | 1 |
| *Pycnanthemum pycnanthemoides* | S4 | S4 | S2 | S3 | S2 | S3 | S2 | S3 | 12 | 54,345 | 11 |
| *Pycnanthemum tenuifolium* | S5 | S5 | S4 | S4 | S4 | S4 | S4 | S4 | 146 | 103,216 | 139 |
| *Pycnanthemum torreyi* |  | S1 | S1 | S1 | S1 | S1 | S1 | S1 | 2 | <1 | 1 |
| *Pycnanthemum verticillatum* | S3 | S2 | S2 | S3 | S3 | S3 | S2 | S2 | 21 | 21,756 | 20 |
| *Pycnanthemum virginianum* | S1 | S3 | S1 | S1 | S1 | S1 | S1 | S1 | 3 | 700 | 3 |
| *Salvia azurea* |  | S1 | S2 | S2 | S2 | S3 | S2 | S3 | 7 | 19,340 | 6 |
| *Salvia lyrata* | S5 | S5 | S4 | S5 | S4 | S5 | S4 | S5 | 818 | 125,938 | 720 |
| *Salvia urticifolia* | S4 | S4 | S3 | S3 | S3 | S3 | S3 | S3 | 31 | 52,631 | 30 |
| *Scutellaria arguta* |  | S1 |  |  |  |  |  |  |  |  |  |
| *Scutellaria australis* |  | S4 | S1 | S1 | S1 | S1 | S1 | S1 | 2 | <1 | 2 |
| *Scutellaria elliptica* | S5 | S5 | S4 | S4 | S4 | S4 | S4 | S4 | 140 | 99,586 | 131 |
| *Scutellaria incana* |  | S5 | S3 | S3 | S3 | S3 | S3 | S3 | 43 | 68,901 | 42 |
| *Scutellaria integrifolia* | S5 | S5 | S3 | S3 | S3 | S3 | S3 | S3 | 58 | 91,408 | 57 |
| *Scutellaria lateriflora* |  | S5 | S2 | S3 | S2 | S3 | S2 | S3 | 19 | 96,670 | 19 |
| *Scutellaria leonardii* | S1 | S3 | S1 | S1 | S1 | S1 | S1 | S1 | 1 | <1 | 1 |
| *Scutellaria montana* |  | S2 |  |  | S3 | S3 | S3 | S3 | 86 | 1,353 | 109 |
| *Scutellaria nervosa* | S1 | S4 | S2 | S3 | S2 | S3 | S2 | S3 | 8 | 39,235 | 7 |
| *Scutellaria ovata* |  | S4 | S3 | S3 | S3 | S3 | S3 | S3 | 57 | 74,836 | 54 |
| *Scutellaria parvula* |  | S4 | S3 | S3 | S3 | S3 | S3 | S3 | 77 | 52,416 | 70 |
| *Scutellaria pseudoserrata* |  | S3 | S1 | S1 | S2 | S2 | S2 | S2 | 5 | 1,347 | 5 |
| *Scutellaria saxatilis* |  | S3 |  |  |  |  |  |  |  |  |  |
| *Scutellaria serrata* |  | S3 | S2 | S2 | S2 | S2 | S2 | S2 | 8 | 11,662 | 8 |
| *Stachys clingmanii* |  | S2 |  |  | S2 | S2 | S2 | S2 | 12 | 1,901 | 12 |
| *Stachys cordata* |  | S4 | S3 | S3 | S3 | S3 | S3 | S3 | 25 | 43,272 | 24 |
| *Stachys hispida* |  | S2 | S1 | S1 | S1 | S1 | S1 | S1 | 1 | <1 | 1 |
| *Stachys latidens* |  | S2 | S2 | S2 | S1 | S1 | S2 | S2 | 6 | 5,113 | 5 |
| *Stachys nuttallii* |  | S4 | S2 | S2 | S2 | S2 | S2 | S2 | 7 | 6,809 | 7 |
| *Stachys salvioides* |  | S3 | S2 | S2 | S2 | S2 | S2 | S2 | 11 | 1,687 | 11 |
| *Stachys subcordata* |  | S1 |  |  |  |  |  |  |  |  |  |
| *Stachys tenuifolia* |  | S4 | S2 | S3 | S2 | S3 | S2 | S3 | 8 | 40,322 | 8 |
| *Synandra hispidula* | S1 | S3 | S3 | S3 | S3 | S3 | S3 | S3 | 43 | 47,110 | 39 |
| *Teucrium canadense* |  | S5 | S4 | S4 | S4 | S4 | S4 | S4 | 159 | 122,031 | 147 |
| *Trichostema brachiatum* |  | S4 | S3 | S3 | S3 | S3 | S3 | S3 | 49 | 46,406 | 46 |
| *Trichostema dichotomum* |  | S5 | S3 | S3 | S3 | S3 | S3 | S3 | 45 | 87,628 | 45 |
| *Trichostema setaceum* |  | S2 | S2 | S3 | S2 | S3 | S2 | S3 | 7 | 37,580 | 7 |
| **LENTIBULARIACEAE** |  |  |  |  |  |  |  |  |  |  |  |
| *Utricularia cornuta* | S1 | S1 | S1 | S1 | S1 | S1 | S1 | S1 | 1 | <1 | 1 |
| *Utricularia gibba* |  | S3 | S2 | S3 | S2 | S3 | S2 | S3 | 14 | 45,257 | 14 |
| *Utricularia inflata* | S3 | S1 | S1 | S1 | S1 | S1 | S1 | S1 | 2 | 45 | 2 |
| *Utricularia macrorhiza* | S1 | S1 | S1 | S1 | S1 | S1 | S1 | S1 | 1 | <1 | 1 |
| *Utricularia resupinata* | S1 | S1 |  |  |  |  |  |  |  |  |  |
| *Utricularia subulata* |  | S2 |  |  | S2 | S2 | S2 | S2 | 6 | 3,554 | 6 |
| **LINACEAE** |  |  |  |  |  |  |  |  |  |  |  |
| *Linum curtissii* | S5 | S4 | S1 | S1 | S1 | S1 | S1 | S1 | 1 | <1 | 1 |
| *Linum intercursum* |  | S3 | S1 | S1 | S1 | S1 | S1 | S1 | 1 | <1 | 1 |
| *Linum striatum* | S5 | S5 | S2 | S3 | S2 | S3 | S2 | S3 | 17 | 39,303 | 16 |
| *Linum sulcatum* | S3 | S3 | S2 | S3 | S2 | S2 | S2 | S3 | 7 | 23,586 | 6 |
| *Linum virginianum* | S4 | S4 | S2 | S3 | S2 | S3 | S2 | S3 | 8 | 36,993 | 8 |
| **LINDERNIACEAE** |  |  |  |  |  |  |  |  |  |  |  |
| *Lindernia anagallidea* |  | S3 | S1 | S1 | S1 | S1 | S1 | S1 | 1 | <1 | 1 |
| *Lindernia dubia* | S5 | S5 | S3 | S3 | S3 | S3 | S3 | S3 | 33 | 112,415 | 33 |
| **LOGANIACEAE** |  |  |  |  |  |  |  |  |  |  |  |
| *Mitreola petiolata* | S1 | S3 | S2 | S2 | S2 | S2 | S2 | S2 | 8 | 6,040 | 8 |
| *Spigelia marilandica* |  | S5 | S4 | S5 | S4 | S5 | S4 | S4 | 337 | 112,390 | 307 |
| **LYTHRACEAE** |  |  |  |  |  |  |  |  |  |  |  |
| *Ammannia coccinea* | S4 | S5 | S3 | S3 | S3 | S3 | S3 | S3 | 21 | 51,244 | 22 |
| *Ammannia robusta* |  | S2 |  |  |  |  |  |  |  |  |  |
| *Cuphea viscosissima* |  | S5 | S3 | S3 | S3 | S3 | S3 | S3 | 29 | 57,877 | 30 |
| *Decodon verticillatus* |  | S3 | S2 | S3 | S2 | S3 | S2 | S3 | 10 | 28,967 | 10 |
| *Didiplis diandra* | S1 | S1 |  |  | S2 | S3 | S2 | S3 | 6 | 39,675 | 6 |
| *Lythrum alatum* |  | S3 | S2 | S3 | S2 | S3 | S2 | S3 | 19 | 30,776 | 18 |
| *Lythrum lanceolatum* | S1 | S1 |  |  |  |  |  |  |  |  |  |
| *Rotala ramosior* | S5 | S5 | S2 | S3 | S2 | S3 | S2 | S3 | 12 | 48,813 | 12 |
| **MALVACEAE** |  |  |  |  |  |  |  |  |  |  |  |
| *Callirhoe alcaeoides* |  | S1 |  |  |  |  |  |  |  |  |  |
| *Hibiscus laevis* |  | S4 | S3 | S3 | S3 | S3 | S3 | S3 | 62 | 106,113 | 58 |
| *Hibiscus lasiocarpos* |  | S2 |  |  | S3 | S3 | S3 | S3 | 22 | 75,052 | 21 |
| *Hibiscus moscheutos* | S5 | S5 | S3 | S3 | S3 | S3 | S3 | S3 | 75 | 114,568 | 71 |
| *Malvastrum hispidum* | S2 | S3 | S2 | S2 | S2 | S2 | S2 | S2 | 7 | 2,934 | 7 |
| *Sida elliottii* |  | S2 | S1 | S1 | S1 | S1 | S1 | S1 | 3 | 412 | 3 |
| *Tilia americana* | S5 | S5 | S3 | S4 | S3 | S4 | S3 | S4 | 96 | 84,870 | 83 |
| **MELASTOMATACEAE** |  |  |  |  |  |  |  |  |  |  |  |
| *Rhexia interior* |  | S3 | S1 | S2 | S1 | S2 | S2 | S2 | 3 | 16,175 | 3 |
| *Rhexia mariana* | S5 | S5 | S3 | S3 | S3 | S3 | S3 | S3 | 59 | 88,256 | 57 |
| *Rhexia virginica* | S5 | S5 | S3 | S3 | S3 | S3 | S3 | S3 | 54 | 85,735 | 50 |
| **MENISPERMACEAE** |  |  |  |  |  |  |  |  |  |  |  |
| *Calycocarpum lyonii* |  | S4 | S2 | S2 | S2 | S2 | S2 | S3 | 6 | 16,062 | 6 |
| *Menispermum canadense* |  | S5 | S3 | S3 | S3 | S3 | S3 | S3 | 62 | 103,791 | 56 |
| *Nephroia carolina* | S5 | S3 | S4 | S4 | S4 | S4 | S4 | S4 | 209 | 115,364 | 184 |
| **MOLLUGINACEAE** |  |  |  |  |  |  |  |  |  |  |  |
| *Mollugo verticillata* |  | S5 | S3 | S3 | S3 | S3 | S3 | S3 | 39 | 88,377 | 38 |
| **MONTIACEAE** |  |  |  |  |  |  |  |  |  |  |  |
| *Claytonia caroliniana* | S4 | S5 | S3 | S4 | S3 | S4 | S3 | S4 | 100 | 75,649 | 89 |
| *Claytonia virginica* | S5 | S5 | S4 | S5 | S4 | S5 | S4 | S5 | 478 | 129,205 | 430 |
| *Phemeranthus calcaricus* |  | S2 |  |  | S4 | S4 | S4 | S4 | 135 | 13,303 | 155 |
| *Phemeranthus mengesii* | S3 | S1 |  |  | S2 | S2 | S2 | S2 | 7 | 2,143 | 7 |
| *Phemeranthus teretifolius* | S1 | S1 |  |  | S2 | S2 | S2 | S2 | 11 | 6,385 | 11 |
| **MORACEAE** |  |  |  |  |  |  |  |  |  |  |  |
| *Morus rubra* | S5 | S5 | S4 | S4 | S4 | S4 | S4 | S4 | 149 | 125,117 | 142 |
| **MYRICACEAE** |  |  |  |  |  |  |  |  |  |  |  |
| *Comptonia peregrina* | S1 | S1 | S1 | S1 | S1 | S1 | S1 | S1 | 1 | <1 | 1 |
| *Morella cerifera* |  | S1 |  |  |  |  |  |  |  |  |  |
| **MYRSINACEAE** |  |  |  |  |  |  |  |  |  |  |  |
| *Centunculus minimus* |  | S1 | S1 | S1 | S1 | S1 | S1 | S1 | 1 | <1 | 1 |
| *Lysimachia × producta* |  | S1 |  |  | S1 | S1 | S1 | S1 | 1 | <1 | 1 |
| *Lysimachia fraseri* |  | S2 |  |  | S2 | S3 | S3 | S3 | 12 | 27,175 | 11 |
| *Lysimachia quadrifolia* | S5 | S5 | S4 | S4 | S4 | S4 | S4 | S4 | 153 | 77,758 | 140 |
| *Lysimachia terrestris* | S1 | S2 | S1 | S2 | S1 | S2 | S1 | S2 | 4 | 14,832 | 4 |
| *Steironema ciliatum* | S5 | S5 | S3 | S3 | S3 | S3 | S3 | S3 | 59 | 106,594 | 57 |
| *Steironema hybridum* |  | S3 |  |  | S1 | S1 | S1 | S1 | 1 | <1 | 1 |
| *Steironema lanceolatum* | S5 | S5 | S3 | S3 | S3 | S3 | S3 | S3 | 37 | 81,709 | 36 |
| *Steironema lewisii* |  | S1 | S2 | S1 | S2 | S1 | S2 | S1 | 10 | 172 | 10 |
| *Steironema quadriflorum* | S3 | S2 | S1 | S1 | S1 | S1 | S1 | S1 | 1 | <1 | 1 |
| *Steironema radicans* |  | S3 | S1 | S1 | S1 | S1 | S1 | S1 | 3 | 3,492 | 3 |
| *Steironema tonsum* | S4 | S4 | S2 | S3 | S3 | S3 | S2 | S3 | 22 | 28,366 | 20 |
| *Trientalis borealis* | S1 | S1 |  |  | S1 | S1 | S2 | S2 | 5 | 4,223 | 5 |
| **NELUMBONACEAE** |  |  |  |  |  |  |  |  |  |  |  |
| *Nelumbo lutea* |  | S3 | S3 | S3 | S3 | S3 | S3 | S3 | 38 | 100,430 | 33 |
| **NYCTAGINACEAE** |  |  |  |  |  |  |  |  |  |  |  |
| *Mirabilis albida* | S2 | S1 |  |  | S2 | S2 | S2 | S2 | 10 | 1,670 | 11 |
| **NYSSACEAE** |  |  |  |  |  |  |  |  |  |  |  |
| *Nyssa aquatica* |  | S4 | S2 | S2 | S2 | S2 | S2 | S2 | 11 | 10,629 | 11 |
| *Nyssa biflora* | S5 | S3 | S2 | S3 | S2 | S3 | S2 | S3 | 8 | 30,837 | 8 |
| *Nyssa sylvatica* | S5 | S5 | S4 | S4 | S4 | S4 | S4 | S4 | 160 | 111,371 | 158 |
| **OLEACEAE** |  |  |  |  |  |  |  |  |  |  |  |
| *Chionanthus virginicus* | S5 | S5 | S3 | S4 | S3 | S4 | S3 | S4 | 95 | 97,999 | 87 |
| *Forestiera acuminata* |  | S4 | S2 | S2 | S2 | S2 | S2 | S2 | 5 | 26,877 | 5 |
| *Forestiera ligustrina* | S1 | S4 | S3 | S3 | S3 | S3 | S3 | S3 | 28 | 11,095 | 28 |
| *Fraxinus americana* | S5 | S5 | S3 | S3 | S3 | S3 | S3 | S3 | 83 | 98,639 | 78 |
| *Fraxinus biltmoreana* |  | S5 | S2 | S3 | S2 | S3 | S2 | S3 | 10 | 33,144 | 9 |
| *Fraxinus pennsylvanica* | S5 | S5 | S3 | S3 | S3 | S3 | S3 | S3 | 57 | 98,293 | 54 |
| *Fraxinus profunda* | S3 | S3 | S1 | S2 | S1 | S2 | S1 | S2 | 5 | 15,020 | 5 |
| *Fraxinus quadrangulata* |  | S5 | S3 | S3 | S3 | S3 | S3 | S3 | 60 | 59,962 | 58 |
| *Fraxinus smallii* |  | S2 | S1 | S1 | S1 | S1 | S1 | S1 | 1 | <1 | 1 |
| **ONAGRACEAE** |  |  |  |  |  |  |  |  |  |  |  |
| *Chamaenerion angustifolium* |  | S1 | S1 | S1 | S1 | S1 | S1 | S1 | 4 | 840 | 3 |
| *Circaea alpina* |  | S3 | S1 | S1 | S1 | S1 | S2 | S1 | 5 | 3,025 | 5 |
| *Circaea canadensis* | S5 | S4 | S3 | S3 | S3 | S3 | S3 | S3 | 27 | 69,252 | 28 |
| *Epilobium ciliatum* | S2 | S2 |  |  | S1 | S1 | S2 | S2 | 4 | 3,250 | 4 |
| *Epilobium coloratum* |  | S5 | S3 | S3 | S3 | S3 | S3 | S3 | 22 | 64,308 | 22 |
| *Epilobium leptophyllum* | S2 | S1 |  |  | S1 | S1 | S1 | S1 | 2 | <1 | 2 |
| *Ludwigia alternifolia* | S5 | S5 | S3 | S4 | S3 | S4 | S3 | S4 | 85 | 120,362 | 82 |
| *Ludwigia decurrens* | S5 | S5 | S3 | S3 | S3 | S3 | S3 | S3 | 24 | 75,586 | 23 |
| *Ludwigia glandulosa* | S4 | S4 | S1 | S2 | S1 | S1 | S1 | S2 | 3 | 5,207 | 3 |
| *Ludwigia hirtella* |  | S3 | S1 | S1 | S2 | S1 | S2 | S1 | 5 | 1,825 | 5 |
| *Ludwigia leptocarpa* | S3 | S3 | S2 | S3 | S2 | S3 | S2 | S3 | 7 | 38,334 | 7 |
| *Ludwigia linearis* | S5 | S3 | S1 | S1 | S1 | S1 | S1 | S2 | 4 | 4,076 | 4 |
| *Ludwigia microcarpa* |  | S3 | S2 | S3 | S2 | S2 | S2 | S3 | 13 | 20,114 | 11 |
| *Ludwigia palustris* | S5 | S5 | S3 | S3 | S3 | S3 | S3 | S3 | 33 | 112,022 | 33 |
| *Ludwigia peploides* |  | S3 | S3 | S3 | S3 | S3 | S3 | S3 | 25 | 95,867 | 26 |
| *Ludwigia repens* |  |  | S1 | S1 | S1 | S1 | S1 | S1 | 1 | <1 | 1 |
| *Ludwigia sphaerocarpa* | S3 | S1 | S1 | S1 | S1 | S1 | S1 | S1 | 3 | 239 | 3 |
| *Oenothera biennis* | S5 | S5 | S3 | S4 | S3 | S4 | S3 | S4 | 91 | 122,233 | 87 |
| *Oenothera curtiflora* | S3 | S1 | S1 | S1 | S1 | S1 | S1 | S1 | 1 | <1 | 1 |
| *Oenothera filiformis* |  | S1 | S1 | S1 | S1 | S1 | S1 | S1 | 2 | 44 | 2 |
| *Oenothera filipes* |  | S3 | S2 | S2 | S2 | S2 | S2 | S2 | 8 | 15,447 | 8 |
| *Oenothera fruticosa* | S5 | S4 | S2 | S3 | S2 | S3 | S2 | S3 | 20 | 44,822 | 19 |
| *Oenothera gaura* | S5 | S3 | S3 | S3 | S3 | S3 | S3 | S3 | 36 | 94,059 | 33 |
| *Oenothera glauca* | S5 | S1 | S1 | S1 | S1 | S1 | S1 | S1 | 1 | <1 | 1 |
| *Oenothera laciniata* | S5 | S5 | S3 | S3 | S3 | S3 | S3 | S3 | 30 | 95,666 | 28 |
| *Oenothera linifolia* | S1 | S3 | S1 | S2 | S1 | S2 | S1 | S2 | 4 | 6,097 | 4 |
| *Oenothera macrocarpa* |  | S2 | S2 | S1 | S2 | S1 | S2 | S1 | 15 | 622 | 14 |
| *Oenothera nutans* |  | S2 | S1 | S1 | S1 | S1 | S1 | S1 | 1 | <1 | 1 |
| *Oenothera parviflora* | S3 | S2 |  |  | S2 | S2 | S2 | S2 | 7 | 12,577 | 7 |
| *Oenothera perennis* | S2 | S1 |  |  |  |  |  |  |  |  |  |
| *Oenothera pilosella* |  | S1 | S1 | S1 | S1 | S1 | S1 | S1 | 1 | <1 | 1 |
| *Oenothera tetragona* |  | S3 | S2 | S3 | S2 | S2 | S2 | S3 | 8 | 22,150 | 8 |
| *Oenothera triloba* | S1 | S3 | S3 | S3 | S3 | S3 | S3 | S3 | 24 | 18,829 | 21 |
| *Oenothera villosa* |  | S2 |  |  |  |  |  |  |  |  |  |
| **OROBANCHACEAE** |  |  |  |  |  |  |  |  |  |  |  |
| *Agalinis auriculata* | S1 | S2 |  |  | S2 | S3 | S2 | S3 | 9 | 30,626 | 10 |
| *Agalinis decemloba* |  | S1 | S1 | S1 | S1 | S1 | S1 | S1 | 4 | 700 | 4 |
| *Agalinis fasciculata* |  | S4 | S3 | S3 | S3 | S3 | S3 | S3 | 22 | 40,258 | 21 |
| *Agalinis gattingeri* |  | S4 | S3 | S3 | S3 | S3 | S3 | S3 | 34 | 52,738 | 33 |
| *Agalinis heterophylla* |  | S1 |  |  | S1 | S1 | S1 | S1 | 1 | <1 | 1 |
| *Agalinis obtusifolia* | S4 | S3 | S1 | S1 | S1 | S1 | S1 | S1 | 2 | 12 | 2 |
| *Agalinis oligophylla* |  | S1 | S1 | S1 | S1 | S1 | S1 | S1 | 2 | 2 | 2 |
| *Agalinis plukenetii* |  | S1 | S1 | S1 | S1 | S1 | S1 | S1 | 2 | 2 | 2 |
| *Agalinis purpurea* | S5 | S5 | S3 | S3 | S3 | S3 | S3 | S3 | 51 | 113,089 | 48 |
| *Agalinis skinneriana* |  | S1 | S1 | S1 | S1 | S1 | S1 | S1 | 2 | 2 | 2 |
| *Agalinis tenuifolia* |  | S5 | S3 | S4 | S3 | S4 | S3 | S4 | 91 | 101,002 | 86 |
| *Aureolaria flava* | S5 | S5 | S2 | S3 | S2 | S3 | S2 | S3 | 15 | 73,940 | 14 |
| *Aureolaria levigata* | S5 | S4 | S2 | S3 | S2 | S2 | S2 | S3 | 10 | 21,891 | 9 |
| *Aureolaria patula* | S1 | S3 |  |  | S3 | S3 | S3 | S3 | 53 | 36,431 | 54 |
| *Aureolaria pectinata* | S3 | S5 | S3 | S3 | S3 | S3 | S3 | S3 | 23 | 70,916 | 23 |
| *Aureolaria pedicularia* | S4 | S3 | S1 | S1 | S1 | S1 | S1 | S1 | 1 | <1 | 1 |
| *Aureolaria virginica* | S5 | S5 | S3 | S3 | S3 | S3 | S3 | S3 | 24 | 46,435 | 23 |
| *Buchnera americana* | S4 | S3 | S2 | S2 | S2 | S2 | S2 | S2 | 9 | 13,349 | 9 |
| *Castilleja coccinea* | S1 | S3 | S2 | S3 | S2 | S2 | S2 | S3 | 17 | 29,121 | 16 |
| *Conopholis americana* | S5 | S5 | S4 | S5 | S4 | S5 | S4 | S5 | 481 | 90,841 | 395 |
| *Dasistoma macrophyllum* | S1 | S4 | S3 | S3 | S3 | S3 | S3 | S3 | 50 | 78,693 | 48 |
| *Epifagus virginiana* | S5 | S5 | S4 | S4 | S4 | S4 | S4 | S4 | 142 | 116,212 | 138 |
| *Melampyrum lineare* |  | S3 | S3 | S3 | S3 | S3 | S3 | S3 | 57 | 40,860 | 53 |
| *Orobanche uniflorum* |  | S4 | S3 | S3 | S3 | S3 | S3 | S3 | 56 | 53,919 | 44 |
| *Pedicularis canadensis* | S5 | S5 | S4 | S4 | S4 | S4 | S4 | S4 | 205 | 111,996 | 183 |
| *Pedicularis lanceolata* | S1 | S2 | S2 | S3 | S2 | S3 | S2 | S3 | 8 | 30,987 | 8 |
| **OXALIDACEAE** |  |  |  |  |  |  |  |  |  |  |  |
| *Oxalis colorea* |  | S1 |  |  |  |  |  |  |  |  |  |
| *Oxalis dillenii* |  | S5 | S3 | S3 | S3 | S3 | S3 | S3 | 50 | 93,501 | 50 |
| *Oxalis florida* |  | S3 | S1 | S1 | S1 | S1 | S1 | S1 | 1 | <1 | 1 |
| *Oxalis grandis* |  | S5 | S3 | S3 | S3 | S3 | S3 | S3 | 33 | 51,884 | 30 |
| *Oxalis illinoensis* |  | S4 | S3 | S3 | S3 | S3 | S3 | S3 | 21 | 24,215 | 21 |
| *Oxalis macrantha* |  | S3 | S2 | S2 | S2 | S2 | S2 | S2 | 8 | 2,280 | 8 |
| *Oxalis montana* | S4 | S2 | S3 | S3 | S3 | S3 | S3 | S3 | 67 | 36,188 | 51 |
| *Oxalis stricta* | S5 | S5 | S3 | S3 | S3 | S3 | S3 | S3 | 60 | 110,848 | 58 |
| *Oxalis violacea* |  | S5 | S4 | S5 | S4 | S5 | S4 | S5 | 360 | 116,093 | 320 |
| **PAPAVERACEAE** |  |  |  |  |  |  |  |  |  |  |  |
| *Adlumia fungosa* |  | S3 | S2 | S2 | S2 | S2 | S2 | S2 | 8 | 1,534 | 7 |
| *Capnoides sempervirens* | S3 | S1 | S2 | S1 | S2 | S1 | S2 | S1 | 9 | 779 | 9 |
| *Corydalis flavula* | S5 | S5 | S3 | S4 | S3 | S4 | S3 | S4 | 94 | 108,979 | 84 |
| *Corydalis micrantha* |  | S1 |  |  |  |  |  |  |  |  |  |
| *Dicentra canadensis* | S5 | S4 | S3 | S3 | S3 | S4 | S3 | S4 | 92 | 53,219 | 80 |
| *Dicentra cucullaria* | S5 | S5 | S4 | S4 | S4 | S4 | S4 | S4 | 214 | 111,103 | 195 |
| *Dicentra eximia* |  | S4 | S3 | S3 | S3 | S3 | S3 | S3 | 39 | 46,107 | 33 |
| *Sanguinaria canadensis* | S5 | S5 | S4 | S5 | S4 | S5 | S4 | S5 | 430 | 129,608 | 383 |
| *Stylophorum diphyllum* | S1 | S5 | S4 | S4 | S4 | S4 | S4 | S4 | 146 | 95,368 | 130 |
| **PARNASSIACEAE** |  |  |  |  |  |  |  |  |  |  |  |
| *Parnassia asarifolia* | S1 | S3 | S3 | S3 | S3 | S3 | S3 | S3 | 36 | 35,755 | 32 |
| *Parnassia grandifolia* |  | S3 | S3 | S3 | S3 | S3 | S3 | S3 | 33 | 17,288 | 32 |
| **PASSIFLORACEAE** |  |  |  |  |  |  |  |  |  |  |  |
| *Passiflora incarnata* | S5 | S5 | S4 | S5 | S4 | S5 | S4 | S5 | 700 | 131,263 | 643 |
| *Passiflora lutea* | S5 | S5 | S4 | S4 | S4 | S4 | S4 | S4 | 191 | 122,962 | 188 |
| **PENTHORACEAE** |  |  |  |  |  |  |  |  |  |  |  |
| *Penthorum sedoides* | S5 | S5 | S3 | S3 | S3 | S3 | S3 | S3 | 41 | 106,932 | 41 |
| **PHRYMACEAE** |  |  |  |  |  |  |  |  |  |  |  |
| *Mimulus alatus* |  | S5 | S4 | S4 | S3 | S4 | S4 | S4 | 129 | 117,168 | 123 |
| *Mimulus ringens* | S1 | S5 | S3 | S3 | S3 | S3 | S3 | S3 | 75 | 87,826 | 73 |
| *Phryma leptostachya* | S5 | S5 | S3 | S4 | S3 | S4 | S3 | S4 | 116 | 110,956 | 105 |
| **PHYLLANTHACEAE** |  |  |  |  |  |  |  |  |  |  |  |
| *Phyllanthopsis phyllanthoides* |  | S1 | S1 | S1 | S1 | S1 | S1 | S1 | 1 | <1 | 1 |
| *Phyllanthus caroliniensis* | S4 | S4 | S2 | S3 | S2 | S3 | S2 | S3 | 17 | 91,779 | 17 |
| **PHYTOLACCACEAE** |  |  |  |  |  |  |  |  |  |  |  |
| *Phytolacca americana* | S5 | S5 | S4 | S5 | S4 | S5 | S4 | S5 | 913 | 130,873 | 800 |
| **PLANTAGINACEAE** |  |  |  |  |  |  |  |  |  |  |  |
| *Bacopa caroliniana* |  | S1 |  |  |  |  |  |  |  |  |  |
| *Bacopa rotundifolia* |  | S3 | S1 | S2 | S1 | S2 | S1 | S2 | 5 | 7,473 | 5 |
| *Callitriche heterophylla* | S5 | S5 | S2 | S3 | S2 | S2 | S2 | S3 | 7 | 24,176 | 7 |
| *Callitriche terrestris* |  | S4 | S1 | S1 | S1 | S1 | S1 | S1 | 1 | <1 | 1 |
| *Chelone glabra* |  | S5 | S3 | S4 | S3 | S4 | S3 | S4 | 91 | 94,756 | 88 |
| *Chelone lyonii* | S1 | S3 | S3 | S3 | S3 | S3 | S3 | S3 | 68 | 53,453 | 60 |
| *Chelone obliqua* | S2 | S2 | S1 | S1 | S1 | S1 | S1 | S1 | 4 | 2,344 | 4 |
| *Collinsia verna* | S1 | S2 | S3 | S3 | S3 | S3 | S3 | S3 | 26 | 38,661 | 24 |
| *Gratiola brevifolia* | S3 | S2 |  |  | S1 | S2 | S2 | S2 | 3 | 2,340 | 3 |
| *Gratiola floridana* | S1 | S2 | S1 | S1 | S1 | S1 | S1 | S1 | 2 | 4 | 2 |
| *Gratiola neglecta* |  | S5 | S2 | S3 | S2 | S3 | S2 | S3 | 18 | 98,528 | 16 |
| *Gratiola quartermaniae* |  | S2 | S2 | S2 | S2 | S2 | S2 | S2 | 13 | 2,035 | 11 |
| *Gratiola virginiana* | S5 | S5 | S2 | S3 | S2 | S3 | S2 | S3 | 13 | 60,927 | 13 |
| *Gratiola viscidula* | S2 | S2 | S1 | S1 | S1 | S1 | S1 | S1 | 4 | 2,323 | 4 |
| *Leucospora multifida* | S3 | S5 | S3 | S3 | S3 | S3 | S3 | S3 | 34 | 78,415 | 32 |
| *Linaria canadensis* |  | S4 | S2 | S3 | S2 | S3 | S2 | S3 | 17 | 49,370 | 17 |
| *Linaria texana* |  | S1 | S2 | S3 | S3 | S3 | S2 | S3 | 20 | 43,079 | 20 |
| *Mecardonia acuminata* | S5 | S5 | S3 | S3 | S3 | S3 | S3 | S3 | 36 | 43,285 | 33 |
| *Penstemon alluviorum* |  | S2 |  |  |  |  |  |  |  |  |  |
| *Penstemon australis* |  | S3 | S1 | S1 | S1 | S1 | S1 | S1 | 2 | 29 | 2 |
| *Penstemon brevisepalus* |  | S5 | S3 | S3 | S3 | S3 | S3 | S3 | 26 | 33,140 | 24 |
| *Penstemon calycosus* |  | S5 | S3 | S3 | S3 | S3 | S3 | S3 | 59 | 65,589 | 58 |
| *Penstemon canescens* |  | S5 | S3 | S3 | S3 | S3 | S3 | S3 | 55 | 47,153 | 54 |
| *Penstemon digitalis* | S5 | S4 | S3 | S3 | S3 | S3 | S3 | S3 | 62 | 104,365 | 56 |
| *Penstemon hirsutus* |  | S4 | S2 | S3 | S2 | S3 | S2 | S3 | 7 | 30,397 | 7 |
| *Penstemon kralii* |  | S1 | S1 | S1 | S1 | S1 | S1 | S1 | 1 | <1 | 1 |
| *Penstemon laevigatus* | S5 | S5 | S2 | S3 | S2 | S3 | S2 | S3 | 10 | 47,567 | 9 |
| *Penstemon pallidus* | S1 | S4 | S2 | S3 | S2 | S3 | S2 | S3 | 18 | 60,951 | 18 |
| *Penstemon smallii* | S1 | S3 | S2 | S2 | S2 | S2 | S2 | S2 | 16 | 12,686 | 15 |
| *Penstemon tenuiflorus* |  | S4 | S3 | S3 | S3 | S3 | S3 | S3 | 56 | 34,025 | 54 |
| *Penstemon tubaeflorus* |  | S2 |  |  | S1 | S1 | S1 | S1 | 3 | 74 | 3 |
| *Plantago aristata* | S5 | S5 | S3 | S3 | S3 | S3 | S3 | S3 | 52 | 76,504 | 51 |
| *Plantago cordata* |  | S1 | S1 | S1 | S1 | S1 | S1 | S1 | 1 | <1 | 1 |
| *Plantago heterophylla* |  | S2 | S1 | S1 | S1 | S1 | S1 | S1 | 1 | <1 | 1 |
| *Plantago pusilla* |  | S4 | S1 | S2 | S1 | S2 | S2 | S2 | 3 | 16,969 | 3 |
| *Plantago rugelii* | S5 | S5 | S4 | S4 | S4 | S4 | S4 | S4 | 242 | 128,817 | 237 |
| *Plantago virginica* | S5 | S5 | S3 | S3 | S3 | S3 | S3 | S3 | 42 | 108,991 | 40 |
| *Sophronanthe pilosa* | S4 | S3 | S1 | S1 | S1 | S1 | S1 | S1 | 4 | 2,804 | 4 |
| *Veronica americana* |  | S1 | S1 | S1 | S1 | S1 | S1 | S1 | 4 | 1,778 | 4 |
| *Veronica anagallis-aquatica* |  | S4 | S2 | S2 | S2 | S2 | S2 | S2 | 13 | 16,849 | 14 |
| *Veronica catenata* |  | S1 | S1 | S1 | S1 | S1 | S1 | S1 | 1 | <1 | 1 |
| *Veronica peregrina* | S5 | S5 | S3 | S3 | S3 | S3 | S3 | S3 | 27 | 91,479 | 26 |
| *Veronica scutellata* |  | S1 |  |  | S1 | S1 | S1 | S1 | 1 | <1 | 1 |
| *Veronicastrum virginicum* |  | S4 | S3 | S3 | S3 | S3 | S3 | S3 | 25 | 93,352 | 23 |
| **PLATANACEAE** |  |  |  |  |  |  |  |  |  |  |  |
| *Platanus occidentalis* | S5 | S5 | S4 | S5 | S4 | S5 | S4 | S5 | 548 | 132,776 | 492 |
| **PODOSTEMACEAE** |  |  |  |  |  |  |  |  |  |  |  |
| *Podostemum ceratophyllum* | S5 | S4 | S2 | S3 | S2 | S3 | S2 | S3 | 12 | 30,996 | 12 |
| **POLEMONIACEAE** |  |  |  |  |  |  |  |  |  |  |  |
| *Phlox amoena* | S3 | S5 | S3 | S4 | S3 | S4 | S3 | S4 | 86 | 63,160 | 83 |
| *Phlox amplifolia* | S2 | S4 | S2 | S2 | S2 | S3 | S2 | S3 | 11 | 17,496 | 10 |
| *Phlox bifida* |  | S1 | S3 | S3 | S3 | S3 | S3 | S3 | 48 | 3,660 | 42 |
| *Phlox divaricata* |  | S5 | S4 | S5 | S4 | S5 | S4 | S5 | 424 | 120,377 | 363 |
| *Phlox glaberrima* |  | S5 | S3 | S3 | S3 | S3 | S3 | S3 | 38 | 60,283 | 40 |
| *Phlox maculata* |  | S4 | S3 | S3 | S3 | S3 | S3 | S3 | 27 | 55,704 | 27 |
| *Phlox ovata* | S4 | S3 | S1 | S2 | S1 | S2 | S1 | S2 | 3 | 9,680 | 3 |
| *Phlox paniculata* | S5 | S5 | S4 | S4 | S4 | S4 | S4 | S4 | 207 | 118,712 | 189 |
| *Phlox pilosa* |  | S5 | S3 | S3 | S3 | S3 | S3 | S3 | 71 | 88,348 | 69 |
| *Phlox stolonifera* | S3 | S3 | S3 | S3 | S3 | S3 | S3 | S3 | 73 | 53,311 | 52 |
| *Phlox subulata* |  | S4 | S3 | S3 | S3 | S3 | S3 | S3 | 48 | 84,862 | 45 |
| *Polemonium reptans* | S1 | S5 | S3 | S4 | S3 | S4 | S3 | S4 | 107 | 108,438 | 94 |
| **POLYGALACEAE** |  |  |  |  |  |  |  |  |  |  |  |
| *Polygaloides paucifolia* |  | S1 | S3 | S3 | S3 | S3 | S3 | S3 | 42 | 31,352 | 40 |
| *Senega ambigua* |  | S5 | S2 | S3 | S2 | S3 | S2 | S3 | 14 | 51,271 | 14 |
| *Senega boykinii* | S3 | S1 | S1 | S1 | S2 | S2 | S2 | S2 | 5 | 2,101 | 5 |
| *Senega cruciata* |  | S4 | S3 | S3 | S3 | S3 | S3 | S3 | 26 | 20,873 | 26 |
| *Senega curtissii* | S5 | S5 | S3 | S3 | S3 | S4 | S3 | S4 | 84 | 64,649 | 80 |
| *Senega incarnata* | S4 | S4 | S3 | S3 | S3 | S3 | S3 | S3 | 21 | 36,644 | 21 |
| *Senega mariana* | S4 | S3 | S2 | S2 | S2 | S2 | S2 | S3 | 6 | 18,850 | 6 |
| *Senega nana* |  | S1 |  |  | S1 | S1 | S1 | S1 | 3 | <1 | 2 |
| *Senega nuttallii* |  | S1 |  |  | S1 | S1 | S1 | S1 | 1 | <1 | 1 |
| *Senega officinalis* | S2 | S4 | S2 | S3 | S2 | S3 | S2 | S3 | 17 | 54,763 | 16 |
| *Senega polygama* | S2 | S2 | S1 | S1 | S1 | S1 | S1 | S1 | 3 | 465 | 3 |
| *Senega sanguinea* |  | S5 | S3 | S3 | S3 | S3 | S3 | S3 | 31 | 63,507 | 30 |
| *Senega verticillata* | S5 | S4 | S3 | S3 | S3 | S3 | S3 | S3 | 24 | 53,258 | 24 |
| **POLYGONACEAE** |  |  |  |  |  |  |  |  |  |  |  |
| *Brunnichia ovata* |  | S4 | S3 | S3 | S3 | S3 | S3 | S3 | 33 | 36,465 | 31 |
| *Fallopia scandens* |  | S4 | S3 | S3 | S3 | S3 | S3 | S3 | 52 | 114,599 | 51 |
| *Parogonum ciliinode* |  | S1 |  |  |  |  |  |  |  |  |  |
| *Persicaria amphibia* |  | S3 | S2 | S3 | S2 | S3 | S2 | S3 | 12 | 47,014 | 12 |
| *Persicaria arifolia* |  | S1 | S1 | S1 | S1 | S1 | S1 | S1 | 1 | <1 | 1 |
| *Persicaria bicornis* |  | S1 | S1 | S2 | S1 | S2 | S2 | S2 | 4 | 15,030 | 4 |
| *Persicaria glabra* |  | S2 | S1 | S1 | S1 | S1 | S1 | S1 | 3 | 1,094 | 3 |
| *Persicaria hydropiperoides* | S5 | S4 | S2 | S3 | S2 | S3 | S2 | S3 | 16 | 71,022 | 16 |
| *Persicaria lapathifolia* | S5 | S4 | S3 | S3 | S3 | S3 | S3 | S3 | 34 | 98,396 | 33 |
| *Persicaria pensylvanica* | S5 | S5 | S3 | S3 | S3 | S3 | S3 | S3 | 26 | 62,719 | 26 |
| *Persicaria punctata* | S5 | S5 | S2 | S3 | S3 | S3 | S2 | S3 | 20 | 54,290 | 20 |
| *Persicaria sagittata* | S5 | S4 | S3 | S3 | S3 | S3 | S3 | S3 | 26 | 100,649 | 26 |
| *Persicaria setacea* |  | S3 | S2 | S3 | S2 | S3 | S2 | S3 | 13 | 69,760 | 13 |
| *Persicaria virginiana* | S5 | S4 | S4 | S4 | S4 | S4 | S4 | S4 | 279 | 126,477 | 250 |
| *Polygonella americana* | S1 | S1 | S1 | S1 | S1 | S1 | S1 | S1 | 4 | 10 | 4 |
| *Polygonum erectum* |  | S4 | S1 | S2 | S1 | S2 | S2 | S2 | 5 | 15,676 | 5 |
| *Polygonum tenue* |  | S3 | S1 | S1 | S1 | S1 | S1 | S1 | 4 | 2,250 | 4 |
| *Rumex altissimus* | S3 | S4 | S2 | S3 | S2 | S3 | S2 | S3 | 11 | 44,205 | 11 |
| *Rumex verticillatus* | S5 | S4 | S2 | S2 | S2 | S2 | S2 | S3 | 7 | 15,546 | 7 |
| **PORTULACACEAE** |  |  |  |  |  |  |  |  |  |  |  |
| *Portulaca pilosa* | S3 | S1 | S2 | S1 | S2 | S1 | S2 | S1 | 7 | 581 | 7 |
| **PRIMULACEAE** |  |  |  |  |  |  |  |  |  |  |  |
| *Hottonia inflata* | S1 | S2 |  |  | S2 | S3 | S2 | S3 | 6 | 1,286 | 5 |
| *Primula meadia* | S2 | S4 | S4 | S4 | S4 | S4 | S4 | S4 | 141 | 64,569 | 129 |
| **RANUNCULACEAE** |  |  |  |  |  |  |  |  |  |  |  |
| *Aconitum reclinatum* | S3 | S1 |  |  | S1 | S1 | S1 | S1 | 1 | <1 | 1 |
| *Aconitum uncinatum* | S1 | S4 | S2 | S3 | S2 | S3 | S2 | S3 | 15 | 48,372 | 15 |
| *Actaea pachypoda* | S5 | S5 | S4 | S4 | S4 | S4 | S4 | S4 | 203 | 118,076 | 175 |
| *Actaea podocarpa* | S4 | S1 | S2 | S2 | S2 | S2 | S2 | S2 | 16 | 4,784 | 14 |
| *Actaea racemosa* | S4 | S1 | S4 | S4 | S4 | S4 | S4 | S4 | 151 | 99,455 | 136 |
| *Actaea rubifolia* | S1 | S5 | S1 | S2 | S1 | S2 | S1 | S2 | 4 | 6,043 | 4 |
| *Anemone caroliniana* | S1 | S2 | S2 | S2 | S2 | S2 | S2 | S2 | 13 | 1,451 | 14 |
| *Anemone lancifolia* |  | S3 | S1 | S1 | S1 | S1 | S1 | S1 | 3 | 461 | 3 |
| *Anemone minima* |  | S1 |  |  |  |  |  |  |  |  |  |
| *Anemone quinquefolia* |  | S4 | S4 | S4 | S4 | S4 | S4 | S4 | 147 | 88,478 | 131 |
| *Anemone virginiana* | S5 | S5 | S4 | S4 | S4 | S4 | S4 | S4 | 129 | 98,141 | 115 |
| *Aquilegia canadensis* | S5 | S5 | S4 | S4 | S4 | S4 | S4 | S4 | 268 | 89,856 | 241 |
| *Caltha palustris* |  | S1 | S2 | S2 | S2 | S1 | S2 | S2 | 8 | 1,008 | 8 |
| *Clematis catesbyana* | S2 | S4 | S2 | S2 | S2 | S2 | S2 | S3 | 10 | 18,195 | 9 |
| *Clematis crispa* | S4 | S3 | S2 | S2 | S2 | S2 | S2 | S3 | 7 | 17,158 | 7 |
| *Clematis cumberlandensis* |  | S2 | S2 | S2 | S2 | S2 | S2 | S2 | 6 | 5,247 | 6 |
| *Clematis fremontii* | S1 | S1 | S2 | S1 | S2 | S1 | S2 | S1 | 17 | 329 | 15 |
| *Clematis glaucophylla* | S1 | S3 | S2 | S2 | S2 | S2 | S2 | S2 | 11 | 12,656 | 10 |
| *Clematis morefieldii* |  | S1 |  |  | S2 | S2 | S2 | S2 | 24 | 683 | 24 |
| *Clematis reticulata* |  | S1 | S1 | S1 | S1 | S1 | S1 | S1 | 2 | 64 | 2 |
| *Clematis subreticulata* |  | S1 |  |  |  |  |  |  |  |  |  |
| *Clematis versicolor* |  | S4 | S3 | S3 | S3 | S3 | S3 | S3 | 33 | 24,749 | 30 |
| *Clematis vinacea* |  | S1 | S2 | S1 | S2 | S1 | S2 | S1 | 8 | 153 | 7 |
| *Clematis viorna* |  | S5 | S3 | S3 | S3 | S3 | S3 | S3 | 35 | 63,970 | 35 |
| *Clematis virginiana* | S5 | S5 | S3 | S4 | S3 | S4 | S3 | S4 | 107 | 124,082 | 104 |
| *Delphinium carolinianum* |  | S3 | S3 | S3 | S3 | S3 | S3 | S3 | 40 | 10,431 | 36 |
| *Delphinium exaltatum* | S2 | S1 | S1 | S1 | S1 | S1 | S1 | S1 | 3 | 248 | 3 |
| *Delphinium tricorne* | S2 | S5 | S4 | S4 | S4 | S4 | S4 | S4 | 228 | 113,068 | 201 |
| *Enemion biternatum* |  | S4 | S3 | S3 | S3 | S3 | S3 | S3 | 28 | 40,277 | 25 |
| *Hepatica acutiloba* |  | S5 | S4 | S4 | S4 | S4 | S4 | S4 | 238 | 97,313 | 200 |
| *Hepatica americana* | S5 | S5 | S3 | S4 | S3 | S4 | S3 | S4 | 92 | 88,805 | 89 |
| *Hydrastis canadensis* |  | S5 | S3 | S4 | S3 | S4 | S3 | S4 | 118 | 117,831 | 117 |
| *Myosurus minimus* |  | S5 | S2 | S2 | S2 | S2 | S2 | S2 | 4 | 34,502 | 4 |
| *Ranunculus abortivus* | S5 | S5 | S3 | S4 | S3 | S4 | S3 | S4 | 84 | 113,654 | 81 |
| *Ranunculus allegheniensis* |  | S2 | S1 | S1 | S1 | S1 | S1 | S1 | 5 | 160 | 5 |
| *Ranunculus ambigens* |  | S2 | S1 | S1 | S1 | S1 | S1 | S1 | 1 | <1 | 1 |
| *Ranunculus fascicularis* |  | S5 | S3 | S3 | S3 | S3 | S3 | S3 | 34 | 81,915 | 33 |
| *Ranunculus flabellaris* | S1 | S3 |  |  | S2 | S3 | S2 | S3 | 7 | 30,214 | 7 |
| *Ranunculus harveyi* |  | S2 | S1 | S1 | S1 | S1 | S1 | S1 | 3 | 335 | 3 |
| *Ranunculus hispidus* | S5 | S5 | S3 | S3 | S3 | S3 | S3 | S3 | 34 | 64,410 | 31 |
| *Ranunculus laxicaulis* |  | S3 | S1 | S1 | S1 | S1 | S1 | S1 | 4 | 446 | 4 |
| *Ranunculus longirostris* | S2 | S1 | S1 | S1 | S1 | S1 | S1 | S1 | 1 | <1 | 1 |
| *Ranunculus micranthus* | S4 | S4 | S2 | S3 | S2 | S3 | S2 | S3 | 17 | 44,546 | 18 |
| *Ranunculus pusillus* | S5 | S5 | S2 | S3 | S2 | S3 | S2 | S3 | 7 | 37,517 | 7 |
| *Ranunculus recurvatus* | S5 | S5 | S3 | S4 | S3 | S4 | S3 | S4 | 115 | 114,448 | 105 |
| *Ranunculus sceleratus* |  | S3 | S2 | S2 | S2 | S2 | S2 | S2 | 5 | 33,527 | 5 |
| *Ranunculus septentrionalis* |  | S4 | S1 | S1 | S1 | S1 | S1 | S1 | 2 | <1 | 2 |
| *Thalictrum amphibolum* | S4 | S4 | S2 | S3 | S2 | S3 | S2 | S3 | 17 | 35,434 | 17 |
| *Thalictrum clavatum* | S4 | S4 | S3 | S3 | S3 | S3 | S3 | S3 | 36 | 40,661 | 33 |
| *Thalictrum coriaceum* | S4 | S3 |  |  |  |  |  |  |  |  |  |
| *Thalictrum dioicum* |  | S5 | S3 | S3 | S3 | S3 | S3 | S3 | 37 | 102,169 | 36 |
| *Thalictrum mirabile* |  | S2 | S1 | S1 | S1 | S1 | S1 | S1 | 4 | 3,473 | 4 |
| *Thalictrum pubescens* | S5 | S4 | S2 | S3 | S2 | S3 | S2 | S3 | 17 | 34,831 | 15 |
| *Thalictrum thalictroides* | S5 | S5 | S4 | S5 | S4 | S5 | S4 | S5 | 569 | 110,447 | 487 |
| *Trautvetteria applanata* | S1 | S1 |  |  | S2 | S3 | S2 | S3 | 13 | 21,007 | 11 |
| *Trautvetteria caroliniensis* | S5 | S4 | S3 | S3 | S3 | S3 | S3 | S3 | 51 | 71,520 | 52 |
| *Trautvetteria fonticalcarea* |  | S1 | S2 | S1 | S2 | S1 | S2 | S1 | 11 | 415 | 13 |
| *Xanthorhiza simplicissima* | S5 | S4 | S4 | S4 | S3 | S4 | S4 | S4 | 128 | 74,847 | 110 |
| **RHAMNACEAE** |  |  |  |  |  |  |  |  |  |  |  |
| *Berchemia scandens* | S5 | S4 | S3 | S3 | S3 | S3 | S3 | S3 | 51 | 78,920 | 46 |
| *Ceanothus americanus* | S5 | S5 | S3 | S4 | S3 | S4 | S3 | S4 | 117 | 95,633 | 113 |
| *Ceanothus herbaceus* |  | S1 |  |  |  |  |  |  |  |  |  |
| *Endotropis alnifolia* |  | S1 | S1 | S1 | S1 | S1 | S1 | S1 | 1 | <1 | 1 |
| *Endotropis lanceolata* |  | S3 | S2 | S2 | S2 | S2 | S2 | S2 | 7 | 9,376 | 7 |
| *Frangula caroliniana* | S3 | S5 | S4 | S5 | S4 | S5 | S4 | S5 | 415 | 110,321 | 384 |
| **ROSACEAE** |  |  |  |  |  |  |  |  |  |  |  |
| *Agrimonia gryposepala* |  | S3 | S2 | S3 | S2 | S3 | S2 | S3 | 11 | 59,127 | 10 |
| *Agrimonia microcarpa* | S4 | S2 | S1 | S1 | S1 | S1 | S1 | S1 | 2 | 2 | 2 |
| *Agrimonia parviflora* | S5 | S5 | S3 | S4 | S3 | S4 | S3 | S4 | 123 | 109,812 | 116 |
| *Agrimonia pubescens* |  | S5 | S2 | S3 | S3 | S3 | S2 | S3 | 20 | 62,470 | 20 |
| *Agrimonia rostellata* |  | S5 | S2 | S3 | S2 | S3 | S2 | S3 | 14 | 68,176 | 14 |
| *Amelanchier arborea* | S5 | S5 | S3 | S3 | S3 | S3 | S3 | S3 | 55 | 103,688 | 51 |
| *Amelanchier canadensis* |  | S4 | S2 | S2 | S2 | S2 | S2 | S3 | 8 | 18,701 | 8 |
| *Amelanchier laevis* | S3 | S5 | S2 | S3 | S2 | S3 | S2 | S3 | 16 | 29,308 | 16 |
| *Amelanchier sanguinea* |  | S3 |  |  | S2 | S2 | S3 | S2 | 9 | 8,387 | 9 |
| *Amelanchier spicata* |  | S2 | S1 | S2 | S1 | S2 | S1 | S2 | 3 | 13,791 | 3 |
| *Aronia × prunifolia* |  | S2 | S2 | S2 | S2 | S2 | S2 | S2 | 7 | 15,120 | 7 |
| *Aronia arbutifolia* | S5 | S4 | S3 | S3 | S3 | S3 | S3 | S3 | 21 | 30,014 | 21 |
| *Aronia melanocarpa* |  | S4 | S3 | S3 | S3 | S3 | S3 | S3 | 30 | 37,016 | 28 |
| *Aruncus dioicus* | S4 | S5 | S3 | S3 | S3 | S3 | S3 | S3 | 57 | 78,969 | 55 |
| *Crataegus × disperma* |  | S1 |  |  |  |  |  |  |  |  |  |
| *Crataegus × vailiae* |  |  |  |  |  |  |  |  |  |  |  |
| *Crataegus alleghaniensis* |  | S1 |  |  |  |  |  |  |  |  |  |
| *Crataegus aprica* |  | S1 |  |  |  |  |  |  |  |  |  |
| *Crataegus beata* |  | S1 |  |  |  |  |  |  |  |  |  |
| *Crataegus berberifolia* |  | S2 | S1 | S1 | S1 | S1 | S1 | S1 | 1 | <1 | 1 |
| *Crataegus calpodendron* |  | S4 | S2 | S3 | S2 | S3 | S2 | S3 | 9 | 31,527 | 9 |
| *Crataegus coccinioides* |  | S1 |  |  |  |  |  |  |  |  |  |
| *Crataegus collina* |  | S4 | S2 | S3 | S2 | S2 | S2 | S3 | 9 | 22,575 | 9 |
| *Crataegus crus-galli* |  | S5 | S2 | S3 | S2 | S3 | S2 | S3 | 18 | 46,940 | 18 |
| *Crataegus intricata* | S4 | S4 | S2 | S3 | S2 | S3 | S2 | S3 | 16 | 43,107 | 16 |
| *Crataegus iracunda* |  | S2 | S1 | S1 | S1 | S1 | S1 | S1 | 2 | 49 | 2 |
| *Crataegus macrosperma* |  | S4 | S2 | S3 | S2 | S3 | S2 | S3 | 17 | 43,588 | 16 |
| *Crataegus margaretiae* |  | S1 |  |  |  |  |  |  |  |  |  |
| *Crataegus marshallii* |  | S2 | S1 | S1 | S1 | S1 | S1 | S1 | 2 | 98 | 2 |
| *Crataegus mollis* | S1 | S2 | S1 | S1 | S1 | S1 | S1 | S1 | 3 | 218 | 3 |
| *Crataegus phaenopyrum* | S3 | S3 | S1 | S1 | S1 | S1 | S1 | S1 | 4 | 2,898 | 4 |
| *Crataegus pruinosa* |  | S3 | S1 | S2 | S1 | S2 | S1 | S2 | 5 | 18,327 | 5 |
| *Crataegus punctata* | S4 | S2 | S1 | S1 | S1 | S1 | S1 | S1 | 4 | 3,495 | 4 |
| *Crataegus reverchonii* |  | S1 |  |  |  |  |  |  |  |  |  |
| *Crataegus schuettei* |  | S3 | S1 | S1 | S1 | S1 | S1 | S1 | 1 | <1 | 1 |
| *Crataegus spathulata* | S1 | S2 | S1 | S1 | S1 | S1 | S1 | S1 | 3 | 4 | 2 |
| *Crataegus uniflora* | S5 | S3 | S1 | S1 | S1 | S1 | S1 | S1 | 1 | <1 | 1 |
| *Crataegus viridis* | S4 | S4 | S2 | S2 | S2 | S2 | S2 | S2 | 5 | 24,637 | 5 |
| *Fragaria americana* |  | S1 | S1 | S1 | S1 | S1 | S1 | S1 | 1 | <1 | 1 |
| *Fragaria virginiana* | S5 | S5 | S3 | S3 | S3 | S3 | S3 | S3 | 60 | 85,629 | 56 |
| *Geum aleppicum* |  | S1 |  |  | S1 | S1 | S1 | S1 | 1 | <1 | 1 |
| *Geum canadense* | S5 | S5 | S4 | S4 | S4 | S4 | S4 | S4 | 185 | 127,409 | 171 |
| *Geum geniculatum* | S1 | S1 | S1 | S1 | S1 | S1 | S1 | S1 | 3 | 39 | 3 |
| *Geum laciniatum* | S1 | S1 |  |  | S1 | S1 | S1 | S1 | 1 | <1 | 1 |
| *Geum radiatum* |  | S1 | S2 | S1 | S1 | S1 | S2 | S1 | 8 | 165 | 8 |
| *Geum vernum* | S1 | S5 | S3 | S3 | S3 | S3 | S3 | S3 | 31 | 53,669 | 27 |
| *Geum virginianum* | S5 | S5 | S2 | S3 | S2 | S3 | S2 | S3 | 18 | 52,190 | 17 |
| *Gillenia stipulata* |  | S5 | S3 | S3 | S3 | S3 | S3 | S3 | 69 | 80,043 | 63 |
| *Gillenia trifoliata* |  | S4 | S3 | S4 | S3 | S4 | S3 | S4 | 117 | 49,891 | 111 |
| *Malus angustifolia* | S4 | S4 | S2 | S3 | S2 | S3 | S2 | S3 | 19 | 65,228 | 19 |
| *Malus coronaria* |  | S4 | S2 | S2 | S2 | S2 | S2 | S2 | 11 | 1,859 | 11 |
| *Neviusia alabamensis* | S1 | S2 |  |  | S2 | S2 | S2 | S2 | 11 | 6,633 | 11 |
| *Physocarpus opulifolius* |  | S5 | S3 | S3 | S3 | S3 | S3 | S3 | 36 | 78,376 | 36 |
| *Potentilla canadensis* | S5 | S5 | S3 | S3 | S3 | S3 | S3 | S3 | 64 | 54,972 | 61 |
| *Potentilla norvegica* | S4 | S4 | S2 | S2 | S2 | S2 | S2 | S2 | 6 | 9,423 | 5 |
| *Potentilla simplex* | S5 | S5 | S3 | S4 | S3 | S4 | S3 | S4 | 90 | 110,186 | 87 |
| *Prunus americana* |  | S5 | S3 | S3 | S3 | S3 | S3 | S3 | 35 | 101,005 | 32 |
| *Prunus angustifolia* | S5 | S5 | S2 | S3 | S2 | S3 | S2 | S3 | 16 | 71,937 | 15 |
| *Prunus hortulana* |  | S4 | S1 | S1 | S1 | S1 | S1 | S1 | 1 | <1 | 1 |
| *Prunus mexicana* |  | S4 | S2 | S2 | S2 | S2 | S2 | S3 | 11 | 17,210 | 11 |
| *Prunus munsoniana* |  | S4 | S2 | S3 | S2 | S3 | S2 | S3 | 9 | 35,057 | 9 |
| *Prunus pensylvanica* |  | S2 | S2 | S2 | S2 | S2 | S2 | S2 | 19 | 4,917 | 18 |
| *Prunus pumila* | S1 | S1 | S1 | S1 | S1 | S1 | S1 | S1 | 1 | <1 | 1 |
| *Prunus serotina* | S5 | S5 | S4 | S4 | S4 | S4 | S4 | S4 | 228 | 128,272 | 220 |
| *Prunus umbellata* |  | S2 | S1 | S1 | S1 | S1 | S1 | S1 | 2 | 47 | 2 |
| *Prunus virginiana* |  | S3 | S2 | S3 | S2 | S3 | S2 | S3 | 8 | 32,702 | 8 |
| *Rosa carolina* |  | S5 | S3 | S3 | S3 | S3 | S3 | S3 | 53 | 76,792 | 51 |
| *Rosa palustris* | S5 | S5 | S3 | S3 | S3 | S3 | S3 | S3 | 29 | 114,952 | 28 |
| *Rosa setigera* |  | S5 | S3 | S3 | S3 | S3 | S3 | S3 | 72 | 75,756 | 71 |
| *Rubus allegheniensis* |  | S5 | S3 | S3 | S3 | S3 | S3 | S3 | 48 | 92,115 | 48 |
| *Rubus argutus* |  | S5 | S2 | S3 | S2 | S3 | S2 | S3 | 11 | 40,512 | 11 |
| *Rubus canadensis* |  | S3 | S2 | S2 | S2 | S2 | S2 | S2 | 15 | 9,558 | 12 |
| *Rubus flagellaris* | S5 | S5 | S3 | S3 | S3 | S3 | S3 | S3 | 49 | 100,586 | 48 |
| *Rubus hispidus* | S5 | S4 | S2 | S3 | S2 | S3 | S2 | S3 | 13 | 42,721 | 13 |
| *Rubus occidentalis* |  | S5 | S3 | S3 | S3 | S3 | S3 | S3 | 66 | 105,291 | 65 |
| *Rubus odoratus* | S4 | S1 | S3 | S3 | S3 | S3 | S3 | S3 | 71 | 36,970 | 63 |
| *Rubus pensilvanicus* |  | S3 | S3 | S3 | S3 | S3 | S3 | S3 | 40 | 102,240 | 38 |
| *Rubus strigosus* | S2 | S1 |  |  |  |  |  |  |  |  |  |
| *Rubus trivialis* | S3 | S4 | S2 | S3 | S2 | S3 | S2 | S3 | 15 | 65,268 | 15 |
| *Sanguisorba canadensis* | S1 | S1 |  |  | S1 | S1 | S1 | S1 | 2 | 3 | 2 |
| *Sibbaldiopsis tridentata* |  | S1 |  |  | S1 | S1 | S1 | S1 | 2 | <1 | 2 |
| *Sorbus americana* |  | S3 | S2 | S3 | S2 | S3 | S3 | S3 | 24 | 2,916 | 24 |
| *Spiraea alba* |  | S2 |  |  | S2 | S2 | S2 | S2 | 9 | 1,102 | 9 |
| *Spiraea tomentosa* | S1 | S4 | S3 | S3 | S3 | S3 | S3 | S3 | 31 | 33,677 | 30 |
| *Spiraea virginiana* | S1 | S2 |  |  | S3 | S3 | S3 | S3 | 27 | 17,499 | 26 |
| *Waldsteinia doniana* |  | S1 | S1 | S1 | S1 | S1 | S1 | S1 | 1 | <1 | 1 |
| *Waldsteinia fragarioides* |  | S4 | S2 | S3 | S2 | S3 | S2 | S3 | 11 | 32,111 | 11 |
| **RUBIACEAE** |  |  |  |  |  |  |  |  |  |  |  |
| *Cephalanthus occidentalis* | S5 | S5 | S4 | S4 | S4 | S4 | S4 | S4 | 276 | 127,220 | 259 |
| *Diodia virginiana* | S5 | S5 | S4 | S4 | S4 | S4 | S4 | S4 | 181 | 115,951 | 176 |
| *Galium aparine* | S5 | S5 | S4 | S4 | S4 | S4 | S4 | S4 | 221 | 116,307 | 206 |
| *Galium asprellum* |  | S2 | S1 | S1 | S1 | S1 | S1 | S1 | 1 | <1 | 1 |
| *Galium circaezans* | S5 | S5 | S3 | S3 | S3 | S3 | S3 | S3 | 76 | 101,048 | 72 |
| *Galium concinnum* |  | S2 | S1 | S1 | S1 | S1 | S1 | S1 | 2 | 69 | 2 |
| *Galium lanceolatum* |  | S4 | S2 | S2 | S2 | S2 | S2 | S2 | 11 | 12,699 | 10 |
| *Galium latifolium* | S5 | S4 | S2 | S2 | S2 | S2 | S2 | S2 | 8 | 4,006 | 8 |
| *Galium obtusum* |  | S5 | S2 | S2 | S2 | S2 | S2 | S3 | 6 | 18,630 | 6 |
| *Galium orizabense* |  | S1 | S1 | S1 | S1 | S1 | S1 | S1 | 1 | <1 | 1 |
| *Galium palustre* |  | S1 |  |  | S1 | S1 | S1 | S1 | 1 | <1 | 1 |
| *Galium pilosum* | S5 | S5 | S3 | S3 | S3 | S3 | S3 | S3 | 44 | 79,513 | 43 |
| *Galium tinctorium* |  | S5 | S2 | S3 | S2 | S3 | S2 | S3 | 17 | 42,711 | 17 |
| *Galium triflorum* | S5 | S5 | S3 | S3 | S3 | S3 | S3 | S3 | 55 | 67,640 | 49 |
| *Galium uniflorum* | S4 | S2 | S1 | S1 | S1 | S1 | S1 | S1 | 3 | 100 | 3 |
| *Galium virgatum* |  | S1 | S1 | S1 | S1 | S1 | S1 | S1 | 4 | 116 | 3 |
| *Hedyotis nigricans* |  | S3 | S2 | S2 | S2 | S2 | S2 | S2 | 14 | 3,264 | 14 |
| *Hexasepalum teres* | S5 | S3 | S3 | S3 | S3 | S3 | S3 | S3 | 52 | 102,848 | 51 |
| *Houstonia caerulea* | S5 | S5 | S4 | S4 | S4 | S4 | S4 | S4 | 258 | 110,524 | 244 |
| *Houstonia canadensis* | S1 | S4 | S2 | S3 | S2 | S3 | S2 | S3 | 12 | 38,728 | 12 |
| *Houstonia lanceolata* | S4 | S4 | S2 | S2 | S2 | S2 | S2 | S3 | 10 | 17,341 | 10 |
| *Houstonia longifolia* |  | S4 | S3 | S3 | S3 | S3 | S3 | S3 | 47 | 72,132 | 46 |
| *Houstonia micrantha* |  | S2 | S3 | S3 | S3 | S3 | S3 | S3 | 26 | 35,814 | 24 |
| *Houstonia montana* |  | S1 |  |  | S1 | S1 | S1 | S1 | 1 | <1 | 1 |
| *Houstonia purpurea* | S5 | S5 | S4 | S5 | S4 | S5 | S4 | S5 | 388 | 107,237 | 342 |
| *Houstonia pusilla* | S4 | S5 | S4 | S4 | S4 | S4 | S4 | S4 | 233 | 112,017 | 219 |
| *Houstonia serpyllifolia* | S3 | S3 | S3 | S3 | S3 | S4 | S3 | S3 | 85 | 38,075 | 71 |
| *Mitchella repens* | S5 | S5 | S4 | S4 | S4 | S5 | S4 | S5 | 393 | 116,301 | 300 |
| *Oldenlandia boscii* |  | S2 |  |  |  |  |  |  |  |  |  |
| *Oldenlandia uniflora* | S2 | S1 |  |  |  |  |  |  |  |  |  |
| *Spermacoce glabra* |  | S3 | S1 | S1 | S1 | S1 | S1 | S1 | 5 | 446 | 5 |
| **RUTACEAE** |  |  |  |  |  |  |  |  |  |  |  |
| *Ptelea trifoliata* |  | S5 | S3 | S3 | S3 | S3 | S3 | S3 | 26 | 31,493 | 24 |
| *Zanthoxylum americanum* | S1 | S2 |  |  | S2 | S2 | S2 | S3 | 14 | 15,714 | 13 |
| **SALICACEAE** |  |  |  |  |  |  |  |  |  |  |  |
| *Populus deltoides* | S4 | S5 | S3 | S3 | S3 | S3 | S3 | S3 | 68 | 116,873 | 69 |
| *Populus grandidentata* |  | S4 | S2 | S3 | S2 | S3 | S2 | S3 | 9 | 38,478 | 9 |
| *Populus heterophylla* | S4 | S3 | S1 | S1 | S1 | S1 | S1 | S1 | 3 | 1,025 | 3 |
| *Salix caroliniana* | S5 | S5 | S3 | S3 | S3 | S3 | S3 | S3 | 24 | 78,018 | 24 |
| *Salix eriocephala* | S4 | S3 | S1 | S2 | S1 | S2 | S1 | S2 | 3 | 7,320 | 3 |
| *Salix humilis* |  | S4 | S3 | S3 | S3 | S3 | S3 | S3 | 41 | 61,367 | 40 |
| *Salix interior* |  | S3 | S1 | S2 | S1 | S2 | S1 | S2 | 3 | 12,340 | 3 |
| *Salix nigra* | S5 | S5 | S3 | S3 | S3 | S4 | S3 | S3 | 80 | 121,252 | 76 |
| *Salix sericea* | S5 | S4 | S2 | S3 | S2 | S3 | S2 | S3 | 12 | 39,454 | 12 |
| **SANTALACEAE** |  |  |  |  |  |  |  |  |  |  |  |
| *Buckleya distichophylla* | S2 | S2 | S2 | S2 | S2 | S2 | S2 | S2 | 15 | 1,667 | 14 |
| *Comandra umbellata* | S3 | S4 | S3 | S3 | S3 | S3 | S3 | S3 | 30 | 62,687 | 29 |
| *Nestronia umbellula* |  | S2 |  |  | S2 | S2 | S2 | S2 | 6 | 4,725 | 6 |
| *Phoradendron leucarpum* | S5 | S4 | S3 | S3 | S3 | S4 | S3 | S3 | 81 | 115,560 | 79 |
| *Pyrularia pubera* | S5 | S4 | S4 | S4 | S4 | S4 | S4 | S4 | 130 | 42,045 | 114 |
| **SAPINDACEAE** |  |  |  |  |  |  |  |  |  |  |  |
| *Acer negundo* | S5 | S5 | S4 | S5 | S4 | S5 | S4 | S5 | 577 | 130,647 | 525 |
| *Acer pensylvanicum* | S3 | S4 | S4 | S4 | S4 | S4 | S4 | S4 | 231 | 46,406 | 185 |
| *Acer rubrum* | S5 | S5 | S4 | S5 | S4 | S5 | S4 | S5 | 460 | 129,921 | 435 |
| *Acer saccharinum* |  | S5 | S4 | S4 | S4 | S4 | S4 | S4 | 160 | 116,361 | 156 |
| *Acer saccharum* | S5 | S5 | S4 | S4 | S4 | S4 | S4 | S4 | 223 | 113,405 | 204 |
| *Acer spicatum* |  | S3 | S3 | S3 | S2 | S3 | S3 | S3 | 29 | 4,142 | 27 |
| *Aesculus flava* |  | S3 | S3 | S4 | S3 | S4 | S3 | S4 | 84 | 87,730 | 83 |
| *Aesculus glabra* | S2 | S5 | S3 | S3 | S3 | S3 | S3 | S3 | 25 | 56,155 | 25 |
| *Aesculus pavia* |  | S5 | S4 | S4 | S4 | S4 | S4 | S4 | 173 | 110,129 | 146 |
| *Aesculus sylvatica* | S5 | S4 | S2 | S2 | S2 | S2 | S2 | S2 | 8 | 8,744 | 7 |
| **SAPOTACEAE** |  |  |  |  |  |  |  |  |  |  |  |
| *Sideroxylon lycioides* | S3 | S4 | S3 | S3 | S3 | S3 | S3 | S3 | 37 | 31,760 | 34 |
| **SAXIFRAGACEAE** |  |  |  |  |  |  |  |  |  |  |  |
| *Astilbe biternata* |  | S4 | S3 | S3 | S3 | S3 | S3 | S3 | 27 | 33,979 | 25 |
| *Boykinia aconitifolia* |  | S3 | S1 | S2 | S1 | S2 | S1 | S2 | 4 | 14,707 | 4 |
| *Chrysosplenium americanum* |  | S3 | S2 | S2 | S2 | S2 | S2 | S2 | 19 | 3,758 | 17 |
| *Heuchera americana* | S5 | S5 | S3 | S4 | S3 | S4 | S3 | S4 | 92 | 91,414 | 88 |
| *Heuchera hirsuticaulis* |  | S1 | S1 | S1 | S1 | S1 | S1 | S1 | 1 | <1 | 1 |
| *Heuchera longiflora* |  | S3 | S2 | S2 | S2 | S2 | S2 | S2 | 6 | 1,425 | 6 |
| *Heuchera macrorhiza* |  | S2 | S1 | S1 | S1 | S1 | S1 | S1 | 1 | <1 | 1 |
| *Heuchera missouriensis* |  | S1 | S1 | S1 | S1 | S1 | S1 | S1 | 5 | 654 | 5 |
| *Heuchera parviflora* | S3 | S3 | S3 | S3 | S3 | S3 | S3 | S3 | 37 | 20,159 | 33 |
| *Heuchera villosa* |  | S5 | S3 | S3 | S3 | S4 | S3 | S3 | 85 | 79,539 | 78 |
| *Micranthes careyana* | S1 | S3 | S3 | S3 | S3 | S3 | S3 | S3 | 27 | 23,528 | 27 |
| *Micranthes caroliniana* | S3 | S1 |  |  | S1 | S1 | S2 | S2 | 5 | 1,373 | 5 |
| *Micranthes micranthidifolia* | S3 | S2 | S3 | S3 | S3 | S4 | S3 | S3 | 72 | 22,540 | 57 |
| *Micranthes pensylvanica* |  | S1 |  |  | S1 | S1 | S1 | S1 | 1 | <1 | 1 |
| *Micranthes petiolaris* |  | S2 | S3 | S3 | S3 | S3 | S3 | S3 | 31 | 29,486 | 27 |
| *Micranthes virginiensis* | S5 | S4 | S4 | S4 | S4 | S4 | S4 | S4 | 149 | 92,600 | 135 |
| *Mitella diphylla* |  | S5 | S4 | S4 | S3 | S4 | S4 | S4 | 126 | 73,793 | 104 |
| *Sullivantia sullivantii* |  | S1 |  |  | S1 | S1 | S1 | S1 | 2 | 2 | 2 |
| *Tiarella cordifolia* |  | S5 | S3 | S3 | S3 | S3 | S3 | S3 | 33 | 50,896 | 33 |
| **SCROPHULARIACEAE** |  |  |  |  |  |  |  |  |  |  |  |
| *Scrophularia marilandica* |  | S5 | S3 | S3 | S3 | S3 | S3 | S3 | 32 | 118,073 | 31 |
| **SOLANACEAE** |  |  |  |  |  |  |  |  |  |  |  |
| *Physalis angulata* | S4 | S4 | S3 | S3 | S3 | S3 | S3 | S3 | 59 | 102,409 | 58 |
| *Physalis cordata* |  | S1 | S1 | S1 | S1 | S1 | S1 | S1 | 1 | <1 | 1 |
| *Physalis grisea* |  | S3 | S1 | S2 | S1 | S2 | S1 | S2 | 3 | 12,027 | 3 |
| *Physalis heterophylla* | S5 | S5 | S3 | S3 | S3 | S3 | S3 | S3 | 41 | 100,918 | 41 |
| *Physalis longifolia* |  | S4 | S3 | S3 | S3 | S3 | S3 | S3 | 65 | 116,012 | 63 |
| *Physalis pubescens* | S4 | S4 | S2 | S3 | S2 | S3 | S2 | S3 | 9 | 42,432 | 9 |
| *Physalis virginiana* | S4 | S4 | S2 | S3 | S2 | S3 | S2 | S3 | 14 | 43,469 | 14 |
| *Solanum carolinense* | S5 | S5 | S4 | S5 | S4 | S5 | S4 | S5 | 669 | 132,877 | 595 |
| *Solanum elaeagnifolium* |  | S2 |  |  |  |  |  |  |  |  |  |
| *Solanum emulans* |  | S3 | S3 | S4 | S3 | S4 | S3 | S4 | 87 | 111,038 | 83 |
| **STAPHYLEACEAE** |  |  |  |  |  |  |  |  |  |  |  |
| *Staphylea trifolia* | S5 | S5 | S4 | S4 | S4 | S4 | S4 | S4 | 133 | 111,982 | 123 |
| **STYRACACEAE** |  |  |  |  |  |  |  |  |  |  |  |
| *Halesia carolina* |  | S4 | S3 | S3 | S3 | S3 | S3 | S3 | 27 | 46,469 | 26 |
| *Styrax americanus* | S3 | S3 | S1 | S1 | S1 | S1 | S1 | S1 | 2 | 64 | 2 |
| *Styrax grandifolius* |  | S4 | S2 | S3 | S3 | S3 | S2 | S3 | 20 | 22,543 | 20 |
| **SYMPLOCACEAE** |  |  |  |  |  |  |  |  |  |  |  |
| *Symplocos tinctoria* | S5 | S2 |  |  | S3 | S3 | S3 | S3 | 20 | 29,541 | 21 |
| **TETRACHONDRACEAE** |  |  |  |  |  |  |  |  |  |  |  |
| *Polypremum procumbens* | S5 | S4 | S2 | S3 | S2 | S3 | S2 | S3 | 10 | 30,281 | 10 |
| **THEACEAE** |  |  |  |  |  |  |  |  |  |  |  |
| *Stewartia ovata* | S3 | S4 | S3 | S3 | S3 | S3 | S3 | S3 | 68 | 24,305 | 58 |
| **THEOPHRASTACEAE** |  |  |  |  |  |  |  |  |  |  |  |
| *Samolus parviflorus* | S4 | S5 | S3 | S3 | S3 | S3 | S3 | S3 | 51 | 122,392 | 48 |
| **THYMELAEACEAE** |  |  |  |  |  |  |  |  |  |  |  |
| *Dirca palustris* | S4 | S5 | S3 | S3 | S3 | S3 | S3 | S3 | 55 | 70,514 | 52 |
| **ULMACEAE** |  |  |  |  |  |  |  |  |  |  |  |
| *Planera aquatica* |  | S4 | S2 | S3 | S2 | S2 | S2 | S3 | 17 | 22,298 | 17 |
| *Ulmus alata* | S5 | S5 | S4 | S4 | S4 | S4 | S4 | S4 | 180 | 120,087 | 166 |
| *Ulmus americana* | S5 | S5 | S3 | S3 | S3 | S3 | S3 | S3 | 72 | 118,526 | 69 |
| *Ulmus crassifolia* |  | S1 |  |  | S2 | S2 | S2 | S2 | 8 | 2,043 | 7 |
| *Ulmus rubra* | S5 | S5 | S3 | S3 | S3 | S3 | S3 | S3 | 53 | 109,823 | 54 |
| *Ulmus serotina* | S3 | S4 | S3 | S3 | S3 | S3 | S3 | S3 | 22 | 22,741 | 21 |
| *Ulmus thomasii* | S2 | S4 | S1 | S1 | S1 | S1 | S1 | S1 | 3 | 351 | 3 |
| **URTICACEAE** |  |  |  |  |  |  |  |  |  |  |  |
| *Boehmeria cylindrica* | S5 | S5 | S3 | S3 | S3 | S3 | S3 | S3 | 65 | 118,911 | 63 |
| *Laportea canadensis* | S5 | S5 | S3 | S4 | S3 | S4 | S3 | S4 | 114 | 124,513 | 107 |
| *Parietaria pensylvanica* |  | S5 | S2 | S3 | S2 | S3 | S2 | S3 | 15 | 69,620 | 15 |
| *Pilea pumila* | S5 | S5 | S3 | S3 | S3 | S3 | S3 | S3 | 77 | 105,384 | 78 |
| *Urtica chamaedryoides* | S4 | S4 | S3 | S3 | S3 | S3 | S3 | S3 | 29 | 44,061 | 27 |
| *Urtica gracilis* |  | S1 |  |  |  |  |  |  |  |  |  |
| **VALERIANACEAE** |  |  |  |  |  |  |  |  |  |  |  |
| *Valeriana pauciflora* | S1 | S4 | S3 | S3 | S3 | S3 | S3 | S3 | 24 | 24,541 | 22 |
| *Valerianella radiata* | S5 | S5 | S3 | S4 | S3 | S4 | S3 | S4 | 107 | 120,896 | 103 |
| *Valerianella umbilicata* |  | S4 | S2 | S3 | S2 | S3 | S2 | S3 | 16 | 29,539 | 16 |
| **VERBENACEAE** |  |  |  |  |  |  |  |  |  |  |  |
| *Glandularia canadensis* | S1 | S3 | S2 | S2 | S2 | S2 | S2 | S2 | 7 | 1,825 | 7 |
| *Phyla lanceolata* |  | S4 | S3 | S3 | S3 | S3 | S3 | S3 | 75 | 112,797 | 74 |
| *Verbena bracteata* |  | S3 | S1 | S1 | S1 | S1 | S1 | S1 | 2 | 33 | 2 |
| *Verbena hastata* |  | S4 | S2 | S3 | S2 | S3 | S2 | S3 | 16 | 97,077 | 16 |
| *Verbena simplex* | S5 | S5 | S4 | S4 | S4 | S4 | S4 | S4 | 154 | 79,787 | 150 |
| *Verbena stricta* |  | S3 | S1 | S1 | S1 | S1 | S1 | S1 | 2 | <1 | 2 |
| *Verbena urticifolia* | S5 | S5 | S4 | S4 | S4 | S4 | S4 | S4 | 133 | 120,243 | 132 |
| **VIBURNACEAE** |  |  |  |  |  |  |  |  |  |  |  |
| *Sambucus canadensis* | S5 | S5 | S4 | S4 | S4 | S4 | S4 | S4 | 209 | 128,778 | 199 |
| *Sambucus racemosa* |  | S2 | S3 | S3 | S3 | S3 | S3 | S3 | 41 | 32,691 | 40 |
| *Viburnum acerifolium* | S5 | S5 | S4 | S4 | S4 | S4 | S4 | S4 | 255 | 73,404 | 217 |
| *Viburnum carolinianum* |  | S2 | S2 | S2 | S2 | S2 | S2 | S2 | 13 | 1,883 | 13 |
| *Viburnum cassinoides* | S4 | S4 | S3 | S3 | S3 | S3 | S3 | S3 | 23 | 33,496 | 22 |
| *Viburnum lantanoides* | S4 | S2 | S3 | S3 | S3 | S3 | S3 | S3 | 70 | 10,523 | 56 |
| *Viburnum molle* |  | S1 |  |  | S1 | S1 | S1 | S1 | 1 | <1 | 1 |
| *Viburnum nudum* | S5 | S4 | S3 | S3 | S3 | S3 | S2 | S3 | 21 | 27,824 | 21 |
| *Viburnum prunifolium* | S5 | S5 | S3 | S3 | S3 | S3 | S3 | S3 | 24 | 65,495 | 24 |
| *Viburnum rafinesqueanum* |  | S1 |  |  | S1 | S1 | S1 | S1 | 1 | <1 | 1 |
| *Viburnum recognitum* |  | S1 | S1 | S1 | S1 | S1 | S1 | S1 | 2 | 28 | 2 |
| *Viburnum rufidulum* | S5 | S5 | S3 | S4 | S3 | S4 | S4 | S4 | 117 | 102,507 | 112 |
| **VIOLACEAE** |  |  |  |  |  |  |  |  |  |  |  |
| *Cubelium concolor* |  | S3 | S3 | S3 | S3 | S3 | S3 | S3 | 64 | 82,381 | 63 |
| *Viola affinis* | S4 | S4 | S1 | S2 | S1 | S2 | S1 | S2 | 3 | 9,616 | 3 |
| *Viola bicolor* |  | S5 | S4 | S4 | S4 | S4 | S4 | S4 | 270 | 124,756 | 258 |
| *Viola blanda* | S4 | S5 | S3 | S3 | S3 | S3 | S3 | S3 | 69 | 58,753 | 61 |
| *Viola canadensis* |  | S5 | S3 | S4 | S3 | S4 | S3 | S4 | 105 | 60,860 | 90 |
| *Viola cucullata* |  | S5 | S3 | S3 | S3 | S3 | S3 | S3 | 27 | 84,689 | 27 |
| *Viola egglestonii* |  | S3 | S3 | S3 | S3 | S3 | S3 | S3 | 31 | 20,337 | 28 |
| *Viola hastata* | S5 | S4 | S4 | S4 | S4 | S4 | S4 | S4 | 267 | 53,961 | 233 |
| *Viola hirsutula* | S4 | S5 | S3 | S3 | S3 | S3 | S3 | S3 | 43 | 72,432 | 43 |
| *Viola labradorica* |  | S3 | S3 | S3 | S3 | S3 | S3 | S3 | 24 | 28,862 | 23 |
| *Viola lanceolata* | S4 | S3 | S2 | S3 | S2 | S3 | S2 | S3 | 17 | 30,823 | 17 |
| *Viola minuscula* | S3 | S2 | S1 | S2 | S2 | S2 | S2 | S2 | 5 | 7,395 | 5 |
| *Viola missouriensis* |  | S3 | S1 | S2 | S1 | S2 | S1 | S2 | 4 | 6,108 | 4 |
| *Viola palmata* |  | S5 | S3 | S3 | S3 | S3 | S3 | S3 | 77 | 109,187 | 74 |
| *Viola pedata* |  | S5 | S4 | S4 | S4 | S4 | S4 | S4 | 145 | 97,767 | 137 |
| *Viola primulifolia* |  | S4 | S3 | S3 | S3 | S3 | S3 | S3 | 33 | 44,174 | 32 |
| *Viola pubescens* | S5 | S5 | S3 | S3 | S3 | S4 | S3 | S3 | 33 | 103,646 | 31 |
| *Viola rostrata* | S5 | S5 | S4 | S4 | S4 | S4 | S4 | S4 | 142 | 62,232 | 127 |
| *Viola rotundifolia* |  | S4 | S3 | S3 | S3 | S3 | S3 | S3 | 29 | 26,798 | 24 |
| *Viola sagittata* |  | S5 | S3 | S3 | S3 | S3 | S3 | S3 | 26 | 61,145 | 24 |
| *Viola septemloba* |  | S2 | S1 | S1 | S1 | S1 | S1 | S1 | 1 | <1 | 1 |
| *Viola sororia* | S5 | S5 | S4 | S5 | S4 | S5 | S4 | S5 | 401 | 123,332 | 363 |
| *Viola striata* | S4 | S5 | S3 | S3 | S3 | S3 | S3 | S3 | 74 | 80,905 | 73 |
| *Viola subsinuata* |  | S4 | S2 | S3 | S2 | S3 | S2 | S3 | 9 | 47,696 | 9 |
| *Viola tripartita* |  | S4 | S2 | S2 | S2 | S2 | S2 | S2 | 10 | 4,680 | 9 |
| *Viola walteri* |  | S3 | S1 | S1 | S2 | S1 | S2 | S1 | 5 | 2,701 | 5 |
| **VITACEAE** |  |  |  |  |  |  |  |  |  |  |  |
| *Ampelopsis cordata* | S4 | S5 | S3 | S3 | S3 | S3 | S3 | S3 | 73 | 104,740 | 72 |
| *Muscadinia rotundifolia* | S5 | S3 | S2 | S3 | S2 | S2 | S2 | S3 | 10 | 23,402 | 9 |
| *Nekemias arborea* |  | S3 | S3 | S3 | S3 | S3 | S3 | S3 | 72 | 89,824 | 69 |
| *Parthenocissus quinquefolia* | S5 | S5 | S4 | S5 | S4 | S5 | S4 | S5 | 974 | 133,767 | 861 |
| *Vitis aestivalis* | S5 | S5 | S3 | S3 | S3 | S3 | S3 | S3 | 41 | 92,167 | 40 |
| *Vitis baileyana* | S4 | S3 | S2 | S2 | S2 | S2 | S2 | S2 | 7 | 6,509 | 7 |
| *Vitis cinerea* |  | S4 | S3 | S3 | S3 | S3 | S3 | S3 | 24 | 99,132 | 24 |
| *Vitis labrusca* |  | S4 | S2 | S3 | S2 | S3 | S2 | S3 | 18 | 60,469 | 18 |
| *Vitis palmata* |  | S3 | S1 | S1 | S1 | S1 | S1 | S1 | 2 | 144 | 2 |
| *Vitis riparia* | S5 | S3 | S2 | S3 | S2 | S3 | S2 | S3 | 8 | 25,311 | 8 |
| *Vitis rupestris* | S1 | S2 |  |  | S2 | S2 | S2 | S2 | 8 | 9,516 | 8 |
| *Vitis vulpina* | S5 | S5 | S3 | S3 | S3 | S3 | S3 | S3 | 50 | 90,890 | 51 |

Note: S-ranks = Subnational Conservation Status Ranks, FME = Feature Manipulation Engine, AOO = Area of Occupancy, RARECAT = Rapid Analysis of Rarity and Endangerment Conservation Assessment Tool.

^a^ Taxonomy follow the second edition of the *Guide to the Vascular Plants of Tennessee* (Shaw et al., in press). Authorities were excluded due to space limitations.

^b^ Scientific names highlighted light gray indicate species ranked using rare species data from the Tennessee Department of Environment and Conservation Division of Natural Areas.

^c^ The area of occupancy, range extent, and number of occurrences criteria presented here were obtained from the FME ranking method. Criteria for species that did not have a rank assigned using FME were obtained from the RARECAT ranking method.
