# Supplementary material for: Semi‐automated assessment for NatureServe subnational conservation status ranks for state floras
Source: Appl Plant Sci. 2026 Mar 19;14(2):e70046. doi: 10.1002/aps3.70046 (PMC13103536; doi:10.1002/aps3.70046)
Supplement: Supplementary file 3 — Appendix S3. Manual for calculating rarity criteria and provisionary S‐ranks using a series of geoprocessing tools in ArcGIS Pro (without a Toolbox). [file APS3-14-e70046-s001.docx]

Appendix S3. Manual for calculating rarity criteria and provisionary S-ranks using a series of geoprocessing tools in ArcGIS Pro (without a Toolbox).

Using ArcGIS Pro to Calculate Rarity Criteria for NatureServe Conservation Status Assessments

By: Julia Prins

The University of Tennessee at Chattanooga

May 2025

This document describes the steps to be taken when using ArcGIS Pro to calculate rarity criteria for NatureServe conservation status assessments for species. More specifically, it describes how to gather species occurrence data, clip data to a boundary, calculate range extent, calculate number of occurrences, and calculate area of occupancy. This information can then be used directly to assign conservation status ranks using the NatureServe rank calculator (available at natureserve.org; NatureServe, 2020b[)](https://www.natureserve.org/conservation-tools/conservation-rank-calculator). These processes can be used to calculate criteria for an individual species or as a batch process to calculate criteria for multiple species simultaneously. Additionally, the processes can be applied at all geographic scales (global, national, and subnational) provided that the appropriate boundary is selected.

To follow these instructions, a basic understanding of ArcGIS software is required. Developed in 2025, these guidelines use Esri ArcGIS Pro Version 3.0.0. Please note that as applications are continuously evolving, the applicability of these instructions may change slightly over time. Questions and further information related to the instructions below can be directed to Julia Prins (juliaprins19@gmail.com).

I would like to thank Dillon Blankenship (Tennessee Natural Heritage Program) and Nyssa Hunt (The University of Tennessee at Chattanooga - Interdisciplinary Geospatial Technology Lab) for their GIS expertise and assistance with this manual.

**Gathering Species Occurrence Data**

Species occurrence records containing geographic coordinates are necessary to calculate the rarity criteria of range extent, number of occurrences, and area of occupancy. These records can be obtained from internal sources such as Biotics or external sources such as GBIF and the Symbiota portals.

Obtaining occurrences records from GBIF (GBIF.org, 2024):

1. Visit gbif.org and select occurrences.
2. Select the All filters tab.
3. Under the Taxon category, apply a filter to only include records of the taxon of interest.
4. Under the Location category apply a filter to only include records from your area of interest by selecting the desired Country or area and State province. Under location select including coordinates.
5. Under the Event category apply a filter to exclude historic records using the desired cutoff year.
6. In the display window select Download and choose either the Simple or Darwin Core download option.

Obtaining occurrences records from a Symbiota portal (SERNEC Data Portal, 2024):

Note: the instructions below are for the Symbiota portal of the Southeast Regional Network of Expertise and Collections (SERNEC) but similar steps can be taken to obtain occurrence records from the other Symbiota portals (available at symbiota.org).

1. Visit https://sernecportal.org/portal/
2. Under the Specimen Search tab select Search Collections.
3. Under the Taxon category, apply a filter to only include records of the taxon of interest.
4. Under the Locality category, enter the Country or State of interest.
5. Under the Sample Properties category, check the Limit to Specimens with Geocoordinates box.
6. Under the Collecting Event category, enter the desired historic cutoff date.
7. Under Results Display Format, select Table and hit Search.
8. Click the Download Specimen Data button at the top of the page.
9. Select the settings of your choice and hit Download Data.

Prepping Occurrence Data for Upload:

If gathering occurrence data from multiple sources, all occurrences need to be merged into a single spreadsheet and several steps need to be taken to ensure information matches.

1. Ensure the same scientific names are being used across datasets. If any synonyms are being used in a dataset replace the synonym names with the correct scientific name. This can be done by obtaining a synonym list and cross-checking it against the dataset.
2. Ensure the same coordinate system is being used across datasets. Coordinates should be in decimal degrees of latitude and longitude, with latitudes south of the equator represented by negative values and longitudes west of the Prime Meridian also indicated by negative values.
3. Next, the datasets can be combined into a single spreadsheet. One column with scientific name, one column with latitude, and one column with longitude must be present.
4. If ranking at the species level all infraspecific designations should be removed and simplified to species level (e.g. *Asplenium scolopendrium var. americanum* simplified to *Asplenium scolopendrium*). This ensures that all occurrences for a species are being accounted for in its calculations.
5. Lastly, ensure no spaces exist in column names or scientific names. Any spaces should be replaced with an underscore (“_”) using the find and replace tool. The same should be done for any special characters (e.g. the genus *Isoëtes* should be replaced with *Isoetes* so the special character is replaced). This is because ArcGIS Pro can have difficulty reading file names that contain spaces or special characters.

**Clipping the Data to Area of Interest**

When using occurrence records from external sources, such as GBIF and Symbiota portals, it is crucial to verify that all records fall within the area of interest when mapped. Even if data is filtered during the initial collection, inaccuracies in locality information can lead to records being mapped outside the intended area. Therefore, to ensure the accuracy of data, it is important to clip the occurrence records to the boundary of the area of interest as described below.

Step 1: Upload a boundary file.

If you already have a layer defining the boundary of your area of interest add this to your map by navigating to the ribbon at the top of the page and under the Map tab in the Layer group, select Add Data. Select the boundary layer and hit OK.


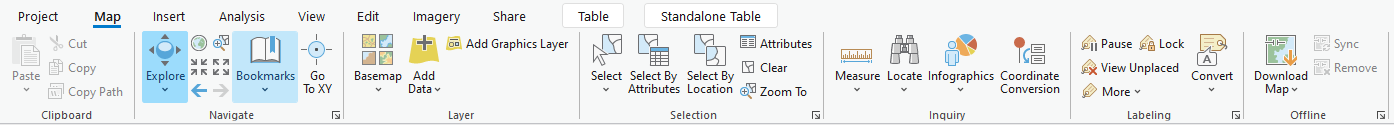


If you do not have a layer defining the boundary of your area of interest, you can use a pre-existing layer from the ArcGIS Online Living Atlas (option 1 below) or define your boundary (option 2 below).

*Option 1: Using a layer from the ArcGIS Online Living Atlas for U.S. States or Canadian Provinces/Territories*

1. In the Catalog pane select the Portal tab. Select Living Atlas.


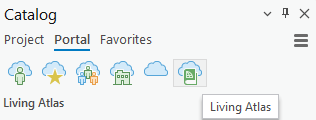


1. Search “USA State Boundaries” or “Provinces and Territories of Canada”. Multiple layers containing the boundaries of your search will appear. Right-click the feature layer of your choice and select Add to Current Map.
2. To only include the boundary of your state or province/territory of interest a definition query can be applied to the layer. In the Contents pane right-click the boundary layer and select Properties. In the Properties window select the Definition Query tab. Select New definition query and add a query where state or province/territory name is equal to state or province/territory of interest. Select Apply and hit OK.


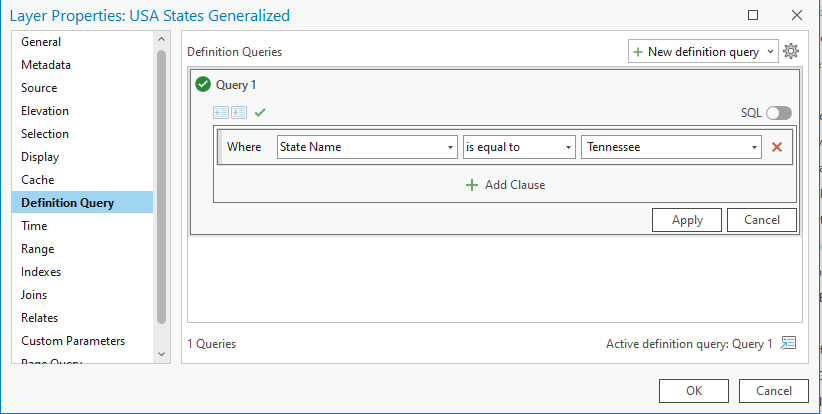


1. In the Contents pane right-click the boundary layer and select Data and Export Features. Name the Output Feature Class as the state or province/territory boundary and select OK. This will add the living atlas layer to the local geodatabase so it can be used for geoprocessing tools.


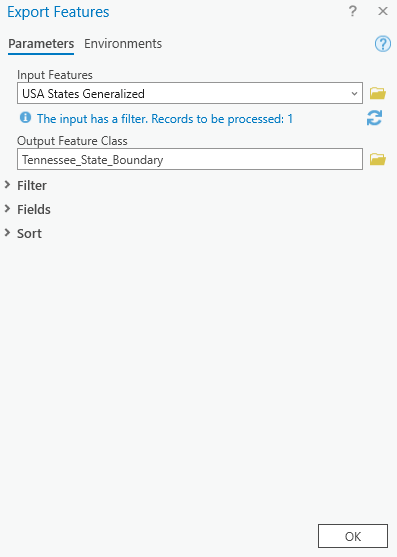


*Option 2: Manually Defining a Boundary*

1. In the Catalog pane, under the Project tab expand Databases. Right-click the current project geodatabase and select New and Feature Class.


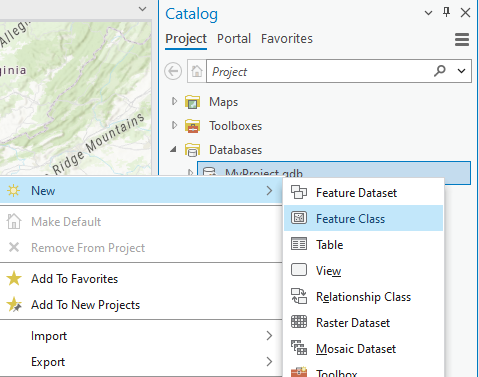


1. Name the Feature Class “AreaBoundary” and ensure Polygon is selected as the Feature Class Type. Select Finish.


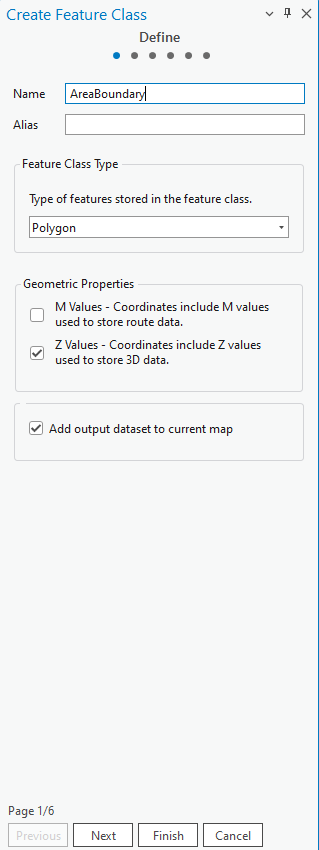


1. Navigate to the ribbon at the top of the page. Under the Edit tab in the Features group select Create.


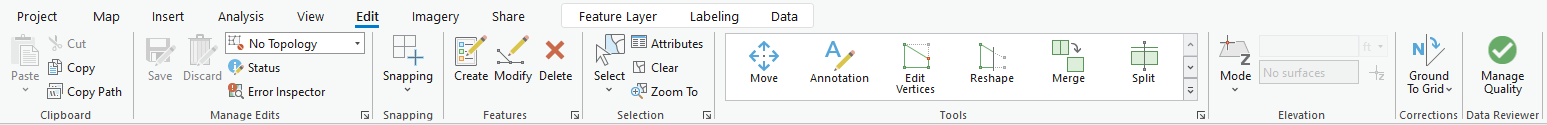


1. In the Create Features pane select the new AreaBoundary layer and use the Polygon tool to draw a boundary for your area of interest on the map. Double-click to finish the polygon.


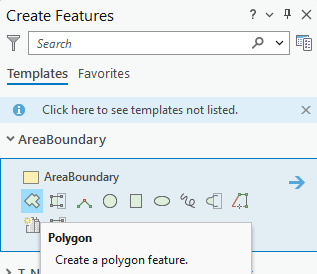


1. Navigate to the ribbon at the top of the page. Under the Edit tab in the Manage Edits group select Save.

Step 2: Upload your occurrence records.

1. Ensure your spreadsheet is downloaded as a CSV file.
2. Navigate to the ribbon at the top of the page. Under the Map tab in the Layer group select Add Data.


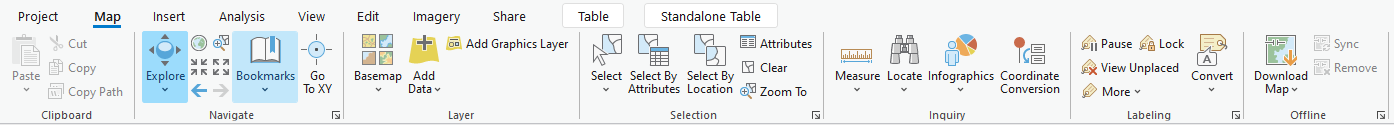


1. Select the file containing your occurrence records and hit OK.
2. In the Contents pane right-click the newly added attribute table containing your occurrence records and select Display XY Data (Note: newer versions of ArcGIS Pro may require you to select Create Points From Table and then XY Table to Point instead).
   1. Ensure your occurrence file is selected as the Input Table.
   2. Name your Output Feature Class as “Occurrence_Records_ALL”.
   3. For the X Field ensure the column containing longitude is selected.
   4. For the Y Field ensure the column containing latitude is selected.
   5. Hit OK.
   6. This will add the occurrence points to your map.


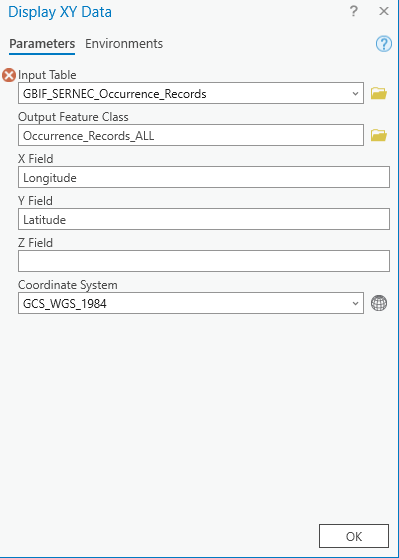


Step 3: Ensure the coordinate system of your dataset is UTM (Universal Transverse Mercator)

1. To check the coordinate system of your dataset, in the Contents pane right-click the layer containing all occurrence records and select Properties. In the Properties window select the Source tab. Expand Spatial Reference and the Geographic Coordinate System will be listed. If the coordinate system is not UTM the occurrence record layer will need to be Projected into a UTM coordinate system using the following steps (Note: the image below shows a coordinate system *not* in UTM).


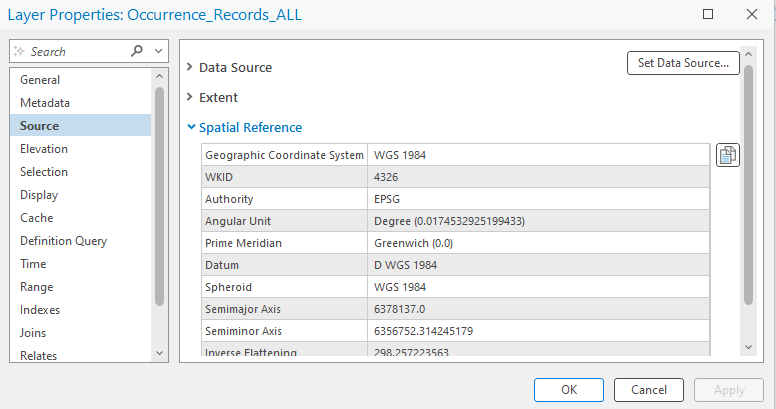


1. Open the geoprocessing pane by navigating to the ribbon at the top of the page. Under the Analysis tab in the Geoprocessing group select Tools.


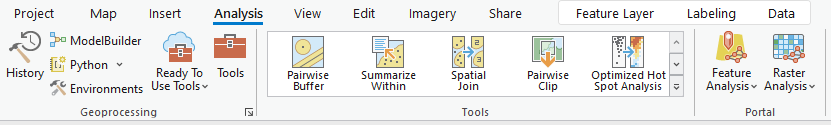


1. In the geoprocessing pane search for and open the *Project* tool.
2. For Input Dataset or Feature Class select the layer containing all occurrence records.
3. Name the Output Dataset or Feature Class “Occurrence_Records_UTM”.
4. Select a UTM Output Coordinate System.
   1. Click the globe icon to select a coordinate system.


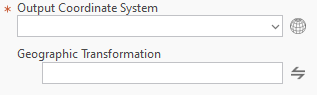


- 1. In the Output Coordinate System window under XY Coordinate Systems Available expand Projected Coordinate System.


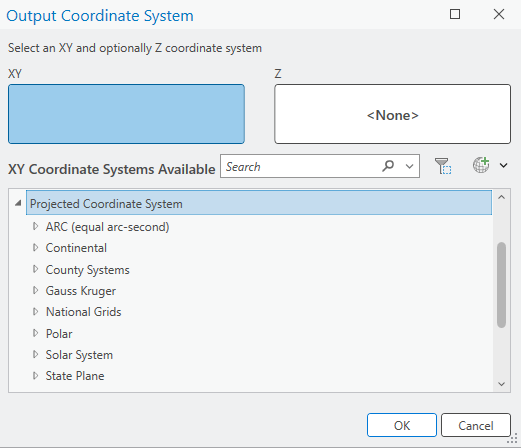


- 1. Scroll down and expand UTM.


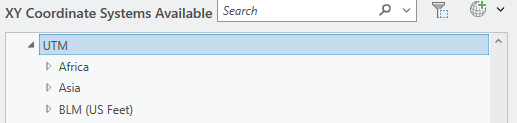


- 1. Scroll down and expand WGS 1984 and expand Northern Hemisphere.


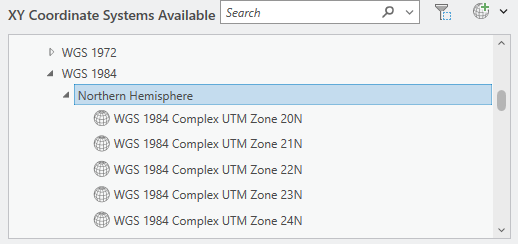


- 1. Find and select the UTM zone located at the center of your area of interest (this information is readily available on the internet).


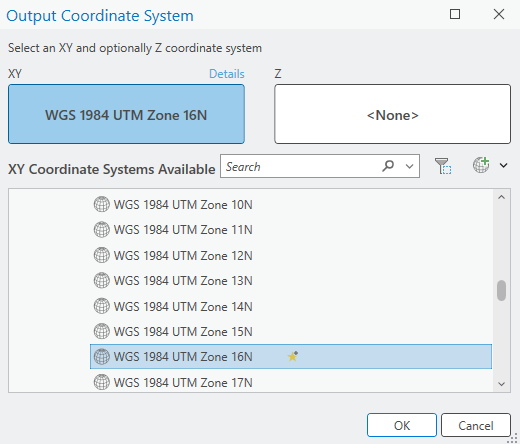


- 1. Click OK.


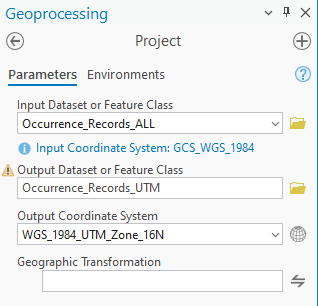


1. Click Run.

Step 4: Use the *Clip Layer* tool to remove all occurrence points outside of the area of interest.

1. In the geoprocessing pane search for and open the *Clip Layer* tool.
   1. For Input Layer select the layer containing all occurrence records in the UTM coordinate system created in step 3.
   2. For Clip Layer select the layer containing the boundary of your area of interest.
   3. Name the Output Feature Class “Occurrence_Records_Clipped”.
   4. Click Run.


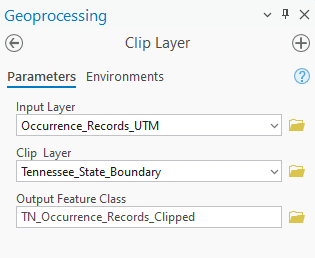


**Creating An Individual Occurrence Layer for Each Species (Only Necessary for Batch Processing)**

If calculating criteria for multiple species you will need to separate your occurrence data into individual layers for each species. This ensures that criteria calculations for each species are based solely on occurrences records of that species. This can be done by creating a ModelBuilder in ArcGIS Pro as outlined below. If calculating criteria for a single species this section can be skipped.

Create a ModelBuilder.

1. Open a ModelBuilder by navigating to the ArcGIS Pro ribbon at the top of the page. Under the Analysis tab in the Geoprocessing group select ModelBuilder. This will open a new blank model page.


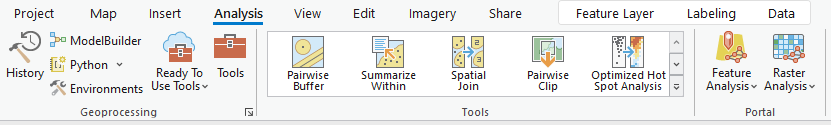


1. Under the ModelBuilder tab in the Insert group select Iterators and choose *Iterate Feature Selection.* This will add the iterator to your model.


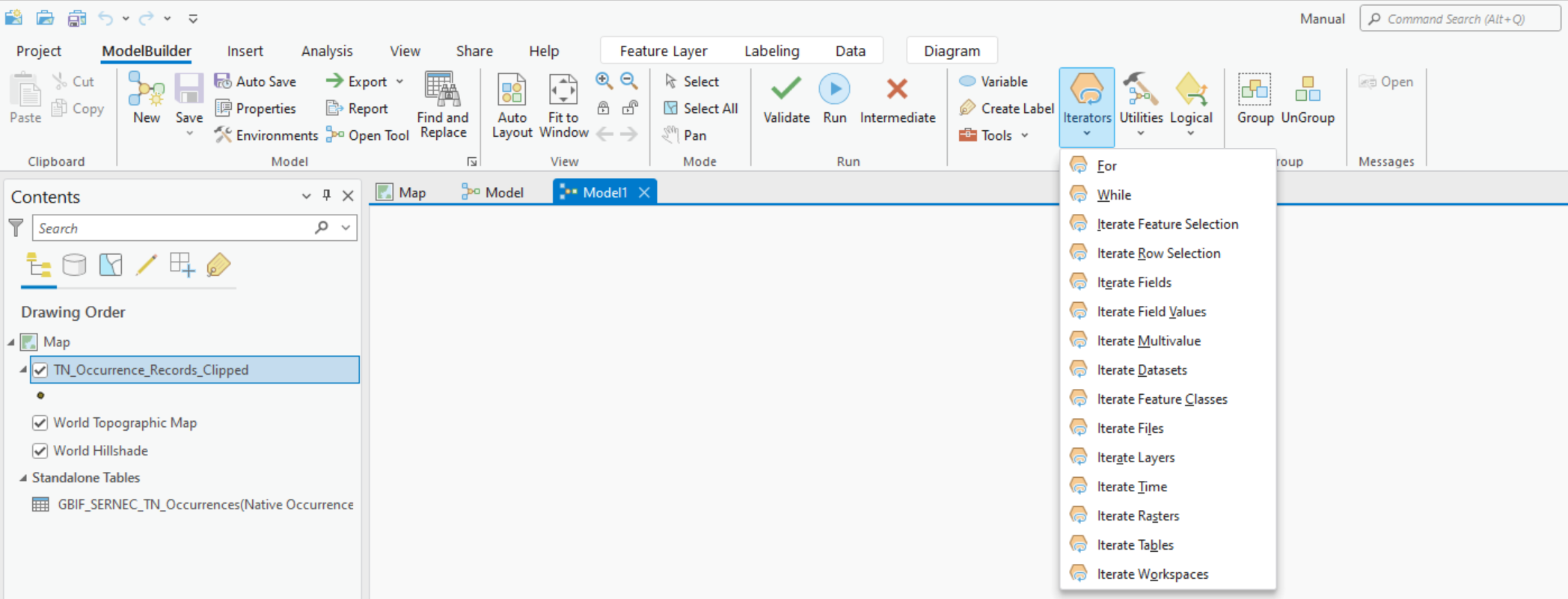


1. In your model open the Iterate Feature Selection element by right-clicking and selecting open.
   1. For In Features select the clipped occurrence layer created in the previous step.
   2. Under Group By Fields from the dropdown select the field that contains the scientific name of your species.
   3. Ensure the Skip Null Values box is checked.
   4. Select OK.


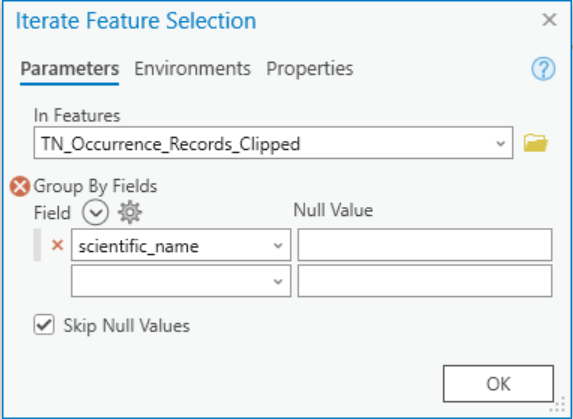


1. Under the ModelBuilder tab in the Insert group select Tools and search for the *Export Features* tool*.* Select this tool and drag it into your model.


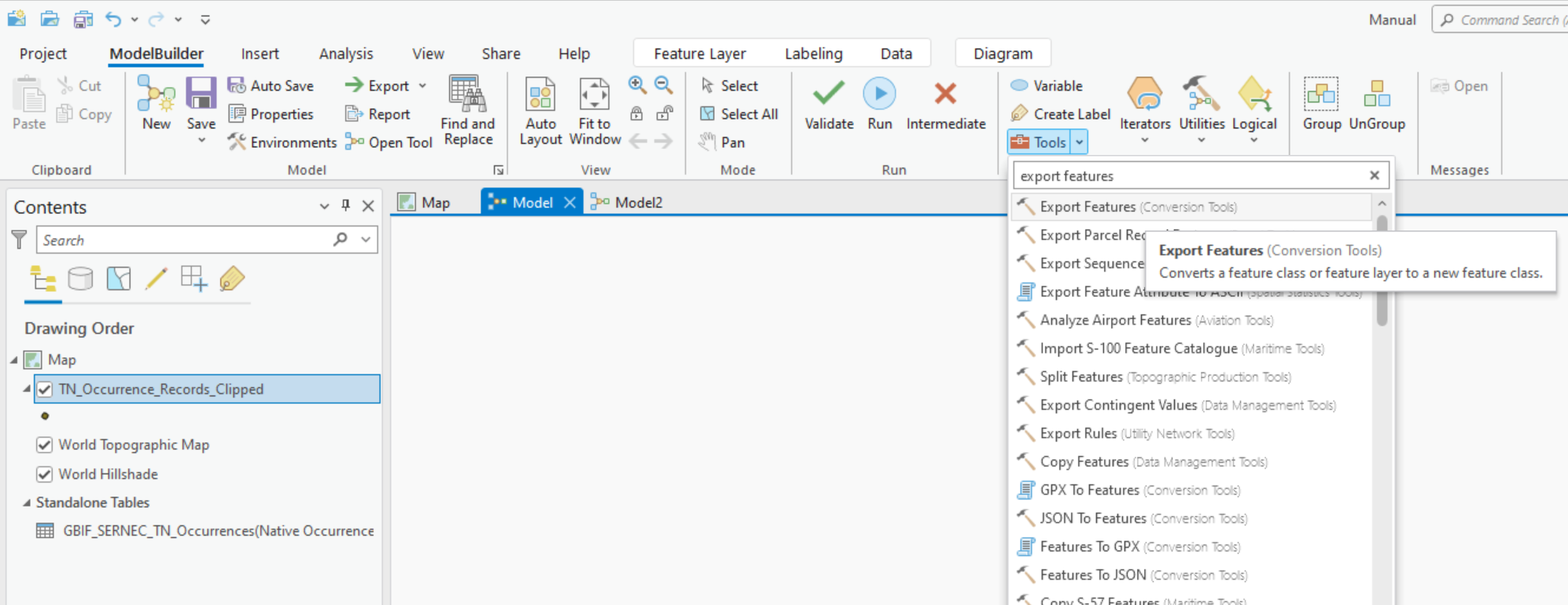


1. In your model drag an arrow from the selected features of your Iterate Feature Selection element to the Export Features element and select Input Features.


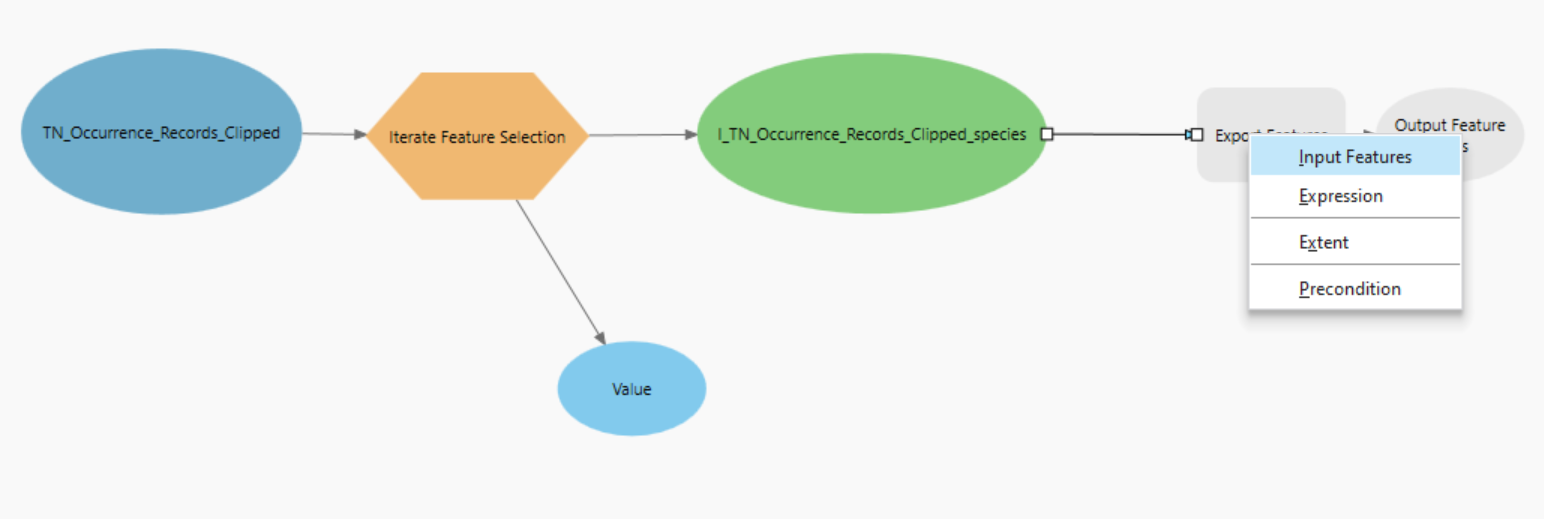


1. Open the Export Features element by right-clicking and selecting open.
   1. For Output Feature Class type “Occurrences_%Value%”. The “%Value%” command tells the model to pull in the name from the selected scientific name field from step 3 when creating and naming each unique layer. This ensures that each new layer will contain its scientific name (e.g. “Occurrences_Acer_rubrum”, “Occurrence_Abies_fraseri”, etc.).
   2. Do not edit Input Features. This was determined when dragging the arrow to connect the Iterate Feature Selection element and the Export Features element.
   3. Select OK.


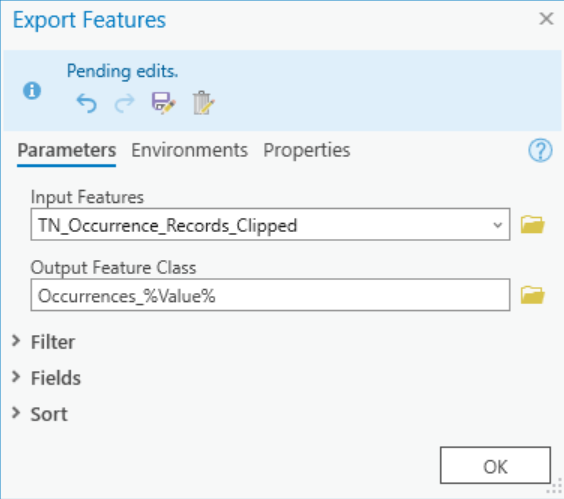

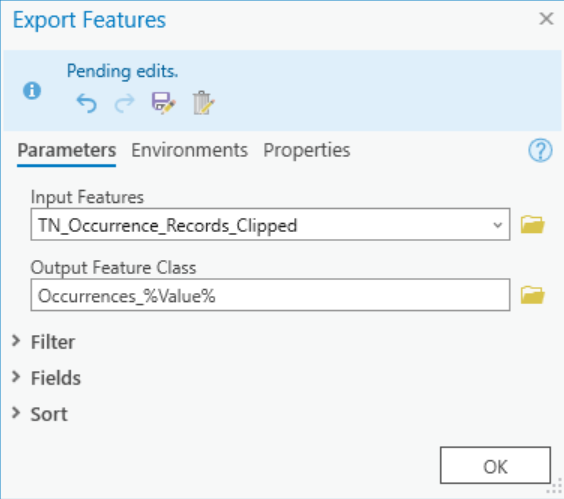


1. Your model should now look something like this.


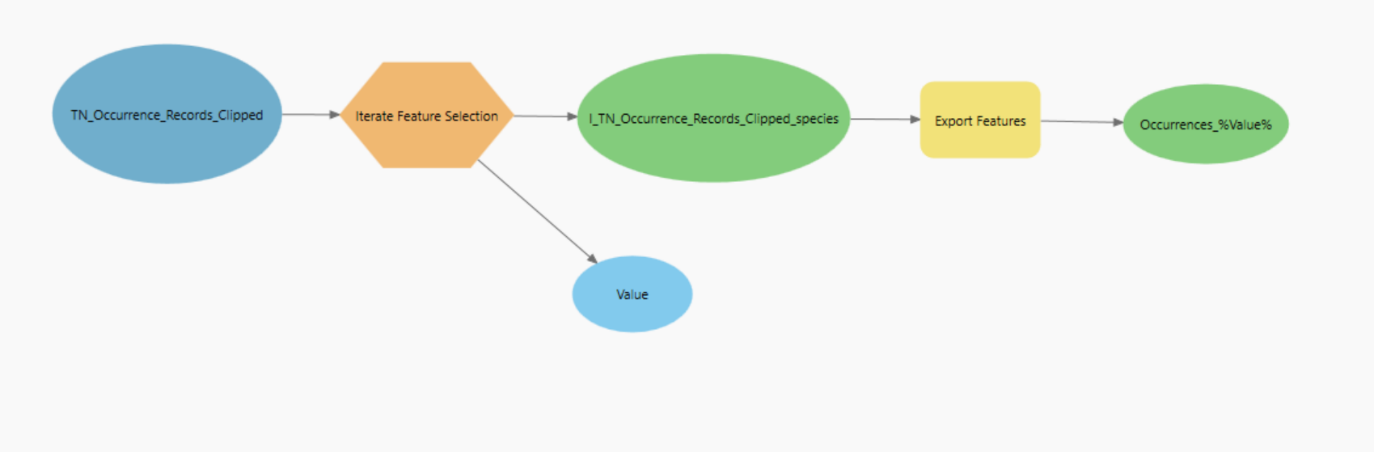


1. Navigate to the ribbon at the top of the page and under the ModelBuilder tab in the Run group select Run. This should start your model, and it will begin creating new occurrence layers for each species. Depending on how many occurrences records are in your file and the processing speed of your device this may take several hours (e.g. when running this model with 200,000 occurrence records this step took over 24 hours).
2. Once the model has finished running, in the Catalog pane, under the Project tab expand Databases and expand your project geodatabase. Select all newly created occurrences layers, right-click, and select Add To Current Map (Note: if the new occurrence layers are not immediately showing up you may need to refresh the geodatabase by right-clicking and selecting refresh).


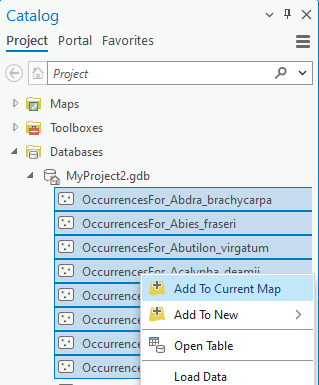


**Calculating Range Extent**

Range extent is defined as the smallest possible boundary that encompasses all known occurrence points of a taxon within the area of interest (Master et al., 2012). The NatureServe rank calculator uses square kilometers as the unit of measurement for this criterion.

Step 1: Use the *Minimum Bounding Geometry* tool to map species range.

1. Open the geoprocessing pane by navigating to the ribbon at the top of the page. Under the Analysis tab in the Geoprocessing group select Tools.


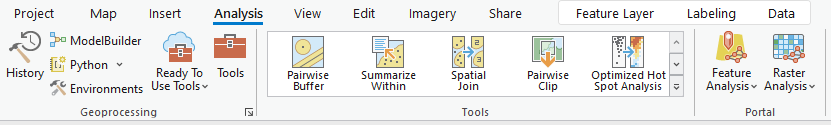


1. In the geoprocessing pane search for the *Minimum Bounding Geometry* tool.
   1. For single species processing: Select and open the tool. For Input Feature select the species occurrence layer.
   2. For batch processing: Right-click the tool and select Batch. Ensure Input Features is selected as the batch parameter and hit Next. Next to Batch Input Features click the down arrow and select all individual species occurrence layers. Click Add.


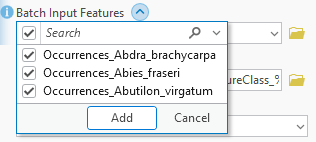


1. Name the output feature class “Range_%Name%”. The “%Name%” command will be replaced by the name of each unique occurrence layer. This ensures that each output feature class contains the species name.
2. For Geometry Type select Convex hull.
3. For Group Option select All.
4. Click Run. This may take several hours depending on the number of layers being run.


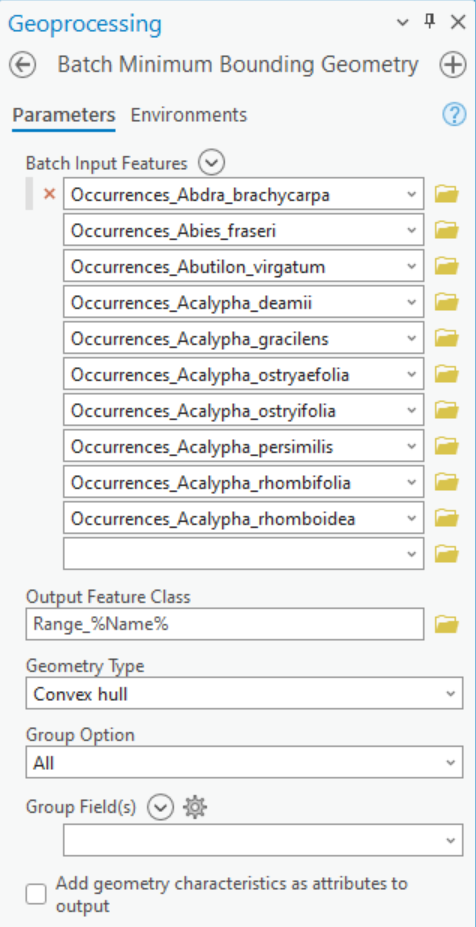


1. For single species processing: open the attribute table of the newly created species range layer and range extent in square meters will be listed under the “Shape_Area” column (Note: this will need to be converted into square kilometers before uploading to the NatureServe rank calculator).
2. For batch processing proceed to step 2 below.

Step 2: Use the *Merge* tool to create a single table listing range extent for all species (only necessary for batch processing).

1. In the geoprocessing pane search for and open the *Merge* tool.
2. Next to Input Datasets click the down arrow and select all individual range layers created in step 1. Click Add.
3. Name your Output Dataset.
4. Ensure the Add source information to output box **is checked**. This ensures that each file name, and therefore species name, is included in the output table along with its range.
5. Click Run.


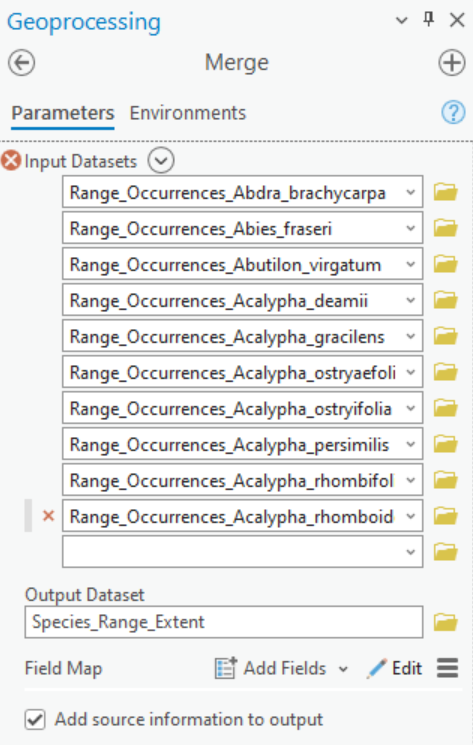


1. Open the attribute table of the new Output Dataset. Range extent in square meters for each species will be listed under the “Shape_Area” column and the input file name which contains species name will be listed under the “MERGE_SRC” column (Note: range will need to be converted into square kilometers before uploading to the NatureServe rank calculator).

**Calculating Number of Occurrences**

Number of occurrences (often referred to as Element Occurrences or EO) is the number of “on-the-ground” locations where a taxon is present (Master et al., 2012). To eliminate the effect of including multiple occurrence records of the same “on-the-ground” location, it is recommended that a minimum of 1 km separation distance be present between occurrences. If records are less than 1 km apart, they should be counted as a single occurrence (NatureServe, 2020a).

Step 1: Use the *Buffer* tool to create a 500 m buffer around each point.

1. Open the geoprocessing pane by navigating to the ribbon at the top of the page. Under the Analysis tab in the Geoprocessing group select Tools.


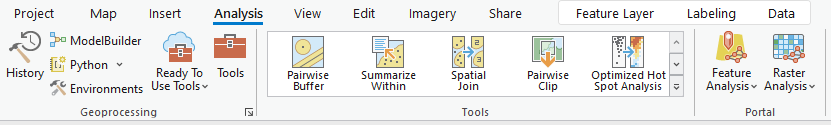


1. In the geoprocessing pane search for the *Buffer* tool.
   1. For single species processing: Select and open the tool. For Input Feature select the species occurrence layer.
   2. For batch processing: Right-click the tool and select Batch. Ensure Input Features is selected as the batch parameter and hit Next. Next to Batch Input Features click the down arrow and select all individual species occurrence layers. Click Add.


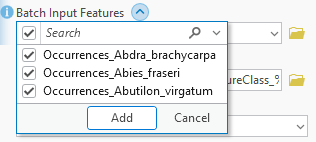


1. Name your output feature class “Buffer_%Name%”. The “%Name%” command will be replaced by the name of each unique occurrence layer. This ensures the output feature class contains the species name.
2. For Distance enter 500 and as the unit select Meters.
3. Ensure the following parameters are selected.
   1. Side Type: Full
   2. End Type: Round
   3. Dissolve Type: No Dissolve
   4. Method: Planar
4. Click Run. This may take several hours depending on the number of occurrence layers being run.


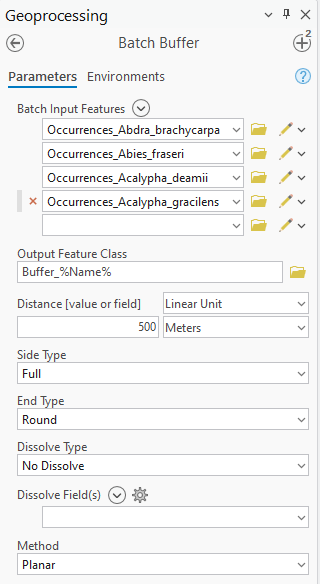


Step 2: Use the *Dissolve Boundaries* tool to merge occurrence points with overlapping boundaries into a single point.

1. In the geoprocessing pane search for the *Dissolve Boundaries* tool.
   1. For single species processing: Select and open the tool. For Input Feature select the buffer layer created in step 1.
   2. For batch processing: Right-click the tool and select Batch. Ensure Input Features is selected as the batch parameter and hit Next. Next to Batch Input Features click the down arrow and select all individual buffer layers created in step 1. Click Add.
2. Name the output feature class “ElementOccurence_%Name%”.
3. Ensure all other parameters are left blank and boxes left unchecked.
4. Click Run. This may take several hours depending on the number of layers being run.


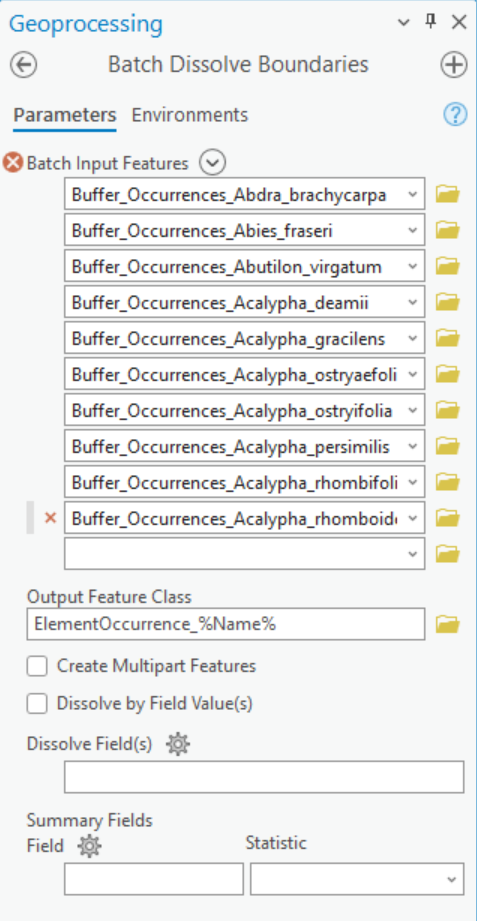


Step 3: Use the *Summary Statistics* tool to count the number of occurrences for each species.

1. In the geoprocessing pane search for the *Summary Statistics* tool.
   1. For single species processing: Select and open the tool. For Input Table select the dissolved buffer layer created in step 2.
   2. For batch processing: Right-click the tool and select Batch. Ensure Input Table is selected as the batch parameter and hit Next. Next to Batch Table Features click the down arrow and select all individual dissolved buffer layers created in step 2. Click Add.
2. Name the output feature class “EO_Count_%Name%”.
3. Under Statistics Fields from the dropdown select the OBJECTID field and as the Statistic Type select Count.
4. Ensure all other parameters are left blank.
5. Click Run. This may take several hours depending on the number of layers being run.


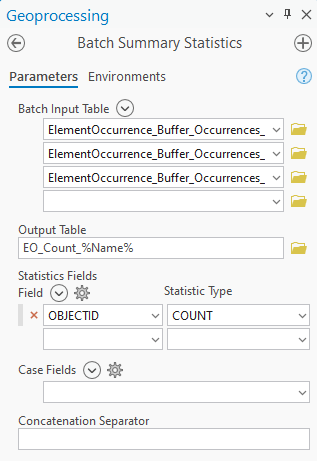


1. For single species processing: open the attribute table of the newly created occurrence count layer and the number of occurrences will be listed under the “FREQUENCY” column.
2. For batch processing proceed to step 4 below.

Step 4: Use the *Merge* tool to create a single table listing the number of occurrences for all species (only necessary for batch processing).

1. In the geoprocessing pane search for and open the *Merge* tool.
2. Next to Input Datasets click the down arrow and select all individual occurrence count layers created in step 3. Click Add.
3. Name the Output Dataset.
4. Ensure the Add source information to output box **is checked**. This ensures that each file name, and therefore species name, is included in the output table along with its number of occurrences.
5. Click Run.


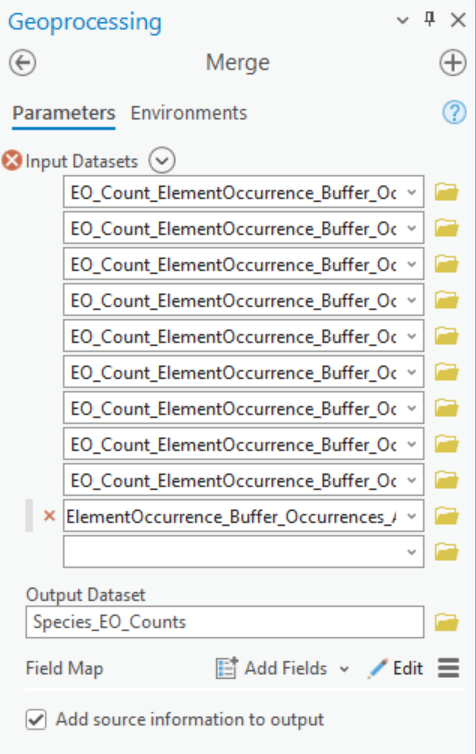


1. Open the new attribute table created. Number of occurrences for each species will be listed under the “FREQUENCY” column and the input file name which contains species name will be listed under the “MERGE_SRC” column.

**Calculating Area of Occupancy**

Area of occupancy (AOO) is the area within its range extent that is occupied by the taxon. It is calculated by counting the number of 2 x 2 km grid cells that contain an occurrence record. This reflects that a taxon may not occupy the entirety of its range extent due to unsuitable or unoccupied habitat (Master et al., 2012).

Step 1: Use the *Generate Tessellation* tool to create a layer of 2 x 2 km grid cells.

1. Open the geoprocessing pane by navigating to the ribbon at the top of the page. Under the Analysis tab in the Geoprocessing group select Tools.


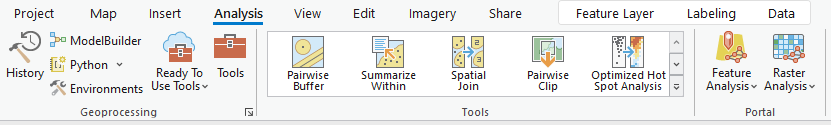


1. In the geoprocessing pane search for and open the *Generate Tessellation* tool.
2. Name the Output Feature Class “AOO_Grid_Cells”.
3. For Extent click the dropdown and select the layer containing the boundary for your area of interest (this was created during step 1 of the Clipping the Data to Area of Interest section of the manual). Extent will auto populate based on this layer.
4. For Shape Type select Square.
5. For Size enter 4 and select Square Kilometers as the unit.
6. Click Run.


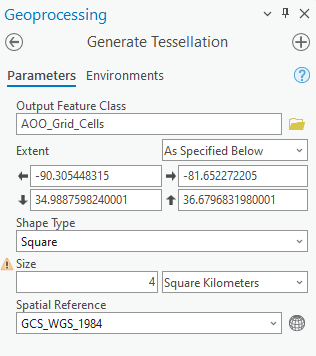


Step 2: Use the *Spatial Join* tool to create a layer of occupied 2 x 2 km grid cells for each species.

1. In the geoprocessing pane search for the *Spatial Join* tool.
   1. For single species processing: Select and open the tool. For Join Feature select the species occurrence layer.
   2. For batch processing: Right-click the tool and select Batch. For the batch parameter choose Join Features and hit Next. Next to Batch Join Features click the down arrow and select all individual species occurrence layers. Click Add.


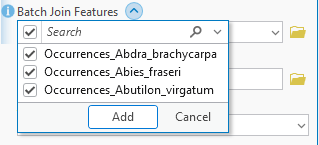


1. For Target Features select the 2 x 2 km grid cells created in step 1.
2. Name your output feature class “SpatialJoin_%Name%”. The “%Name%” command will be replaced by the name of each unique occurrence layer. This ensures the output feature class contains the species name.
3. For Join Operation select Join one to one.
4. Ensure the Keep All Target Features box is **unchecked**.
5. For Match Option select Intersect.
6. Ensure all other parameters are left blank.
7. Click Run. This may take several hours depending on the number of layers being run.


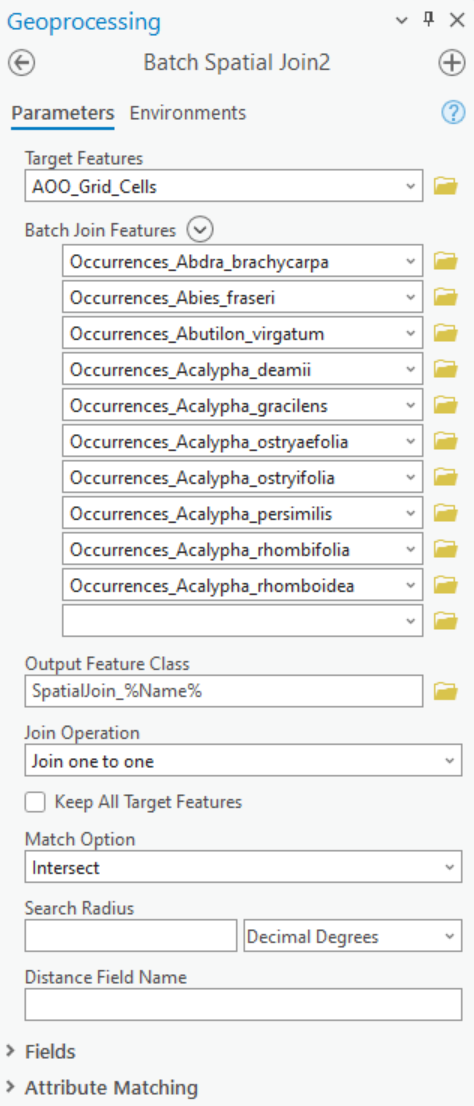


Step 3: Use the *Summary Statistics* tool to count the number of occupied 2 x 2 km grid cells for each species.

1. In the geoprocessing pane search for the *Summary Statistics* tool.
   1. For single species processing: Select and open the tool. For Input Table select the spatial join layer created in step 2.
   2. For batch processing: Right-click the tool and select Batch. Ensure Input Table is selected as the batch parameter and hit Next. Next to Batch Input Table click the down arrow and select all the individual spatial join layers created in step 2. Click Add.
2. Name the output feature class “AOO_Count_%Name%”.
3. Under Statistics Fields from the dropdown select the OBJECTID field and as the Statistic Type select Count.
4. Ensure all other parameters are left blank.
5. Click Run. This may take several hours depending on the number of layers being run.


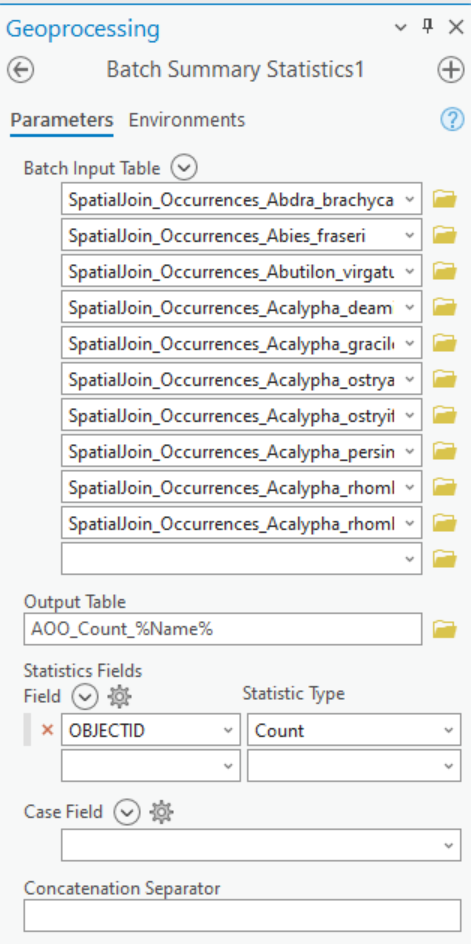


1. For single species processing: open the attribute table of the newly created area of occupancy count layer and area of occupancy will be listed under the “FREQUENCY” column.
2. For batch processing proceed to step 4 below.

Step 4: Use the *Merge* tool to create a single table listing area of occupancy for all species (only necessary for batch processing).

1. In the geoprocessing pane search for and open the *Merge* tool.
2. Next to Input Datasets click the down arrow and select all individual area of occupancy count layers created in step 3. Click Add.
3. Name the Output Dataset.
4. Ensure the Add source information to output box **is** **checked**. This ensures that each file name, and therefore species name, is included in the output table along with its area of occupancy.
5. Click Run.


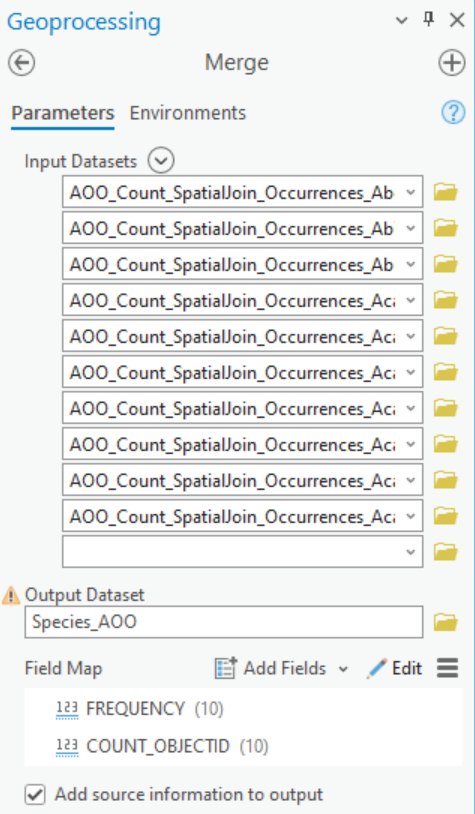


1. Open the new attribute table created. Area of occupancy for each species will be listed under the “FREQUENCY” column and the input file name which contains species name will be listed under the “MERGE_SRC” column.

**Using the NatureServe Rank Calculator to Generate a Rank**

The calculated rarity criteria can be individually, or batch uploaded to the NatureServe rank calculator to generate a rank. This calculator includes 10 criteria which fall under three categories: rarity, trends, and threats. A minimum of two criteria are needed when assigning ranks using the calculator. Criteria are entered and an S-rank is automatically assigned. The user cannot adjust any weightings or point values (NatureServe, 2020b; Faber-Langendoen et al., 2012).

Step 1: Export range extent, number of occurrences, and area of occupancy merge tables from ArcGIS Pro to Microsoft Excel

1. In ArcGIS Pro open the geoprocessing pane by navigating to the ribbon at the top of the page. Under the Analysis tab in the Geoprocessing group select Tools.


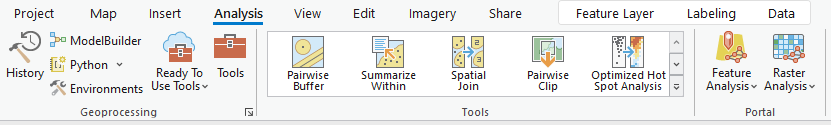


1. In the geoprocessing pane search for and open the *Table To Excel* tool.
2. Next to Input Table click the down arrow and select the three merge tables created for area of occupancy, number of occurrences, and range extent. Each table should contain the calculated criteria for all species. Click Add.
3. Name your Output Excel File and select where you want the file to be saved.
4. Click Run.


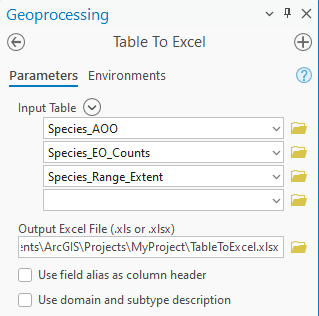


1. Open the newly created Excel file. The file should contain a separate sheet for each of the criteria (range, EO, and AOO).


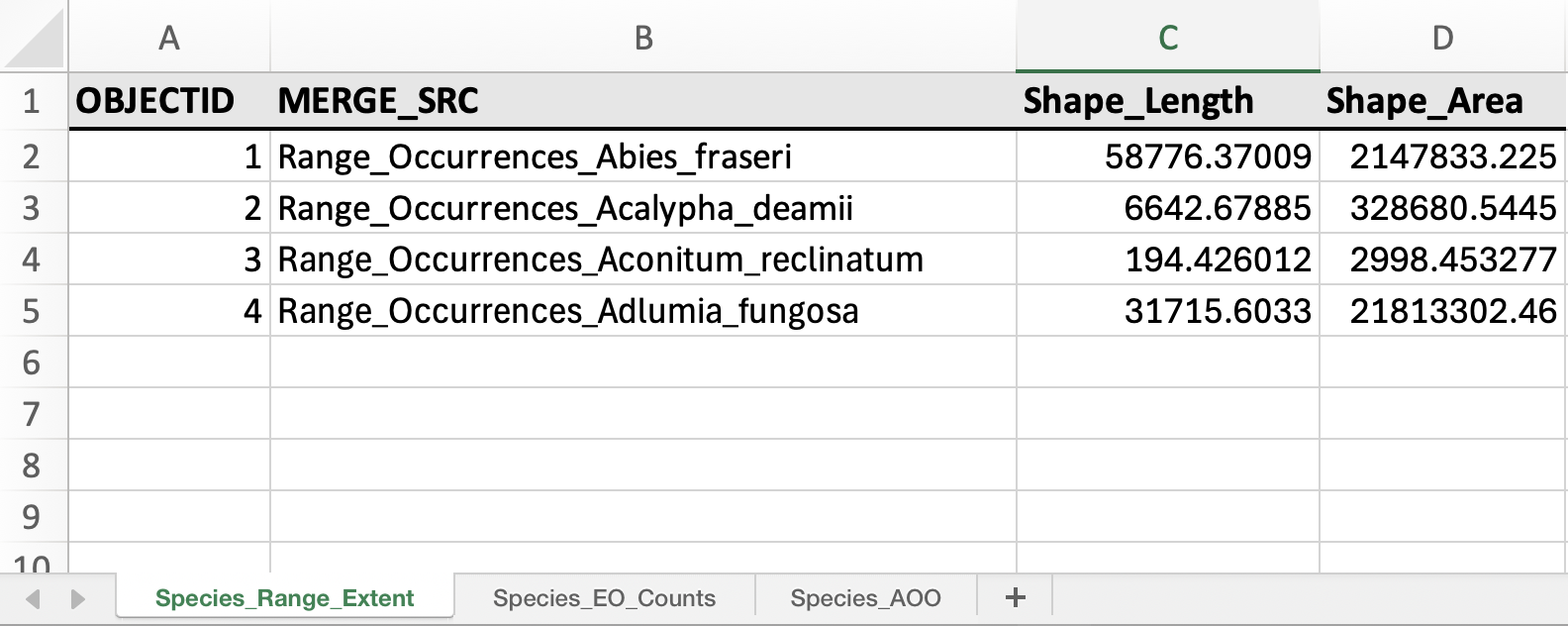


Step 2: Convert calculated values into rating codes

Values must be converted into the appropriate rating codes used by the NatureServe rank calculator based on where the values fall in designated ranges (Table 1). This can easily be done using Microsoft Excel (or equivalent).

1. Convert exported range extent values from ArcGIS to rating codes based on ranges found in Table 1.
   1. Range extent values first need to be converted from square meters to square kilometers by dividing values by 1,000,000. This can be done in Excel by creating a new column adjacent to the column containing range extent values and in the first cell entering the formula =D2/1000000 (assuming your range extent values are in column D and start in the second row). Pull the formula down by selecting the cell that contains the formula and clicking on the small square that appears in the bottom-right corner of the selected cell (this is called the fill handle). Drag the fill handle down until the formula is copied to all rows. Excel will automatically adjust the cell number in the formula to match the adjacent cell number.


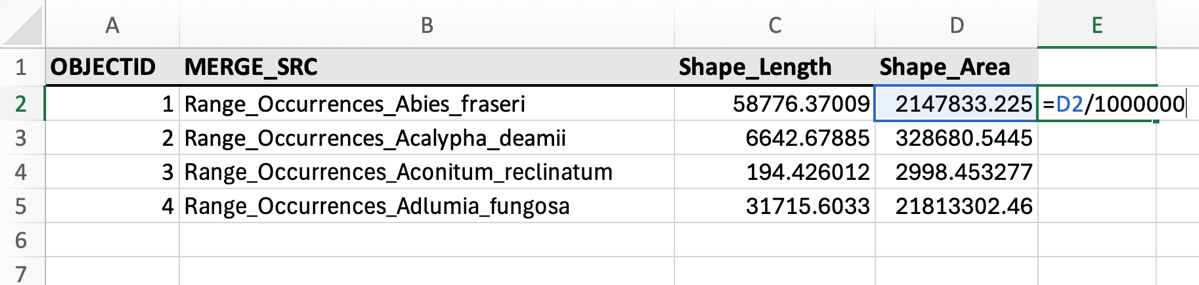


- 1. To convert the range extent values now in km^2^ to the appropriate rating code the IF formula can be used. This formula will return a value based on a specified condition. The ranges from Table 1 and their associated rating codes can be entered into the formula as the condition and the formula will return the appropriate rating code based on the range extent value in a specified cell. The following formula applies the ranges and their associated rating codes for range extent from Table 1 as the condition. This formula can be entered into the first cell adjacent to the column containing range extent in km^2^ and pulled down to all rows using the fill handle.

=IF(AND(E2>=0,E2<=99),"A",IF(AND(E2>=100,E2<=249),"B",IF(AND(E2>=250,E2<=999),"C",IF(AND(E2>=1000,E2<=4999),"D",IF(AND(E2>=5000,E2<=19999),"E",IF(AND(E2>=20000,E2<=199999),"F",IF(AND(E2>=200000,E2<=2499999),"G",IF(AND(E2>=2500000),"H","N/A"))))))))

This formula assumes that your range extent values in km^2^ are in column E and start in the second row.


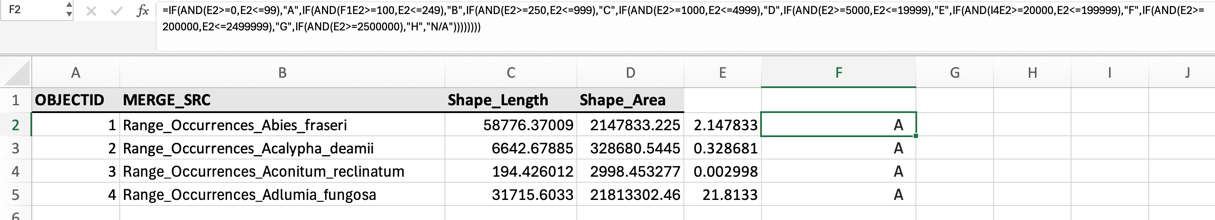


1. Convert exported number of occurrences values from ArcGIS to rating codes based on ranges found in Table 1.
   1. To convert the number of occurrences values to the appropriate rating code the IF formula can again be used. The following formula applies the ranges and their associated rating codes for number of occurrences from Table 1 as the condition. This formula can be entered into the first empty cell adjacent to the occupied columns and pulled down to all rows using the fill handle.

=IF(AND(B2>=1,B2<=5),"A",IF(AND(B2>=6,B2<=20),"B",IF(AND(B2>=21,B2<=80),"C",IF(AND(B2>=81,B2<=300),"D",IF(AND(B2>=301),"E","N/A")))))

This formula assumes that your number of occurrences values are in column B and start in the second row.


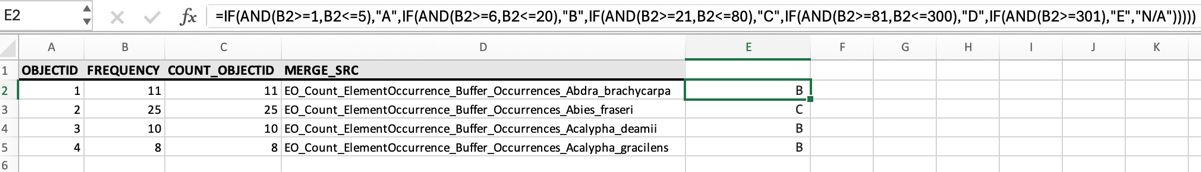


1. Convert exported area of occupancy values from ArcGIS to rating codes based on ranges found in Table 1.
   1. To convert the area of occupancy values to the appropriate rating code the IF formula can again be used. The following formula applies the ranges and their associated rating codes for area of occupancy from Table 1 as the condition. This formula can be entered into the first empty cell adjacent to the occupied columns and pulled down to all rows using the fill handle.

=IF(AND(B2=1),"A",IF(AND(B2=2),"B",IF(AND(B2>=3,B2<=5),"C",IF(AND(B2>=6,B2<=25),"D",IF(AND(B2>=26,B2<=125),"E",IF(AND(B2>=126,B2<=500),"F",IF(AND(B2>=501,B2<=2500),"G",IF(AND(B2>=2501,B2<=12500),"H",IF(AND(B2>12500),"I","N/A")))))))))

This formula assumes that your area of occupancy values are in column B and start in the second row.


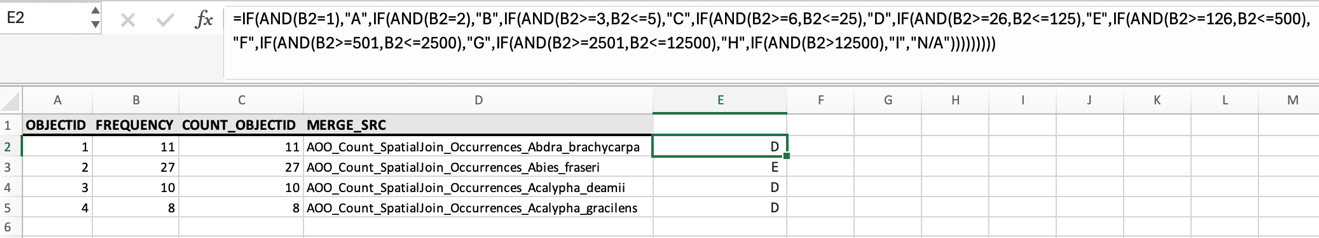


Table 1: Rating codes for rarity criteria (Master et al., 2012)

| Range Extent | Number of Occurrences | Area of Occupancy |
| --- | --- | --- |
| Z = 0 | Z = 0 | Z = 0 |
| A = <100 km^2^ | A = 1 – 5 | A = 1 |
| B = 100 – 250 km^2^ | B = 6 – 20 | B = 2 |
| C = 250 – 1,000 km^2^ | C = 21 – 80 | C = 3 – 5 |
| D = 1,000 – 5,000 km^2^ | D = 81 – 300 | D = 6 – 25 |
| E = 5,000 – 20,000 km^2^ | E = >300 | E = 26 – 125 |
| F = 20,000 – 200,000 km^2^ |  | F = 126 – 500 |
| G = 200,000 – 2,500,000 km^2^ |  | G = 501 – 2,500 |
| H > 2,500,000 km^2^ |  | H = 2,501 – 12,500 |
|  |  | I = >12,500 |

Step 3: Download the NatureServe rank calculator

1. Visit natureserve.org/products/conservation-rank-calculator and download the latest version of the rank calculator.
2. Enter your personal information and email. A download link will be sent to your email.
3. Click the download link and open the downloaded Microsoft Excel file (Note: you will need to enable macros for the file to work properly).
4. Select Enter

Step 4: Insert rating codes into NatureServe rank calculator (the following steps use Version 3.2)

1. Open the “Calculator Table” tab of the rank calculator Excel spreadsheet
2. Copy scientific names and paste them into Column C of the rank calculator titled “Species or Community Scientific Name”
3. Copy range extent rating codes and paste them into Column K of the rank calculator titled “Range Extent” (ensure the appropriate rating code is in the same row as its correct scientific name)
4. Copy area of occupancy rating codes and paste them into Column M of the rank calculator titled “Area of Occup 4-km^2^ grid cells” (ensure the appropriate rating code is in the same row as its correct scientific name)
5. Copy number of occurrences rating codes and paste them into Column O of the rank calculator titled “# Occur” (ensure the appropriate rating code is in the same row as its correct scientific name)
6. Ranks will auto-populate from the entered information in Column A titled “Calc Rank”


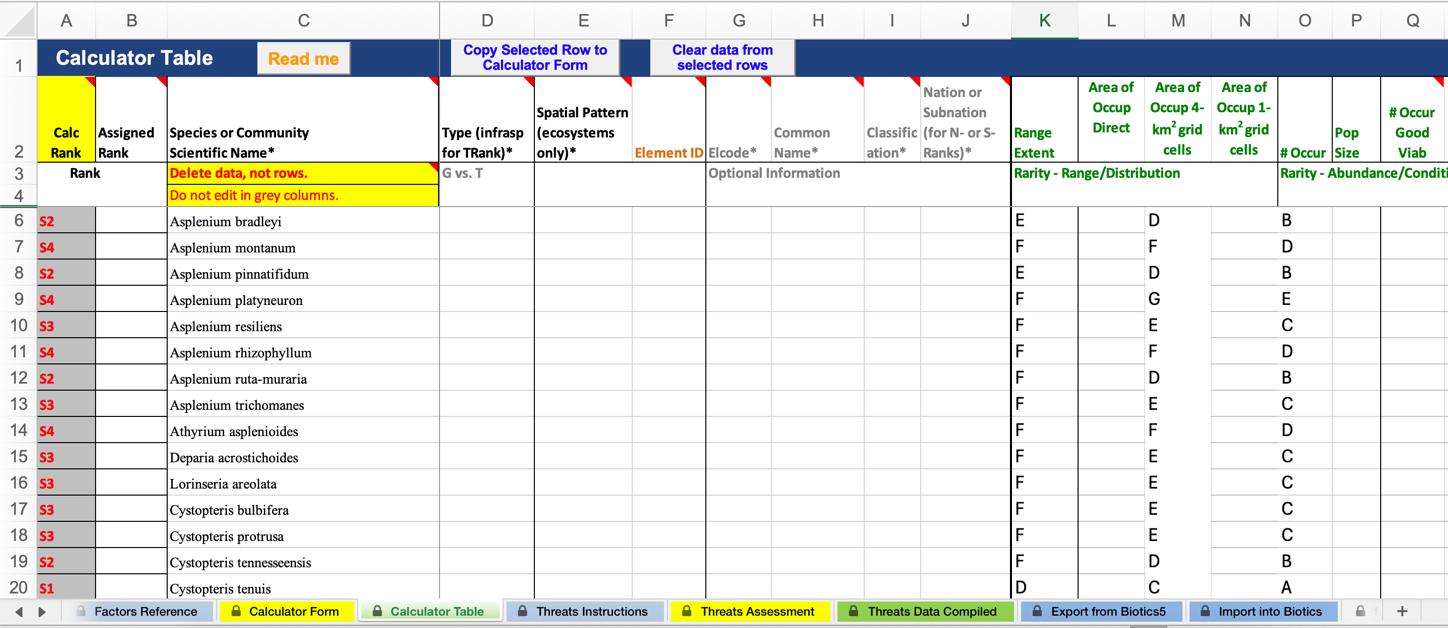


1. If available, information on threats, trends, or additional rarity criteria can be entered into the appropriate columns and the rank will automatically be adjusted. A breakdown of the rating codes for additional criteria can be found in the “Factors Reference” tab. The “Calculator Form” tab is useful for species-by-species assessments and allows for the selection of the appropriate rating code directly with the ranges for each code provided.

References

Faber-Langendoen, D., J. Nichols, L. Master, K. Snow, A. Tomaino, R. Bittman, G. Hammerson, et al. 2012. NatureServe Conservation Status Assessments: Methodology for Assigning Ranks. NatureServe, Arlington, Virginia, USA.

GBIF.org. 2024. Occurrence Search [online]. Website https://doi.org/10.15468/dl.7h2ehs [accessed 9 Jan 2024].

Master, L. L., D. Faber-Langendoen, R. Bittman, G. A. Hammerson, B. Heidal, L. Ramsay, K.

Snow, et al. 2012. NatureServe Conservation Status Assessments: Factors for Evaluating

Species and Ecosystem Risk. NatureServe, Arlington, Virginia, USA.

NatureServe. 2020a. Habitat-based Plant Element Occurrence Delimitation Guide. NatureServe, Arlington, Virginia, USA.

NatureServe. 2020b. NatureServe Conservation Status Assessments: Rank Calculator, Version

3.2. NatureServe, Arlington, Virginia, USA. Website

https://www.natureserve.org/conservation-tools/conservation-rank-calculator [accessed 9 Nov 2023].

SERNEC Data Portal. 2024. SERNEC Collections Search Result [online]. Website

https://sernecportal.org/portal/ [accessed 9 Jan 2024].
